# Anti-SARS-CoV-2 antibody-containing plasma improves outcome in patients with hematologic or solid cancer and severe COVID-19: a randomized clinical trial

**DOI:** 10.1038/s43018-022-00503-w

**Published:** 2022-12-29

**Authors:** Claudia M. Denkinger, Maike Janssen, Ulrike Schäkel, Julia Gall, Albrecht Leo, Patrick Stelmach, Stefan F. Weber, Johannes Krisam, Lukas Baumann, Jacek Stermann, Uta Merle, Markus A. Weigand, Christian Nusshag, Lars Bullinger, Jens-Florian Schrezenmeier, Martin Bornhäuser, Nael Alakel, Oliver Witzke, Timo Wolf, Maria J. G. T. Vehreschild, Stefan Schmiedel, Marylyn M. Addo, Felix Herth, Michael Kreuter, Phil-Robin Tepasse, Bernd Hertenstein, Mathias Hänel, Anke Morgner, Michael Kiehl, Olaf Hopfer, Mohammad-Amen Wattad, Carl C. Schimanski, Cihan Celik, Thorsten Pohle, Matthias Ruhe, Winfried V. Kern, Anita Schmitt, Hanns-Martin Lorenz, Margarida Souto-Carneiro, Mary Gaeddert, Niels Halama, Stefan Meuer, Hans-Georg Kräusslich, Barbara Müller, Paul Schnitzler, Sylvia Parthé, Ralf Bartenschlager, Martina Gronkowski, Jennifer Klemmer, Michael Schmitt, Peter Dreger, Katharina Kriegsmann, Richard F. Schlenk, Carsten Müller-Tidow

**Affiliations:** 1 Division of Infectious Disease and Tropical Medicine, Department of Infectious Diseases, Heidelberg University Hospital, Heidelberg, Germany; 2 Partner site Heidelberg University Hospital, German Center for Infection Research, Heidelberg, Germany; 3 Department of Internal Medicine V, Heidelberg University Hospital, Heidelberg, Germany; 4 NCT Trial Center, National Center for Tumor Diseases, Heidelberg University Hospital and German Cancer Research Center, Heidelberg, Germany; 5 Institute for Clinical Transfusion Medicine and Cell Therapy Heidelberg, Heidelberg, Germany; 6 Institute of Medical Biometry, University of Heidelberg, Heidelberg, Germany; 7 Department of Internal Medicine IV, Heidelberg University Hospital, Heidelberg, Germany; 8 Department of Anesthesiology, Heidelberg University Hospital, Heidelberg, Germany; 9 Department of Nephrology, University of Heidelberg, Heidelberg, Germany; 10 Department of Hematology, Oncology and Tumor Immunology, Charité – Universitätsmedizin Berlin, Berlin, Germany; 11 Department of Internal Medicine I, University Hospital Dresden and Faculty of Medicine Carl Gustav Carus of TU Dresden, Dresden, Germany; 12 Department of Infectious Diseases, West German Centre for Infectious Diseases, University Hospital Essen, University of Duisburg-Essen, Essen, Germany; 13 Department of Internal Medicine, Infectious Diseases, University Hospital Frankfurt, Goethe University Frankfurt, Frankfurt, Germany; 14 Department of Medicine, University Medical Center Hamburg–Eppendorf, Hamburg, Germany; 15 Partner site Hamburg–Lübeck–Borstel–Riems, German Center for Infection Research, Hamburg, Germany; 16 University Medical Center Hamburg-Eppendorf, Institute for Infection Research and Vaccine Development, Hamburg, Germany; 17 Pneumology and Critical Care Medicine, Thoraxklinik, University of Heidelberg and Translational Lung Research Center, Heidelberg, Germany; 18 Center for Interstitial and Rare Lung Diseases, Pneumology and Critical Care Medicine, Thoraxklinik, University of Heidelberg and German Center for Lung Research, Heidelberg, Germany; 19 Department of Medicine B, Gastroenterology and Hepatology, University Hospital Münster, Münster, Germany; 20 Medical Department I, Klinikum Bremen-Mitte, Bremen, Germany; 21 Department of Internal Medicine III, Klinikum Chemnitz, Chemnitz, Germany; 22 Department of Internal Medicine I, Frankfurt (Oder) General Hospital, Frankfurt (Oder), Germany; 23 Department of Hematology, Oncology, Palliative Care and Stem Cell Transplantation, Klinikum Hochsauerland, Meschede, Germany; 24 Department of Internal Medicine II, Klinikum Darmstadt, Darmstadt, Germany; 25 Department of Internal Medicine I, Klinikum Herford, Herford, Germany; 26 Department of Medicine II, Division of Infectious Diseases and Travel Medicine, University Medical Centre Freiburg, Freiburg, Germany; 27 Department of Medical Oncology, National Center for Tumor Diseases, Heidelberg University Hospital, Heidelberg, Germany; 28 Department of Translational Immunotherapy (D240), German Cancer Research Center, Heidelberg, Germany; 29 Helmholtz Institute for Translational Oncology, Mainz, Germany; 30 Department of Infectious Diseases, Virology, Heidelberg University Hospital, Heidelberg, Germany; 31 Department of Infectious Diseases, Molecular Virology, Heidelberg University Hospital, Heidelberg, Germany; 32 National Center for Tumor Diseases, Heidelberg, Germany

**Keywords:** SARS-CoV-2, Viral infection, Clinical trials, Cancer

## Abstract

Patients with cancer are at high risk of severe coronavirus disease 2019 (COVID-19), with high morbidity and mortality. Furthermore, impaired humoral response renders severe acute respiratory syndrome coronavirus 2 (SARS-CoV-2) vaccines less effective and treatment options are scarce. Randomized trials using convalescent plasma are missing for high-risk patients. Here, we performed a randomized, open-label, multicenter trial (https://www.clinicaltrialsregister.eu/ctr-search/trial/2020-001632-10/DE) in hospitalized patients with severe COVID-19 (*n* = 134) within four risk groups ((1) cancer (*n* = 56); (2) immunosuppression (*n* = 16); (3) laboratory-based risk factors (*n* = 36); and (4) advanced age (*n* = 26)) randomized to standard of care (control arm) or standard of care plus convalescent/vaccinated anti-SARS-CoV-2 plasma (plasma arm). No serious adverse events were observed related to the plasma treatment. Clinical improvement as the primary outcome was assessed using a seven-point ordinal scale. Secondary outcomes were time to discharge and overall survival. For the four groups combined, those receiving plasma did not improve clinically compared with those in the control arm (hazard ratio (HR) = 1.29; *P* = 0.205). However, patients with cancer experienced a shortened median time to improvement (HR = 2.50; *P* = 0.003) and superior survival with plasma treatment versus the control arm (HR = 0.28; *P* = 0.042). Neutralizing antibody activity increased in the plasma cohort but not in the control cohort of patients with cancer (*P* = 0.001). Taken together, convalescent/vaccinated plasma may improve COVID-19 outcomes in patients with cancer who are unable to intrinsically generate an adequate immune response.

## Abstract (web summary)

Müller-Tidow and colleagues perform a randomized clinical trial and show that administration of convalescent plasma improves COVID-19 outcome in patients with cancer who are unable to generate an adequate immune response.

## Main

The coronavirus disease 2019 (COVID-19)-associated risk of death is particularly high for patients with hematologic or solid cancer^1–3^, advanced age^4,5^ and other conditions^6,7^. Both humoral^8^ and cellular^9^ immunodeficiency contribute to unfavorable outcomes. Despite this, severe acute respiratory syndrome coronavirus 2 (SARS-CoV-2) vaccine availability and waning vaccine efficacy in these patients remain concerning^10,11^.

Few therapies improve outcomes in severe COVID-19 with impaired oxygenation^12^. Monoclonal antibodies as pre- or postexposure prophylaxis or as early treatment can reduce the risk of severe COVID-19 (refs. 13,14). Evidence for the benefit of monoclonal antibodies in patients requiring oxygen supplementation is missing^15^ or pending^16^.

Clinical trials on convalescent plasma therapy for COVID-19 have been mostly negative^17–24^. Relevant determinants causing heterogeneity in plasma efficacy were: (1) the timing from disease onset to therapy initiation, with early therapy being most effective^18^; and (2) titers of neutralizing antibodies^18,23,24^. Still, it is unknown whether patients without sufficient antibody response benefit from therapy with plasma from convalescent or vaccinated donors, but several subgroup analyses have pointed toward better outcomes with plasma therapy. In a Bayesian re-analysis of the Randomized Evaluation of COVID-19 Therapy (RECOVERY) trial, the subgroup of patients who had not yet developed an antibody response to SARS-CoV-2 appeared to have slightly better outcomes when treated with convalescent plasma^25^. A similar subgroup analysis of the Randomised, Embedded, Multi-factorial, Adaptive Platform Trial for Community-Acquired Pneumonia (REMAP-CAP) trial pointed toward a potential benefit for immunosuppressed patients^26^. Two observational propensity score-matched cohort studies in patients with hematological malignancies showed a marked decrease in mortality despite in parts delayed transfusion of convalescent plasma^27,28^.

Here, we performed a randomized controlled clinical trial with convalescent/vaccinated plasma in high-risk patients, including patients with cancer with severe COVID-19, and analyzed the association between plasma therapy and the response of neutralizing antibody titers in plasma recipients.

## Results

### Trial population

**Fig. 1:**
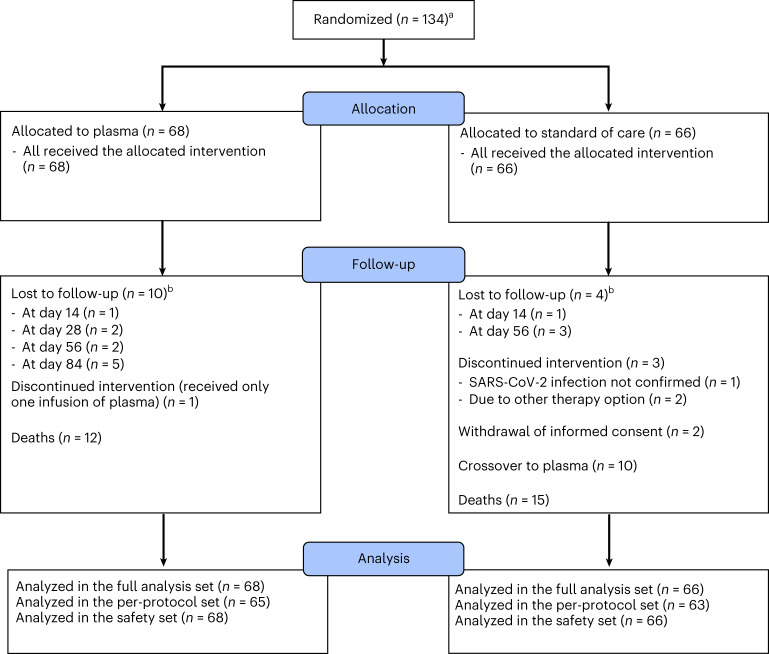
Consort diagram. Patient flow within the RECOVER trial. ^a^Two more patients were initially randomized but were later removed from the randomization tool and database. For one patient, informed consent was lost. The other patient withdrew consent and requested the deletion of all data. ^b^All patients lost to follow-up reached the primary endpoint, as lost to follow up occurred after discharge.

A total of 136 patients meeting eligibility criteria were randomized (Fig. 1). The inclusion criteria were: (1) PCR-confirmed infection with SARS-CoV-2 in a respiratory tract sample; (2) oxygen saturation on ambient air of ≤94% or a partial oxygen pressure − inspired oxygen fraction ratio of <300 mmHg; (3) provision of written informed consent; and (4) meeting at least one high-risk criterion to define the patient group (see the study protocol described in the Supplementary Information):

- Group 1 (cancer): patients with pre-existing or concurrent hematological cancer and/or receiving active cancer therapy for any cancer (including chemotherapy, radiotherapy and surgical treatments) within the past 24 months
- Group 2 (immunosuppression): patients experiencing chronic immunosuppression, either pharmacological or due to underlying diseases not meeting group 1 criteria
- Group 3 (lymphopenia/elevated d-dimers): patients aged >50 years and ≤75 years and not meeting group 1 or 2 criteria who had lymphopenia (<0.8 × 10^9^ cells per liter) and/or d-dimers (>1 µg ml^−1^)
- Group 4 (age >75 years): patients aged >75 years and not meeting group 1, 2 or 3 criteria

**Extended Data Fig. 1:**
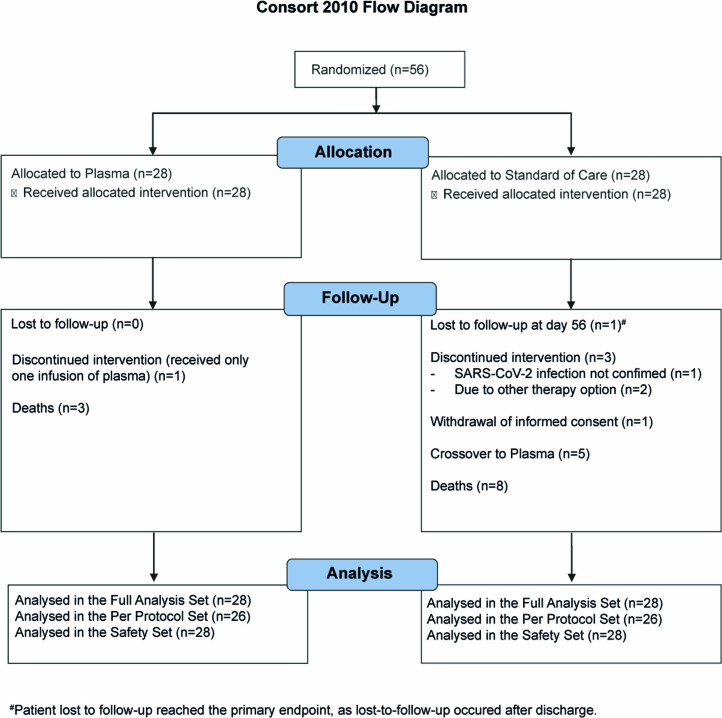
Consort diagram for group-1 (hematological and solid cancer). Consort diagram for group 1 (hematological and solid cancer).

Two patients were excluded—one due to the absence of a signed informed consent and the other due to withdrawal of consent after signature. Thus, 134 patients were enrolled and randomized between 3 September 2020 and 20 January 2022. A total of 68 patients were assigned to the plasma arm and 66 were assigned to the control arm. Eligible patients underwent randomization into the experimental (plasma) or control arm at a 1:1 ratio using block randomization for the patient group strata defined above (groups 1–4). Patients in the plasma cohort received at least one unit of ABO-compatible plasma, and ten control patients crossed over to the plasma group at day 10 after randomization. Plasma donor eligibility required high titers of neutralizing antibody activity in a live virus neutralization assay (titers ≥ 1:80; <20% of potential donors) (see the information on plasma donation and the neutralization assay used for the determination of titers in donors in the Supplementary Information, as well as Extended Data Fig. 6 and Supplementary Table 4). Recruitment was stopped on 20 January 2022, after enrollment of 77% of the target population (Methods). The average age was 69 years (range = 36–95 years) (Table 1 and Supplementary Table 5) and 43 patients were female (32.1%). Eastern Cooperative Oncology Group performance status (median = 2; interquartile range (IQR) = 2–3), clinical frailty scale (median = 3; IQR = 2–4) and time from symptom onset to randomization (median = 7.0 d; IQR = 4.0–10.0 d) were similar in both arms. Only 11.2% of patients were fully vaccinated. The allocation of patients to the predefined high-risk patient groups was: 42% for group 1 (*n* = 56; Extended Data Fig. 1), 12% for group 2 (*n* = 16), 27% for group 3 (*n* = 36) and 19% for group 4 (*n* = 26) (Table 1; group details in Supplementary Table 5). The most common cancers were B cell malignancies (*n* = 20), acute myeloid leukemia/myelodysplastic syndrome (*n* = 12), myeloma (*n* = 11) and solid cancer (*n* = 9). Two patients suffered from Hodgkin’s lymphoma and one patient each suffered from chronic myeloid leukemia or T cell lymphoma (Table 1). The most common cause of chronic immunosuppression in group 2 was solid organ transplantation (*n* = 12). In group 3, 27 patients showed lymphopenia and 21 patients had elevated d-dimers, whereas both criteria were present in 12 patients.

**Table 1:** Patient and treatment characteristics for the overall patient collective for both treatment arms combined and each separately

Characteristic	All (*n* = 134)	Control (*n* = 66)	Plasma (*n* = 68)
General			
Mean ± s.d. age (years)	68.5 ± 11.3	69.7 ± 10.5	67.4 ± 12.1
Sex			
Male	91 (67.9%)	46 (69.7%)	45 (66.2%)
Female	43 (32.1%)	20 (30.3%)	23 (33.8%)
Ethnic origin			
Asian	1 (0.7%)	1 (0.7%)	0 (0%)
Caucasian/White	130 (97.0%)	64 (97.0%)	66 (97.1%)
Hispanic	2 (1.5%)	0 (0%)	2 (2.9%)
Other	1 (0.7%)	1 (1.5%)	0 (0%)
Median (25th percentile, 75th percentile) time from symptom onset to randomization (d)^a^	7.0 (4.0, 10.0)	7.0 (4.0, 10.0)	7.0 (5.0, 10.0)
Comorbidities			
Chronic lung disease	37 (27.6%)	20 (30.3%)	17 (25.0%)
Cardiovascular disease	94 (70.1%)	47 (71.2%)	47 (69.1%)
Chronic liver disease	15 (11.2%)	11 (16.7%)	4 (5.9%)
Rheumatic/immunologic disease	16 (11.9%)	8 (12.1%)	8 (11.8%)
Organ transplant	17 (12.7%)	10 (15.2%)	7 (10.3%)
Diabetes	34 (25.4%)	19 (28.8%)	15 (22.1%)
Chronic kidney disease	35 (26.1%)	21 (31.8%)	14 (20.6%)
Chronic kidney disease with hemodialysis	13 (9.7%)	9 (13.6%)	4 (5.9%)
Median (25th percentile, 75th percentile) clinical frailty scale score^a^	3.0 (2.0, 4.0)	3.0 (2.0, 4.0)	3.0 (2.0, 4.0)
WHO performance status^a^			
ECOG = 0	3 (2.3%)	1 (1.5%)	2 (3.0%)
ECOG = 1	27 (20.5%)	12 (18.5%)	15 (22.4%)
ECOG = 2	51 (38.6%)	29 (44.6%)	22 (32.8%)
ECOG = 3	35 (26.5%)	16 (24.6%)	19 (28.4%)
ECOG = 4	16 (12.1%)	7 (10.8%)	9 (13.4%)
Cancer^b^			
All entities	56 (41.8%)	28 (42.4%)	28 (41.2%)
B cell non-Hodgkin lymphoma/chronic lymphocytic leukemia	18 (32.1%)	7 (25.0%)	11 (39.3%)
Acute myeloid leukemia/myelodysplastic syndromes	12 (21.4%)	8 (28.6%)	4 (14.3%)
Myeloma	11 (19.6%)	6 (21.4%)	5 (17.9%)
B cell acute lymphoblastic leukemia	2 (3.6%)	0 (0%)	2 (7.1%)
Hodgkin lymphoma	2 (3.6%)	1 (3.6%)	1 (3.6%)
Chronic myeloid leukemia	1 (1.8%)	1 (3.6%)	0 (0%)
T cell non-Hodgkin lymphoma	1 (1.8%)	1 (3.6%)	0 (0%)
Solid tumor	9 (16.1%)	4 (14.3%)	5 (17.9%)
SARS-CoV-2 baseline			
Median (25th percentile, 75th percentile) percentage inhibition (as measured by NeutraLISA)^a^	9.3 (4.8, 26.2)	8.5 (4.0, 20.3)	10.2 (5.5, 28.8)
Mean ± s.d. Ct value on day of randomization/day 1^a^	23.6 ± 5.6	23.3 ± 5.2	23.9 ± 6.1
Study assessments			
7POS at randomization			
7POS = 3	26 (19.4%)	12 (18.2%)	14 (20.6%)
7POS = 4	80 (59.7%)	40 (60.6%)	40 (58.8%)
7POS = 5	28 (20.9%)	14 (21.2%)	14 (20.6%)
Laboratory			
Median (25th percentile, 75th percentile) WBC count (10^9^ cells per liter)	5.7 (3.7, 8.6)	6.1 (4.0, 8.9)	5.4 (3.6, 7.5)
Median (25th percentile, 75th percentile) lymphocytes (10^9^ cells per liter)^a^	0.6 (0.3, 0.9)	0.5 (0.3, 0.9)	0.6 (0.3, 0.8)
Median (25th percentile, 75th percentile) CRP (mg l^−1^)^a^	80.8 (42.5, 147.2)	85.0 (48.2, 138.7)	72.7 (39.8, 157.6)
Median (25th percentile, 75th percentile) LDH (U l^−1^)^a^	359.0 (277.0, 473.1)	368.5 (278.0, 497.0)	354.0 (277.0, 457.0)
Median (25th percentile, 75th percentile) d-dimer (mg l^−1^)^a^	1.3 (0.7, 2.1)	1.4 (0.7, 2.4)	1.1 (0.7, 1.6)
Median (25th percentile, 75th percentile) troponin (pg ml^−1^)^a^	17.2 (11.4, 32.0)	23.0 (10.5, 48.6)	15.9 (11.4, 25.3)
Treatment (including crossover day 10)			
Plasma received			
Convalescent plasma	67	6	61
Convalescent plus vaccinated plasma	7	3	4
Vaccinated plasma only	4	1	3
Other COVID-19 medication			
Anti-inflammatory	49 (36.6%)	22 (33.3%)	27 (39.7%)
Small-molecule antiviral	11 (8.2%)	3 (4.5%)	8 (11.8%)
Biologic antiviral	3 (2.2%)	1 (1.5%)	2 (2.9%)
Antibiotics	6 (4.5%)	3 (4.5%)	3 (4.4%)
Anticoagulants	2 (1.5%)	2 (3.0%)	0 (0%)
Other concomitant medication	9 (6.7%)	5 (7.6%)	4 (5.9%)

^a^Numbers were as follows (*n* ≠ 134): *n* = 116 for time from symptom onset to randomization, *n* = 127 for clinical frailty scale score, *n* = 132 for World Health Organisation (WHO) performance status, *n* = 119 for percentage inhibition (as measured by NeutraLISA), *n* = 119 for Ct values, *n* = 117 for lymphocytes, *n* = 132 for C-reactive protein (CRP), *n* = 127 for lactate dehydrogenase (LDH), *n* = 125 for d-dimer and *n* = 122 for troponin.

^b^Pre-existing or concurrent hematological malignancy and/or active cancer therapy (including chemotherapy, radiotherapy or surgery) within the last 24 months or less.

EGOG, Eastern Cooperative Oncology Group; WBC, white blood cell.

### Follow-up and primary endpoint

A clinical seven-point ordinal scale (7POS)^29,30^ was determined daily, which was defined as: (1) not hospitalized, with resumption of normal activities; (2) not hospitalized, but unable to resume normal activities; (3) hospitalized, but not requiring supplemental oxygen; (4) hospitalized and requiring supplemental oxygen; (5) hospitalized and requiring nasal high-flow oxygen therapy, noninvasive mechanical ventilation or both; (6) hospitalized and requiring extracorporeal membrane oxygenation, invasive mechanical ventilation or both; and (7) death^31^. At baseline, the 7POS was at a median of 4 (range = 3–5) and oxygen supplementation (through a nasal cannula or high-flow oxygen therapy) was required in *n* = 108 (80.6%) patients, with equal distribution in both arms.

**Fig. 2:**
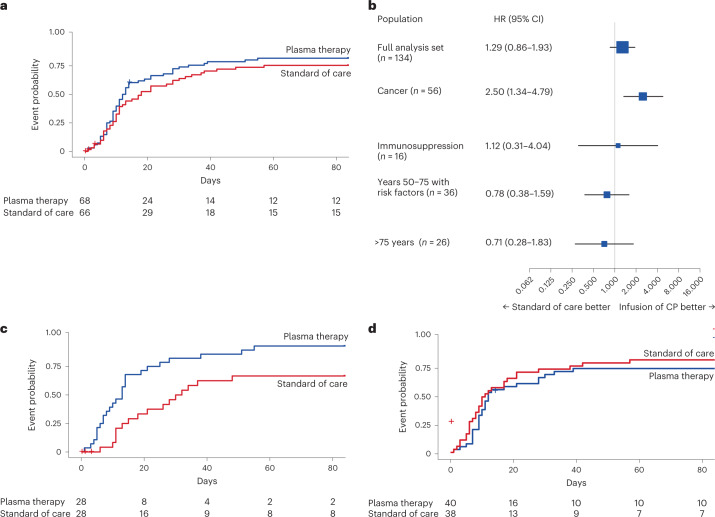
Primary endpoint (time to improvement of two points on the 7POS or live hospital discharge). **a**, Kaplan–Meier curve for the primary endpoint of a two-point improvement on the 7POS or live hospital discharge for the overall study cohort (groups 1–4) by plasma arm (blue) and control arm (red). The median time to improvement was 12.5 d (95% CI = 10–17) for the plasma arm and 18 d (95% CI = 11–28) for the control arm (log-rank *P* = 0.205). **b**, Forest plot with HRs for the primary endpoint overall (full analysis set) and by predefined subgroups. 95% CIs are provided in parentheses. The HRs are presented as the centers of the error bars. The error bars range from the lower to the upper 95% confidence limit. CP, convalescent plasma. **c**, Kaplan–Meier curve for the primary endpoint for group 1 by plasma arm (blue) and control arm (red). The median time to improvement was 13 d (95% CI = 7–14) for the plasma arm and 31 d (95% CI = 15–NA) for the control arm (log-rank *P* = 0.003). **d**, Kaplan–Meier curve for the primary endpoint for combined groups 2–4 by plasma arm (blue) and control arm (red). The median time to improvement was 12 d (95% CI = 10–28) for the plasma arm and 11 d (95% CI = 8–21) for the control arm (log-rank *P* = 0.3902). In **a**, **c** and **d**, the numbers of participants at risk are detailed below the Kaplan–Meier plot. See Extended Data Fig. 3 for separate data for groups 2–4. Source data

**Extended Data Fig. 2:**
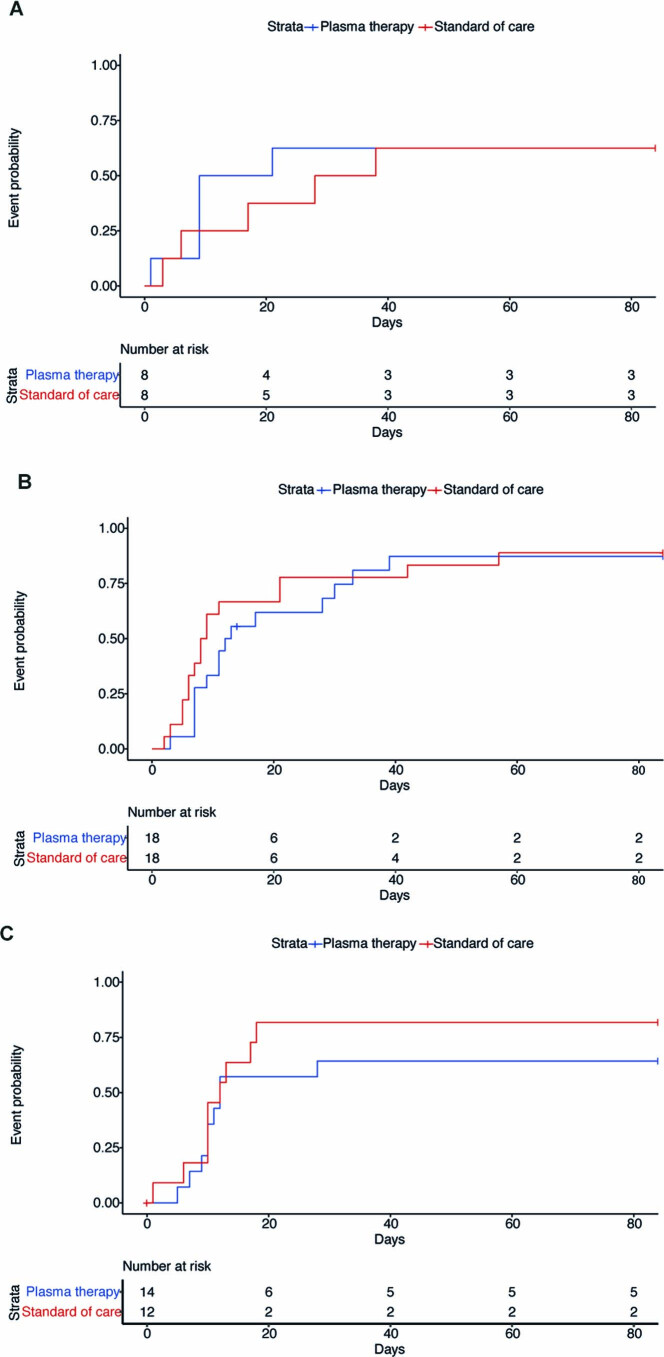
Kaplan Meier curves for time to improvement on 7-point ordinal scale or live hospital discharge for group-2 to −4. Kaplan Meier curves for cumulative probability of endpoint 2-point improvement on the 7-point ordinal scale or discharge by PLASMA (blue) and CONTROL (red) with number of subjects at risk below. (**a**) group 2; log-rank *p* = 0.860; (**b**) group 3; log-rank *p* = 0.472; (**c**) group 4; log-rank *p* = 0.452. Source data

In the full analysis set, the median time from randomization to improvement of two points on the 7POS or live hospital discharge was 12.5 d (95% confidence interval (CI) = 10–17) in the plasma arm and 18 d (95% CI = 11–28) in the control arm (hazard ratio (HR) = 1.29; 95% CI = 0.86–1.93; log-rank *P* = 0.205) (Fig. 2a,b). Pre-specified subgroup analyses revealed benefit in patients with cancer (group 1; *n* = 56). For patients with cancer, the median time to improvement was 13 d (95% CI = 7–14) for the plasma arm and 31 d (95% CI = 15–not available (NA)) for the control arm (HR = 2.50; 95% CI = 1.34–4.79; log-rank *P* = 0.003; Fig. 2b,c). Given potential confounders in age and gender distributions between the plasma and control arms, we adjusted for these variables in a sensitivity analysis. This resulted in a similar HR in group 1 (HR = 2.79; 95% CI = 1.35–5.94), supporting the beneficial role of plasma for patients with cancer. No significant differences between arms were observed in groups 2–4 (Fig. 2b,d, Extended Data Fig. 2 and Table 2).

**Table 2:** Outcome data for all patients combined and for group 1 and groups 2–4 separately

	All patients			Group 1 (cancer)			Groups 2–4 (other risk groups)		
	All (*n* = 134)	Control (*n* = 66)	Plasma (*n* = 68)	All (*n* = 56)	Control (*n* = 28)	Plasma (*n* = 28)	All (*n* = 78)	Control (*n* = 38)	Plasma (*n* = 40)
Overall improvement rate (95% CI)									
At 28 d		0.622 (0.503–0.742)	0.725 (0.615–0.825)		0.458 (0.286–0.673)	0.821 (0.663–0.935)		0.730 (0.582–0.859)	0.656 (0.509–0.798)
At 56 d		0.737 (0.623–0.840)	0.817 (0.716–0.899)		0.667 (0.481–0.841)	0.929 (0.796–0.987)		0.784 (0.642–0.898)	0.735 (0.592–0.861)
At 84 d		0.754 (0.641–0.853)	0.817 (0.716–0.899)		0.667 (0.481–0.841)	0.929 (0.796–0.987)		0.811 (0.672–0.917)	0.735 (0.592–0.861)
Median (95% Cl) time to improvement (d)		18 (11–28)	12.5 (10–17)		31 (15–NA)	13 (7–14)		11 (8–21)	12 (10–28)
Overall survival rate (95% Cl)									
At 28 d		0.835 (0.715–0.908)	0.864 (0.754–0.927)		0.710 (0.485–0.850)	0.929 (0.743–0.982)		0.918 (0.767–0.973)	0.815 (0.650–0.908)
At 56 d		0.765 (0.636–0.854)	0.832 (0.717–0.903)		0.710 (0.485–0.850)	0.893 (0.704–0.964)		0.803 (0.631–0.901)	0.787 (0.618–0.888)
At 84 d		0.748 (0.616–0.840)	0.815 (0.696–0.890)		0.665 (0.440–0.817)	0.893 (0.704–0.964)		0.803 (0.631–0.901)	0.753 (0.576–0.864)
Overall need for mechanical ventilation (*n* = 130)									
No ventilation	93 (71.5%)	44 (71.0%)	49 (72.1%)	38 (71.7%)	16 (64.0%)	22 (78.6%)	55 (71.4%)	28 (75.7%)	27 (67.5%)
Mechanical ventilation	37 (28.5%)	18 (29.0%)	19 (27.9%)	15 (28.3%)	9 (36.0%)	6 (21.4%)	22 (28.6%)	9 (24.3%)	13 (32.5%)

### Overall survival and other secondary endpoints

**Fig. 3:**
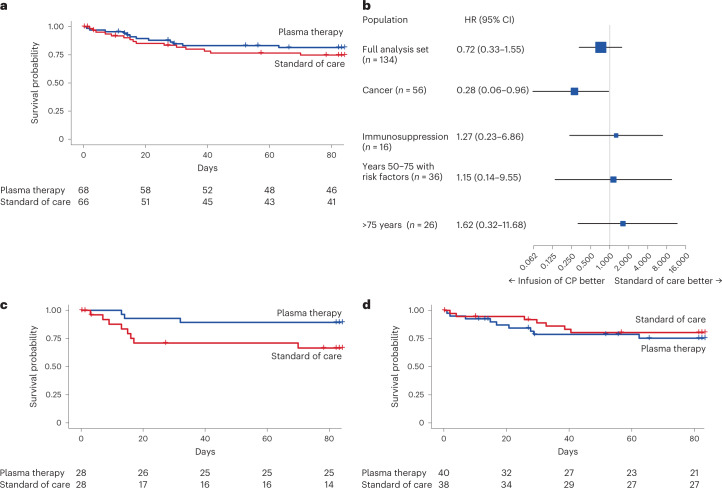
Secondary endpoint (overall survival). **a**, Kaplan–Meier curve for survival probability for the overall study cohort (groups 1–4) by plasma arm (blue) and control arm (red) (log-rank *P* = 0.403). **b**, Forest plot with HRs for survival probability overall and by predefined subgroups. 95% CIs are provided in parentheses. The HRs are presented as the centers of the error bars. The error bars range from the lower to the upper 95% confidence limit. **c**, Kaplan–Meier curve for survival probability for group 1 by plasma arm (blue) and control arm (red) (log-rank *P* = 0.042). **d**, Kaplan–Meier curve for survival probability for combined groups 2–4 by plasma arm (blue) and control arm (red) (log-rank *P* = 0.555). In **a**, **c** and **d**, the numbers of participants at risk are detailed below the Kaplan–Meier plot. See Extended Data Fig. 4 for separate data for groups 2–4. Source data

**Extended Data Fig. 3:**
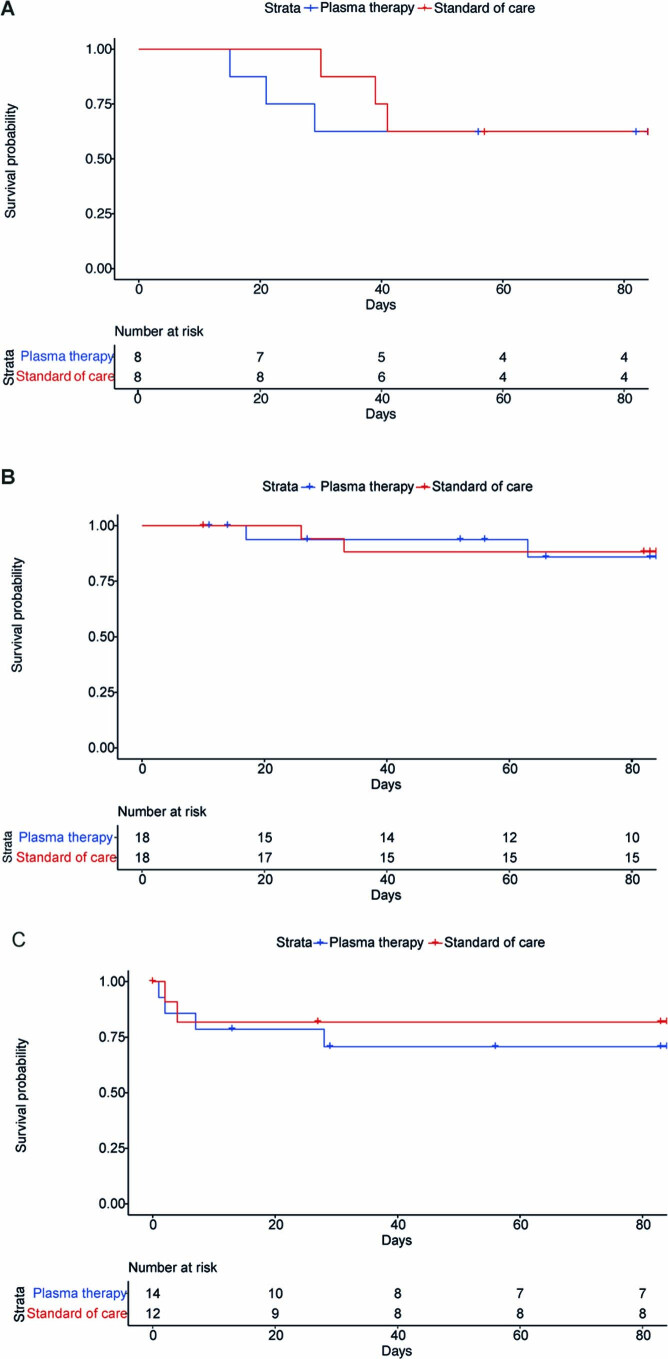
Kaplan Meier curves for Survival for group-2 to −4. Kaplan Meier curves for endpoint event survival by PLASMA (blue) and CONTROL (red) with number of subjects at risk. (**a**) group 2; log-rank *p* = 0.774; (**b**) group 3; log-rank *p* = 0.892 ; (**c**) group 4; log-rank *p* = 0.571. Source data

Overall, *n* = 27 patients died and no significant difference was seen for overall survival according to randomization (HR = 0.72; 95% CI = 0.33–1.55; log-rank *P* = 0.403) (Fig. 3a). In the cancer group (group 1), improved overall survival was observed in the plasma arm compared with the control arm (HR = 0.28; 95% CI = 0.06–0.96; log-rank *P* = 0.042) (Fig. 3b,c). The treatment arms of groups 2–4 did not differ in survival (Fig. 3b,d, Extended Data Fig. 3 and Table 2).

**Extended Data Fig. 4:**
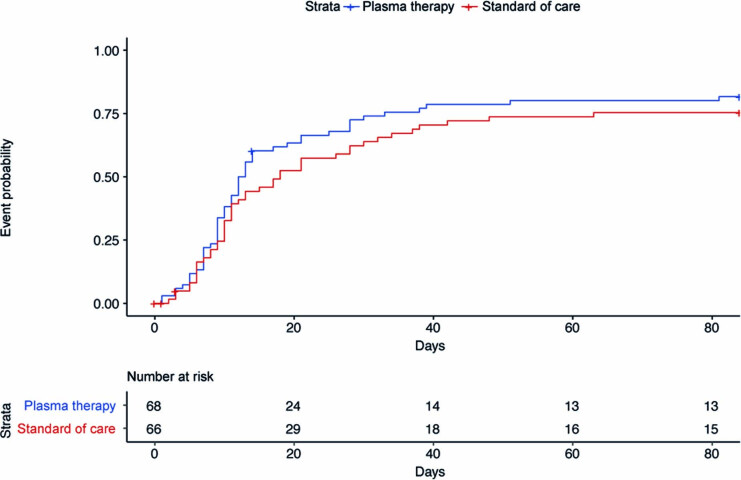
Kaplan Meier curves for Time to discharge overall. Kaplan Meier curves for cumulative endpoint event discharge from hospital by PLASMA (blue) and CONTROL (red) with number of subjects at risk ; log-rank *p* = 0.217. Source data

The time to discharge did not differ (HR = 1.28; 95% CI = 0.86–1.91; log-rank *P* = 0.217) in the overall study population (12.5 d (95% CI = 10–17) for the plasma arm versus 18 d (95% CI = 11–28) for the control arm) (Extended Data Fig. 4). Discharge occurred earlier in group 1 for the plasma arm (median = 13 d; 95% CI = 8–14) versus the control arm (median = 31 d; 95% CI = 15–NA) (HR = 2.50; 95% CI = 1.34–4.78; log-rank *P* = 0.003).

Mechanical ventilation was initiated in 28.5% of patients. No significant difference was observed between the treatment groups (27.9% (95% CI = 18.7–39.6) for the plasma arm versus 29% (95% CI = 19.2–41.3) for the control arm; odds ratio (OR) = 0.95 (95% CI = 0.44–2.06); *P* = 0.892) or within the subgroups (Table 2). The outcome for patients who crossed over was not substantially different from that for other patients in the control arm.

### Neutralizing antibody titers

**Fig. 4:**
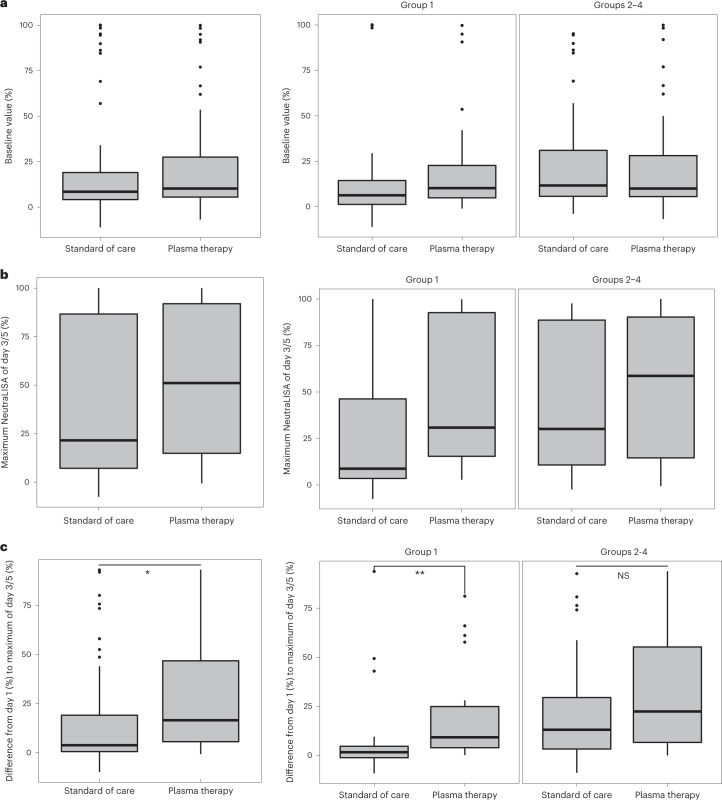
SARS-CoV-2 neutralizing activity in patient plasma. **a**, Baseline neutralizing activity in the overall analysis set (*n* = 56 for the control arm and *n* = 63 for the plasma arm), group 1 (*n* = 24 for the control arm and *n* = 25 for the plasma arm) and groups 2–4 (*n* = 32 for the control arm and *n* = 38 for the plasma arm), as measured by a surrogate inhibition assay on day 1 (after randomization and before plasma treatment). **b**, Highest levels of neutralizing activity on day 3/5 in the overall analysis set (*n* = 58 for the control arm and *n* = 64 for the plasma arm), group 1 (*n* = 25 for the control arm and *n* = 26 for the plasma arm) and groups 2–4 (*n* = 33 for the control arm and *n* = 38 for the plasma arm), as measured by a surrogate inhibition assay. **c**, Increase in neutralizing activity, analyzed as the percentage difference in neutralizing activity as measured by a surrogate inhibition assay on day 1 (after randomization and before plasma treatment) compared with the highest level from day 3/5, in the overall analysis set (*n* = 55 for the control arm and *n* = 62 for the plasma arm; *P = 0.012, two-sided van Elteren test stratified for patient group), group 1 (*n* = 24 for the control arm and *n* = 25 for the plasma arm; **P = 0.001, two-sided Wilcoxon signed-rank test) and groups 2–4 (*n* = 31 for the control arm and *n* = 37 for the plasma arm; *P* = 0.724 (not significant (NS)), two-sided van Elteren test stratified for patient group). In all panels, the boxplots indicate the IQR and the whisker length is limited to 1.5 times the IQR. Medians are indicated as horizontal lines within the boxes. Source data

**Extended Data Fig. 5:**
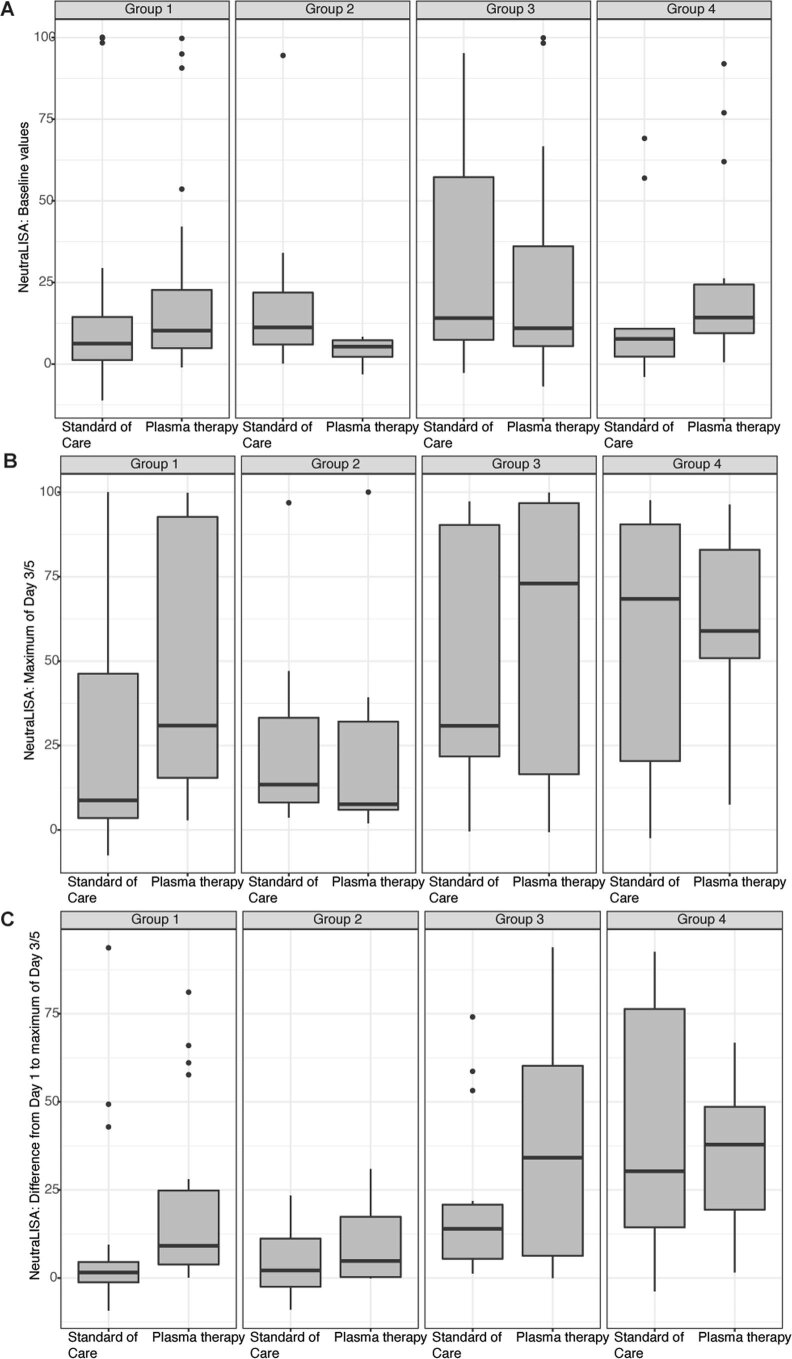
NeutraLISA measurement in the four subgroups. (**a**) Baseline neutralizing activity in group-1 (CONTROL: n = 24, PLASMA: n = 25), group-2 (CONTROL: n = 8, PLASMA: n = 6), group-3 (CONTROL: n = 14, PLASMA: n = 18) and group-4 (CONTROL: n = 9, PLASMA: n = 14) measured by a surrogate inhibition assay on day 1 (after randomization and prior to plasma treatment). Boxplots indicate the interquartile range and whisker length is limited to 1.5 times the interquartile range. Medians are indicated within the boxes. (**b)** Highest levels of neutralizing activity on day 3/5 in group-1 (CONTROL: n = 25, PLASMA: n = 26), group-2 (CONTROL: n = 8, PLASMA: n = 7), group-3 (CONTROL: n = 15, PLASMA: n = = 18) and group-4 (CONTROL: n = 10, PLASMA: n = 13) measured by a surrogate inhibition assay on day 1 (after randomization and prior to plasma treatment). Boxplots indicate the interquartile range and whisker length is limited to 1.5 times the interquartile range. Medians are indicated within the boxes. (**c**) Increase in neutralizing activity analyzed as the percent difference in neutralizing activity as measured by a surrogate inhibition assay on day 1 (after randomization and prior to plasma treatment) and compared to the highest level of day 3/5 in group-1 (CONTROL: n = 24, PLASMA: n = 25), group-2 (CONTROL: n = 8, PLASMA: n = 6), group-3 (CONTROL: n = 14, PLASMA: n = 18) and group-4 (CONTROL: n = 9, PLASMA: n = 13) measured by a surrogate inhibition assay on day 1 (after randomization and prior to plasma treatment). Boxplots indicate the interquartile range and whisker length is limited to 1.5 times the interquartile range. Medians are indicated within the boxes.

At the time of randomization, the average percentage inhibition of SARS-CoV-2 virus measured with the surrogate neutralizing enzyme-linked immunosorbent assay was 9.3% (IQR = 4.8–26.2; 10.2% (IQR = 5.5–28.8) for the plasma arm versus 8.5% (IQR = 4.0–20.3) for the control arm) (Fig. 4a and Supplementary Table 6). Neutralizing activity increased over time in both arms (Fig. 4b,c and Extended Data Fig. 5). The highest levels at day 3/5 were overall higher in the plasma cohort (51.1% (IQR = 14.7–92.5) for the plasma cohort compared with 21.6% (IQR = 7.2–87.3) for the control cohort) (Fig. 4b,c). In patients with cancer, the neutralizing activity did not increase over time in the absence of plasma therapy. In contrast, plasma therapy increased the neutralizing activity in patients with cancer who had higher levels on day 3/5 (group 1; 30.9% (IQR = 15.4–98.0) for the plasma arm compared with 8.8% (IQR = 3.5–46.3) for the control arm; Fig. 4c and Extended Data Fig. 5). Accordingly, for group 1, the median difference from day 3/5 to baseline differed significantly in the plasma arm (9.1% (IQR = 3.8–24.9)) compared with the control arm (1.6% (IQR = −1.5–4.7) (*P* = 0.001; Fig. 4c, left). In groups 3 and 4, neutralizing antibodies were already present at the time of study inclusion (Extended Data Fig. 5) and titers further increased over time regardless of the therapy arm. Thus, there was no benefit in neutralizing antibody titers for group 3 and 4 patients treated with plasma. Of note, in the few patients included in group 2 (immunosuppression), titers of neutralizing antibodies were low at the time of inclusion and remained low regardless of therapy arm (Extended Data Fig. 5).

### Adverse events

Adverse events observed after plasma administration were in accordance with published data^17^. No serious adverse events were observed related to plasma therapy. Adverse events are provided in Supplementary Table 7. Infusion reactions are described in Supplementary Table 8.

## Discussion

The results of this trial provide evidence that patients with cancer (group 1) who develop severe COVID-19 benefit from anti-SARS-CoV-2 plasma from convalescent/vaccinated donors and experience improved overall recovery. Although the size of group 1 was relatively small, with 56 patients, differences in the primary endpoint were substantial (13 versus 31 d) and are supported by earlier discharge and improved overall survival. The likelihood of improved outcomes upon plasma therapy was substantial for patients with cancer, with shortened time to the primary endpoint, time to discharge and also survival. In contrast, no benefits were observed in groups 2–4, pointing toward a specific benefit of vaccinated/convalescent plasma in patients with cancer.

These results from a specifically designed clinical trial are in line with two retrospective propensity-matched cohort analyses with a total of 244 patients treated with plasma^27,28^ and one prospective nonrandomized study using neutralizing monoclonal antibodies^14^.

Antivirals and monoclonal antibodies for COVID-19 are most effective in early disease stages and are usually not recommended beyond 5 d after symptom onset. The same was shown for early convalescent plasma therapy in a prehospital setting without the need for supplemental oxygen^32^. In our study, patients were randomized and treated on average within 7 d of symptom onset and few crossed over at day 10 after randomization. An even earlier intervention with plasma might further increase the efficacy in patients with cancer. Few therapies with proven efficacy are available for these patients at later time points, particularly for those presenting with impaired oxygenation, making plasma an attractive treatment approach even at advanced disease stages.

Unlike monoclonal antibodies^33^, convalescent plasma holds the potential to evolve in real time with the virus and retain activity against new variants. Furthermore, it does not involve patent fees and can be obtained within the regular blood donor pool. Since plasma from vaccinated donors contained higher levels of neutralizing antibodies^34^, and antibody titer has been associated with efficacy^35^, we included vaccinated donors once vaccination was widely available (see Supplementary Table 4).

Our study has limitations. Compared with studies with nonselected patients, the overall cohort was relatively small. Also, while the overall cohort was well balanced, we observed imbalances between enrollment arms (for example, with respect to age, sex, comorbidities and therapy) in the subgroups. Therefore, we adjusted for the two variables (that is, age and sex) most likely to be associated with outcomes in a sensitivity analysis, which did not impact on the primary outcome in group 1. Another limitation could be the open-label design of our trial. However, the primary endpoint results for subgroups are supported by the secondary endpoint results for overall survival and neutralizing activity, showing the unique effectivity of plasma therapy in patients with cancer.

The group of patients with cancer was diverse, with most patients suffering from hematological malignancies. Thus, the conclusions might not be applicable to all types of cancer. Lastly, recruitment occurred over an extended time span with different virus variants and evolving standards of care. Nonetheless, randomization was in place and plasma was obtained during the respective waves of the pandemic. Our conclusions cannot formally be extended to novel variants not covered within the trial (starting with Omicron). While studies have suggested that neutralizing antibodies were broadly active before Omicron, the Omicron variant in particular has shown that variant-specific plasma would be important to control virus replication^33^.

There are several strengths of our trial. Plasma was obtained from donors with confirmed high titers of neutralizing antibodies, as indicated by the fact that <20% of patients in the donor pool met the criteria (≥1:80 titer and corresponding high saturation in the NeutraLISA; Extended Data Fig. 7). The relevant subgroups were predefined in the protocol. HRs and CIs indicated large effect sizes in group 1. Plasma therapy effects on neutralizing antibody levels matched clinical benefit, although causality cannot be proven. The inclusion of patient groups now known not to benefit from plasma (for example, groups 3 and 4) suggests that underlying disease characteristics determine the benefit of plasma therapy in patients with cancer.

We found that in patients with cancer an increase in neutralizing antibodies was observed after plasma infusion, which further supports the restriction of the beneficial effects of plasma to patients with limited ability to react to the antigen with a humoral response. While the subgroup analysis was exploratory, the effect sizes were substantial. Given the limited available effective treatment options for patients with cancer and the favorable safety profile, convalescent/vaccinated plasma should be considered. Further confirmation of the findings is expected to come from other larger trials (for example, REMAP-CAP).

Taken together, these data suggest that plasma therapy may improve outcomes in patients with cancer with severe COVID-19.

## Methods

### Ethics and regulatory requirements

This study was carried out in accordance with the Declaration of Helsinki and the International Conference on Harmonization and Good Clinical Practice (ICH-GCP) E6 (R2) guidelines. The study was approved by the Paul Ehrlich Institut (Federal Institute for Vaccines and Biomedicines) and the ethics committee of the Medical Faculty Heidelberg. Regulatory authority requirements with respect to plasma manufacturing, according to §67 Arzneimittelgesetz (Germany) and §13 GCP-V, were met.

### Study sites and trial eligibility

Fifteen trial sites in Germany enrolled study participants (ten university hospitals and five urban hospitals; Supplementary Tables 1 and 3). Adult patients requiring hospital admission for COVID-19 were assessed for eligibility irrespective of previous SARS-CoV-2 infection or vaccination status. The inclusion criteria were: (1) PCR-confirmed infection with SARS-CoV-2 in a respiratory tract sample; (2) oxygen saturation on ambient air of ≤94% or a partial oxygen pressure − inspired oxygen fraction ratio of mmHg; (3) provision of written informed consent; and (4) meeting at least one high-risk criterion to define the patient group:

- Group 1 (cancer): patients with pre-existing or concurrent hematological cancer and/or receiving active cancer therapy for any cancer (including chemotherapy, radiotherapy and surgical treatments) within the past 24 months
- Group 2 (immunosuppression): patients experiencing chronic immunosuppression, either pharmacological or due to underlying diseases not meeting group 1 criteria
- Group 3 (lymphopenia/elevated d-dimers): patients aged >50 years and ≤75 years and not meeting group 1 or 2 criteria, who had lymphopenia (<0.8 × 10^9^ cells per liter) and/or d-dimers (>1 µg ml^−1^).
- Group 4 (age >75 years): patients aged >75 years and not meeting group 1, 2 or 3 criteria

Inclusion criteria were consecutively checked for groups 1–4 in ascending order.

Patients with a history of reaction to blood products, patients requiring mechanical ventilation (including noninvasive ventilation), patients with selective immunoglobulin A deficiency and patients participating in another trial of investigational medicinal products were excluded. Further details on inclusion/exclusion are provided in the published protocol^31^. Modifications of the protocol and the statistical analysis plan are described in Supplementary Table 2.

**Extended Data Fig. 6:**
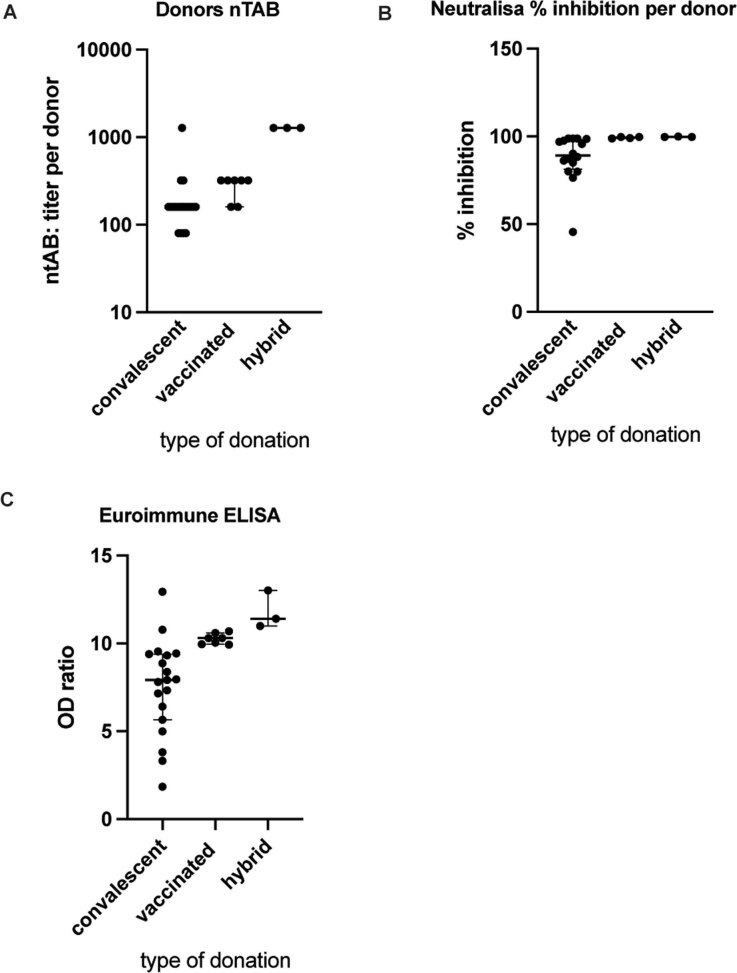
Column scatter plots for neutralization characteristics of donated plasma. (**a**) ntAB titers per plasma donor; convalescent donors (n = 19, median = 160 (IQR: 160-160)); vaccinated donors (n = 7, median = 320 (IQR: 160-320)); hybrid donors (n = 3, median = 1280 (IQR: 1280-1280)). (**b**) NeutraLISA assay result as % inhibition; convalescent donors (n = 19, median = 89.23 (IQR: 81.32-98.33)); vaccinated donors (n = 4, median = 99.38 (IQR:98.85-99.68)); hybrid donors (n = 3, median = 99.69 (IQR: 99.69-99.91)). (**c**) OD ratio determined by antibody values measured with the Euroimmune ELISA; convalescent donors (n = 19, median = 7.92 (IQR: 5.65-9.39)); vaccinated donors (n = 7, median = 10.30 (IQR: 9.96-10.60)); hybrid donors (n = 3, median = 11.40 (IQR:10.99-13.02)). Abbreviations: ntAB neutralizing Antibodies.

Eligible patients underwent randomization into the experimental (plasma) or control arm at a 1:1 ratio using block randomization for the patient group strata defined above (groups 1–4). Patients randomized into the control arm were offered to crossover on day 10 (plus a maximum of 2 d) after randomization in the absence of clinical improvement. Control arm patients received the standard of care as defined by the respective hospital at the time of trial inclusion. Patients in the plasma arm received two units of ABO-compatible plasma (238–337 ml each from two different donors) on the day of randomization (day 1) and on a later day. This was administered intravenously in addition to the standard of care. Convalescent and/or vaccinated donor plasma was obtained at the IKTZ Heidelberg. Plasma donor eligibility required high titers of neutralizing antibody activity in a live virus neutralization assay (titers ≥ 1:80; <20% of potential donors) (see the information on plasma donation and the neutralization assay used for determination of titers in donors in the Supplementary Information, as well as Extended Data Fig. 6 and Supplementary Table 4). Data collection and analysis were not performed blind to the conditions of the experiments.

### Procedures

After obtaining informed consent, a clinical 7POS^29,30^ was determined daily, which was defined as: (1) not hospitalized, with resumption of normal activities; (2) not hospitalized, but unable to resume normal activities; (3) hospitalized, but not requiring supplemental oxygen; (4) hospitalized and requiring supplemental oxygen; (5) hospitalized and requiring nasal high-flow oxygen therapy, noninvasive mechanical ventilation or both; (6) hospitalized and requiring extracorporeal membrane oxygenation, invasive mechanical ventilation or both; and (7) death^31^.

### Endpoints

The primary endpoint was defined as the time from randomization to a two-point improvement on the 7POS or live hospital discharge, whichever occurred first. Patients who withdrew their informed consent without a previous two-point improvement or live hospital discharge were censored at the respective date. Patients who were lost to follow-up were censored at the date of last contact. Administrative censoring was conducted at day 84 for all patients who were still alive but did not experience an improvement or discharge until day 84. The event ‘death from any cause’ was handled by censoring deceased patients without previous two-point improvement or live discharge at day 84 (in analogy to the approach of ref. 36). Using this approach ensures that deceased patients are considered as not improved over the whole observation period of 84 d. Secondary endpoints were overall survival (time from randomization until death from any cause, applying the same censoring rules as the primary endpoint for withdrawal of informed consent, loss to follow-up and administrative censoring at day 84), antibody titers, requirement of mechanical ventilation at any time during the hospital stay and time from randomization until live hospital discharge (applying the same censoring rules as the primary endpoint for withdrawal of informed consent, loss to follow-up and administrative censoring at day 84, as well as censoring patients who died from any cause at day 84 analogously to the primary endpoint).

### Statistics and reproducibility

The analysis of the primary endpoint was done via a log-rank test, stratified for the factor ‘patient group’. The event ‘death from any cause’ was handled by censoring those patients at day 84 (ref. 36). HRs were determined via Cox regression stratified by patient group (1–4). A post-hoc sensitivity analysis was performed using an adjusted Cox regression considering age and sex to account for differences observed in the distribution of these variables between study arms. Time to discharge was assessed analogously to the primary endpoint. Overall survival was assessed by means of a log-rank test and Cox regression, both stratified for patient group. Requirement of mechanical ventilation (yes versus no) was analyzed by means of a logistic regression model adjusting for the factors treatment and patient group, including all patients with more than 1 d of follow-up. Patients who died were accounted for as having received mechanical ventilation. For neutralizing antibodies, the difference between baseline and the highest value on day 3/5 was assessed to compare the plasma versus control arm titers stratified by patient group (1–4), and a van Elteren test was performed. Predefined subgroup analyses were conducted for each patient group, as well as an exploratory analysis of the treatment effect interaction between the patients in group 1 versus groups 2–4 combined. Complete case analyses were performed, and no imputation of missing data was conducted. Patients in the control arm with crossover at day 10 after randomization were analyzed according to initial group assignment in the control arm. A post-hoc analysis of patients in the control arm comparing the outcomes of patients with crossover and those without crossover was performed. Adverse events were summarized descriptively. The assumption of proportional hazards for the Cox regression models was graphically assessed by inspecting the Kaplan–Meier curves; otherwise, due to the exclusive use of nonparametric tests, no further assessments of the distribution of the underlying data were required. The analysis of efficacy endpoints was done in the full analysis set including all randomized patients, while the safety endpoints were analyzed according to the treatment actually received. The trial was designed to enroll 174 patients (87 per arm) to detect a HR of 1.6 for shortening the time to improvement of two points on the 7POS or live hospital discharge in the plasma arm compared with the control arm at a two-sided significance level of 5% with a power of 80%. Additional details are provided in the protocol and statistical analysis plan (see the study protocol and statistical analysis plan in the Supplementary Information). The statistical analysis plan was written while investigators were blinded to treatment allocation.

All findings, including clinical and laboratory data, were documented by the investigator or an authorized member of the study team in the patient’s medical record and in the electronic case report forms (ClinCase Software Version 2.7.0.3). A responsible monitor checked all flagged data and generated questions that were sent back to the responsible investigator. The investigator had to resolve all discrepancies. Further checks for plausibility, consistency and completeness of data were performed after completion of the study. Statistical analyses were performed using the software packages SAS version 9.4, R Base (version 4.0; https://r-project.org) and GraphPad Prism version 9.

### Early trial termination

The first patient was randomized on 3 September 2020. Enrollment fluctuated with SARS-CoV-2 incidence in Germany. In January 2022, the Omicron variant became dominant in Germany. The neutralizing activity of stored plasma against Omicron was unknown. Also, enrollment had slowed considerably following new guidelines (from the World Health Organisation and others) on convalescent plasma use. The data-monitoring board thus recommended to stop recruitment, which was enacted on 20 January 2022, after enrollment of 77% of the target population.

### Safety assessments

All adverse events were graded according to Common Terminology Criteria for Adverse Events version 5.0. Pharmacovigilance was performed according to the ICH-GCP E6 (R2) guidelines. An independent data-monitoring committee regularly assessed outcomes and serious adverse events.

### Laboratory analyses

**Extended Data Fig. 7:**
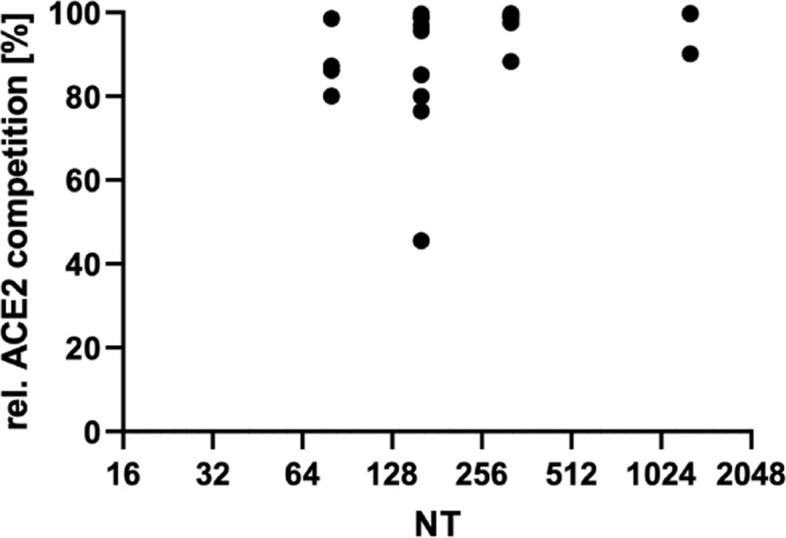
Correlation between live virus neutralization assay and NeutraLISA. NeutraLISA results on y-axis and live virus neutralization assay (as described in text S3) on the x-axis. Results show saturation of ACE2 competition in the NeutraLISA with titers of 1:80 and above.

Standard laboratory tests were performed locally. Reverse transcription PCR from nose and throat swabs for SARS-CoV-2 and antibody determination were performed at the Department of Infectious Diseases, Virology, Heidelberg University Hospital. NeutraLISA assay (Euroimmun) measures serum competition with angiotensin-converting enzyme 2–S1 subunit binding and was used as a surrogate for neutralizing SARS-CoV-2 antibody activity in plasma. A live virus neutralization assay was performed as previously described^37^. A live virus neutralization assay and NeutraLISA correlation for donor plasma is provided in Extended Data Fig. 7.

### Trial registration

This trial was registered with EudraCT number 2020-001632-10 on 4 April 2020.

### Reporting summary

Further information on research design is available in the Nature Portfolio Reporting Summary linked to this article.

## Supplementary information

Supplementary Information Statistical analysis plan, study protocol and consort checklist.

Reporting Summary

Supplementary Tables Supplementary Tables 1–8.

## Data Availability

All of the data supporting the findings of this study are available from the corresponding author upon reasonable request. Please contact Carsten.müller-tidow@med.uni-heidelberg.de for data availability. Source data are provided with this paper.

## Source data

Source Data Fig 2–4 and Source Data Extended Data Fig 2–5 Source data for Fig. 2 (primary endpoint: time to improvement in patients with cancer and in subgroups 2–4), source data for Fig. 3 (secondary endpoint: overall survival in patients with cancer and in subgroups 2–4), source data for Fig. 4 and Extended Data Fig. 5 (baseline neutralizing activity and maximum neutralizing activity on day 3/5 in all patients, in group 1 and in groups 2–4 combined), source data for Extended Data Fig. 2 (primary endpoint: time to improvement in subgroups), source data for Extended Data Fig. 3 (primary endpoint: time to improvement in subgroups) and source data for Extended Data Fig. 4 (time to discharge in all patients).

## Code availability

Statistical analyses have been performed using the software package SAS Version 9.4 and R Base (version 4.0; https://r-project.org). No unique code has been developed. Please contact Carsten.müller-tidow@med.uni-heidelberg.de for code availability.
