# Supplementary material for: Anti-SARS-CoV-2 antibody-containing plasma improves outcome in patients with hematologic or solid cancer and severe COVID-19: a randomized clinical trial
Source: Nat Cancer. 2022 Dec 29;4(1):96–107. doi: 10.1038/s43018-022-00503-w (PMC9886549; doi:10.1038/s43018-022-00503-w)
Supplement: Supplementary file 1 — Statistical analysis plan, study protocol and consort checklist. [file 43018_2022_503_MOESM1_ESM.pdf]

nature cancer

Article https://doi.org/10.1038/s43018-022-00503-w

Anti-SARS-CoV-2 antibody-containing plasma improves outcome in patients with hematologic or solid cancer and severe COVID-19: a randomized clinical trial

In the format provided by the authors and unedited

Table of contents

1. Texts
    - Text supplement S1 Study protocol
    - Text supplement S2 Information on Plasma donation
    - Text supplement S3 Neutralization assay used for determination of titer in donors
    - Text supplement S4 Statistical analysis plan
2. Consort checklist

Text Supplement S1: Study protocol
Anti-SARS-CoV-2 antibody containing plasma improves outcome in patients with hematologic or solid cancer and severe COVID-19: a randomized clinical trial

Claudia Denkinger, Maike Janssen et al.

This supplement contains the following items:

1. Original (first) study protocol V1.2, 9th July 2020 page 2
2. Last (final) study protocol V3.0, 8th March 2021 page 57
3. Summary of all changes to the original study protocol page112
4. Statistical analysis plan V1.0 page 117
5. Changes to the SAP V1.0 page 142

Clinical Trial Protocol

A Randomized Open label Phase-II Clinical Trial with or without Infusion of Plasma from Subjects after Convalescence of SARS-CoV-2 Infection in High-Risk Patients with Confirmed Severe SARS-CoV-2 Disease

RECOVER (Study Acronym)

**Clinical TrialCode:**	**SARS-CoV-2CP-HD-001**
**EudraCT No.:**	**2020-001632-10**
**Clinicaltrials.gov:**	
**Clinical Phase:**	Phase II
**Version and date:**	Version 1.2 July 09th, 2020
**Sponsor:**	*Ruprecht-Karls-Universität Heidelberg, Medical Faculty*<br>Represented in law by Universitätsklinikum Heidelberg and its acting Commercial Director Ms. Katrin Erk<br>Im Neuenheimer Feld 672,<br>69120 Heidelberg, Germany

CONFIDENTIAL: This protocol contains confidential information and is intended solely for the guidance of the clinical investigation. This protocol may not be disclosed to parties not associated with the clinical investigation or used for any purpose without the prior written consent of the Principal Investigator/Coordinating Investigator.

Responsibilities

Coordinating Investigator
(Leiter der klinischen Prüfung*)

Prof. Dr. Carsten Müller-Tidow
University Hospital Heidelberg
Internal Medicine V
Hematology,Oncology and Rheumatology
Im Neuenheimer Feld 410
69120 Heidelberg
Phone: +49 6221 - 56 8001
Fax: +49 6221 - 56 33639
E-Mail:
Carsten.Mueller-Tidow@med.uni-heidelberg.de

* According to § 40 German Drug Law (AMG)

Sponsor

Ruprecht-Karls-Universität Heidelberg,
Medical Faculty
represented by Universitätsklinikum
Heidelberg and its Commercial Director
Ms Katrin Erk
Im Neuenheimer Feld 672
69120 Heidelberg, Germany
Phone: +49 (0)6221 56 7081
Fax: +49 (0)6221 56 5925
E-Mail: Katrin.Erk@med.uni-heidelberg.de

Co-Coordinating Investigators
(Medizinische Studien-Koordinatoren)

Prof. Dr. Uta Merle, Internal Medicine IV, HD
Prof. Dr. Markus Weigand, Anesthesiology, HD
Dr. Claudia Denkinger, Tropic Medicine, HD
Prof. Dr. Hans-Georg Kräusslich, Infectious Diseases, Virology, HD
Prof. Dr. Maria Vehreschild, Div. Infectious Dis, University Hospital Frankfurt
Prof. Dr. Lars Bullinger, Hematology/Oncology, Charite, Berlin

Scientific Coordination
(Wissenschaftliche Leiter)

Prof. Dr. Richard Schlenk, NCT Trial Center
Prof. Dr. Michael Schmitt, Internal Medicine V

Project Management
Dr. med Ulrike Schäkel, Internal Medicine V
Dr. Carine Djuika Fokou, NCT Trial Center

Biostatistician
Dr. Johannes Krisam
Institute of Medical Biometry and Informatics
Im Neuenheimer Feld 130.3
69120 Heidelberg
Phone: +49 6221 - 56 32630
Fax: +49 6221 - 56 4195
E-Mail: krisam@imbi.uni-heidelberg.de
and
Prof. Dr. Richard F. Schlenk
National Center for Tumor Diseases (NCT)
Trial Center
Im Neuenheimer Feld 130.3
69120 Heidelberg
Phone: +49 6221 - 56 6228

Trial Coordination

NCT Trial Center
Im Neuenheimer Feld 130/3
69120 Heidelberg, Germany
Phone: +49 (0)6221 56 6522
Fax: +49 (0)6221 56 5863
E-Mail: studienzentrale@nct-heidelberg.de

Fax: +49 6221 - 56 5863
E-Mail: richard.schlenk@nct-heidelberg.de

Clinical Monitoring and Pharmakovigilance
Coordination Centre for Clinical Trials (KKS)
Heidelberg University Hospital
Im Neuenheimer Feld 130/3
69120Heidelberg, Germany
Phone:+49 (0)6221 56-34507
Fax: +49 (0)6221 56-33508
E-Mail: V-KKS.SAE@med.uni-heidelberg.de
Karsten.thelen@med.uni-heidelberg.de

Data Management
Christina Klose and Jacek Stermann
Institute of Medical Biometry and Informatics (IMBI)
Im Neuenheimer Feld 130/3
69120 Heidelberg, Germany
Phone: +49 (0)6221 56- 37235
Fax: +49 (0)6221 56-4195
E-Mail: stermann@imbi.uni-heidelberg.de

BioBank Med V
Dr. med. Katharina Kriegsmann, MBA
Universitätsklinikum Heidelberg
Innere Medizin V: Hämatologie,Onkologie and Rheumatologie
Im Neuenheimer Feld 410
69120 Heidelberg
Tel: +49 6221 – 56 37238
Fax: +49 6221 – 56 8588
E-Mail: katharina.kriegsmann@med.uni-heidelberg.de

Labor AG Halama
PD Dr. Niels Halama
Med. Onkologie NCT
Im Neuenheimer Feld 460
69120 Heidelberg
Tel.:: +49 6221 – 56 38397
Fax: +49 6221 – 56 7225
E-Mail: niels.halama@nct-heidelberg.de

Zentrum für Infektiologie
Prof. Dr. H.-G. Kräusslich (Leiter)
Universitätsklinikum Heidelberg
Zentrum für Infektiologie
Department für Infektiologie, Virologie
Im Neuenheimer Feld 344
69120 Heidelberg
Tel: +49 6221 – 56 5001
Fax: +49 6221 – 56 5006
E-Mail: Hans-Georg.Kraeusslich@med.uni-heidelberg.de

Prof. Dr. Ralf Bartenschlager
AG Molekulare Virologie
Im Neuenheimer Feld 344
69120 Heidelberg
Tel: +49 6221 - 56 4225
Fax: +49 6221 - 56 4570
E-Mail: ralf.bartenschlager@med.uni-heidelberg.de

Protocol Writing Committee:

Heidelberg: Lead: Prof. Dr. Richard Schlenk, Head, NCT and Hematology Study Center, and in alphabetical order (all University Heidelberg, if not otherwise specified)
Prof. Dr. Ralf Bartenschläger, DKFZ Heidelberg
Prof. Dr. Claudia Denkinger, Head, Division of Tropical Medicine,
Dr. Carine Djuika Fokou, NCT Trial Center
Prof. Dr. Peter Dreger, Director of Stem Cell Program, Medicine V
Dr. Niels Halama, National Center for Tumor Diseases, NCT-Heidelberg
Dr. Irina Idler, Ethics and Patient Oriented Care in Oncology, NCT-Heidelberg
Prof. Dr. Dirk Jäger, National Center for Tumor Diseases, Heidelberg
Prof. Dr. Meinhard Kieser, Director, Institute of Medical Biometry and Informatics (IMBI)
Prof. Dr. Hans-Georg Kräusslich, Dean of the Medical Faculty, Director of the Infectious Disease Center
PD Dr. Katharina Kriegsmann, Resident, Head, Biobank Medicine V
Dr. Johannes Krisam, Institute of Medical Biometry and Informatics (IMBI)
Dr. Albrecht Leo, Transfusion Specialist, Head, Blood Bank, Vice-Director Institute for Clinical Transfusion Medicine and Cell Therapy (ITKZ) gGmbH,
PD Dr. Tobias Liebregts, Attending, ICU Hematology, Medicine V
Prof. Dr. Hannes Lorenz, Head, Division of Rheumatology, Medicine V
Prof. Dr. Uta Merle, Director, Department of Gastroenterology and Infectious Diseases
Prof. Dr. Stefan Meuer, Director, IKTZ gGmbH,
Prof. Dr. Carsten Müller-Tidow, Director, Medicine V
Dr. Caroline Pabst, Resident, Medicine V
Dr. Ulrike Schäkel, Resident, Study Center, Medicine V
PD Dr. Anita Schmitt, Transfusion Specialist, QP Vice-Head, GMP Core Facility, Medicine V
Prof. Michael Schmitt, QP, Head, GMP Core Facility, Medicine V
Dr. Margarida Souto Carneiro, Senior Research Fellow, Division of Rheumatology,
Dr. Stefan Weber, Resident, Division of Tropical Medicine
Prof. Dr. Markus Weigand, Director, Dept. of Anaesthesiology

Frankfurt :
Prof. Dr. Maria Vehreschild, Johann-Wolfgang-Goethe-University
Dr. Timo Wolf, Frankfurt, Johann-Wolfgang-Goethe-University
Prof. Dr. Hubert Serve, Johann-Wolfgang-Goethe-University

Berlin: Prof. Dr. Lars Bullinger, Director, Department of Hematology and Oncology, Charite

Summary

Convalescent plasma has been proposed to decrease mortality in patients with SARS, Ebola and in epidemic influenza. Evidence is based on case reports, retrospective analyses and small clinical trials. The timing, when best to give plasma is unclear. Convalescent plasma was used during the COVID-19 outbreak in the Hubei province. Plasma was usually applied in critically ill patients with severe ARDS that may restrict the potential benefit.

This trial will analyze the effects of convalescent plasma from recovered subjects in high-risk patients with SARS-CoV-2 infection. Plasma obtained from subjects after SARS-CoV-2 infection with SARS-CoV-2 antibodies will be used in this trial. Patients at high risk for a poor outcome due to underlying disease (group 1, pre-existing or concurrent hematological malignancy or stem cell transplantation and/or active cancer therapy (incl. chemotherapy, radiotherapy, surgery) within the last 24 months or less; group 2, chronic immunosuppression not meeting the criteria of group 1; group 3, age ≥ 50 - 75 years and lymphopenia < 0.8 x G/l and/or D-dimer > 1µg/mL meeting neither the criteria of group 1 nor group 2; group 4, age ≥ 75 years meeting neither the criteria of group 1 nor group 2) are eligible for enrollment. In addition, eligible patients have a confirmed SARS-CoV-2 infection and O_2 saturation ≤ 94% while breathing ambient air. Patients are randomized to receive (experimental arm) or not receive (standard arm) convalescent plasma in two bags (238 - 337 ml plasma each) from different donors (day 1, day 2). A cross over from the standard arm into the experimental arm is possible after day 10 in case of not improving or worsening clinical condition. The primary endpoint is the time to clinical improvement defined as two points improvement (within 84 days) on a seven point ordinal scale or live discharge from the hospital. Secondary outcomes are mortality, changes in clinical scores, rate of mechanical ventilation, inflammatory cytokine changes and viral load dynamics. This trial systematically assesses the therapeutic role of convalescent plasma for treating SARS-CoV-2 infection in a high-risk patient population.

Zusammenfassung

Das Plasma rekonvaleszenter Patienten soll die Mortalität bei Patienten mit SARS, Ebola und Influenza verringern können. Dabei beruht die Evidenz auf kleineren Fallserien, retrospektiven Analysen und kleinen Klinischen Studien. In China wird Plasma bei schwerkranken Patienten mit COVID-19 eingesetzt ohne dass klare klinische Evidenz vorliegt. Plasma wurde üblicherweise bei kritisch Kranken mit fortgeschrittenem ARDS eingesetzt, was den therapeutischen Benefit fraglich erscheinen läßt.

Diese klinische Studie wird die Effekte von rekonvaleszentem Plasma von Menschen mit überstandener SARS-CoV-2 Infektion bei Hoch-Risiko COVID-19 Erkrankung testen. Plasma wird eingesetzt, das nachgewiesene Antikörper gegen SARS-Cov-2 enthält. Patienten mit SARS-Cov-2 Infektion und hohem Risiko für einen schweren Verlauf können in die Studie eingeschlossen werden. Gruppe 1: hämatologische oder onkologische Erkrankung mit/ohne Stammzelltransplantation und/oder laufende Krebstherapie (inkl. Chemotherapie, Strahlentherapie, Operation) innerhalb der letzten 24 Monate; Gruppe 2: Medikamentöse Immunsuppression und nicht in Gruppe 1; Gruppe 3: Alter 50-75 Jahre und nicht in Guppe 1 oder 2 mit mindestens Lymphopenie < 0.8 x G/l oder D-dimere > 1µg/mL; Gruppe 4: Alter ≥ 75 Jahre und nicht in Gruppe 1 oder 2. Alle einzuschliessenden Patienten haben RT-PCR bestätigte SARS-Cov-2 Infektion und O_2 Sättigung ≤ 94% bei Raumluft. Patienten werden randomisiert für rekonvaleszentes Plasma (Experimenteller Arm) mit zwei Beuteln Plasma (238 - 337 ml jeweils) von verschiedenen Spendern (Tag 1 und 2). Im Standardarm wird keine Plasmatherapie gegeben aber jeweils die vom Zentrum festgelegte antivirale und supportive Therapie. Ein Cross-Over vom Standardarm in den experimentellen Arm ist ab Tag 10 möglich im Falle keiner Änderung oder Verschlechterung des klinischen Verlaufs. Primärer Endpunkt ist die Zeit bis zur klinischen Verbesserung entsprechend einer Zwei-Punkte-Verbesserung (innerhalb 84 Tagen) auf einer Sieben-Punkte-Ordinalskala oder Entlassung lebend aus dem Krankenhaus. Sekundäre Endpunkte sind Sterblichkeit, Änderungen in klinischen Scores, Rate mechanischer Beatmung, Zytokinänderungen und

Viruslast über die Zeit. Diese Studie untersucht die therapeutische Bedeutung von rekonvaleszentem Plasma für die Behandlung von COVID-19 bei hohem Risiko für einen schlechten Verlauf.

Table of Contents

RESPONSIBILITIES 2
SUMMARY 5
ZUSAMMENFASSUNG 5
TABLE OF CONTENTS 7
TRIAL SCHEDULE 14
ABBREVIATIONS 18
1 INTRODUCTION 21
1.1 Scientific and Therapeutic Background 21
1.2 Trial Rationale/ Benefit- Risk Assessment 22
1.2.1 Trial Rationale *22*
1.2.2 Benefit - Risk Assessment *22*
1.3 Reference Committees 22
1.3.1 Data Monitoring Committee (DMC) *22*
2 TRIAL OBJECTIVES 22
2.1 Primary Objective 22
2.2 Secondary Objectives 22
3 TRIAL DESIGN 23
4 TRIAL DURATION AND SCHEDULE 23
5 SELECTION OF PATIENTS 24
5.1 Number of Patients and Recruitment 24
5.2 General Criteria for Patients' Selection 24
5.3 Inclusion Criteria 24
5.4 Exclusion Criteria 25
5.5 Criteria for Withdrawal 25
5.5.1 Withdrawal of Patients *25*
5.5.2 Handling of Withdrawals *26*
5.5.3 Premature Closure of the Clinical Trial or a Single Center *26*
5.6 Prior and Concomitant Illnesses 26
5.7 Concomitant Therapy 26
6 INVESTIGATIONAL MEDICINAL PRODUCT (IMP) 27
6.1 General Information about Convalescent Plasma 27
6.2 Therapeutic / Diagnostic Effects 27
6.3 Known Side Effects of Convalescent Plasma Infusions 28
6.4 Dosage Schedule 28
6.5 Treatment discontinuation 28
6.6 Enrolment, Randomisation, Treatment Assignment 28
6.7 Packaging and Labeling 29
6.8 Cross Over 29
6.9 Supplies and Accountability 29
6.10 Compliance 30
7 TRIAL METHODS 30
7.1 Description of Study Visits 30
7.2 Methods of Data and Sample Collection 31
7.2.1 Data Collection and Handling *31*
7.3 Measurement of Efficacy Parameters 32
7.3.1 Seven point ordinal scale *32*
7.3.2 Oxygen saturation (SaO_2) while breathing ambient air *32*
7.3.3 Laboratory parameters *32*
7.4 Measurement of Further Parameters 33
8 ANCILLARY AND POST TRIAL CARE 33
9 ASSESSMENT OF SAFETY 33

9.1 SPECIFICATION OF SAFETY PARAMETERS 33
9.1.1 Adverse Events 33
9.1.2 Serious Adverse Event 34
9.1.3 Expectedness 34
9.1.4 Suspected Unexpected Serious Adverse Reaction (SUSAR) 34
9.2 PERIOD OF SURVEILLANCE AND DOCUMENTATION 34
9.2.1 Grading of AEs 35
9.2.2 Coherence between AEs and the IMP 35
9.2.3 Outcome of AEs 35
9.2.4 Action taken 36
9.2.5 Countermeasures 36
9.3 REPORTING OF SERIOUS ADVERSE EVENTS BY INVESTIGATOR 36
9.4 EXPEDITED REPORTING 36
9.7 EMERGENCY TREATMENT 37
9.7 EVENTS OF SPECIAL INTEREST 37
10 STATISTICAL CONSIDERATIONS 37
10.1 STUDY DESIGN 37
10.2 ANALYSIS VARIABLES 37
10.2.1 Primary endpoint and primary estimand 37
10.3.2 Secondary Endpoints 38
10.2.3 Safety Endpoints 38
10.3 SAMPLE SIZE CALCULATION 38
10.4 ANALYSIS POPULATIONS 40
10.4.1 Full Analysis Population 40
10.4.2 Per Protocol Population 40
10.4.3 Safety Population 40
10.5 STATISTICAL METHODS 41
10.5.1 General Considerations 41
10.5.2 Demographic and other Baseline Characteristics 41
10.5.3 Study Therapy, Treatment Compliance, and Follow-up 41
10.5.4 Analysis of the Primary Endpoint 41
10.5.6 Handling of Missing Data 43
11 DATA MANAGEMENT 43
11.1 DATA COLLECTION 43
11.2 DATA HANDLING 44
11.3 STORAGE AND ARCHIVING OF DATA 44
12 ETHICAL AND LEGAL ASPECTS 44
12.1 GOOD CLINICAL PRACTICE 44
12.2 PATIENT INFORMATION AND INFORMED CONSENT 45
12.3 CONFIDENTIALITY 45
12.4 RESPONSIBILITIES OF INVESTIGATOR 45
12.5 APPROVAL OF TRIAL PROTOCOL AND AMENDMENTS 45
12.6 CONTINUOUS INFORMATION TO INDEPENDENT ETHICS COMMITTEE 46
12.7 NOTIFICATION OF REGULATORY AUTHORITIES 46
12.8 REGISTRATION OF THE TRIAL 46
12.9 INSURANCE 46
13 QUALITY ASSURANCE 47
13.1 MONITORING 47
13.2 INSPECTIONS/ AUDITS 47
14 AGREEMENTS 47
14.1 FINANCING OF THE TRIAL 47
14.2 DECLARATION OF INTERESTS 47
14.3 DISSEMINATION POLICY 47
14.3.1 Access to data 47
14.3.2 Reports 48

15 SIGNATURES 49
16 DECLARATION OF INVESTIGATOR 50
17 REFERENCES 51
18 APPENDICES 53
18.1 ORDINAL SCALE FOR CLINICAL IMPROVEMENT 53
18.2 SEQUENTIAL ORGAN FAILURE ASSESSMENT (SOFA) SCORE 54

List of Tables

Table 1: Trial schedule detailing protocol activities to be completed 14
Table 2: Clinical Laboratory Measurements 31
Table 3: Power for the RECOVER trial considering varying treatment effects across patient groups, assuming medC1= medC2 = medC3 = medC4=16 days 40

Protocol Synopsis

Title

A Randomized Open label Phase-II Clinical Trial with or without Infusion of Plasma from Subjects after Convalescence of SARS-CoV-2 Infection in High-risk Patients with Confirmed SARS-CoV-2 Disease and Confirmed Severe SARS-CoV-2 Disease

Phase

Phase II

Sponsor

Heidelberg University Hospital
represented in law by its acting Commercial Director Ms. Katrin Erk
Im Neuenheimer Feld 672, 69120 Heidelberg, Germany

Coordinating Investigator (LKP)

Prof. Dr. Carsten Müller-Tidow

Trial Site (s)

Multi-centre trial conducted in several German university medical centres (10 - 15)

Financing/Status of the Sponsor

The trial is co-financed by the BMBF program on emergency research funding for COVID-19 and the funds of Heidelberg University Hospital

Indication

SARS-CoV-2 infection requiring hospital admission

Inclusion Criteria

1. PCR confirmed SARS-CoV-2 infection in a respiratory tract sample.
2. Oxygen saturation (SaO_2) of 94% or less while breathing ambient air or a ratio of the partial pressure of oxygen (PaO_2) to the fraction of inspired oxygen (FiO_2) of less than 300 mm Hg.
3. High risk due to either

    pre-existing or concurrent hematological malignancy and/or active cancer therapy (incl. chemotherapy, radiotherapy, surgery) within the last 24 months or less. (group 1)

and/or

    chronic immunosuppression not meeting the criteria of group 1 (group 2)

and/or

    Age ≥ 50 - 75 years meeting neither the criteria of group 1 nor group 2 (group 3) and at least one of these criteria: Lymphopenia < 0.8 x G/l
    and/or

D-dimer > 1µg/mL

and/or

Age ≥ 75 years meeting neither the criteria of group 1 nor group 2 (group 4)

4. Blood hemoglobin concentration ≥ 10g/dl.
5. Provision of written informed consent.
6. Patient is able to understand and comply with the protocol for the duration of the study, including treatment and scheduled visits and examinations.
7. Male or female patient aged ≥ 18 years.
8. Postmenopausal or evidence of non-childbearing status. For women of childbearing potential: negative urine or serum pregnancy test within 14 days prior to study treatment.

Exclusion Criteria

1. Dementia, psychiatric or cognitive illness or recreational drug/alcohol use that in the opinion of the principle investigator, would affect subject safety and/or compliance.
2. Contraindication to transfusion or history of prior reactions to transfusion blood products.
3. Patients with known selective IgA deficiency.
4. Patients with mechanical ventilation and/or extracoporal membrane oxygenation (ECMO) at time of initial inclusion into the trial.
5. Participation in another trial with an investigational medicinal product.
6. Treatment with SARS-CoV-2 convalescent plasma in the past.

Objectives

Primary Objective:

- To assess the time from randomization until an improvement (within 84 days) defined as two points on a seven point ordinal scale or live discharge from the hospital in high-risk patients (group 1 to group 4) with a SARS-CoV-2 infection requiring hospital admission by infusion of plasma from subjects after convalescence of a SARS-CoV-2 infection or standard of care.

Secondary Objectives

1. To assess overall survival, and the overall survival rate at 28, 56 and 84 days.
2. To assess SARS-CoV-2 viral clearance and load, cytokine changes over time as well as antiviral antibody titres.
3. To assess percentage of patients that required mechanical ventilation
4. To assess time from randomization until discharge

Trial Design

Randomized, open-label, multicenter phase II trial, designed to assess the clinical outcome of SARS-CoV-2 disease in high-risk patients (group 1 to group 4) following treatment with anti-SARS-CoV-2 convalescent plasma or standard of care.

Investigational Medicinal Products

- Anti-SARS-CoV-2 convalescent plasma with SARS-CoV-2 antibodies obtained from subjects following recovery of a SARS-CoV-2 infection

Sample Size

Total sample size: n=174 patients (considering drop-outs)

Statistical Analysis

The trial is conducted as randomized, open-label, multicenter phase II study comparing clinical outcome in SARS-CoV-2-infected high-risk patients (group 1 to group 4) following treatment with anti-SARS-CoV-2 convalescent plasma or standard of care

Patients are stratified into different groups that will be used for randomization and for statistical analysis. It is expected that 25% of trial participants belong to group 1 including patients with hematological malignancies, history of stem cell transplantation or active cancer therapy (incl. chemotherapy, radiotherapy, surgery) within the last 24 months or less; 25% of trial participants (group 2) consist of patients with drug induced immunosuppression not meeting the criteria of group 1, 25% of trial participant (group 3) consist of patients with either age ≥ 50 -75 years and Lymphopenia < 0.8 x G/l and/or D-dimer > 1µg/mL meeting neither the criteria of group 1 nor group 2 and finally 25% of trial participant (group 4) are beyond > 75 years meeting neither the criteria of group 1 nor group 2.

Based on recently published data (Cao B, et al. NEJM 2020) median time to an improvement defined as two points on a seven point ordinal scale or live discharge from the hospital is assumed to be at day 16 from randomization.

We assume that based on the mechanism of action (neutralizing antibodies) a rapid improvement is induced by fresh frozen plasma from subjects after convalescence of SARS-CoV-2 infection on two days from two different donors with a median time of 10 days (HR=1.6).

While it is allowed for patients from the control arm to switch to the experimental arm starting from day 10, we assume this will not have a major effect on the median time of clinical improvement for these patients, since we expect that plasma infusions will have a larger advantage if given early. While methods for handling patients switching from control to treatment group are available, they are subject to strong and unverifiable assumptions (Watkins et al. 2013). Hence, we will ignore treatment switch in the primary analysis, representing a treatment policy strategy, which can be regarded as a conservative approach since switching is only allowed from the control to the experimental arm. However, in supplementary analyses of the primary endpoint, we will consider such a hypothetical scenario in which a treatment effect is estimated for the case that patients would not have switched by means of inverse probability censoring weighting according to Robins & Finkelstein (2000), relying on the assumption that there are no unmeasured confounders influencing the probability to switch, and a rank preserving structural failure time (RPSFT) model (Robins & Tsiatis 1991), assuming a common treatment effect which remains constant over time, regardless when treatment is initiated.

To detect the assumed difference between treatment groups using a log-rank test investigating the Kaplan-Meier curves for the event "clinical improvement by 2 points or live discharge from hospital", taking "death from any cause" into account by right-censoring deceased patients at day 84, using a significance level of 5% (two-sided) with a power of 80%, a total number of 174 patients is required for the entire trial (87 patients per group) when additionally considering a dropout rate of 5%. It is expected that adjusting for the confounder "patient group" in the analysis will lead to an increase in power. Sample size calculation was done assuming an equally long follow-up period of 38 days for each patient in order to properly estimate the probability of experiencing an event of interest, taking into

account that deceased patients will not experience the event of interest (see Section 10.3 for justification).

The primary analysis of the trial will compare the Kaplan-Meier curves for the primary endpoint at two-sided significance level of α=5%. This will be achieved by using a log-rank test stratified for factor “patient group”. The event “death from any cause” will be taken into account by right-censoring deceased patients at the last day of follow-up (day 84).

The fact that some patients from the control group might switch to the experimental group from day 10 will be ignored in the primary analysis. Patients who drop out during the trial or are lost to follow-up are taken into account as censored observations. Analysis will be based on the Full Analysis Population according to the Intention-to-treat principle.

A supplementary analysis of the primary endpoint will involve the inverse probability censoring weighting (IPCW) approach to estimate a treatment effect in the hypothetical scenario that patients from the control group had not switched to the experimental group (Robins & Finkelstein 2000). As another supplementary analysis modeling this hypothetical scenario, a rank preserving structural failure time (RPSFT) model will be fitted (Robins & Tsiatis 1991). Other supplementary analyses include a model by Fine and Gray (1999) targeting the subdistribution hazard for ‘clinical improvement by 2 points or live discharge from hospital’.

Further sensitivity analyses of the primary endpoint include an analysis based on the per protocol population.

Secondary outcomes are:

1. Overall survival and survival rates at days 28, 56 and 84
2. Differences in SARS-CoV-2 viral clearance and load as well as antibody titres
3. Percentage of patients that required mechanical ventilation
4. Time until discharge.

Safety: Safety analyses are performed according to established CTCAE guidelines

Trial Duration and Dates

Total trial duration:	18 months
Duration of the clinical phase:	12 months
First patient first visit (FPFV):	3rd Quarter 2020
Last patient first visit (LPFV):	2nd Quarter 2021
Last patient last visit (LPLV):	3rd Quarter 2021
Trial Report completed:	4th Quarter 2021

Table 1: Trial schedule detailing protocol activities to be completed

Trial Schedule

**Trial Period**	**Screening/ Baseline**	**Treatment**		**Daily Follow-up** (until hospital discharge)										**Weekly Follow-up**	
**Study day**	**≤ 7 days**	**Day 1**	**Day 2**	**Day 3**	**Day 4**	**Day 5**	**Day 6**	**Day 7**	**Day 8**	**Day 9**	**Day 10**	**Day 11 - 28**	**Day 14[swabs], 17, 21, 24, 28[swabs]**	**Day 35, 42, 49, 56**	**Day 70,84**
**Visit window (days)**		0	0	0	0	0	0	0	0	0	0	0	± 2	± 2	± 2
**Procedures / Assessments**															
Informed consent[2]	X														
Demographics[3]	X														
Medical history[4]	X														
Smoking history[5]	X														
Signs/symptoms[6]	X										X*				
Vital signs[7]	X	X	X	X	X	X	X	X	X	X	X	X			
Physical examination[8]	X										X*				
Clinical Frailty Scale (CFS)	X	X	X	X	X	X	X	X	X	X	X	X		(X)	(X)
Seven point ordinal scale[9#]	X	X	X	X	X	X	X	X	X	X	X	X		(X)	(X)
SOFA-Score[10#]	X	X	X	X	X	X	X	X	X	X	X	X		(X)	(X)
Oxygen saturation (SaO_2) while breathing ambient air[11#]	X	X	X	X	X	X	X	X	X	X	X	X		(X)	(X)
Fraction of Inspired Oxygen (FiO_2)[#]	X	X	X	X	X	X	X	X	X	X	X	X		(X)	(X)
PaO_2, $PaCO_2$[#]	X	X	X	X	X	X	X	X	X	X	X	X		(X)	(X)
Amount of oxygen supplementation[12#]	X	X	X	X	X	X	X	X	X	X	X	X		(X)	(X)
Cross-over Assessment[13]											X				
ECHO[14]	X														
ECG[15]	X										X*				

**Trial Period**	**Screening/ Baseline**	**Treatment**		**Daily Follow-up** (until hospital discharge)										**Weekly Follow-up**	
**Study day**	**≤ 7 days**	**Day 1**	**Day 2**	**Day 3**	**Day 4**	**Day 5**	**Day 6**	**Day 7**	**Day 8**	**Day 9**	**Day 10**	**Day 11 - 28**	**Day 14[swabs], 17, 21, 24, 28[swabs]**	**Day 35, 42, 49, 56**	**Day 70,84**
**Visit window (days)**		0	0	0	0	0	0	0	0	0	0	0	± 2	± 2	± 2
**Laboratory Tests at *Local Lab***															
Blood collection for Hematology *(local lab)*[16#]	X	X	X	X	X	X	X	X	X	X	X		X	(X)	(X)
Blood collection for chemistry and coagulation *(local lab)*[17#]	X	X	X	X	X	X	X	X	X	X	X		X	(X)	(X)
Urinalysis *(local lab)*[18]	X													(X)	(X)
Blood collection for HAV, HBV, HCV, HIV-1 screen *(local lab)*[19]	X														
Pregnancy test *(local lab)*[20]	X														
**Viral Diagnostic and Biobanking *(Central Lab, Heidelberg)***															
Nose/throat swabs for SARS-Cov-2_PCR *(central lab)* [21]		X[µ]	X	X		X		X			X		X ‹d14 & d28›		
Blood collection for SARS-Cov-2_Serology *(central lab)*[21]		X[µ]	X	X		X		X			X*		X ‹d14 & d28›	X[ɸ]	X[ɸ]
Blood samples for biobanking *(central lab)*[22]		X[µ]	X	X		X		X			X		X ‹d14 & d28›	X[ɸ]	X[ɸ]
Inclusion and exclusion criteria	X	X									X*				
Enrollment[23]	X														
Randomisation[2]		X													
**Treament / Convalescent Plasma[24]**		X	X												
Assessment of Concomitant medications[25]	X	X	X	X	X	X	X	X	X	X	X	X	X	X	X
AE assessments[26]		X	X	X	X	X	X	X	X	X	X	X	X	X	X

Footnotes for schedule of events:

*In case of cross-over — #measured until hospital discharge — ϕOnce between day 35 and day 84

( ) Follow-up by regular phone calls — μBlood collection before CP administration

1. **Observation time** for all patients is expected to be at least 3 months after entry into the study. Patients receive convalescent plasma for two days (day 1 and day 2) or standard of care. For patients in the standard arm, cross over is allowed from day 10 in case of not improving or worsening clinical condition. There is a regular *follow-up* of 3 months. All discharged patients are followed by regular phone calls (scheduled days: 14, 17, 21, 24, 28, 32, 42, 49, 56, 70, 84). In person visits after discharge are only advised if clinically indicated. Timing of all visits must be based on the date of first CP administration. The study period ends on the day of the last follow-up visit of the last randomized patient. All Patients with ongoing plasma-related toxicities must be followed until all plasma-related toxicities are resolved.
2. **Informed consent and patient registration**: every patient must sign the informed consent to participate in this trial before starting any trial-related procedures. After signature, a patient ID (three-digit) is generated via registration in the eCRF system. After completion of the screening/baseline visit and assessment of the inclusion and exclusion criteria, **randomisation** is carried out by the responsible investigator **online via https:/randomizer.at** (Uni Graz) with assignment of the random number.
3. **Demographics**: gender, year of birth, ethnicity
4. **Medical history**: date and type of first symptoms and detailed information on pretreatment including type and composition of prior therapy, family history (cancer, cardiovascular disease, diabetes, rheumatological disease), additional medical history on concomitant diseases in particular cancer and autoimmune diseases and their treatment.
5. **Information on smoking;** duration and amout, pack-years
6. **Signs/symptoms:** infection related and unrelated signs and symptoms, in particular cough, myalgia, fatigue, diarrhoea, vomiting,
7. **Vital signs**: WHO performance status, temperature (in grade centigrade), blood pressure/pulse, respiratory rate, at baseline: height (in cm), weight (in kg). After hospital discharge the patients are contacted by phone or e-mail and asked about their performance status until 84 days after randomization.
8. **Physical examination:** inspection, lung, cardiac and abdominal auscultation and percussion, palpation of the abdomen and lymph node sites, neurological examination
9. **Seven point ordinal scale:** Assessment of the seven point ordinal scale (1, not hospitalized with resumption of normal activities; 2, not hospitalized, but unable to resume normal activities; 3, hospitalized, not requiring supplemental oxygen; 4, hospitalized, requiring supplemental oxygen; 5, hospitalized, requiring nasal high-flow oxygen therapy, noninvasive mechanical ventilation, or both; 6, hospitalized, requiring ECMO, invasive mechanical ventilation, or both; and 7, death.
10. **SOFA-Score:** The sequential organ failure assessment score (SOFA score) is assessed to determine the extent of a person's organ function or rate of failure. It integrates scoring of the respiratory system, nervous system, cardiovascular system, liver, coagulation and kidneys (Appendix).
11. **Oxygen saturation (SaO_2)** while breathing ambient air measured by pulse oximetry. See 7.3.2 for details.
12. Amount of oxygen supplementation: i) supplemental oxygen l/min; nasal high-flow oxygen therapy l/min noninvasive mechanical ventilation, ECMO, invasive ventilation
13. **Cross-over Assessment:** For cross-over, all inclusion / exclusion criteria (except mechanical ventilation/ECMO) must again be met as specified in Chapters 5.3 and 5.4. After Cross over, the visit schedule is restarted at day 1 in the experimental arm. The timing of subsequent visits is based on the day of first CP administration.
14. **ECHO**: at baseline and thereafter at investigator's discretion

15. **ECG:** 12-lead ECG, at baseline, and thereafter at investigator's discretion
16. **Hematology (local lab):** hemoglobin, RBC, PLT, WBC, Differential cell counts at baseline.
17. **Blood chemistry (local lab):** BUN, creatinine, albumin, AST/SGOT, ALT/SGPT, total bilirubin, gGT, AP, LDH, sodium, potassium, magnesium, calcium, serum uric acid, LDH, Troponin, CK and CK-MB, Quick, PTT, ATIII, D-Dimer, Fibrinogen, Ferritin, transferrin levels and transferrin saturation, CRP, total protein, albumin, IL6, procalcitonin, total IgG, IgA, IgM.
18. **Urinalysis (local lab):** pH, glucose, proteins (qualitative, dipstick accepted): at baseline and at investigator's discretion during treatment.
19. **HAV, HBV, HCV, and HIV-1 testing**: at baseline.
20. **Serum/urine pregnancy test** (local lab). Only for pre-menopausal women of childbearing potential. Must be done within 14 days prior to the start of study treatment and or prior to randomisation.
21. **SARS-Cov-2_PCR nose/throat swabs and SARS-Cov-2_Serology:** Swabs are collected at day 1, 2, 3, 5, 7, 10, 14, 28 or until discharge, and serum once during follow-up within 84 days. Convalescent plasma is administered on day 1 and 2. **On day 1 blood sampling for serology and nose/throat swabs must be done before CP administration.** Further details will be provided in the lab manual.
22. **Procurement of Samples for Biobanking** at IM-V Heidelberg University Hospital: 7.5 ml serum and 2x 7.5 ml Li-Heparin (only at day 1 and day 14). Further details will be provided in the lab manual.
23. **Enrollment:** patients are enrolled and registered through the NCT trials office patient registration system. The investigator is requested to sign the Request for Enrollment form. This signature guarantees that eligibility criteria are met.
24. **Convalescent plasma is administered on days 1-2. On day 1, blood collection for SARS-Cov-2 serology and nasopharyngeal swabs must be done PRIOR CP administration.**
25. **Concomitant medications** is reported in the relevant CRF pages, including supportive care drugs, prophylaxis, and drugs used for treating AEs or chronic diseases.
26. **AE assessments:** events are documented and recorded continuously. Patients must be followed for AEs from first plasma administration up to 28 days after last plasma administration or until all drug-related toxicities have been resolved, whichever is later, or until the investigator assesses AEs as "chronic" or "stable". Each AE must be reported, indicating the worst CTC (Version 5.0) grade. If an event stops and later restarts, all occurrences must be reported. A specific procedure for definition and reporting of SAEs is described in the protocol.

Abbreviations

ADE	Antibody Dependent Enhancement
AE	Adverse Event
ALT	Alanine Amino Transferase, also known as SGPT
AMG	Deutsches Arzneimittelgesetz (German Drug Law)
AP	Alkaline Phosphatase
ARDS	Acute Respiratory Distress Syndrome
AST	Aspartate Amino Transferase, also known as SGOT
ATIII	Antithrombin III
BUN	Blood Urea Nitrogen
CK	Creatine Kinase
CMV	Cytomegalovirus
COVID-19	Coronavirus Disease 2019
CP	Convalescent plasma
CRF	Case Report Form
CRP	C-Reactive Protein
CTC	Common Toxicity Criteria
CTCAE	Common Toxicity Criteria for Adverse Events
DMC	Data Monitoring Committee
DSUR	Development Safety Update Report
EC	Ethics Committee
ECHO	Echocardiogram
ECMO	Extracoporal Membrane Oxygenation
ELISA	Enzyme-linked Immunosorbent Assay
EMA	European Medicines Agency
EOS	End of Study
EOT	End of Treatment
FAP	Full Analysis Population
FiO2	Fraction of Inspired Oxygen
FPFV	First Patient First Visit
FSH	Follicle Stimulating hormone
GCP	Good Clinical Practice
GDPR	General Data Protection Regulation

gGT	Gamma-glutamyl transferase
H1N1	Hemagglutinin Type 1 and Neuraminidase Type 1 (Swine Flu)
H5N1	Hemagglutinin Type 5 and Neuraminidase Type 1 (Avian Influenza)
HAV	Hepatitis A Virus
HBV	Hepatitis B Virus
HCV	Hepatitis C Virus
HIV	Human Immune-deficiency Virus.
HR	Hazard Ratio
IC	Informed Consent
ICH	International Council on Harmonization of Technical Requirements for Registration of Pharmaceuticals for Human Use
ICTRP	International Clinical Trials Registry Platform
ICTRP	International Clinical Trials Registry Platform
Ig	Immunoglobulin
IKTZ	Institut für Klinische Transfusionsmedizin und Zelltherapie (Institute for Clinical Transfusion Medicine and Cellular Therapy)
IL-6	Interleukin-6
IMP	Investigational Medicinal Product
IMPD	Investigational Medicinal Product Dossier
IPCW	Inverse Probability Censoring Weighting
ISF	Investigator Site File
ITT	Intention-To-Treat
LDH	Lactate Dehydrogenase
LH	Luteinizing hormone
LKP	Leiter der Klinischen Prüfung (Coordinating Investigator according to AMG)
LPFV	Last Patient First Visit
LPLV	Last Patient Last Visit
MERS	Middle East Respiratory Syndrome
NCT	National Center for Tumor Diseases
PaO_2	Partial Pressure of Oxygen
PCR	Polymerase Chain Reaction
pH	Potential Hydrogen
PLT	Platelet
PRO	Patient Reported Outcome
PTT	Partial Thromboplastin Time
RBC	Red Blood Cell

RPSFT	Rank Preserving Structural Failure Time
SAE	Serious Adverse Event
SaO_2	Oxygen saturation
SAP	Statistical Analysis Plan
SARS-CoV	Severe Acute Respiratory Syndrome Coronavirus 2
SDV	Source Data Verification
SGOT	Serum Glutamic-Oxaloacetic Transaminase, also known as AST
SGPT	Serum Glutamic-Pyruvat Transaminase, also known as ALT
SOFA	Sequential Organ Failure Assessment
SOP	Standard Operating Procedures
SUSAR	Suspected Serious Unexpected Adverse Reaction
TACO	Transfusion Associated Circulatory Overload
TMF	Trial Master File
TRALI	Transfusion Associated Lung Injury
WBC	White Blood Cell
WHO	World Health Organization

1 Introduction

1.1 Scientific and Therapeutic Background

The SARS-CoV-2 disease, also known as coronavirus disease 2019 (COVID-19) is a respiratory illness caused by the newly discovered Severe Acute Respiratory Syndrome Coronavirus 2 (SARS-CoV-2). First cases of COVID-19 were reported in December 2019 [1]. The viral infection subsequently rapidly spread worldwide resulting in a pandemic with severe implications on health care systems due to high morbidity and mortality. The infection is characterized by a mild to moderate respiratory illness in most affected patients. However, older people, and patients with concomitant diseases such as chronic respiratory diseases, cardiovascular diseases, cancers, and/or chronic immunosuppression are at high risk to develop severe respiratory distress [2-4].

Current control strategies of SARS-CoV-2 disease are limited to prevention, case monitoring and supportive care. A validated vaccine or curative treatment is not available yet, resulting in disease widespread and increasing mortality [5]. Therefore, novel therapeutic options are urgently needed. Outbreaks have overwhelmed the health care systems in several regions of the world, notably in China (Heibei province), Italy (Lombardy) and in France (Alsace). Patients requiring mechanical ventilation were not intubated due to shortages in ventilators, material and staff [6]. Accordingly, early effective treatment is urgently required to avoid the need for mechanical ventilation.

Over the last past decades, convalescent plasma (CP) has been used for the prevention or treatment of various life-threatening infections, especially respiratory viral infections such as H1N1, H5N1 and the coronaviruses SARS1 and MERS [7-10]. Recently, encouraging results of the treatment of 5 critically ill COVID-19 patients with convalescent plasma have been reported [11]. There are additional non-peer reviewed reports about benefit in SARS-CoV-2 infected patients [12]. SARS-Cov-2 infection is associated with cytokine changes that may partially drive the pathogenesis of Acute Respiratory Distress Syndrome, ARDS [13]. Plasma therapy has a long standing history with an excellent safety profile [14]. From a theoretical perspective, there are *in vitro* concerns whether antibodies against SARS-CoV-2 might lead to Antibody Dependent Enhancement (ADE) of virus entry into cells [15].

Thus, there is now accumulating evidence suggesting that passive immunotherapy using CP decreases the morbidity and mortality in SARS and epidemic Influenza [9,11,16,17]. However, these data were gained through retrospective studies, case reports, and CP treatment was usually combined with other drugs [18]. Hence, reliable evidence of the association of CP treatment with favourable clinical course – particularly in SARS-Cov-2 infections – has not been properly demonstrated yet.

1.1.2 Passive antibody therapy

The principle of passive antibody therapy using infusions of CP is based on the fact that following infection with an infectious agent, the infected person produces specific antibodies against the latter. Thus, CP obtained from subjects, who have recovered from an infection provides a specific prophylactic or therapeutic weapon against the corresponding infectious agent. Unlike vaccination, passive antibody therapy provides an immediate protection [19,20]. This is of particular importance in an infectious outbreak context, especially for high-risk, patients who might not develop a prompt and adequate immune response due to underlying diseases or concomitant therapies.

1.2 Trial Rationale/ Benefit- Risk Assessment

1.2.1 Trial Rationale

The aim of this randomized phase-II study is to gain evidence on the effect of CP in the treatment of SARS-CoV-2 infection in high-risk patients. High-risk is defined within 4 groups; group 1, pre-existing or concurrent hematological malignancy and/or active cancer therapy (incl. chemotherapy, radiotherapy, surgery) within the last 24 months or less; group 2, chronic immunosuppression not meeting the criteria of group 1; group 3, age ≥ 50-75 years and meeting neither the criteria of group 1 nor group 2 and lymphopenia < 0.8 x G/l and/or D-dimer > 1µg/mL; group 4, age ≥ 75 years and meeting neither the criteria of group 1 nor group 2). The main focus of the protocol is to assess the clinical outcome in high-risk patients after infusion of CP in patients with confirmed SARS-CoV-2 infection and a O_2 saturation ≤ 94% while breathing ambient air. Patients requiring mechanical ventilation and/or extracoporal membrane oxygenation (ECMO) are not eligible for the trial.

Based on previous studies, we hypothesize that an infusion of CP relatively early during the SARS-CoV-2 infection might reduce the severity of the disease rapidly and thus avoid a progression to a more severe disease state and death.

1.2.2 Benefit - Risk Assessment

Currently, clinical management of high-risk COVID-19 patients remains a challenge [5]. In general, treatment of SARS-CoV-2 infections is purely symptomatic and depends on the underlying diseases. After failure of supportive care, there are currently no approved options available for these patients; clinical studies with remdesivir, hydroxychloroquine and tocilizumab are ongoing while a randomized study lopinavir-ritonavir was negative [21].

Taking into consideration the beneficial safety profile, infusion of CP may offer access to a potentially effective therapy in high-risk patients whose treatment options are limited [20].

1.3 Reference Committees

1.3.1 Data Monitoring Committee (DMC)

The DMC is composed of at least three independent experts, assessing the progress and safety data. The mission of the DMC is to ensure the ethical conduct of the trial and to protect the safety interests of patients in this trial.

The DMC meetings are planned after treatment of 5, 10 and 25 patients in the experimental arm of the study and according to the DMC charter. Based on its review, the DMC provides the sponsor with recommendations.

Further details including DSUR reviews and DMC members is specified in the DMC charter.

2 Trial Objectives

2.1 Primary Objective

- To assess the time from randomization until an improvement within 84 days defined as two points on a seven point ordinal scale or live discharge from the hospital in high-risk patients (group 1 to group 4) with SARS-CoV-2 infection requiring hospital admission by infusion of plasma from subjects after convalescence of SARS-CoV-2 infection or standard of care

2.2 Secondary Objectives

- To assess overall survival, and the overall survival rate at 28, 56 and 84 days.
- To assess SARS-CoV-2 viral clearance and load as well as antibody titres.
- To assess percentage of patients that required mechanical ventilation.

- To assess time from randomization until discharge

For detailed endpoints see sections 10.3.2. and 10.3.3

3 Trial Design

This is a multicentre, randomized, open-label, phase II clinical trial designed to evaluate the clinical outcome in high-risk patients with SARS-CoV-2 infection by infusion of plasma from subjects after convalescence of SARS-CoV-2 infection.

High-risk is defined as SARS-CoV-2 positive infection with Oxygen saturation at ≤ 94% at ambient air with additional risk features as categorized in 4 groups:

- group 1, pre-existing or concurrent hematological malignancy and/or active cancer therapy (incl. chemotherapy, radiotherapy, surgery) within the last 24 months or less.
- group 2, chronic immunosuppression not meeting the criteria of group 1
- group 3, age ≥ 50 - 75 years meeting neither the criteria of group 1 nor group 2
  and at least one of these criteria:
  Lymphopenia < 0.8 x G/l
  and/or
  D-dimer > 1µg/mL
- group 4, age ≥ 75 years meeting neither the criteria of group 1 nor group 2

The primary end point is the time to clinical improvement within 84 days, defined as the time from randomization to either an improvement of two points on a seven-category symptom ordinal scale or live discharge from the hospital, whichever comes first. A total of 174 patients are planned to be included into the trial.

4 Trial Duration and Schedule

The duration of the trial for each patient is expected to be about 3 months, including two days of intervention (infusion of frozen CP), followed by a follow-up of 3 months. Furthermore viral load is measured daily in nasopharagyngeal swabs at day 1, 2, 3, 5, 7, 10, 14, 28 or until hospital discharge within 84 days after randomization.

Patients randomized into the standard arm of the study have the possibility to cross over into the experimental arm of the study starting from day 10 in case of not improving or worsening clinical condition.

Conditions leading to patient withdrawal from the study are detailed in Section 5.5 Criteria for Withdrawal.

Treatment response is assessed continuously until day 28, thereafter weekly until day 56, 70 and finally at day 84. Primary endpoint is the time to clinical improvement by two points on a seven point ordinal scale or live discharge from the hospital.

A recruitment period of approximately 9 months and an overall study duration of approximately 12 months is anticipated. Recruitment of patients starts in the third quarter of 2020.

The study duration of an individual patient is planned to be 3 months.

After finishing all study-relevant procedures, therapy, and follow-up period, the patient is followed in terms of routine care and treated if necessary.

Total trial duration:	18 months
Duration of the clinical phase:	12 months
First patient first visit (FPFV):	3rd Quarter 2020
Last patient first visit (LPFV):	2nd Quarter 2021
Last patient last visit (LPLV):	3rd Quarter 2021
Trial Report completed:	4th Quarter 2021

5 Selection of Patients

5.1 Number of Patients and Recruitment

As described in Section 10.2 (Size Calculation) a total of 174 patients are planned to be enrolled in the clinical trial.

5.2 General Criteria for Patients' Selection

This clinical trial can fulfill its objectives only if appropriate patients are enrolled. The following eligibility criteria are designed to select patients for whom protocol treatment is considered appropriate. All relevant medical and non-medical conditions should be taken into consideration when deciding whether this protocol is suitable for a particular patient. Any questions regarding a patient's eligibility should be discussed with the Coordinating-, Co-Coordinating-Investigator or the Scientific Coordinator.

5.3 Inclusion Criteria

Both female and male patients are included in this clinical trial. Patients must meet all of the following inclusion criteria to be eligible for enrollment into the study:

1. PCR confirmed SARS-CoV-2 infection in a respiratory tract sample.
2. Oxygen saturation (SaO_2) of 94% or less while breathing ambient air or a ratio of the partial pressure of oxygen (PaO_2) to the fraction of inspired oxygen (FiO_2) of less than 300 mm Hg.
3. High risk due to either

   pre-existing or concurrent hematological malignancy and/or active cancer therapy (incl. chemotherapy, radiotherapy, surgery) within the last 24 months or less (group 1)

   and/or

   chronic immunosuppression not meeting the criteria of group 1 (group 2)

   and/or

   Age ≥ 50 -75 years meeting neither the criteria of group 1 nor group 2 (group 3)
   and at least one of these criteria:
   Lymphopenia < 0.8 x G/l
   and/or
   D-dimer > 1µg/mL

   and/or

   Age ≥ 75 years meeting neither the criteria of group 1 nor group 2 (group 4).
4. Blood hemoglobin concentration ≥ 10g/dl.
5. Provision of written informed consent.
6. Patient is able to understand and comply with the protocol for the duration of the study, including treatment and scheduled visits and examinations.

7. Male or female patient aged ≥ 18 years
8. Postmenopausal or evidence of non-childbearing status. For women of childbearing potential: negative urine or serum pregnancy test within 14 days prior to study treatment.

   Postmenopausal or evidence of non-childbearing status is defined as:

   - Amenorrheic for 1 year or more following cessation of exogenous hormonal treatments
   - Luteinizing hormone (LH) and Follicle stimulating hormone (FSH) levels in the post menopausal range for women under 50 except in patients with a history of surgical sterilisation (bilateral oophorectomy or hysterectomy)
   - Radiation-induced oophorectomy with last menses > 1 year ago
   - Chemotherapy-induced menopause with > 1 year interval since last menses
   - Surgical sterilisation (bilateral oophorectomy or hysterectomy)
   - Female patients of child bearing potential and male patients with partners of child bearing potential, who are sexually active, must agree to the use of highly effective forms of contraception. This should be started from the signing of the informed consent and continue throughout the study and for 1 month (female patients) / 3 months (male patients) after last infusion of CP

5.4 Exclusion Criteria

Patients fulfilling any of the following criteria cannot be enrolled in the trial:

1. Dementia, psychiatric or cognitive illness or recreational drug/alcohol use that in the opinion of the principle investigator, would affect subject safety and/or compliance.
2. Contraindication to transfusion or history of prior reactions to transfusion blood products.
3. Patients with selective IgA deficiency.
4. Patients with mechanical ventilation and/or extracoporal membrane oxygenation (ECMO) at time of initial inclusion into the trial. Mechanical ventilation is defined as either NIV – non-invasive ventilation or positive pressure ventilation. Enrollment into another clinical trial evaluating specific therapies for COVID-19 is encouraged.
5. Participation in another trial with an investigational medicinal product.
6. Treatment with SARS-CoV-2 convalescent plasma in the past.
7. Blood hemoglobin concentration below lower limit of normal.

5.5 Criteria for Withdrawal

5.5.1 Withdrawal of Patients

A patient must be withdrawn from the trial treatment or/and all trial-related procedures for the following reasons:

1. At any time at their own request withdrawal of patient's consent to continue therapy. The patient is at any time free to discontinue treatment, without prejudice to further treatment.
2. Changes in medical status of the patient such that the investigator believes that patient safety is compromised or that it would be in the best interest of the patient to stop treatment
3. Pregnancy

A patient may be withdrawn from the trial treatment or/and all trial-related procedures for the following reasons:

1. Non-compliance by the patient with protocol requirements

2. Patient is lost to follow-up. If a patient does not return for scheduled visits, every effort should be made to re-establish contact. In any circumstance, every effort should be made to document patient outcome if possible

Conditions leading to treatment discontinuation are specified in Section 6.5.

If the patient withdraws from the trial and also withdraws consent for disclosure of future information (e.g. follow-up visits), no further evaluations are allowed to be performed and no additional data can be collected. The sponsor may retain and continue to use any data collected before such withdrawal of consent. Unresolved AEs in withdrawn patients have to be followed.

5.5.2 Handling of Withdrawals

In all cases, the reason for withdrawal must be recorded in the eCRF and in the patient's medical records. In case of withdrawal of a patient at his/ her own request, the reason should be asked for as extensively as possible and documented.

5.5.3 Premature Closure of the Clinical Trial or a Single Center

The trial can be prematurely closed or suspended by the Sponsor after consulting the Coordinating Investigator. The Ethics Committee (EC) and the Competent Regulatory Authorities must then be informed. Furthermore, the Ethics Committee(s) and Competent Regulatory Authorities themselves may decide to stop or suspend the trial.

Should the trial be closed prematurely, all trial material (completed, partially completed, and blank CRFs, investigational medicinal product and other material) must be returned to the Sponsor in Heidelberg or treated according Sponsor notice.

All involved investigators have to be informed immediately about a cessation/suspension of the trial. The decision is binding to all trial centers and investigators.

The Sponsor after consulting the Coordinating Investigator has the right to close a center, at any time, in case of:

- Non-compliance with the protocol
- Poor data quality
- No recruitment

5.6 Prior and Concomitant Illnesses

Relevant additional illnesses present at the time of informed consent are regarded as concomitant illnesses and are documented on the appropriate pages of the electronic case report form (eCRF). Included are conditions that are seasonal, cyclic, or intermittent (e.g. seasonal allergies; intermittent headache).

Abnormalities that appear for the first time or worsen (intensity, frequency) during the trial are adverse events (AEs) and must be documented on the appropriate pages of the eCRF.

5.7 Concomitant Therapy

While only Dexamethasone has shown mortality benefits in advanced COVID-19 in a non-peer reviewed study, there are currently no established therapies for COVID-19 early in the course of disease to prevent progression. Nonetheless, centers will follow guidelines and explore other therapies applied to COVID-19 patients. In this trial, each center upfront reports their standard therapy for the COVID-19 study population defined in the RECOVER trial. Standard arm patients receive the therapy outlined by the investigator of the participating center. CP arm patients receive CP therapy in addition to standard therapy. In case that the treatment algorithm of participating centers changes (likely during the course of the trial), these changes are reported. All treatments prescribed as antiviral or SARS-Cov-2 specific therapies are indicated with dosing and timing in the eCRF. Therapies which need to be noted in the eCRF include: all antiviral drugs, anti-IL6 therapies, Chloroquin,

Hydroxychloroquin, Azithromycin, steroids as well as other therapies aimed at altering immune response or viral replication. Patients may receive these drugs or combinations thereof during the entire trial.

There is no prohibited co-medication during the trial.

6 **Investigational Medicinal Product (IMP)**

The IMP consists of human plasma obtained by apheresis from patients/donors who have recovered from COVID-19 infection and have consented to donate. The IMP is composed of human convalescent plasma and a stabilizer solution of sodium citrate. IMP Name: RECOVER CT Convalescent Plasma COVID-19 (HD)

6.1 General Information about Convalescent Plasma

Covalescent plasma (CP) is generated by the IKTZ Heidelberg (Institut für Klinische Transfusionsmedizin und Zelltherapie) using the equipment and technologies already in operation at the IKTZ following standard operating procedures. A detailed description of the manufacturing process is provided in the IMPD. The drug product will be kept frozen during storage and thawed immediately prior to transfusion. During the course of the trial, further centers may produce CP if all preconditions and formal requirements are met.

Origin:	Plasma from apheresis obtained from donors, who have recovered from SARS-CoV-2 infection
Container:	Plastic Bag with CE certificate
Route of administration:	Intravenous
Storage conditions:	Temperature: ≤ - 30°C in<br>Maximum storage period: 2 years including the day of manufacture
Manufacturer / Importer:	Institut für Klinische Transfusionsmedizin und Zelltherapie Heidelberg gGmbH, Im Neuenheimer Feld 305, 69120 Heidelberg (IKTZ Heidelberg)

The IKTZ Heidelberg as a blood donation[1]center provides convalescent plasma bags directly to the respective and named addressee of the trial site, according to sponsor´s indication. The IKTZ Heidelberg ensures that donors are healthy at the time of donation and have not received prior treatment with hydroxychloroquine and/or 4-aminoquinolines within 6 months before donation. CP is packaged and labelled in accordance with the Good Manufacturing Practice guidelines and national and/or local regulatory requirements. CP has to be kept in a secure place in a blood bank / blood depot under appropriate storage conditions.

6.2 Therapeutic / Diagnostic Effects

Although the therapeutic effect of COVID-19 CP is not well known, previous reports suggest that neutralizing antibodies present in the sera may suppress viremia, when transferred to SARS-CoV-2-infected patients – leading to an improvement of symptoms, a shorter duration

[1] Only donors without exposure to hydroxychloroquine and / or 4-aminoquinolines within the last 6 months prior plasma donation are eligible.

of hospitalization and reduced mortality compare to patients not treated with CP [11,16,20]. Furthermore, infusion of CP at an early stage of other coronavirus infections (H1N1 and SARS) has been associated with high effectiveness [9,16,22]. Thus, we anticipate that early CP treatment of high-risk COVID-19 patients results in a favourable outcome in a vulnerable patient population.

6.3 Known Side Effects of Convalescent Plasma Infusions[2]

According to previous reports, no significant side effects or serious adverse events have been specifically associated with CP infusions so far [18,23,24]. The most common side effects of CP infusion are similar to side effects of application of human plasma [20,25]:

- Volume overload with high infusion rates and high infusion volumes (Transfusion associated circulatory overload TACO) especially in patients with cardiac risk factors with the risk of cardiac failure and lung edema.
- Citrate intoxication with high infusion rates and high infusion volumes especially in liver failure, shock, acidosis, hypothermia and in newborns.
- Allergic reactions, in rare cases anaphylactic shock.
- Rare: Risk of transfusion transmitted infections: bacteria, viruses (HIV, HCV, HBV, CMV and others).
- Rare: transfusion associated lung injury (TRALI)
- There are theoretical risks that antibodies might enhance viral entry into cells or that antibodies might trigger. Accordingly, CP effects will be closely evaluated in this trial.

6.4 Dosage Schedule

Preparation of CP infusions must be conducted in a manner consistent with recommended safety standards for handling blood and its derivates.

CP infusions is administered on two days: visit day 1 (≤ 7 days after baseline) and day 2. Each CP bag contains approx. 238 - 337 ml anti-SARS-Cov-2 CP for infusion. The CP can be administered after thawing of cryopreserved CP < -30°C. CP is used ABO compatible. The CPs are going to be administered with an infusion rate of 80-100 ml/ hour within a four hour period for each CP bag. CP is infused over a standard transfusion device with a pore size of 170-230 µm.

6.5 Treatment discontinuation

Definition of conditions leading to CP treatment discontinuation:

- **Adverse event with the transfusion of CP**: continuation of CP treatment after AE resolution to < grade 2 according to CTCAE
- **Severe allergic reactions or anaphylactic shock / serious adverse event upon CP transfusion:** transfusion must be stopped immediately and no further CP treatment is applied. A second CP administration is skipped.

6.6 Enrolment, Randomisation, Treatment Assignment

A subject is considered enrolled when he or she has signed the Informed Consent form. The patient receives a screening number at the clinical site (number of site plus number of patient in ascending order, e. g. 001-001 for the first enrolled patient at site 01) via registration in the eCRF system (www.xxx.). The screening number is used to identify the

[2] This section of the study protocol is defined as reference safety information (RSI).

subject throughout the clinical study and must be used on all study documentation related to the subject.

Clinical sites must complete the baseline case report forms for all enrolled subjects, even if the subject is not randomised or treated in this study.

Upon confirmation of eligibility (patients must meet all inclusion criteria and must not meet exclusion criteria), the clinical site must contact a centralized internet randomization system (https://randomizer.at/). Patients are randomized using block randomisation to one of the two arms, experimental arm or standard arm, in a 1:1 ratio considering a stratification accoding to the 4 risk groups (as described in the inclusion criteria).

All subjects randomised receive a unique randomisation number. All patients in the experimental group have to start CP treatment within 7 days of randomization. Patients withdrawn from the trial retain their Patient ID and randomization number.

The study is open-label.

6.7 Packaging and Labeling

Labels are prepared in accordance with Good Manufacturing Practice (GMP) and local regulatory guidelines. The labels fulfill German GCP Ordinance (GCP-V § 5).

The following aspects are part of labeling (minimum requirements):

1) Name, Address and phone number of the sponsor
2) Name of IMP
3) AB0 and Rhesus blood group
4) Unique coding of blood bag with clear batch number ("Ch.-B.")
5) Dosage form and route of Administration
6) Volume and Ingredients
7) Storage conditions
8) Directions for use
9) Manufacturing and expiry date
10) Protocol number; EudraCT number
11) Recipient: Name, Surname, Date of Birth, subject identification number / ID, visit number / day, Treating Department, Treating Physician

Caution: Must be used only for this clinical trial. Please follow the instructions in the information leaflet.

6.8 Cross Over

Cross-over into the experimental arm is intended for patients randomized into the standard arm if the following criteria is met:

- Starting from day 10 after randomization
- No improvement or worsening of clinical condition

For cross-over, all inclusion / exclusion criteria (except mechanical ventilation/ECMO) must again be met as specified in Sections 5.3 and 5.4.

The actual possibility for cross-over into the experimental arm is based on discussion with the coordinating investigator on a case-by-case decision.

6.9 Supplies and Accountability

The local investigator keeps an account of the trial medication and acknowledge the receipt of all shipments of the IMP (Investigational Medicinal Product). All trial IMP must be kept in a

blood bank / blood depot with access restricted to designated trial staff. The trial IMP must be stored dry and in accordance with manufacturer's instructions.

The investigator also keeps accurate records of the quantities of trial IMP dispensed and used. The documentation must include date of dispense, patient's sreening number, batch/serial numbers or other identification of trial IMP. The site monitor periodically checks the supplies and documentation of trial IMP to ensure the correct accountability of all trial IMP used. At the end of the trial, all unused trial IMP and all medication containers are returned or destroyed locally according sponsor´s instruction. IMP return and/or destruction must be documented appropriately. The Investigator is responsible for a continuous drug accountability throughout the study. A final drug accountability report must be filed in the Investigator's site file (ISF) at the end of the trial.

6.10 Compliance

Trial IMP is dispensed/administered to the patients by the investigator or another authorized study physician. Compliance is assessed by drug accountability of trial IMP. Details are recorded on the Drug Accountability Form.

7 Trial Methods

7.1 Description of Study Visits

All visits, time points and study assessments are summarized in the Trial Schedule (Table 1).

All participating trial sites will be supplied with study specific visit worksheets that list all assessments / procedures to be completed at each visit.

Patients will be informed about the study and the informed consent obtained before starting any study-related procedures.

The screening/baseline visit may be conducted on up to 7 days before randomisation. Once all screening/baseline assessments are completed, patients will be randomized to one of the two treatment arms. The first CP administration occurs at day 1.

The order of assessments is at the discretion of the investigator except for the study day 1: at day 1, all blood samples for SARS-Cov-2 diagnostic and biobanking must be collected before CP administration / transfusion.

General comments on clinical laboratory assessments

Laboratory safety assessments (hematology, coagulation and blood chemistry) are measured at each visit until hospital discharge.

Urinalysis by dipstick should be performed at screening/baseline and then only if clinically indicated. Microscopic analysis should be performed by the hospital's local laboratory if required.

Any clinically significant abnormal laboratory values should be repeated as clinically indicated and recorded into the eCRF.

Serum or urine pregnancy test is performed for pre-menopausal women of childbearing potential within 14 days prior to the start of study treatment and or prior to randomisation. Tests are performed by the hospital's local laboratory. If results are positive the patient is ineligible/must be discontinued from the study. In the event of a suspected pregnancy during the study, the test should be repeated.

Viral load in nose/throat swabs and antibody titers will be determined for the last time at the time of discharge within 84 days after randomization.

Serology tests for SARS-CoV-2 virus IgG antibodies and SARS-CoV-2 neutralizing antibodies will be performed by a central laboratory: Zentrum für Infektiologie, Virologie, Universitätsklinikum Heidelberg, Im Neuenheimer Feld 344, 69120 Heidelberg.

Instructions regarding sample collection, storage and shipment will be provided in the laboratory manual.

Table 2: Clinical Laboratory Measurements

Category	Lab Parameters
Hematology (local lab)	Haemoglobin, RBC, platelets, WBC with differential cell counts at baseline. If absolute differentials are not available, % differentials should be provided.
Blood chemistry and Coagulation (local lab)	BUN, creatinine, albumin, AST/SGOT, ALT/SGPT, total bilirubin, gGT, AP, LDH, lactate, sodium, potassium, magnesium, calcium, serum uric acid, LDH, Troponin, CK and CK-MB, Quick, PTT, ATIII, D-Dimer, INR, Fibrinogen, Ferritin, transferrin levels and transferrin saturation, CRP, total protein, albumin, IL6, procalcitonin, total IgG, IgA, IgM
Viral Screen (local lab)	HAV, HBV, HCV, and HIV-1
Urinalysis (local lab)	pH, glucose, proteins (qualitative, dipstick accepted)
Others	Serum/urine pregnancy test (local lab)<br>SARS-Cov-2 Viral clearance (positive/negative on standard PCR test and load (semiquantitative via CT value) (central lab, Prof. H.G. Kräusslich)<br>Anti-SARS-Cov-2 antibodies and neutralizing antibody titers (central lab, Prof. H.G. Kräusslich, Prof. Ralf Bartenschlager)<br>Blood samples for Biobanking (central lab, Biobank Med V, PD Dr. K. Kriegsmann)

CROSS-OVER OPTION into experimental arm:

Cross-Over starts with baseline visit and follows the primary schedule of the study as described in the trial schedule (Table 1).

7.2 Methods of Data and Sample Collection

7.2.1 Data Collection and Handling

All findings including clinical and laboratory data are documented by the investigator or an authorized member of the study team in the patient's medical record and in the electronic case report forms (eCRFs). The investigator at the clinical site is responsible for ensuring that all sections of the eCRFs are completed correctly and that entries can be verified against source data. The eCRFs have to be filled out according to the specified CRF Completion Guidelines. The correctness of entries in the eCRFs is confirmed by dated signature of the responsible local principal investigator or deputy principal investigator.

7.2.2 Sample Collection and Handling

For translational analysis samples are obtained at the times indicated in Table 1. Transport is done by TNT courier service or a similar operator providing the needed transport conditions. Further details are provided in the laboratory manual.

Shipment address for Translational research:

Universitätsklinikum Heidelberg, Zentrum für Infektiologie, Department für Infektiologie, Virologie, Im Neuenheimer Feld 344, 69120 Heidelberg.

7.3 Measurement of Efficacy Parameters

The endpoints are analysed according to established criteria as suggested by the WHO.

7.3.1 Seven point ordinal scale

The seven point ordinal scale value is determined at the time of study inclusion and afterwards until discharge within 84 days after randomization or end of trial. This endpoint was also recommended by the WHO R&D Blueprint expert group (Coronavirus disease (COVID-2019) R&D. Geneva: World Health Organization (https://www.who.int/blueprint/priority-diseases/key-action/novel-coronavirus/en/). The seven-category ordinal scale [21] consists of the following categories: 1, not hospitalized with resumption of normal activities; 2, not hospitalized, but unable to resume normal activities; 3, hospitalized, not requiring supplemental oxygen; 4, hospitalized, requiring supplemental oxygen; 5, hospitalized, requiring nasal high-flow oxygen therapy, noninvasive mechanical ventilation, or both; 6, hospitalized, requiring ECMO, invasive mechanical ventilation, or both; and 7, death.

This scale is clearly defined and values will be obtained daily for each patient until day 28, thereafter weekly until day 56, then on day 70 and finally at day 84. A change in two points is clinically meaningful in either direction. For example, a patient with an improvement from #4 (oxygen supplementation, hospitalized) to #2 (discharged, no oxygen) is clearly relevant. Also, a change from #5 (high flow oxygen) to #3 (hospitalized but no supplemental oxygen) is clearly relevant as well. Of note, inclusion criteria of the trial determine that all patients to be included are either in #4 or #5 at time of inclusion into the trial.

Physicians or nurses record the scale value daily at the same time. The time can be fixed individually for each patient. Recordings are obtained +/- one hour of the intended time.

7.3.2 Oxygen saturation (SaO_2) while breathing ambient air

Oxygen saturation while breathing ambient air will only be analysed for patients with an ordinal scale value below 5. Patients requiring supplemental oxygen are going to be analyzed for SaO_2 while breathing ambient air. If SaO_2 levels at ambient air drop below 90%, supplementary oxygen is immediately provided again and the amount of oxygen (liter/min) will be recorded that is required to keep SaO_2 above 94%. Patients at ordinal scales at 5 or higher will not be analysed this way.

7.3.3 Laboratory parameters

Nose/throat swabs are collected at day 0 and day 1 (before first CP administration) and subsequently at day 2, 3, 5, 7, 10, 14, 28 or until discharge. Serum for SARS-Cov-2 diagnostic is collected at baseline and subsequently at day 3, 7, 14 and once during the follow-up period (between day 35 and day 84). Serum collection on the following days is optional: day 1, 2, 3, 10 and 28. Details on sample collection and schedule will be provided in the laboratory manual.

Nasopharyngeal swabs are analysed for SARS-CoV-2 RNA viral load by semi-quantitative real-time RT-PCR in the laboratory of Prof. H.G. Kräusslich (Center for Infectious diseases, University Hospital Heidelberg). Patient serum obtained at the different time points is stored

at -20° C until further use. Antibodies directed against SARS-CoV-2 are going to be analysed in recipient serum before (day1) and after (day 3) treatment. IgG Antibodies are detected in standard ELISA tests. Further, analyses for neutralizing antibodies are performed by Prof. Dr. Ralf Bartenschlager (DKFZ, Heidelberg). Cytokine analyses is performed in the laboratory of PD Dr. Niels Halama (NCT Heidelberg) with the Bio-Plex Pro Human Cytokine 48-Plex Screening Panel (Biorad, Munich, Germany). To evaluate the effects of CP onto inflammation and cytokine levels, a first analysis is performed after the first randomized 40 patients. All other serum specimens are analyzed at the end of trial.

7.4 Measurement of Further Parameters

Vital signs

Height, weight, temperature and blood pressure are collected. Height (in cm) is measured at baseline only. Weight (in kg), temperature (in grade centigrade), and blood pressure/pulse are measured at baseline. Oxygen saturation is regularly assessed by pulse oximetry or invasive analysis if applicable.

Electrocardiogram (ECG)

A 12-lead ECG is performed at baseline as well as additionally at the investigator’s discretion.

Twelve-lead ECGs is obtained after the patient has been rested in a supine position for at least 5 minutes in each case. The Investigator or designated physician reviews the paper copies of each of the timed 12-lead ECGs on each of the study days when they are collected.

8 Ancillary and Post Trial Care

Study ends for individual patients on day 84 (experimental arm and standard arm without cross over), day 94 (standard arm in case of cross over), or death. A telephone survey of patients (if applicable) or treating physician is conducted weekly after hospital discharge before day 84 (experimental arm and standard arm without cross over) or day 94 (standard arm in case of cross over).

9 Assessment of Safety

9.1 Specification of Safety Parameters

9.1.1 Adverse Events

According to GCP, an adverse event (AE) is defined as follows: Any untoward medical occurrence in a patient administered a pharmaceutical product and which does not necessarily have a causal relationship with this treatment. An AE can therefore be any unfavorable and unintended sign (including an abnormal laboratory finding), symptom, or disease temporally associated with the use of a medicinal (investigational) product, whether or not related to the medicinal (investigational) product.

An AE may be:

- New symptoms/ medical conditions
- New diagnosis
- Changes of laboratory parameters
- Intercurrent diseases and accidents
- Worsening of medical conditions/ diseases existing before inclusion into the trial
- Recurrence of disease

Increase of frequency or intensity of episodical diseases.

Each AE must be reported only once, indicating the worst CTC (Version 5.0) grade. If an event stops and later restarts, all occurrences must be reported.

A pre-existing disease or symptom is not considered an adverse event unless there is an untoward change in its intensity, frequency or quality. This change is documented by an investigator.

Surgical procedures themselves are not AEs; they are therapeutic measures for conditions that require surgery. The condition for which the surgery is required may be an AE. Planned surgical measures permitted by the clinical trial protocol and the condition(s) leading to these measures are not AEs, if the condition leading to the measure was present prior to inclusion into the trial.

AEs are classified as "non-serious" or "serious".

9.1.2 Serious Adverse Event

A serious adverse event (SAE) is one that at any dose:

- Results in death
- Is life-threatening (the term life-threatening refers to an event in which the patient was at risk of death at the time of event and not to an event which hypothetically might have cause death if it was more severe)
- Requires patient hospitalization or prolongation of existing hospitalization
- Results in persistent or significant disability/ incapacity
- Results in a congenital anomaly/ birth defect.

Is medically significant (e.g. suspected transmission of an infectious agent via medicinal product). Moreover there are other situations - such as important medical events that may not be immediately life threatening or result in death or hospitalisation but may jeopardize the patient or may require intervention to prevent one of the other outcomes listed above.

9.1.3 Expectedness

An ‘unexpected’ adverse event is one the nature or severity of which is not consistent with the expected side-effects of Convalescent Plasma Infusions mentioned in 6.3. Furthermore, reports which add significant information on specificity or severity of a known adverse reaction are counted as ‘unexpected’ events.

9.1.4 Suspected Unexpected Serious Adverse Reaction (SUSAR)

SAEs that are both suspected, i.e. possibly related to IMP, and ‘unexpected’, i.e. the nature and/or severity of which is not consistent with the applicable product information are to be classified as Suspected Unexpected Serious Adverse Reactions (SUSARs).

In case if either the investigator, who primarily reported the SAE, or the second assessor classify the SAE as ‘suspected’ (“reasonable possibility” or “unknown”, see below) and the SAE is also unexpected, it is categorized as a SUSAR.

All SUSARs are subject to an expedited reporting to the responsible ethics committee(s), the competent authority PEI and to all participating investigators.

9.2 Period of Surveillance and Documentation

All AEs reported by the patient or detected by the investigator are collected during the trial. AEs must also be documented in the patient’s medical records. Whenever possible, the investigator records the main diagnosis instead of the signs and symptoms normally included in the diagnoses.

In this trial, **all AEs that occur after signature of the informed consent** are documented on the pages provided in the CRF. AEs must be followed up from first plasma administration up to 28 days after last plasma administration or until all drug-related toxicities have been resolved, whichever is later, or until the investigator assesses AEs as “chronic” or “stable”.

Each AE must be reported, indicating the worst CTC (Version 5.0) grade. If an event stops and later restarts, all occurrences must be reported. A specific procedure for definition and reporting of SAEs is described in Section 9.3.

9.2.1 Grading of AEs

The grading of AEs in this trial is carried out on the basis of the 5-grade scale defined in the CTCAE v5.0.

**Grade 1:**	Mild; asymptomatic or mild symptoms; clinical or diagnostic observations only; intervention not indicated.
**Grade 2:**	Moderate; minimal, local or noninvasive intervention indicated; limiting age-appropriate instrumental ADL*
**Grade 3:**	Severe or medically significant but not immediately life-threatening; hospitalization or prolongation of hospitalization indicated; disabling; limiting self-care ADL**.
**Grade 4:**	Life-threatening consequences; urgent intervention indicated.
**Grade 5:**	Death related to AE

A Semi-colon indicates ‘or’ within the description of the grade.
Activities of Daily Living (ADL)
*Instrumental ADL refer to preparing meals, shopping for groceries or clothes, using the telephone, managing money, etc.
**Self-care ADL refer to bathing, dressing and undressing, feeding self, using the toilet, taking medications, and not bedridden.

The grading of all AEs listed in the CTCAE v5.0 is based on the information contained therein. The grading of all other AEs, i.e. those which are not listed in the CTCAE v5.0 is performed by a responsible investigator.

9.2.2 Coherence between AEs and the IMP

The Investigator must provide an assessment of causal relationship of each of the clinical trial IMPs to each AE according to the following scale:

**Y** (Yes)	There is a reasonable possibility that the IMP/s caused the AE.
**N** (No)	There is no reasonable possibility that the IMP/s caused the AE and other causes are more probable.
**Uk** (Unknown)	Only to be used in special situations where the Investigator has insufficient information (i.e., the patient was not seen at his/her centre) if none of the above can be used.

9.2.3 Outcome of AEs

The outcome of an AE at the time of the last surveillance is classified as:

**Recovered/ resolved**	All signs and symptoms of an AE disappeared without any sequels at the time of the last interrogation.
**Recovering/ resolving**	The intensity of signs and symptoms has been diminishing and/ or their clinical pattern has been changing up to the time of the last interrogation in a way typical for its resolution.
**Not recovered/ not resolved**	Signs and symptoms of an AE are mostly unchanged at the time of the last interrogation.
**Recovered/ resolved with sequel**	Actual signs and symptoms of an AE disappeared but there are sequels related to the AE.
**Fatal**	Resulting in death. If there are more than one AE only the adverse event leading to death (possibly related) is characterized as ‘fatal‘.
**Unknown**	The outcome is unknown or implausible and the information cannot be

supplemented or verified.

9.2.4 Action taken

The action taken is assigned to one of the following categories:

**Dose not changed**	No change in the dose
**Dose reduced**	Reduction in the dose
**Temporary discontinuation**	Temporary discontinuation of treatment
**Drug withdrawn**	Discontinuation of treatment
**Unknown**	The information is unknown or implausible and it cannot be supplemented or verified.
**Not applicable**	The question is implausible (e.g. the patient is dead).

9.2.5 Countermeasures

The term ‘Countermeasures’ refers to the specific actions taken to treat or alleviate adverse events or to avoid their sequels. The following categories are used to categorize the countermeasures to adverse events:

**None**	No action taken
**Drug treatment**	Newly-prescribed medication or change in dose of a medication
**Others**	Other countermeasures, e.g. an operative procedure

9.3 Reporting of Serious Adverse Events by Investigator

All SAEs must be reported by the investigator to the responsible Safety Officer at the KKS Heidelberg (on behalf of sponsor) within 24 hours after the SAE becomes known using the "Serious Adverse Event" form. The initial report must be as complete as possible, including details of the current illness and (serious) adverse event as well as an assessment of the causal relationship between the event and the trial medication. The reporting is performed by faxing of a completed ‘SAE Form’ to the KKS Heidelberg.

Fax-number: + 49 (0)6221 56 33687

9.4 Expedited Reporting

SUSARs are to be reported to the ethics committee(s), regulatory authorities and to all participating investigators within regulative defined timelines, i.e. they are subject to an expedited reporting.

All SAEs are forwarded by e-mail immediately (not later than 24 hours after receipt) by the responsible person at KKS Heidelberg to the coordinating investigator or the scientific coordinators in order to perform a second assessment. The coordinating investigator or the scientific coordinators fill out a ‘Second Assessment Form’ for each SAE and return it by e-mail to the KKS Heidelberg within 48 hours.

E-mail: V-KKS.SAE@med.uni-heidelberg.de

The ‘Second Assessment Form’ contains the following information:

I) Assessment of relationship between SAE and IMP
II) Assessment of expectedness of SAE (derived from IB or SPC)
III) Assessment of relationship between SAE and the underlying disease
IV) Statement if the benefit/ risk assessment for the trial did change as a result of SAE.

The expedited reporting is carried out by KKS Heidelberg.

9.7 Emergency Treatment

During and following a patient’s participation in the trial, the investigator should ensure that adequate medical care is provided to a patient for any AE, including clinically significant laboratory values. The investigator should inform a patient when medical care is needed for intercurrent illness(es) of which the investigator becomes aware.

9.7 Events of special interest

In cases of **transfusion reactions** after plasma transfusion e.g. anaphylaxis or hemolysis or transfusion related lung injury (TRALI), the safety desk of the KKS should be informed immediately by faxing of a completed SAE form (see above).

9.8 Deaths

All deaths that occur during the study, or before EOS, must be reported as follows:

- Death should be documented in the eCRF and should be reported as an SAE.
- Where death is not due (or not clearly due) to SARS-CoV-2, the AE causing the death must be reported to the Safety Officer at the KKS Heidelberg within 24 hours after the death becomes known using the "Serious Adverse Event" form. (9.1.2 Serious Adverse Event). The report should contain a comment regarding the co-involvement of SARS-Cov-2 disease, if appropriate, and should assign main and contributory causes of death

Deaths with an unknown cause should always be reported as a SAE.

10 Statistical Considerations

10.1 Study Design

This is a randomized open-label, multicenter phase-II study in patients with severe SARS-CoV-2 disease subjects

Randomisation (rf. also to chapter 6.8):

Randomisation is done either into experimental arm E or into standard arm S in a 1:1 ratio using block randomisation stratifed for the factor “patient group”, defined by group 1 (patients with hematological malignancies, history of stem cell transplantation and/or active cancer therapy – incl. chemotherapy, radiotherapy, surgery – within the last 24 months or less.), group 2 (drug induced immunosuppression not meeting the criteria of group 1), group 3 (age ≥ 50 - 75 years and meeting neither the criteria of group 1 nor group 2 and Lymphopenia < 0.8 x G/l and/or D-dimer > 1µg/mL and group 4 (age > 75 years and meeting neither the criteria of group 1 nor group 2).

10.2 Analysis Variables

10.2.1 Primary endpoint and primary estimand

The main purpose of the study is to assess the time from randomization until an improvement within 84 days defined as two points on a seven point ordinal scale or live

discharge from the hospital in high-risk patients (group 1 to group 4) with SARS-CoV-2 infection requiring hospital admission by infusion of plasma from subjects after convalescence of a SARS-CoV-2 infection or standard of care. .

In the Addendum to the ICH E9 guideline (final version), the estimands framework is recommended as clear and transparent definition of "what is to be estimated" (International Council for Harmonization 2019). An estimand is defined through the treatment, the targeted population, the variable, a specification of how to handle intercurrent events (postrandomization events) and a population-level summary. In the following, the primary estimand corresponding to the primary objective is described.

The primary estimand corresponding the primary objective is defined as follows:

Treatment: Infusion of frozen CP (on two days of intervention from two different donors) vs. standard of care (as described in 5.7).

Population: The targeted population is defined through the in- and exclusion criteria.

Variable: Time from randomisation to clinical improvement within 84 days by two points on a seven point ordinal scale or live discharge from the hospital.

Post-randomisation events: live discharge from the hospital is incorporated into the variable definition (composite strategy), death from any cause within 84 days after randomization without previous improvement is taken into account by censoring deceased patients at day 84 (see sections 10.3 and 10.5.4 for rationale)); treatment switch will be ignored (treatment policy strategy, see sections 10.3 and 10.5.4 for rationale); event-free patients at the end of the follow-up period are censored and drop-outs are censored at the last observation (hypothetical strategy).

Summary measure: Hypothesis testing is conducted using a log-rank test. The summary measure for effect quantification is the hazard ratio for the endpoint "clinical improvement by two points or live discharge" between the two treatment arms (which is estimated using Cox regression).

10.3.2 Secondary Endpoints

Overall survival, defined as the time from randomization until death from any cause

28-day, 56-day and 84-day overall survival rates

SARS-CoV-2 viral clearance and load as well as antibody titres.

Requirement mechanical ventilation at any time during hospital stay (yes/no)

Time until discharge from randomization.

Viral load, changes in antibody titers and cytokine profiles are analysed in an exploratory manner using paired non-parametric tests (before – after treatment).

10.2.3 Safety Endpoints

This includes all AEs, their severity, SAEs, the relation of AEs to the study treatment, dose modifications for toxicity and discontinuation of study treatment during the trial phase. Toxic effects are graded according to the National Cancer Institute Common Toxicity Criteria (CTCAE) version 5.0.

10.3 Sample Size Calculation

Based on recently published data [21] median time to an improvement defined as two points on a seven point ordinal scale or live discharge from the hospital is assumed to be at day 16 from randomization. This median estimate considers patients who died without an improvement as censored at day 84 (right-censoring), which ensures that those patients are considered as "not improved" over the whole observation period. In our trial, we follow the same analysis strategy as in Cao *et al.*, 2020 who also censored deceased patients at the end of follow-up [21].

We assume that based on the mechanism of action (neutralizing antibodies) a rapid improvement is induced by fresh frozen plasma from subjects after convalescence of SARS-CoV-2 infection on two days from two different donors with a median time of 10 days (HR=1.6). Assuming exponentially distributed event times, these assumptions correspond to a hazard of $\lambda_C = 0.043$ for the control arm, while the corresponding hazard in the experimental arm should amount to $\lambda_E = 0.043 \cdot 1.6 = 0.69$.

While it is allowed for patients from the control arm to switch to the experimental arm starting at day 10 (see section 6.8), we assume this will not have a major effect on the median time of clinical improvement for these patients, since we expect that plasma infusions will have a larger advantage if given early. While methods for handling patients switching from control to treatment group are available, they are subject to strong and unverifiable assumptions [26]. Hence, we will ignore treatment switch in the primary analysis, representing a treatment policy strategy (see Section 10.2.1), which can be regarded as a conservative approach since switching is only allowed from the control to the experimental arm. However, in supplementary analyses of the primary endpoint, we will consider such a hypothetical scenario in which a treatment effect is estimated for the case that patients would not have switched by means of inverse probability censoring weighting according to Robins & Finkelstein [27], relying on the assumption that there are no unmeasured confounders influencing the probability to switch, and a rank preserving structural failure time (RPSFT) model [28], assuming a common treatment effect which remains constant over time, regardless when treatment is initiated.

Since patients might die before experiencing a clinical improvement or being discharged from the hospital, death will be taken into account via censoring deceased patients at last day of follow-up (day 84). Based on recently published data [21], the mortality rate is assumed to be about 20% in the control arm. Thus, we expect that the cumulative improvement rate of the control group will not become larger than 0.8. Hence, fewer improvement events are expected during the follow-up of 84 days than under the assumption of an exponential distribution, since the improvement curves will flatten at some point. In case of the control group, the flattening is expected to occur at day 38, since the assumed cumulative improvement rate at day 38 amounts to $\exp(-\lambda_C \cdot 38) = \exp(-0.043 \cdot 38) \approx 0.8$. Accordingly, we conservatively assume a follow-up length of 38 days for the sample size calculation to properly estimate the probability of experiencing the event of interest, even though the actual follow-up for each patient will be longer.

To detect the assumed difference between treatment groups using a log-rank test comparing the cumulative improvement curves for the primary endpoint using a significance level of 5% (two-sided) with a power of 80%, a total number of 174 patients is required for the entire trial (87 patients per group) when additionally considering a dropout rate of 5%, meaning that n=164 patients who do not prematurely drop out of the study are required to be enrolled. The required number of events which was calculated using the formula by Schoenfeld, amounts to 142. It is expected that adjusting for the covariate “patient group” in the analysis will lead to an increase in power. Sample size calculation was done conservatively assuming an equally long follow-up period of 38 days for every patient. Sample size calculation was done using RPACT v 2.0.6.

For the sample size calculation, it was assumed that there would be a consistent median time to improvement and overall survival rate across all 4 patient groups. Since this might not necessarily be the case, and it could be likely that patients in group 1 and 2 might have a shorter median time to improvement, we conducted a simulation study to explore the robustness of our model under varying assumptions for the median event times. For our simulation study, we assumed exponentially distributed event times, with a median time to improvement of 16 days across all patient groups in the control group (med_C^1= med_C^2 = med_C^3 = med_C^4=16)), while we assumed differing median times to improvement in the patient groups 1 & 2 compared to patient groups 3 & 4 (med_E^1= med_E^2 , med_E^3 = med_E^4) in the experimental group. We considered a sample size of n=164 evaluable patients for the whole

trial, with 42 patients belonging to groups 1 and 3 each, and 40 patients belonging to groups 2 and 4 each corresponding to patient group membership probability of about 25% for all four groups. Also, we assumed a 38-day mortality rate of 20% in the control arm, while we assumed that the corresponding mortality rate in the experimental arm amounted to 13% (corresponding to a HR for overall survival of 1.6) across all four patient strata assuming exponentially distributed survival times. We simulated 10,000 trials per scenario corresponding to a maximum standard error of sqrt(0.5*0.5/10000)=0.005 for the simulated power.

We used a stratified log-rank test to assess whether the cumulative improvement curves in the two treatment groups (experimental/control) would be equal at a two-sided significance level of α=0.05 stratifying for the factor "patient group" for analysis. The analysis of the simulated datasets was done using the PROC LIFETEST procedure in SAS v9.4. The results are shown in Table 3.

Table 3: Power for the RECOVER trial considering varying treatment effects across patient groups, assuming medC1= medC2 = medC3 = medC4=16 days

Median time to improvement in intervention group for patient groups 1 and 2 in days	Median time to improvement in intervention group for patient groups 1 and 2 in days	Simulated Power 1-β
med_E^1= med_E^2=10	med_E^1= med_E^2=10	0.785
med_E^1= med_E^2=9	med_E^1= med_E^2=11	0.794
med_E^1= med_E^2=8	med_E^1= med_E^2=12	0.813

Assumptions: α=0.05 (two-sided), 10,000 simulations per scenario, n=164 patients not prematurely dropping out of the trial enrolled.

The results in Table 3 show that the aspired power of 80% is approximately achieved in all scenarios, with an even increased power in case of a treatment effect differing between patient groups in the experimental group. This indicates that our planned analysis strategy will achieve the aspired power even under varying treatment effects across the patient strata, when the "average" median time to improvement in the experimental group still amounts to 10 days.

10.4 Analysis Populations

10.4.1 Full Analysis Population

The Full Analysis Population (FAP) includes all randomised patients with treatment groups assigned in accordance with the randomisation, regardless of the treatment actually received. Patients who were randomised but did not subsequently receive treatment are included in the Full analysis population. The analysis of data using the Full analysis population therefore follows the Intention-to-Treat principles (ITT).

10.4.2 Per Protocol Population

In the per protocol population, patients with important protocol deviations are excluded. Definition of important protocol deviations are given in the statistical analysis plan (SAP).

10.4.3 Safety Population

All enrolled patients who received treatment are subjected to the safety analysis. Patients will be evaluated with regard to the treatment actually received, meaning that patients who cross

over from the control to the experimental arm will be evaluated in the experimental arm from day 10 onwards. Details of the safety analysis are specified in the statistical analysis plan (SAP).

10.5 Statistical Methods

10.5.1 General Considerations

The statistical evaluation is carried out under the supervision of the Supervising Statistician. Statistical analysis is based on the International Conference on Harmonization (ICH) Guidelines "Structure and Content of Clinical Study Reports" and "Statistical Principles for Clinical Trials". All statistical procedures are done according to the current Standard Operating Procedures (SOPs) of the Institute of Medical Biometry and Informatics, University of Heidelberg (IMBI).

All data recorded in the eCRF describing the sample, the efficacy and the safety are analyzed descriptively. Categorical data are presented in contingency tables with frequencies and percentages. Continuous data are summarized with at least the following: missing values, median, quartiles, mean, standard deviation, minimum and maximum.

The detailed methodology for the statistical analysis is described in the statistical analysis plan (SAP), which is finalized before database lock. Statistical analysis is performed using SAS v9.4 or higher.

10.5.2 Demographic and other Baseline Characteristics

Patients' disposition and reasons for ending the study are presented in frequency distribution tables and individual data listing. Descriptive statistics of the baseline characteristics are generated across all treated patients. Frequency distributions are presented for the categorical variables. Summary statistics and the number of assessed patients are calculated, as appropriate, for the quantitative variables. Individual data is presented in listings.

Categorical baseline characteristics, like sex, concomitant illness at trial initiation, and concomitant treatment maintained, is summarized by frequency tables. Summary statistics such as mean, standard deviation, median, first and third, quartile, as well as minimum and maximum, are provided for quantitative variables like age, weight, laboratory values.

10.5.3 Study Therapy, Treatment Compliance, and Follow-up

Summary statistics are presented for the amount and dosage of CP received, dose modifications for toxicity, discontinuation and withdrawal from study treatment, as well as drop-out from the follow-up.

10.5.4 Analysis of the Primary Endpoint

The primary analysis will assess the null hypothesis "the cumulative improvement curves for the primary endpoint in the experimental and control arm are equal", i.e. H_0: $S_E = S_C$ against the alternative hypothesis H_1: $S_E \neq S_C$ at a two-sided significance level of α=5%. This will be achieved by using the log-rank test stratified for the factor "patient group" as used in the randomisation procedure. The event "death from any cause" will be handled by censoring those patients at day 84 (in analogy to the approach of Cao et al, NEJM 2020). Using this approach ensures that deceased patients are considered as "not improved" over the whole observation period of 84 days. The hazard ratio for treatment group will be determined using a Cox regression model stratifiying for the factor "patient group" together with a 95% confidence interval.. The cumulative improvement curves are calculated using the Kaplan-Meier method together with 95% log-log-type confidence bands, and will be calculated separately for both treatment groups. In addition, cumulative improvement curves will also be calculated separately for each patient group per treatment group.

The fact that some patients from the control group might switch to the experimental group at day 10 will be ignored in the primary analysis, representing a treatment policy approach

according to the ICH E9 (R1) addendum on estimands [30]. Patients who drop out during the trial or are lost to follow-up are taken into account as censored observations. The primary analysis will be based on the FAS according to the ITT principle.

A supplementary analysis of the primary endpoint will involve the inverse probability censoring weighting (IPCW) approach to estimate a treatment effect in the hypothetical scenario that patients from the control group had not switched to the experimental group [27]. This type of model requires to estimate the probability for a patient to switch to the experimental group based on (time-dependent) covariates, which in our case will be the baseline factor “patient group”, together with the time-varying factors “seven point ordinal scale measuring clinical improvement” and “viral load” over time. It should be noted that this model implicitly assumes that all relevant confounders predicting whether a patient switches from control to treatment have been included into the model, an assumption which is hardly verifiable, especially for such a recently emerged disease about which we currently only have limited understanding, thus being the reason why we did not choose this as the strategy for the primary analysis.

As another supplementary analysis intending to estimate the treatment effect in the hypothetical scenario which assumes that control patients had not switched to the treatment group, a rank preserving structural failure time (RPSFT) model will be fitted [28]. This model assumes a common treatment effect, meaning that the treatment effect is the same for all individuals (with respect to time spent on treatment) regardless of when treatment is received. Since this is another assumption which is hardly verifiable, and because we especially assume that patients will have a larger treatment effect when plasma is given early, we consider this approach as a supplementary analysis only.

Further sensitivity analyses of the primary endpoint include an analysis based on the per protocol population.

10.5.5 Analysis of the Secondary Endpoints

Overall survival is analyzed by using a Cox regression model adjusting for treatment and patient group, determining hazard ratios with 95% confidence intervals and (descriptive) p-values. Survival estimates are calculated using the Kaplan-Meier method together with 95% log-log-type confidence bands, and the 28-day survival rate will be given for both treatment groups together with 95% confidence intervals. Analogously as for the supplementary analysis of the primary endpoint, an IPCW model and a RPSFT model will additionally be fitted for the secondary endpoint overall survival to assess the potential treatment effect in case patients had not switched from the control to the experimental arm.

Time until discharge will be assessed similarly to the primary endpoint, providing Kaplan-Meier estimates for the cumulative discharge rate for both treatment groups and conducting a log-rank test stratified for the factor “patient group”, and censoring patients who died from any cause at day 84 in analogy to the analysis of the primary endpoint. Analogously as for the sensitivity analysis of the primary endpoint, an IPCW and a RPSFT model will additionally be fitted for the secondary endpoint ‘time until discharge’ to assess the potential treatment effect in case patients had not switched from the control to the experimental arm.

The secondary endpoint “requirement of mechanical ventilation (yes/no)” is analyzed by means of a logistic regression model adjusting for the factors treatment and patient group. Also, absolute and relative frequencies will be given for this endpoint, together with 95% confidence intervals.

The secondary endpoints SARS-CoV-2 viral load and antibody titres will be assessed over time by means of linear mixed models adjusting for treatment group, patient group, and

baseline value as fixed factors, as well as time as repeated factor using an unstructured covariance matrix, calculating least square means estimates and 95% confidence intervals.

While it is indirectly assumed by these models that data will be missing at random, i.e. that the missingness can be explained by observed covariate and outcome values, we will assess the robustness of this assumption by means of sensitivity analyses by means of pattern mixture models assuming that data will be not missing at random.

For all secondary endpoints, the analysis will be conducted based on the FAS and as a sensitivity analysis based on the per protocol population.

Safety Analysis

The assessment of safety is based mainly on the frequency of adverse events and on the number of laboratory values that fall outside of pre-determined ranges and/or show prominent worsening from baseline during the study phase. Safety analysis is based on the safety population. Adverse events are summarized by presenting the number and percentage of patients having any adverse events or serious adverse events, and having each individual adverse event, and by determining and summarizing the maximum individual toxicity grade (over all forms of toxicity). Furthermore, the most common AEs (those occurring in at least 10% of the treatment group) are determined. Any other information collected (e.g. severity or relatedness to study drug) are summarized as appropriate. Laboratory data are summarized by presenting summary statistics of raw data and change from baseline values. Incidence rates are summarized along with two-sided Clopper-Pearson 95% confidence intervals and analyzed by (descriptive) chi-squared tests.

Other Analyses

- Patients' disposition and reasons for ending the study are presented in frequency distribution tables and individual data listing.

Individual data are presented in listings.

10.5.6 Handling of Missing Data

For patients with incomplete follow-up, time to last follow-up date is used as the censoring time in the analysis of time-to-event data. Missing data of continuous outcomes over time will be handled via the multi-level approach making the implicit assumption that data are missing at random, thus not requiring any direct imputation of missing continuous data. The robustness of this assumption will be explored in sensitivity analyses by means of pattern mixture models assuming that data are not missing at random. Otherwise, no imputation of missing data will be conducted.

11 Data Management

11.1 Data Collection

As used in this protocol, the term Case Report Form (CRF) should be understood to refer either to a paper form or an electronic data record or both, depending on the data collection method used in this trial.

The data collection will be performed using an eCRF in accordance with the German Corona Consensus Data Set (GECCO).

Data collection using the eCRF can only be done by authorized persons. All study data are password-protected. The eCRF provides several checks for completeness and consistency. Each entry or change of data is tracked with name and exact date. When data has been entered, reviewed, edited and Source Data Verification (SDV) performed, the investigator is

notified to sign the CRF electronically as per agreed project process. A copy of the electronic-CRF will be archived at the study site.

All findings including clinical and laboratory data are documented by the investigator or an authorized member of the study team in the patient's medical record and in the case report form (CRF). The investigator is responsible for ensuring that all sections of the CRF are completed correctly and that entries can be verified by source data. The CRF has to be filled out according to the specified CRF Completion Guidelines.

11.2 Data Handling

Data entries undergo an automatic online check for plausibility and consistency. In case of implausibility, 'warnings' are produced. A responsible investigator is obliged either to correct the implausible data or to confirm its authenticity, and to give appropriate explanation. If not corrected, the data is flagged, enabling a convenient check of all questionable entries. A responsible monitor checks all flagged data and generates questions that are sent back to the responsible investigator. The investigator has to resolve all 'discrepancies'.

Further checks for plausibility, consistency, and completeness of data are performed after completion of the study. Queries are generated on the basis of these checks, combined with a visual control by a responsible monitor/data manager.

All missing data or inconsistencies are reported back to the sites and clarified by the responsible investigator. If no further corrections are to be made in the trial database it is declared closed and used for statistical analysis.

All data management activities are done according to the current Standard Operating Procedures (SOPs) of the IMBI.

11.3 Storage and Archiving of Data

According to §13 of the German GCP-Regulation all important trial documents (e.g. CRFs) will be archived for at least 10 years after the trial termination. Responsible for archiving TMF including protocol, CRFs and reports is the CI.

The investigator(s) will archive all trial data (source data and Investigator Site File (ISF) including Patient Identification List and relevant correspondence) according to the section 4.9 of the ICH Consolidated Guideline on GCP (E6) and to local law or regulations. The Patient Identification List will be archived for at least 15 years after trial termination.

If the investigator relocates, retires, or for any reason withdraws from the study, the Sponsor should be prospectively notified. The study records must be transferred to an acceptable designee, such as another investigator, another institution, or to the Sponsor. The investigator must obtain Sponsor´s written permission before disposing of any records, even if archiving requirements have been met.

12 Ethical and Legal Aspects

12.1 Good Clinical Practice

The procedures set out in this trial protocol, pertaining to the conduct, evaluation, and documentation of this trial, are designed to ensure that all persons involved in the trial abide by Good Clinical Practice (ICH-GCP E6 (R2)) and the ethical principles described in the current version of the Declaration of Helsinki. The trial is carried out in keeping with local legal and regulatory requirements.

12.2 Patient Information and Informed Consent

Before being admitted to the clinical trial, the patient must consent in written form to participate after the nature, scope, and possible consequences of the clinical trial have been explained in a form understandable to him or her. The original personally signed and dated Informed Consent Form must be kept on file by the investigator(s), and documented in the case report form.

A copy of the signed informed consent document must be given to the patient. The documents must be in a language easily understandable to the patient and must clearly state who informed the patient, which is confirmed by the dated signature of the responsible investigator.

If new safety information results in significant changes in the risk/benefit assessment, or if changes are made in the protocol, the consent form should be reviewed and updated if necessary. All patients (including those already being treated) should be informed of the new information and must give their written informed consent to continue the study.

12.3 Confidentiality

The data obtained in the course of the trial is treated pursuant to the applicable Data Protection Law (EU General Data Protection Regulation – GDPR - 2016/679), the Federal Data Protection Act (Bundesdatenschutzgesetz, BDSG), the State Data Protection Act of Baden-Württemberg (Landesdatenschutzgesetz, LDSG BW) as well as § 40 (2a) AMG.

During the clinical trial, patients are identified solely by means of an individual identification code (Patient ID). Storage of trial findings on a computer are done in accordance with local data protection law and are handled in strictest confidence. For protection of these data, organizational procedures are implemented to prevent distribution of data to unauthorized persons. The appropriate regulations of local data legislation are fulfilled in its entirety.

The patient consents in writing to relieve the investigator from his/her professional discretion in so far as to allow inspection of original data for monitoring purposes by health authorities and authorized persons (inspectors, monitors, auditors). Authorized persons (clinical monitors, auditors, inspectors) may inspect the patient-related data collected during the trial, ensuring the data protection law.

The investigator maintains a patient identification list (Patient IDs with the corresponding patient names) to enable records to be identified.

Patients who did not consent to circulate their pseudonymized data are not included into the trial.

12.4 Responsibilities of Investigator

The Principal Investigator should ensure that all persons assisting with the trial are adequately informed about the protocol and any amendments, the trial treatments, and their trial-related duties and functions.

The Principal Investigator should maintain a list of investigators and other appropriately qualified persons to whom he or she has delegated significant trial-related duties (Log of Staff).

The investigator(s) should support monitoring, auditing and inspections as described in sections 14.1 and 14.2.

12.5 Approval of Trial Protocol and Amendments

Before the start of the trial, the trial protocol, informed consent document, and any other appropriate documents are submitted to the independent Ethics Committee (EC) as well as to the competent authority. A written favorable vote of the EC and an (implicit) approval by the competent authority are a prerequisite for initiation of the clinical trial. The statement of EC should contain the title of the trial, the trial code, the trial site, and a list of reviewed

documents. It must mention the date on which the decision was made and must be officially signed by a committee member.

Before the first patient is enrolled in the trial, all ethical and legal requirements must be met. All planned substantial changes (see §10, (1) of German GCP-Ordinance) are submitted to EC and the competent authority in writing as amendments. They have to be approved by the EC and the competent authority.

The Coordinating Investigator or the NCT Trial Center, and if applicable the investigator(s) keep a record of all communication with the EC and the regulatory authorities.

12.6 Continuous Information to Independent Ethics Committee

Pursuant to the German Drug Law (AMG) and the GCP Ordinance, the EC and the competent authority are informed of all suspected serious unexpected adverse reactions (SUSARs) and all AEs resulting in death or being live-threatening which occur during the trial. Both institutions are informed in case the benefit-risk assessment did change or any others new and significant hazards for patients' safety or welfare did occur. Furthermore, a report on all observed serious adverse events (SAEs) is submitted once a year (Development Safety Update Report (DSUR)).

The EC and the regulatory authorities must be informed of the end of the study. They are provided with a summary of trial results within one year after EOS.

12.7 Notification of Regulatory Authorities

The local regulatory authorities as responsible for each particular investigator and the competent authority are informed before the beginning, during and at the end of the trial according to §67 AMG and §13 GCP-V. Each investigator is obliged to notify his/ her local regulatory authority and the competent authority according §67 AMG and §12 (1, 2, 6) GCP-V if nothing else is agreed upon by contract between investigator and sponsor.

12.8 Registration of the Trial

Prior to the beginning of the clinical phase (FPI) the coordinating investigator registers the trial at a public accessible clinical trial register having the status of a primary register according to the International Clinical Trials Registry Platform (ICTRP) and correspondingly is listed at the International Clinical Trials Registry Platform of the World Health Organization (WHO, http://www.who.int/ictrp/en/). The requirements are fulfilled by the European Clinical Trials Register and submission of EMA Module 1 (Clinical Trial Application Form).

The registration is a prerequisite for a publication in many peer-reviewed journals (see Uniform Requirements for Manuscripts Submitted to Biomedical Journals by the International Committee of Medical Journal Editors; (http://www.icmje.org/ publishing_10register.html).

12.9 Insurance

According to § 40 AMG, the sponsor has to subscribe to an insurance policy which covers in its terms and provisions its legal liability for injuries caused to participating persons. The insurance policy also covers any damage done to the patient, even if the harm done arises out of strictly following the procedures described in this protocol and abiding as applicable law and professional standards. The insurance was taken out at **HDI Global SE**, Niederlassung Düsseldorf (insurance number: **Versicherungsschein-Nr.: 5701031003018**, Policy: **Anmelde-Nr.: 14012020107** maximum limit: € 500.000 per participating person).Any impairment of health which might occur in consequence of trial participation must be notified to the insurance company. The patient is responsible for notification. The insured person must agree to help clarify the cause and the extent of damage with all appropriate measures. He is also obliged to take measures himself to reduce damage as much as possible. During the conduct of the trial, the patient must not undergo other clinical treatment except for cases of emergency. The patient is bound to inform the investigator immediately about any adverse

events and additionally drugs taken. The terms and conditions of the insurance must be delivered to the patient.

The insurance company has to be informed about all amendments that could affect patients' safety, and must also receive the actual version of the informed consent.

13 Quality Assurance

13.1 Monitoring

Monitoring is done routinely by personal visits from a clinical monitor according to SOPs of the KKS. The monitor will ensure that the clinical trial is conducted according to the protocol and regulatory requirements by review of source documents, entries into the CRFs and essential documents. The monitor will document the visits in a report for the sponsor. The site will be provided with a follow-up letter of the findings and the necessary actions to be taken.

The investigator must allow the monitor to verify all essential documents and must provide support at all times to the monitor.

If personal visits are not possible due to COVID-19 restrictions alternative measures, e.g. phone visits, might be an alternative option.

Details of monitoring will be defined in the monitoring plan.

13.2 Inspections/ Audits

Regulatory authorities and an auditor authorized by the sponsor may request access to all source documents, CRF, and other trial documentation. Direct access to these documents must be guaranteed by the investigator, who must provide support at all times for these activities.

14 Agreements

14.1 Financing of the Trial

The trial is co-financed by the BMBF program on emergency research funding for COVID-19. and the funds of Heidelberg University Hospital.

14.2 Declaration of Interests

Before the start of the trial, the investigator discloses to the sponsor any proprietary or financial interests he or she might hold in the sponsors/ a funding company, in the investigational product(s), or any commercial organization being involved in the clinical trial. The investigator has also to confirm that he/she has not entered into any financial arrangement whereby the value of compensation paid could affect the outcome of the clinical trial.

The investigator agrees to update this information in case of significant changes.

14.3 Dissemination Policy

14.3.1 Access to data

After the trial has been completed and published, it is planned to make trial data available for re- and meta-analyses. An appropriate repository is defined at the end of the trial.

14.3.2 Reports

The Biostatistician prepares the final trial report together with the Coordinating Investigator within 12 months after the end of the study (EOS).

Annual safety reports (DSURs) are prepared by the pharmacovigilance officer together with the Coordinating Investigator in accordance with legally required timeframes; data reconciliation is carried out where necessary and possible together with the data management of the NCT Trial Center based on already available CRF-AE data.

14.3.3 Publication

All information concerning the trial is confidential before publication. Trial results will be published in medical journals.

15 Signatures

The present trial protocol was subject to critical review and has been approved in the present version by the persons undersigned. The information contained is consistent with:

The current benefit-risk assessment of the investigational medicinal product

Moral, ethical, and scientific principles governing clinical research as set out the principles of GCP and in the applicable version of Declaration of Helsinki.

Date: Signature: ______________________________

16 Declaration of Investigator

I have read the above trial protocol and confirm that it contains all information to conduct the clinical trial. I pledge to conduct the clinical trial according to the protocol.

I will enroll the first patient only after all ethical and regulatory requirements are fulfilled. I pledge to obtain written consent for trial participation from all patients before enrolment.

I know the requirements for accurate reporting of serious adverse events, and I pledge to document and notify such events as described in the protocol.

I pledge to retain all trial-related documents and source data as described.

Date: ______________________

Signature: ______________________

Name (block letters): ______________________

Trial Center (address): ______________________

____

____

17 References

1 WHO.https://www.who.int/blueprint/priority-diseases/key-action/novel-coronavirus/en/. (2020).

2 Yu, J., Ouyang, W., Chua, M. L. K. & Xie, C. SARS-CoV-2 Transmission in Patients With Cancer at a Tertiary Care Hospital in Wuhan, China. *JAMA Oncol*, doi:10.1001/jamaoncol.2020.0980 (2020).

3 Liang, W. *et al.* Cancer patients in SARS-CoV-2 infection: a nationwide analysis in China. *Lancet Oncol* **21**, 335-337, doi:10.1016/s1470-2045(20)30096-6 (2020).

4 Wu, Z. & McGoogan, J. M. Characteristics of and Important Lessons From the Coronavirus Disease 2019 (COVID-19) Outbreak in China: Summary of a Report of 72314 Cases From the Chinese Center for Disease Control and Prevention. *Jama*, doi:10.1001/jama.2020.2648 (2020).

5 UpToDate.com. https://www.uptodate.com/contents/coronavirus-disease-2019-covid-19#. (2020).

6 Rosenbaum, L. Facing Covid-19 in Italy - Ethics, Logistics, and Therapeutics on the Epidemic's Front Line. *N Engl J Med*, doi:10.1056/NEJMp2005492 (2020).

7 Zhou, B., Zhong, N. & Guan, Y. Treatment with convalescent plasma for influenza A (H5N1) infection. *N Engl J Med* **357**, 1450-1451, doi:10.1056/NEJMc070359 (2007).

8 Yeh, K. M. *et al.* Experience of using convalescent plasma for severe acute respiratory syndrome among healthcare workers in a Taiwan hospital. *J Antimicrob Chemother* **56**, 919-922, doi:10.1093/jac/dki346 (2005).

9 Hung, I. F. *et al.* Convalescent plasma treatment reduced mortality in patients with severe pandemic influenza A (H1N1) 2009 virus infection. *Clin Infect Dis* **52**, 447-456, doi:10.1093/cid/ciq106 (2011).

10 Luke, T. C., Kilbane, E. M., Jackson, J. L. & Hoffman, S. L. Meta-analysis: convalescent blood products for Spanish influenza pneumonia: a future H5N1 treatment? *Ann Intern Med* **145**, 599-609, doi:10.7326/0003-4819-145-8-200610170-00139 (2006).

11 Shen, C. *et al.* Treatment of 5 Critically Ill Patients With COVID-19 With Convalescent Plasma. *Jama*, doi:10.1001/jama.2020.4783 (2020).

12 Duan, K. *et al.* The feasibility of convalescent plasma therapy in severe COVID-19 patients: a pilot study. *medRxiv*, 2020.2003.2016.20036145, doi:10.1101/2020.03.16.20036145 (2020).

13 Chen, G. *et al.* Clinical and immunologic features in severe and moderate Coronavirus Disease 2019. *J Clin Invest*, doi:10.1172/jci137244 (2020).

14 Desborough, M. *et al.* Fresh frozen plasma for cardiovascular surgery. *Cochrane Database Syst Rev*, Cd007614, doi:10.1002/14651858.CD007614.pub2 (2015).

15 Wan, Y. *et al.* Molecular Mechanism for Antibody-Dependent Enhancement of Coronavirus Entry. *J Virol* **94**, doi:10.1128/jvi.02015-19 (2020).

16 Cheng, Y. *et al.* Use of convalescent plasma therapy in SARS patients in Hong Kong. *Eur J Clin Microbiol Infect Dis* **24**, 44-46, doi:10.1007/s10096-004-1271-9 (2005).

17 Chen, L., Xiong, J., Bao, L. & Shi, Y. Convalescent plasma as a potential therapy for COVID-19. *Lancet Infect Dis* **20**, 398-400, doi:10.1016/s1473-3099(20)30141-9 (2020).

18 Roback, J. D. & Guarner, J. Convalescent Plasma to Treat COVID-19: Possibilities and Challenges. *Jama*, doi:10.1001/jama.2020.4940 (2020).

19 Casadevall, A. Passive antibody administration (immediate immunity) as a specific defense against biological weapons. *Emerg Infect Dis* **8**, 833-841, doi:10.3201/eid0808.010516 (2002).

20 Casadevall, A. & Pirofski, L. A. The convalescent sera option for containing COVID-19. *J Clin Invest*, doi:10.1172/jci138003 (2020).

21 Cao, B. *et al.* A Trial of Lopinavir-Ritonavir in Adults Hospitalized with Severe Covid-19. *N Engl J Med*, doi:10.1056/NEJMoa2001282 (2020).
22 Soo, Y. O. *et al.* Retrospective comparison of convalescent plasma with continuing high-dose methylprednisolone treatment in SARS patients. *Clin Microbiol Infect* **10**, 676-678, doi:10.1111/j.1469-0691.2004.00956.x (2004).
23 Mair-Jenkins, J. *et al.* The effectiveness of convalescent plasma and hyperimmune immunoglobulin for the treatment of severe acute respiratory infections of viral etiology: a systematic review and exploratory meta-analysis. *J Infect Dis* **211**, 80-90, doi:10.1093/infdis/jiu396 (2015).
24 van Griensven, J. *et al.* Evaluation of Convalescent Plasma for Ebola Virus Disease in Guinea. *New England Journal of Medicine* **374**, 33-42, doi:10.1056/NEJMoa1511812 (2016).
25 Pandey, S. & Vyas, G. N. Adverse effects of plasma transfusion. *Transfusion* **52 Suppl 1**, 65s-79s, doi:10.1111/j.1537-2995.2012.03663.x (2012).
26 Watkins, C., Huang, X., Latimer, N., Tang, Y. & Wright, E. J. Adjusting overall survival for treatment switches: commonly used methods and practical application. *Pharm Stat* **12**, 348-357, doi:10.1002/pst.1602 (2013).
27 Robins, J. M. & Finkelstein, D. M. Correcting for noncompliance and dependent censoring in an AIDS Clinical Trial with inverse probability of censoring weighted (IPCW) log-rank tests. *Biometrics* **56**, 779-788, doi:10.1111/j.0006-341x.2000.00779.x (2000).
28 Robins, J. M. & Tsiatis, A. A. Correcting for non-compliance in randomized trials using rank preserving structural failure time models. *Communications in Statistics - Theory and Methods* **20**, 2609-2631, doi:10.1080/03610929108830654 (1991).
29 Pintilie, M. Dealing with competing risks: testing covariates and calculating sample size. *Statistics in Medicine* **21**, 3317-3324, doi:10.1002/sim.1271 (2002).
30 International Council for Harmonization. E9(R1): Addendum on estimands and sensitivity analysis in clinical trials. International Council for Harmonization. E9(R1): Addendum on estimands and sensitivity analysis in clinical trials, 2019. (2019).
31 Fine, J. P. & Gray, R. J. A Proportional Hazards Model for the Subdistribution of a Competing Risk. *Journal of the American Statistical Association* **94**, 496-509, doi:10.2307/2670170 (1999).
32 Lambden, S., Laterre, P. F., Levy, M. M. & Francois, B. The SOFA score—development, utility and challenges of accurate assessment in clinical trials. *Critical Care* **23**, 374, doi:10.1186/s13054-019-2663-7 (2019).

18 Appendices

18.1 Ordinal Scale for Clinical Improvement

According to Cao et al, NEJM 2020 [21].

1. not hospitalized with resumption of normal activities;

2. not hospitalized, but unable to resume normal activities;

3. hospitalized, not requiring supplemental oxygen;

4. hospitalized, requiring supplemental oxygen;

5. hospitalized, requiring nasal high-flow oxygen therapy or, noninvasive mechanical ventilation

6. hospitalized, requiring ECMO, invasive mechanical ventilation, or both

7. death.

18.2 Sequential Organ Failure Assessment (SOFA) score

Assessment of the Sequential Organ Failure Assessment (SOFA) score [32]

Respiratory system

PaO2/FiO2 (mmHg)	Score
> 400	0
< 400	1
< 300	2
< 200 with respiratory support	3
< 100 with respiratory support	4

Nervous system

Glasgow Coma Scale	Score
15	0
13–14	1
10–12	2
6–9	3
< 6	4

Cardiovascular system

Mean arterial pressure (MAP) OR administration of vasopressors required	Score
MAP > 70 mmHg	0
MAP < 70 mm/Hg	1
Dopamine ≤ 5 µg/kg/min or dobutamine any dose	2
Dopamine > 5 µg/kg/min OR epinephrine ≤ 0.1 µg/kg/min OR norepinephrine 0.1 µg/kg/min	3
Dopamine > 15 µh/kg/min OR epinephrine > 0.1 µg/kg/min OR norepinephrine >0.1 µg/kg/min	4

Liver

Bilirubin (mg/dl) [µmol/L] SOFA score	Score
< 1.2 (< 20)	0
1.2–1.9 [20–32]	1
2.0–5.9 [33–101]	2
6.0–11.9 [102–204]	3
> 12.0 [> 204]	4

Coagulation

Platelets ×103/ml SOFA score	Score
> 150	0
< 150	1
< 100	2
< 50	3
< 20	4

Kidneys

Creatinine (mg/dl) [µmol/L]; urine output	Score
< 1.2 [< 110]	0
1.2–1.9 [110–170]	1
2.0–3.4 [171–299]	2
3.5–4.9 [300–440] (or urine output < 500 ml/day)	3
> 5.0 [> 440]; urine output < 200 ml/day	4

https://www.mdcalc.com/sequential-organ-failure-assessment-sofa-score

Clinical Trial Protocol

A Randomized Open label Phase-II Clinical Trial with or without Infusion of Plasma from Subjects after Convalescence of SARS-CoV-2 Infection in High-Risk Patients with Confirmed Severe SARS-CoV-2 Disease*

RECOVER (Study Acronym)

Clinical TrialCode: **SARS-CoV-2CP-HD-001**

EudraCT No.: **2020-001632-10**

Clinicaltrials.gov:

Clinical Phase: Phase II

Version and date: Version 3.0 Mar 8th, 2021

Sponsor: *Ruprecht-Karls-Universität Heidelberg, Medical Faculty*

Represented in law by Universitätsklinikum Heidelberg and its acting Commercial Director Ms. Katrin Erk

Im Neuenheimer Feld 672,

69120 Heidelberg, Germany

CONFIDENTIAL: This protocol contains confidential information and is intended solely for the guidance of the clinical investigation. This protocol may not be disclosed to parties not associated with the clinical investigation or used for any purpose without the prior written consent of the Principal Investigator/Coordinating Investigator.

* Referring to the amendment of protocol V3.0_Mar 8 2021, the title of the clinical trial protocol was not changed in the ongoing study. The administration of plasma from vaccinated donors in addition to convalescent donors is clearly described in the protocol text.

Responsibilities

Coordinating Investigator
(Leiter der klinischen Prüfung*)

Prof. Dr. Carsten Müller-Tidow
University Hospital Heidelberg
Internal Medicine V
Hematology,Oncology and Rheumatology
Im Neuenheimer Feld 410
69120 Heidelberg
Phone: +49 6221 - 56 8001
Fax: +49 6221 - 56 33639
E-Mail:
Carsten.Mueller-Tidow@med.uni-heidelberg.de

* According to § 40 German Drug Law (AMG)

Sponsor

Ruprecht-Karls-Universität Heidelberg,
Medical Faculty
represented by Universitätsklinikum
Heidelberg and its Commercial Director
Ms Katrin Erk
Im Neuenheimer Feld 672
69120 Heidelberg, Germany
Phone: +49 (0)6221 56 7081
Fax: +49 (0)6221 56 5925
E-Mail: Katrin.Erk@med.uni-heidelberg.de

Co-Coordinating Investigators
(Medizinische Studien-Koordinatoren)

Prof. Dr. Uta Merle, Internal Medicine IV, HD
Prof. Dr. Markus Weigand, Anesthesiology, HD
PD Dr. Claudia Denkinger, Tropic Medicine, HD
Prof. Dr. Hans-Georg Kräusslich, Infectious Diseases, Virology, HD
Prof. Dr. Maria Vehreschild, Div. Infectious Dis, University Hospital Frankfurt
Prof. Dr. Lars Bullinger, Hematology/Oncology, Charite, Berlin

Scientific Coordination
(Wissenschaftliche Leiter)

Prof. Dr. Richard Schlenk, NCT Trial Center
Prof. Dr. Michael Schmitt, Internal Medicine V

Project Management
Dr. Ulrike Schäkel, Internal Medicine V
Dr. Carine Djuika Fokou, NCT Trial Center
Dr. Julia Gáll, NCT Trial Center

Biostatistician

Dr. Johannes Krisam
Institute of Medical Biometry and Informatics
Im Neuenheimer Feld 130.3
69120 Heidelberg
Phone: +49 6221 - 56 32630
Fax: +49 6221 - 56 4195
E-Mail: krisam@imbi.uni-heidelberg.de
and
Prof. Dr. Richard F. Schlenk
National Center for Tumor Diseases (NCT)
Trial Center
Im Neuenheimer Feld 130.3
69120 Heidelberg

Trial Coordination

NCT Trial Center
Im Neuenheimer Feld 130/3
69120 Heidelberg, Germany
Phone: +49 (0)6221 56 6228
Fax: +49 (0)6221 56 5863
E-Mail: recover@nct-heidelberg.de

Phone: +49 6221 - 56 6228
Fax: +49 6221 - 56 5863
E-Mail: richard.schlenk@nct-heidelberg.de

Clinical Monitoring and Pharmakovigilance
Coordination Centre for Clinical Trials (KKS)
Heidelberg University Hospital
Im Neuenheimer Feld 130/3
69120 Heidelberg, Germany
Phone:+49 (0)6221 56-34507
Fax: +49 (0)6221 56-33508
E-Mail: V-KKS.SAE@med.uni-heidelberg.de
Karsten.thelen@med.uni-heidelberg.de

Data Management
Dr. Jacek Stermann
Institute of Medical Biometry and Informatics (IMBI)
Im Neuenheimer Feld 130/3
69120 Heidelberg, Germany
Phone: +49 (0)6221 56- 37235
Fax: +49 (0)6221 56-4195
E-Mail: stermann@imbi.uni-heidelberg.de

BioBank Med V
PD Dr. Katharina Kriegsmann, MBA
Universitätsklinikum Heidelberg
Innere Medizin V: Hämatologie,Onkologie and Rheumatologie
Im Neuenheimer Feld 410
69120 Heidelberg
Tel: +49 6221 – 56 37238
Fax: +49 6221 – 56 8588
E-Mail: katharina.kriegsmann@med.uni-heidelberg.de

Labor AG Halama
PD Dr. Niels Halama
Med. Onkologie NCT
Im Neuenheimer Feld 460
69120 Heidelberg
Tel.:: +49 6221 – 56 38397
Fax: +49 6221 – 56 7225
E-Mail: niels.halama@nct-heidelberg.de

Zentrum für Infektiologie
Prof. Dr. H.-G. Kräusslich (Leiter)
Universitätsklinikum Heidelberg
Zentrum für Infektiologie
Department für Infektiologie, Virologie
Im Neuenheimer Feld 344
69120 Heidelberg
Tel: +49 6221 – 56 5001
Fax: +49 6221 – 56 5006
E-Mail: Hans-Georg.Kraeusslich@med.uni-heidelberg.de

Prof. Dr. Ralf Bartenschlager
AG Molekulare Virologie
Im Neuenheimer Feld 344
69120 Heidelberg
Tel: +49 6221 - 56 4225
Fax: +49 6221 - 56 4570
E-Mail: ralf.bartenschlager@med.uni-heidelberg.de

Protocol Writing Committee:

Heidelberg: Lead: Prof. Dr. Richard Schlenk, Head, NCT and Hematology Study Center, and in alphabetical order (all University Heidelberg, if not otherwise specified)

Prof. Dr. Ralf Bartenschläger, DKFZ Heidelberg
PD Dr. Claudia Denkinger, Head, Division of Tropical Medicine,
Dr. Carine Djuika Fokou, NCT Trial Center
Prof. Dr. Peter Dreger, Director of Stem Cell Program, Medicine V
Dr. Julia Gáll, NCT Trial Center
PD Dr. Niels Halama, National Center for Tumor Diseases, NCT-Heidelberg
Dr. Irina Idler, Ethics and Patient Oriented Care in Oncology, NCT-Heidelberg
Prof. Dr. Dirk Jäger, National Center for Tumor Diseases, Heidelberg
Prof. Dr. Meinhard Kieser, Director, Institute of Medical Biometry and Informatics (IMBI)
Prof. Dr. Hans-Georg Kräusslich, Dean of the Medical Faculty, Director of the Infectious Disease Center
PD Dr. Katharina Kriegsmann, Resident, Head, Biobank Medicine V
Dr. Johannes Krisam, Institute of Medical Biometry and Informatics (IMBI)
Dr. Albrecht Leo, Transfusion Specialist, Head, Blood Bank, Vice-Director Institute for Clinical Transfusion Medicine and Cell Therapy (ITKZ) gGmbH,
PD Dr. Tobias Liebregts, Attending, ICU Hematology, Medicine V
Prof. Dr. Hannes Lorenz, Head, Division of Rheumatology, Medicine V
Prof. Dr. Uta Merle, Director, Department of Gastroenterology and Infectious Diseases
Prof. Dr. Stefan Meuer, Director, IKTZ gGmbH,
Prof. Dr. Carsten Müller-Tidow, Director, Medicine V
Dr. Caroline Pabst, Resident, Medicine V
Dr. Ulrike Schäkel, Resident, Study Center, Medicine V
PD Dr. Anita Schmitt, Transfusion Specialist, QP Vice-Head, GMP Core Facility, Medicine V
Prof. Michael Schmitt, QP, Head, GMP Core Facility, Medicine V
Dr. Margarida Souto Carneiro, Senior Research Fellow, Division of Rheumatology,
Dr. Stefan Weber, Resident, Division of Tropical Medicine
Prof. Dr. Markus Weigand, Director, Dept. of Anaesthesiology

Frankfurt :

Prof. Dr. Maria Vehreschild, Johann-Wolfgang-Goethe-University
PD Dr. Timo Wolf, Frankfurt, Johann-Wolfgang-Goethe-University
Prof. Dr. Hubert Serve, Johann-Wolfgang-Goethe-University

Berlin: Prof. Dr. Lars Bullinger, Director, Department of Hematology and Oncology, Charite

Summary

Convalescent plasma has been proposed to decrease mortality in patients with SARS, Ebola and in epidemic influenza. Evidence is based on case reports, retrospective analyses and small clinical trials. The timing, when best to give plasma is unclear. Convalescent plasma was used during the COVID-19 outbreak in the Hubei province. Plasma was usually applied in critically ill patients with severe ARDS that may restrict the potential benefit.

This trial will analyze the effects of convalescent/vaccine-boosted plasma from recovered/vaccinated subjects in high-risk patients with SARS-CoV-2 infection. Plasma obtained from subjects after SARS-CoV-2 infection or vaccination with SARS-CoV-2 antibodies will be used in this trial. Patients at high risk for a poor outcome due to underlying disease (group 1, pre-existing or concurrent hematological malignancy or stem cell transplantation and/or active cancer therapy (incl. chemotherapy, radiotherapy, surgery) within the last 24 months or less; group 2, chronic immunosuppression not meeting the criteria of group 1; group 3, age ≥ 50 - 75 years and lymphopenia < 0.8 x G/l and/or D-dimer > 1µg/mL meeting neither the criteria of group 1 nor group 2; group 4, age ≥ 75 years meeting neither the criteria of group 1 nor group 2) are eligible for enrollment. In addition, eligible patients have a confirmed SARS-CoV-2 infection and O_2 saturation ≤ 94% while breathing ambient air. Patients are randomized to receive (experimental arm) or not receive (standard arm) convalescent/vaccine-boosted plasma in two bags (238 - 337 ml plasma each) from different donors (day 1, day 2). A cross over from the standard arm into the experimental arm is possible at day 10 (+ a maximum of 2 days) in case of not improving or worsening clinical condition. The primary endpoint is the time to clinical improvement defined as two points improvement (within 84 days) on a seven point ordinal scale or live discharge from the hospital. Secondary outcomes are mortality, changes in clinical scores, rate of mechanical ventilation, inflammatory cytokine changes and viral load dynamics. This trial systematically assesses the therapeutic role of convalescent/vaccine-boosted plasma for treating SARS-CoV-2 infection in a high-risk patient population.

Zusammenfassung

Das Plasma rekonvaleszenter Patienten soll die Mortalität bei Patienten mit SARS, Ebola und Influenza verringern können. Dabei beruht die Evidenz auf kleineren Fallserien, retrospektiven Analysen und kleinen Klinischen Studien. In China wird Plasma bei schwerkranken Patienten mit COVID-19 eingesetzt ohne dass klare klinische Evidenz vorliegt. Plasma wurde üblicherweise bei kritisch Kranken mit fortgeschrittenem ARDS eingesetzt, was den therapeutischen Benefit fraglich erscheinen läßt.

Diese klinische Studie wird die Effekte von rekonvaleszentem/geimpftem Plasma von Menschen mit überstandener SARS-CoV-2 Infektion oder nach Impfung bei Hoch-Risiko COVID-19 Erkrankung testen. Plasma wird eingesetzt, das nachgewiesene Antikörper gegen SARS-Cov-2 enthält. Patienten mit SARS-Cov-2 Infektion und hohem Risiko für einen schweren Verlauf können in die Studie eingeschlossen werden. Gruppe 1: hämatologische oder onkologische Erkrankung mit/ohne Stammzelltransplantation und/oder laufende Krebstherapie (inkl. Chemotherapie, Strahlentherapie, Operation) innerhalb der letzten 24 Monate; Gruppe 2: Medikamentöse Immunsuppression und nicht in Gruppe 1; Gruppe 3: Alter 50-75 Jahre und nicht in Guppe 1 oder 2 mit mindestens Lymphopenie < 0.8 x G/l oder D-dimere > 1µg/mL; Gruppe 4: Alter ≥ 75 Jahre und nicht in Gruppe 1 oder 2. Alle einzuschliessenden Patienten haben RT-PCR bestätigte SARS-Cov-2 Infektion und O_2 Sättigung ≤ 94% bei Raumluft. Patienten werden randomisiert für rekonvaleszentes/geimpftes Plasma (Experimenteller Arm) mit zwei Beuteln Plasma (238 - 337 ml jeweils) von verschiedenen Spendern (Tag 1 und 2). Im Standardarm wird keine Plasmatherapie gegeben aber jeweils die vom Zentrum festgelegte antivirale und supportive Therapie. Ein Cross-Over vom Standardarm in den experimentellen Arm ist an Tag 10 (plus maximal 2 Tage) möglich im Falle keiner Änderung oder Verschlechterung des klinischen Verlaufs. Primärer Endpunkt ist die Zeit bis zur klinischen Verbesserung entsprechend einer Zwei-Punkte-Verbesserung (innerhalb 84 Tagen) auf einer Sieben-Punkte-Ordinalskala oder Entlassung lebend aus dem

Krankenhaus. Sekundäre Endpunkte sind Sterblichkeit, Änderungen in klinischen Scores, Rate mechanischer Beatmung, Zytokinänderungen und Viruslast über die Zeit. Diese Studie untersucht die therapeutische Bedeutung von rekonvaleszentem/geimpftem Plasma für die Behandlung von COVID-19 bei hohem Risiko für einen schlechten Verlauf.

Table of Contents

RESPONSIBILITIES 2
SUMMARY 5
ZUSAMMENFASSUNG 5
TABLE OF CONTENTS 7
TRIAL SCHEDULE 14
ABBREVIATIONS 18
1 INTRODUCTION 21
1.1 Scientific and Therapeutic Background 21
1.2 Trial Rationale/ Benefit- Risk Assessment 22
1.2.1 Trial Rationale 22
1.2.2 Benefit - Risk Assessment 22
1.3 Reference Committees 22
1.3.1 Data Monitoring Committee (DMC) 22
2 TRIAL OBJECTIVES 22
2.1 Primary Objective 22
2.2 Secondary Objectives 22
3 TRIAL DESIGN 23
4 TRIAL DURATION AND SCHEDULE 23
5 SELECTION OF PATIENTS 24
5.1 Number of Patients and Recruitment 24
5.2 General Criteria for Patients' Selection 24
5.3 Inclusion Criteria 24
5.4 Exclusion Criteria 25
5.5 Criteria for Withdrawal 25
5.5.1 Withdrawal of Patients 25
5.5.2 Handling of Withdrawals 26
5.5.3 Premature Closure of the Clinical Trial or a Single Center 26
5.6 Prior and Concomitant Illnesses 26
5.7 Concomitant Therapy 26
6 INVESTIGATIONAL MEDICINAL PRODUCT (IMP) 27
6.1 General Information about Convalescent/Vaccine-boosted Plasma 27
6.2 Therapeutic / Diagnostic Effects 28
6.3 Known Side Effects of Convalescent Plasma Infusions 28
6.4 Dosage Schedule 28
6.5 Treatment discontinuation 29
6.6 Enrolment, Randomisation, Treatment Assignment 29
6.7 Packaging and Labeling 29
6.8 Cross Over 30
6.9 Supplies and Accountability 30
6.10 Compliance 30
7 TRIAL METHODS 30
7.1 Description of Study Visits 30
7.2 Methods of Data and Sample Collection 32
7.2.1 Data Collection and Handling 32
7.3 Measurement of Efficacy Parameters 33
7.3.1 Seven point ordinal scale 33
7.3.2 Oxygen saturation (SaO_2) while breathing ambient air 33
7.3.3 Laboratory parameters 33
7.4 Measurement of Further Parameters 34
8 ANCILLARY AND POST TRIAL CARE 34
9 ASSESSMENT OF SAFETY 34
9.1 Specification of Safety Parameters 34
9.1.1 Adverse Events 34

9.1.2 Serious Adverse Event ... *35*
9.1.3 Expectedness ... *35*
9.1.4 Suspected Unexpected Serious Adverse Reaction (SUSAR) ... *35*
9.2 Period of Surveillance and Documentation ... 35
9.2.1 Grading of AEs ... *35*
9.2.2 Coherence between AEs and the IMP ... *36*
9.2.3 Outcome of AEs ... *36*
9.2.4 Action taken ... *36*
9.2.5 Countermeasures ... *37*
9.3 Reporting of Serious Adverse Events by Investigator ... 37
9.4 Expedited Reporting ... 37
9.7 Emergency Treatment ... 38
9.7 Events of special interest ... 38
10 STATISTICAL CONSIDERATIONS ... 38
10.1 Study Design ... 38
10.2 Analysis Variables ... 38
10.2.1 Primary endpoint and primary estimand ... *38*
10.3.2 Secondary Endpoints ... *39*
10.2.3 Safety Endpoints ... *39*
10.3 Sample Size Calculation ... 39
10.4 Analysis Populations ... 41
10.4.1 Full Analysis Population ... *41*
10.4.2 Per Protocol Population ... *41*
10.4.3 Safety Population ... *41*
10.5 Statistical Methods ... 42
10.5.1 General Considerations ... *42*
10.5.2 Demographic and other Baseline Characteristics ... *42*
10.5.3 Study Therapy, Treatment Compliance, and Follow-up ... *42*
10.5.4 Analysis of the Primary Endpoint ... *42*
10.5.6 Handling of Missing Data ... *44*
11 DATA MANAGEMENT ... 44
11.1 Data Collection ... 44
11.2 Data Handling ... 45
11.3 Storage and Archiving of Data ... 45
12 ETHICAL AND LEGAL ASPECTS ... 45
12.1 Good Clinical Practice ... 45
12.2 Patient Information and Informed Consent ... 45
12.3 Confidentiality ... 46
12.4 Responsibilities of Investigator ... 46
12.5 Approval of Trial Protocol and Amendments ... 46
12.6 Continuous Information to Independent Ethics Committee ... 47
12.7 Notification of Regulatory Authorities ... 47
12.8 Registration of the Trial ... 47
12.9 Insurance ... 47
13 QUALITY ASSURANCE ... 48
13.1 Monitoring ... 48
13.2 Inspections/ Audits ... 48
14 AGREEMENTS ... 48
14.1 Financing of the Trial ... 48
14.2 Declaration of Interests ... 48
14.3 Dissemination Policy ... 48
14.3.1 Access to data ... *48*
14.3.2 Reports ... *48*
15 SIGNATURES ... 49
16 DECLARATION OF INVESTIGATOR ... 50

17 REFERENCES 51
18 APPENDICES 53
18.1 ORDINAL SCALE FOR CLINICAL IMPROVEMENT 53
18.2 SEQUENTIAL ORGAN FAILURE ASSESSMENT (SOFA) SCORE 54

List of Tables

Table 1: Trial schedule detailing protocol activities to be completed................................ 14
Table 2: Clinical Laboratory Measurements.. 32
Table 3: Power for the RECOVER trial considering varying treatment effects across patient groups, assuming medC1= medC2 = medC3 = medC4=16 days........................... 41

Protocol Synopsis

Title

A Randomized Open label Phase-II Clinical Trial with or without Infusion of Plasma from Subjects after Convalescence of SARS-CoV-2 Infection in High-risk Patients with Confirmed SARS-CoV-2 Disease and Confirmed Severe SARS-CoV-2 Disease

Phase

Phase II

Sponsor

Heidelberg University Hospital
represented in law by its acting Commercial Director Ms Katrin Erk
Im Neuenheimer Feld 672, 69120 Heidelberg, Germany

Coordinating Investigator (LKP)

Prof. Dr. Carsten Müller-Tidow

Trial Site (s)

Multi-centre trial conducted in several German Medical Centres (10 - 15)

Financing/Status of the Sponsor

The trial is co-financed by the BMBF program on emergency research funding for COVID-19 and the funds of Heidelberg University Hospital

Indication

SARS-CoV-2 infection requiring hospital admission

Inclusion Criteria

1. PCR confirmed SARS-CoV-2 infection in a respiratory tract sample.
2. Oxygen saturation (SaO_2) of 94% or less while breathing ambient air or a ratio of the partial pressure of oxygen (PaO_2) to the fraction of inspired oxygen (FiO_2) of less than 300 mm Hg.
3. High risk due to either

   pre-existing or concurrent hematological malignancy and/or active cancer therapy (incl. chemotherapy, radiotherapy, surgery) within the last 24 months or less. (group 1)

and/or

   chronic immunosuppression not meeting the criteria of group 1 (group 2)

and/or

   Age ≥ 50 - 75 years meeting neither the criteria of group 1 nor group 2 (group 3) and at least one of these criteria: Lymphopenia < 0.8 x G/l

and/or
D-dimer > 1µg/mL

and/or
Age ≥ 75 years meeting neither the criteria of group 1 nor group 2 (group 4)

4. Blood hemoglobin concentration ≥ 8g/dl.
5. Provision of written informed consent.
6. Patient is able to understand and comply with the protocol for the duration of the study, including treatment and scheduled visits and examinations.
7. Male or female patient aged ≥ 18 years.
8. Postmenopausal or evidence of non-childbearing status. For women of childbearing potential: negative urine or serum pregnancy test within 14 days prior to study treatment.

Exclusion Criteria

1. Dementia, psychiatric or cognitive illness or recreational drug/alcohol use that in the opinion of the principle investigator, would affect subject safety and/or compliance.
2. Contraindication to transfusion or history of prior reactions to transfusion blood products.
3. Patients with known selective IgA deficiency.
4. Patients with mechanical ventilation and/or extracoporal membrane oxygenation (ECMO) at time of initial inclusion into the trial.
5. Participation in another trial with an investigational medicinal product.
6. Treatment with SARS-CoV-2 convalescent/vaccine-boosted plasma in the past.

Objectives

Primary Objective:

- To assess the time from randomization until an improvement (within 84 days) defined as two points on a seven point ordinal scale or live discharge from the hospital in high-risk patients (group 1 to group 4) with a SARS-CoV-2 infection requiring hospital admission by infusion of plasma from subjects after convalescence of a SARS-CoV-2 infection or after vaccination or standard of care.

Secondary Objectives

1. To assess overall survival, and the overall survival rate at 28, 56 and 84 days.
2. To assess SARS-CoV-2 viral clearance and load, cytokine changes over time as well as antiviral antibody titres.
3. To assess percentage of patients that required mechanical ventilation
4. To assess time from randomization until discharge

Trial Design

Randomized, open-label, multicenter phase II trial, designed to assess the clinical outcome of SARS-CoV-2 disease in high-risk patients (group 1 to group 4) following treatment with anti-SARS-CoV-2 convalescent/vaccine-boosted plasma or standard of care.

Investigational Medicinal Products

RECOVER CT SARS-CoV-2-ab Plasma COVID-19 (HD) obtained from subjects following recovery of a SARS-CoV-2 infection or after vaccination

Sample Size

Total sample size: n=174 patients (considering drop-outs)

Statistical Analysis

The trial is conducted as randomized, open-label, multicenter phase II study comparing clinical outcome in SARS-CoV-2-infected high-risk patients (group 1 to group 4) following treatment with anti-SARS-CoV-2 convalescent/vaccine-boosted plasma or standard of care

Patients are stratified into different groups that will be used for randomization and for statistical analysis. It is expected that 25% of trial participants belong to group 1 including patients with hematological malignancies, history of stem cell transplantation or active cancer therapy (incl. chemotherapy, radiotherapy, surgery) within the last 24 months or less; 25% of trial participants (group 2) consist of patients with drug induced immunosuppression not meeting the criteria of group 1, 25% of trial participant (group 3) consist of patients with either age ≥ 50 -75 years and Lymphopenia < 0.8 x G/l and/or D-dimer > 1µg/mL meeting neither the criteria of group 1 nor group 2 and finally 25% of trial participant (group 4) are beyond > 75 years meeting neither the criteria of group 1 nor group 2.

Based on recently published data (Cao B, et al. NEJM 2020) median time to an improvement defined as two points on a seven point ordinal scale or live discharge from the hospital is assumed to be at day 16 from randomization.

We assume that based on the mechanism of action (neutralizing antibodies) a rapid improvement is induced by fresh frozen plasma from subjects after convalescence of SARS-CoV-2 infection or vaccination on two days from two different donors with a median time of 10 days (HR=1.6).

While it is allowed for patients from the control arm to switch to the experimental arm starting at day 10 (+ 2 days), we assume this will not have a major effect on the median time of clinical improvement for these patients, since we expect that plasma infusions will have a larger advantage if given early. While methods for handling patients switching from control to treatment group are available, they are subject to strong and unverifiable assumptions (Watkins et al. 2013). Hence, we will ignore treatment switch in the primary analysis, representing a treatment policy strategy, which can be regarded as a conservative approach since switching is only allowed from the control to the experimental arm. However, in supplementary analyses of the primary endpoint, we will consider such a hypothetical scenario in which a treatment effect is estimated for the case that patients would not have switched by means of inverse probability censoring weighting according to Robins & Finkelstein (2000), relying on the assumption that there are no unmeasured confounders influencing the probability to switch, and a rank preserving structural failure time (RPSFT) model (Robins & Tsiatis 1991), assuming a common treatment effect which remains constant over time, regardless when treatment is initiated.

To detect the assumed difference between treatment groups using a log-rank test investigating the Kaplan-Meier curves for the event “clinical improvement by 2 points or live discharge from hospital”, taking “death from any cause” into account by right-censoring deceased patients at day 84, using a significance level of 5% (two-sided) with a power of 80%, a total number of 174 patients is required for the entire trial (87 patients per group) when additionally considering a dropout rate of 5%. It is expected that adjusting for the confounder “patient group” in the analysis will lead to an increase in power. Sample size calculation was done assuming an equally long follow-up period of 38 days for each patient

in order to properly estimate the probability of experiencing an event of interest, taking into account that deceased patients will not experience the event of interest (see Section 10.3 for justification).

The primary analysis of the trial will compare the Kaplan-Meier curves for the primary endpoint at two-sided significance level of α=5%. This will be achieved by using a log-rank test stratified for factor “patient group”. The event “death from any cause” will be taken into account by right-censoring deceased patients at the last day of follow-up (day 84).

The fact that some patients from the control group might switch to the experimental group at day 10 (+2 days) will be ignored in the primary analysis. Patients who drop out during the trial or are lost to follow-up are taken into account as censored observations. Analysis will be based on the Full Analysis Population according to the Intention-to-treat principle.

A supplementary analysis of the primary endpoint will involve the inverse probability censoring weighting (IPCW) approach to estimate a treatment effect in the hypothetical scenario that patients from the control group had not switched to the experimental group (Robins & Finkelstein 2000). As another supplementary analysis modeling this hypothetical scenario, a rank preserving structural failure time (RPSFT) model will be fitted (Robins & Tsiatis 1991). Other supplementary analyses include a model by Fine and Gray (1999) targeting the subdistribution hazard for ‘clinical improvement by 2 points or live discharge from hospital’.

Further sensitivity analyses of the primary endpoint include an analysis based on the per protocol population.

Secondary outcomes are:

1. Overall survival and survival rates at days 28, 56 and 84
2. Differences in SARS-CoV-2 viral clearance and load as well as antibody titres
3. Percentage of patients that required mechanical ventilation
4. Time until discharge.

Safety: Safety analyses are performed according to established CTCAE guidelines

Trial Duration and Dates

Total trial duration:	18 months
Duration of the clinical phase:	12 months
First patient first visit (FPFV):	3rd Quarter 2020
Last patient first visit (LPFV):	2nd Quarter 2021
Last patient last visit (LPLV):	3rd Quarter 2021
Trial Report completed:	4th Quarter 2021

Table 1: Trial schedule detailing protocol activities to be completed

Trial Schedule

**Trial Period**	**Screening/ Baseline****	**Treatment**		**Follow-up** (until hospital discharge)											
**Study day**	**≤ 7 days**	**Day 1*****	**Day 2**	**Day 3**	**Day 4**	**Day 5**	**Day 6-9**	**Day 10**	**Day 11-13**	**Day 14**	**Day 15-27**	**Day 28**	**Day 29-55**	**Day 56**	**Day 84**
**Visit window (days)**$^{\alpha}$						± 1		± 1		± 2		± 2		± 2	± 2
**Procedures / Assessments**															
Informed consent[2]	X														
Demographics[3]	X														
Medical history[4]	X														
Smoking history[5]	X														
Signs/symptoms[6]	X							X*							
Vital signs[7]	X	X^{μ}		X		X		X		X					
Physical examination[8]	X							X*							
Clinical Frailty Scale (CFS)	X														
Seven point ordinal scale[9#]	X	X	X	X	X	X	X	X	X	X	X	X	X^{β}	X	X
SOFA-Score[10#]		X^{μ}						X							
Oxygen saturation (SaO_2) while breathing ambient air[11#]	X	X^{μ}		X		X		X		X					
Fraction of Inspired Oxygen (FiO_2)[#]	X	X^{μ}		X		X		X		X					
PaO_2, $PaCO_2$[#]		X^{μ}						X							
Amount of oxygen supplementation[12#]	X	X^{μ}		X		X		X		X					
Cross-over Assessment[13]								X							
ECG[14]	X							X*							

**Trial Period**	**Screening/ Baseline****	**Treatment**		**Follow-up** (until hospital discharge)											
**Study day**	**≤ 7 days**	**Day 1*****	**Day 2**	**Day 3**	**Day 4**	**Day 5**	**Day 6-9**	**Day 10**	**Day 11-13**	**Day 14**	**Day 15-27**	**Day 28**	**Day 29-55**	**Day 56**	**Day 84**
**Visit window (days)**[α]						± 1		± 1		± 2		± 2		± 2	± 2
**Laboratory Tests at *Local Lab***															
Blood collection for Hematology *(local lab)*[15#]	X	X[μ]		X		X		X		X		X		X	X
Blood collection for chemistry and coagulation standard *(local lab)*[16#]	X	X[μ]		X		X		X		X		X		X	X
Blood collection for chemistry and coagulation extended *(local lab)*[17#]		X[μ]						X						X	X
Urinalysis *(local lab)*[18]	X														
Blood collection for HAV, HBV, HCV, HIV-1 screen *(local lab)*[19]	X														
Pregnancy test *(local lab)*[20]	X														
**Viral Diagnostic and Biobanking *(Central Lab, Heidelberg)***															
Nose/throat swabs for SARS-Cov-2_PCR *(central lab)* [21]		X[μ]		X		X		X		X		X			
Blood collection for SARS-Cov-2_Serology *(central lab)*[21]		X[μ]		X		X		X		X		X		X	X
Blood samples for biobanking *(central lab)*[22]		X[μ]		X		X		X		X		X		X	X
Inclusion and exclusion criteria	X	X[μ]						X*							
Enrollment[23]	X														
Randomisation[2]		X[μ]													
**Treament / Convalescent/Vaccine-boosted Plasma**[24]		X	X												
Assessment of Concomitant medications[25]	X	X	X	X	X	X	X	X	X	X	X	X	X	X	X
AE assessments[26]		X	X	X	X	X	X	X	X	X	X	X			

**Footnotes for schedule of events:**
*In case of cross-over #measured until hospital discharge βweekly<br>μbefore CP/PVP administration αVisit windows not applicable for assessment of seven point ordinal scale<br>** All screning/baseline assessments maybe completed on day 1. In this case, all screening/baseline assessments must be completed before randomisation and CP/PVP administered after completion of all other visit procedures.<br>*** Day 1 = day of randomisation into standard arm or day of first plasma administration in the experimental arm
1. **Observation time** for all patients is expected to be at least 3 months after entry into the study. Patients receive convalescent/vaccine-boosted plasma for two days (day 1 and day 2) or standard of care. For patients in the standard arm, cross over is allowed at day 10 (+ 2 days) in case of not improving or worsening clinical condition. There is a regular *follow-up* of 3 months. All discharged patients are followed by regular phone calls (scheduled days: 3, 7, 10, 14, 28, 56, 84). In person visits after discharge are only advised if clinically indicated. Timing of all visits must be based on the date of first CP/PVP administration. The study period ends on the day of the last follow-up visit of the last randomized patient. All Patients with ongoing plasma-related toxicities must be followed until all plasma-related toxicities are resolved.
2. **Informed consent and patient registration**: every patient must sign the informed consent to participate in this trial before starting any trial-related procedures. After signature, a patient ID (three-digit) is generated via registration in the eCRF system. After completion of the screening/baseline visit and assessment of the inclusion and exclusion criteria, **randomisation** is carried out by the responsible investigator **online via https:/randomizer.at** (Uni Graz) with assignment of the random number.
3. **Demographics**: gender, year of birth, ethnicity
4. **Medical history**: date and type of first symptoms and detailed information on pretreatment including type and composition of prior therapy, family history (cancer, cardiovascular disease, diabetes, rheumatological disease), additional medical history on concomitant diseases in particular cancer and autoimmune diseases and their treatment.
5. **Information on smoking;** duration and amout, pack-years
6. **Signs/symptoms:** infection related and unrelated signs and symptoms, in particular cough, myalgia, fatigue, diarrhoea, vomiting,
7. **Vital signs**: WHO/ECOG performance status, temperature (in grade centigrade), blood pressure/pulse, respiratory rate, at baseline: height (in cm), weight (in kg). After hospital discharge the patients are contacted by phone or e-mail and asked about their performance status until 84 days after randomization.
8. **Physical examination:** inspection, lung, cardiac and abdominal auscultation and percussion, palpation of the abdomen and lymph node sites, neurological examination
9. **Seven point ordinal scale:** Assessment of the seven point ordinal scale daily until day 28, weekly until day 56 and finally on day 84. (1, not hospitalized with resumption of normal activities; 2, not hospitalized, but unable to resume normal activities; 3, hospitalized, not requiring supplemental oxygen; 4, hospitalized, requiring supplemental oxygen; 5, hospitalized, requiring nasal high-flow oxygen therapy, noninvasive mechanical ventilation, or both; 6, hospitalized, requiring ECMO, invasive mechanical ventilation, or both; and 7, death.
10. **SOFA-Score:** The sequential organ failure assessment score (SOFA score) is assessed to determine the extent of a person's organ function or rate of failure. It integrates scoring of the respiratory system, nervous system, cardiovascular system, liver, coagulation and kidneys (Appendix). In case of earlier discharge the SOFA-Score must be determined on the day of discharge from hospital.
11. **Oxygen saturation (SaO_2)** while breathing ambient air measured by pulse oximetry. See 7.3.2 for details.

12. Amount of oxygen supplementation: i) supplemental oxygen l/min; nasal high-flow oxygen therapy l/min noninvasive mechanical ventilation, ECMO, invasive ventilation
13. **Cross-over Assessment:** For cross-over, all inclusion / exclusion criteria (except mechanical ventilation/ECMO) must again be met as specified in Chapters 5.3 and 5.4. After Cross over, the visit schedule is restarted at day 1 in the experimental arm. The timing of subsequent visits is based on the day of first CP/PVP administration.
14. **ECG:** 12-lead ECG, at baseline, and thereafter at investigator's discretion
15. **Hematology (local lab):** hemoglobin, RBC, PLT, WBC, Differential cell counts at baseline.
16. **Blood chemistry standard (local lab):** BUN, creatinine, albumin, AST/SGOT, ALT/SGPT, total bilirubin, gGT, AP, LDH, lactate, sodium, potassium, magnesium, calcium, serum uric acid, LDH, Troponin, CK and CK-MB, Quick, PTT, ATIII, D-Dimer, INR, Fibrinogen, CRP.<br>17. **Blood chemistry extended (local lab): Ferritin, transferrin levels and transferrin saturation, total protein, albumin, IL6, procalcitonin, total IgG, IgA, IgM.**
18. **Urinalysis (local lab):** pH, glucose, proteins (qualitative, dipstick accepted): at baseline and at investigator's discretion during treatment.
19. **HAV, HBV, HCV, and HIV-1 testing**: at baseline.
20. **Serum/urine pregnancy test** (local lab). Only for pre-menopausal women of childbearing potential. Must be done within 14 days prior to the start of study treatment and or prior to randomisation.
21. **SARS-Cov-2_PCR nose/throat swabs and SARS-Cov-2_Serology:** Swabs are collected at day 1, 3, 5, 10, 14, 28 or until discharge, and serum at day 1, 3, 5, 10, 14, 28, 56 and 84. Convalescent/vaccine-boosted plasma is administered on day 1 and 2. **On day 1 blood sampling for serology and nose/throat swabs must be done before CP/PVP administration.** Further details will be provided in the lab manual.
22. **Procurement of Samples for Biobanking** at IM-V Heidelberg University Hospital: 7.5 ml serum and 2x 7.5 ml Li-Heparin (only at day 1 and day 14). Further details will be provided in the lab manual.
23. **Enrollment:** eligible patients are enrolled and registered in the eCRF. The investigator is requested to sign that eligibility criteria are met.
24. **Convalescent/vaccine-boosted plasma is administered on days 1-2. On day 1, blood collection for SARS-Cov-2 serology and nasopharyngeal swabs must be done PRIOR CP/PVP administration.**
25. **Concomitant medications** is reported in the relevant CRF pages, including supportive care drugs, prophylaxis, and drugs used for treating AEs or chronic diseases.
26. **AE assessments:** events are documented and recorded continuously. Patients must be followed for AEs up to day 28 or until all drug-related toxicities have been resolved, whichever is later, or until the investigator assesses AEs as "chronic" or "stable". Each AE must be reported, indicating the worst CTC (Version 5.0) grade. If an event stops and later restarts, all occurrences must be reported. A specific procedure for definition and reporting of SAEs is described in the protocol.

Abbreviations

ADE	Antibody Dependent Enhancement
AE	Adverse Event
ALT	Alanine Amino Transferase, also known as SGPT
AMG	Deutsches Arzneimittelgesetz (German Drug Law)
AP	Alkaline Phosphatase
ARDS	Acute Respiratory Distress Syndrome
AST	Aspartate Amino Transferase, also known as SGOT
ATIII	Antithrombin III
BUN	Blood Urea Nitrogen
CFS	Clinical Frailty Scale
CK	Creatine Kinase
CMV	Cytomegalovirus
COVID-19	Coronavirus Disease 2019
CP	Convalescent plasma
CRF	Case Report Form
CRP	C-Reactive Protein
CTC	Common Toxicity Criteria
CTCAE	Common Toxicity Criteria for Adverse Events
DMC	Data Monitoring Committee
DSUR	Development Safety Update Report
EC	Ethics Committee
ECMO	Extracoporal Membrane Oxygenation
ECOG	Eastern Cooperative Oncology Group
ELISA	Enzyme-linked Immunosorbent Assay
EMA	European Medicines Agency
EOS	End of Study
EOT	End of Treatment
FAP	Full Analysis Population
FiO2	Fraction of Inspired Oxygen
FPFV	First Patient First Visit
FSH	Follicle Stimulating hormone
GCP	Good Clinical Practice

GDPR	General Data Protection Regulation
gGT	Gamma-glutamyl transferase
H1N1	Hemagglutinin Type 1 and Neuraminidase Type 1 (Swine Flu)
H5N1	Hemagglutinin Type 5 and Neuraminidase Type 1 (Avian Influenza)
HAV	Hepatitis A Virus
HBV	Hepatitis B Virus
HCV	Hepatitis C Virus
HIV	Human Immune-deficiency Virus.
HR	Hazard Ratio
IC	Informed Consent
ICH	International Council on Harmonization of Technical Requirements for Registration of Pharmaceuticals for Human Use
ICTRP	International Clinical Trials Registry Platform
ICTRP	International Clinical Trials Registry Platform
Ig	Immunoglobulin
IKTZ	Institut für Klinische Transfusionsmedizin und Zelltherapie (Institute for Clinical Transfusion Medicine and Cellular Therapy)
IL-6	Interleukin-6
IMP	Investigational Medicinal Product
IMPD	Investigational Medicinal Product Dossier
IPCW	Inverse Probability Censoring Weighting
ISF	Investigator Site File
ITT	Intention-To-Treat
LDH	Lactate Dehydrogenase
LH	Luteinizing hormone
LKP	Leiter der Klinischen Prüfung (Coordinating Investigator according to AMG)
LPFV	Last Patient First Visit
LPLV	Last Patient Last Visit
MERS	Middle East Respiratory Syndrome
NCT	National Center for Tumor Diseases
PaO_2	Partial Pressure of Oxygen
PCR	Polymerase Chain Reaction
pH	Potential Hydrogen
PLT	Platelet
PRO	Patient Reported Outcome
PTT	Partial Thromboplastin Time

PVP	Post-Vaccination Plasma = vaccine-boosted plasma
RBC	Red Blood Cell
RPSFT	Rank Preserving Structural Failure Time
SAE	Serious Adverse Event
SaO_2	Oxygen saturation
SAP	Statistical Analysis Plan
SARS-CoV	Severe Acute Respiratory Syndrome Coronavirus 2
SDV	Source Data Verification
SGOT	Serum Glutamic-Oxaloacetic Transaminase, also known as AST
SGPT	Serum Glutamic-Pyruvat Transaminase, also known as ALT
SOFA	Sequential Organ Failure Assessment
SOP	Standard Operating Procedures
SUSAR	Suspected Serious Unexpected Adverse Reaction
TACO	Transfusion Associated Circulatory Overload
TMF	Trial Master File
TRALI	Transfusion Associated Lung Injury
WBC	White Blood Cell
WHO	World Health Organization

1 Introduction

1.1 Scientific and Therapeutic Background

The SARS-CoV-2 disease, also known as coronavirus disease 2019 (COVID-19) is a respiratory illness caused by the newly discovered Severe Acute Respiratory Syndrome Coronavirus 2 (SARS-CoV-2). First cases of COVID-19 were reported in December 2019 [1]. The viral infection subsequently rapidly spread worldwide resulting in a pandemic with severe implications on health care systems due to high morbidity and mortality. The infection is characterized by a mild to moderate respiratory illness in most affected patients. However, older people, and patients with concomitant diseases such as chronic respiratory diseases, cardiovascular diseases, cancers, and/or chronic immunosuppression are at high risk to develop severe respiratory distress [2-4].

Current control strategies of SARS-CoV-2 disease are limited to prevention, case monitoring and supportive care. A validated vaccine or curative treatment is not available yet, resulting in disease widespread and increasing mortality [5]. Therefore, novel therapeutic options are urgently needed. Outbreaks have overwhelmed the health care systems in several regions of the world, notably in China (Heibei province), Italy (Lombardy) and in France (Alsace). Patients requiring mechanical ventilation were not intubated due to shortages in ventilators, material and staff [6]. Accordingly, early effective treatment is urgently required to avoid the need for mechanical ventilation.

Over the last past decades, convalescent plasma (CP) has been used for the prevention or treatment of various life-threatening infections, especially respiratory viral infections such as H1N1, H5N1 and the coronaviruses SARS1 and MERS [7-10]. Recently, encouraging results of the treatment of 5 critically ill COVID-19 patients with convalescent plasma have been reported [11]. Furthermore, reports from SARS-CoV-2 vaccination trials suggest, that subjects after the second boost immunization develop high titers of neutralizing antibodies and thus may be excellent candidates for plasma donation[12-14]. There are additional non-peer reviewed reports about benefit in SARS-CoV-2 infected patients [15]. SARS-Cov-2 infection is associated with cytokine changes that may partially drive the pathogenesis of Acute Respiratory Distress Syndrome, ARDS [16]. Plasma therapy has a long standing history with an excellent safety profile [17]. From a theoretical perspective, there are *in vitro* concerns whether antibodies against SARS-CoV-2 might lead to Antibody Dependent Enhancement (ADE) of virus entry into cells [18].

Thus, there is now accumulating evidence suggesting that passive immunotherapy using CP decreases the morbidity and mortality in SARS and epidemic Influenza [9,11,19,20]. However, these data were gained through retrospective studies, case reports, and CP treatment was usually combined with other drugs [21]. Hence, reliable evidence of the association of CP treatment with favourable clinical course – particularly in SARS-Cov-2 infections – has not been properly demonstrated yet.

1.1.2 Passive antibody therapy

The principle of passive antibody therapy using infusions of CP/PVP is based on the fact that following infection with an infectious agent, the infected person produces specific antibodies against the latter. Thus, CP/PVP obtained from subjects, who have recovered from an infection or were successfully vaccinated provides a specific prophylactic or therapeutic weapon against the corresponding infectious agent. Unlike vaccination, passive antibody therapy provides an immediate protection [22,23]. This is of particular importance in an infectious outbreak context, especially for high-risk, patients who might not develop a prompt and adequate immune response due to underlying diseases or concomitant therapies.

1.2 Trial Rationale/ Benefit- Risk Assessment

1.2.1 Trial Rationale

The aim of this randomized phase-II study is to gain evidence on the effect of CP/PVP in the treatment of SARS-CoV-2 infection in high-risk patients. High-risk is defined within 4 groups; group 1, pre-existing or concurrent hematological malignancy and/or active cancer therapy (incl. chemotherapy, radiotherapy, surgery) within the last 24 months or less; group 2, chronic immunosuppression not meeting the criteria of group 1; group 3, age ≥ 50-75 years and meeting neither the criteria of group 1 nor group 2 and lymphopenia < 0.8 x G/l and/or D-dimer > 1µg/mL; group 4, age ≥ 75 years and meeting neither the criteria of group 1 nor group 2). The main focus of the protocol is to assess the clinical outcome in high-risk patients after infusion of CP/PVP in patients with confirmed SARS-CoV-2 infection and a O_2 saturation ≤ 94% while breathing ambient air. Patients requiring mechanical ventilation and/or extracoporal membrane oxygenation (ECMO) are not eligible for the trial.

Based on previous studies, we hypothesize that an infusion of CP/PVP relatively early during the SARS-CoV-2 infection might reduce the severity of the disease rapidly and thus avoid a progression to a more severe disease state and death.

1.2.2 Benefit - Risk Assessment

Currently, clinical management of high-risk COVID-19 patients remains a challenge [5]. In general, treatment of SARS-CoV-2 infections is purely symptomatic and depends on the underlying diseases. After failure of supportive care, there are currently no approved options available for these patients; clinical studies with remdesivir, hydroxychloroquine and tocilizumab are ongoing while a randomized study lopinavir-ritonavir was negative [24].

Taking into consideration the beneficial safety profile, infusion of CP/PVP may offer access to a potentially effective therapy in high-risk patients whose treatment options are limited [23].

1.3 Reference Committees

1.3.1 Data Monitoring Committee (DMC)

The DMC is composed of at least three independent experts, assessing the progress and safety data. The mission of the DMC is to ensure the ethical conduct of the trial and to protect the safety interests of patients in this trial.

The DMC meetings are planned after treatment of 5, 10 and 25 patients in the experimental arm of the study and according to the DMC charter. Based on its review, the DMC provides the sponsor with recommendations.

Further details including DSUR reviews and DMC members is specified in the DMC charter.

2 Trial Objectives

2.1 Primary Objective

- To assess the time from randomization until an improvement within 84 days defined as two points on a seven point ordinal scale or live discharge from the hospital in high-risk patients (group 1 to group 4) with SARS-CoV-2 infection requiring hospital admission by infusion of plasma from subjects after convalescence of SARS-CoV-2 infection or vaccination or standard of care

2.2 Secondary Objectives

- To assess overall survival, and the overall survival rate at 28, 56 and 84 days.
- To assess SARS-CoV-2 viral clearance and load as well as antibody titres.
- To assess percentage of patients that required mechanical ventilation.

- To assess time from randomization until discharge

For detailed endpoints see sections 10.3.2. and 10.3.3

3 Trial Design

This is a multicentre, randomized, open-label, phase II clinical trial designed to evaluate the clinical outcome in high-risk patients with SARS-CoV-2 infection by infusion of plasma from subjects after convalescence of SARS-CoV-2 infection or vaccination.

High-risk is defined as SARS-CoV-2 positive infection with Oxygen saturation at ≤ 94% at ambient air with additional risk features as categorized in 4 groups:

- group 1, pre-existing or concurrent hematological malignancy and/or active cancer therapy (incl. chemotherapy, radiotherapy, surgery) within the last 24 months or less.
- group 2, chronic immunosuppression not meeting the criteria of group 1
- group 3, age ≥ 50 - 75 years meeting neither the criteria of group 1 nor group 2
  and at least one of these criteria:
  Lymphopenia < 0.8 x G/l
  and/or
  D-dimer > 1µg/mL
- group 4, age ≥ 75 years meeting neither the criteria of group 1 nor group 2

The primary end point is the time to clinical improvement within 84 days, defined as the time from randomization to either an improvement of two points on a seven-category symptom ordinal scale or live discharge from the hospital, whichever comes first. A total of 174 patients are planned to be included into the trial.

4 Trial Duration and Schedule

The duration of the trial for each patient is expected to be about 3 months, including two days of intervention (infusion of frozen CP/PVP), followed by a follow-up of 3 months. Furthermore viral load is measured in nasopharagyngeal swabs at day 1, 3, 5, 10, 14, 28 or until hospital discharge within 84 days after randomization.

Patients randomized into the standard arm of the study have the possibility to cross over into the experimental arm of the study starting at day 10 (+ 2 days) in case of not improving or worsening clinical condition.

Conditions leading to patient withdrawal from the study are detailed in Section 5.5 Criteria for Withdrawal.

Treatment response is assessed daily until day 28, thereafter weekly until day 56, and finally at day 84. Primary endpoint is the time to clinical improvement by two points on a seven point ordinal scale or live discharge from the hospital.

A recruitment period of approximately 9 months and an overall study duration of approximately 12 months is anticipated. Recruitment of patients starts in the third quarter of 2020.

The study duration of an individual patient is planned to be 3 months.

After finishing all study-relevant procedures, therapy, and follow-up period, the patient is followed in terms of routine care and treated if necessary.

Total trial duration:	18 months
Duration of the clinical phase:	12 months
First patient first visit (FPFV):	3rd Quarter 2020
Last patient first visit (LPFV):	2nd Quarter 2021
Last patient last visit (LPLV):	3rd Quarter 2021
Trial Report completed:	4th Quarter 2021

5 Selection of Patients

5.1 Number of Patients and Recruitment

As described in Section 10.2 (Size Calculation) a total of 174 patients are planned to be enrolled in the clinical trial.

5.2 General Criteria for Patients' Selection

This clinical trial can fulfill its objectives only if appropriate patients are enrolled. The following eligibility criteria are designed to select patients for whom protocol treatment is considered appropriate. All relevant medical and non-medical conditions should be taken into consideration when deciding whether this protocol is suitable for a particular patient. Any questions regarding a patient's eligibility should be discussed with the Coordinating-, Co-Coordinating-Investigator or the Scientific Coordinator.

5.3 Inclusion Criteria

Both female and male patients are included in this clinical trial. Patients must meet all of the following inclusion criteria to be eligible for enrollment into the study:

1. PCR confirmed SARS-CoV-2 infection in a respiratory tract sample.
2. Oxygen saturation (SaO_2) of 94% or less while breathing ambient air or a ratio of the partial pressure of oxygen (PaO_2) to the fraction of inspired oxygen (FiO_2) of less than 300 mm Hg.
3. High risk due to either

   pre-existing or concurrent hematological malignancy and/or active cancer therapy (incl. chemotherapy, radiotherapy, surgery) within the last 24 months or less (group 1)

   and/or

   chronic immunosuppression not meeting the criteria of group 1 (group 2)

   and/or

   Age ≥ 50 -75 years meeting neither the criteria of group 1 nor group 2 (group 3)
   and at least one of these criteria:
   Lymphopenia < 0.8 x G/l
   and/or
   D-dimer > 1µg/mL

   and/or

   Age ≥ 75 years meeting neither the criteria of group 1 nor group 2 (group 4).
4. Blood hemoglobin concentration ≥ 8 g/dl.
5. Provision of written informed consent.
6. Patient is able to understand and comply with the protocol for the duration of the study, including treatment and scheduled visits and examinations.

7. Male or female patient aged ≥ 18 years
8. Postmenopausal or evidence of non-childbearing status. For women of childbearing potential: negative urine or serum pregnancy test within 14 days prior to study treatment.

   Postmenopausal or evidence of non-childbearing status is defined as:

   - Amenorrheic for 1 year or more following cessation of exogenous hormonal treatments
   - Luteinizing hormone (LH) and Follicle stimulating hormone (FSH) levels in the post menopausal range for women under 50 except in patients with a history of surgical sterilisation (bilateral oophorectomy or hysterectomy)
   - Radiation-induced oophorectomy with last menses > 1 year ago
   - Chemotherapy-induced menopause with > 1 year interval since last menses
   - Surgical sterilisation (bilateral oophorectomy or hysterectomy)
   - Female patients of child bearing potential and male patients with partners of child bearing potential, who are sexually active, must agree to the use of highly effective forms of contraception. This should be started from the signing of the informed consent and continue throughout the study and for 1 month (female patients) / 3 months (male patients) after last infusion of CP/PVP

5.4 Exclusion Criteria

Patients fulfilling any of the following criteria cannot be enrolled in the trial:

1. Dementia, psychiatric or cognitive illness or recreational drug/alcohol use that in the opinion of the principle investigator, would affect subject safety and/or compliance.
2. Contraindication to transfusion or history of prior reactions to transfusion blood products.
3. Patients with selective IgA deficiency.
4. Patients with mechanical ventilation and/or extracoporal membrane oxygenation (ECMO) at time of initial inclusion into the trial. Mechanical ventilation is defined as either NIV – non-invasive ventilation or positive pressure ventilation. Enrollment into another clinical trial evaluating specific therapies for COVID-19 is encouraged.
5. Participation in another trial with an investigational medicinal product.
6. Treatment with SARS-CoV-2 convalescent/vaccine-boosted plasma in the past.

5.5 Criteria for Withdrawal

5.5.1 Withdrawal of Patients

A patient must be withdrawn from the trial treatment or/and all trial-related procedures for the following reasons:

1. At any time at their own request withdrawal of patient's consent to continue therapy. The patient is at any time free to discontinue treatment, without prejudice to further treatment.
2. Changes in medical status of the patient such that the investigator believes that patient safety is compromised or that it would be in the best interest of the patient to stop treatment
3. Pregnancy

A patient may be withdrawn from the trial treatment or/and all trial-related procedures for the following reasons:

1. Non-compliance by the patient with protocol requirements

2. Patient is lost to follow-up. If a patient does not return for scheduled visits, every effort should be made to re-establish contact. In any circumstance, every effort should be made to document patient outcome if possible

Conditions leading to treatment discontinuation are specified in Section 6.5.

If the patient withdraws from the trial and also withdraws consent for disclosure of future information (e.g. follow-up visits), no further evaluations are allowed to be performed and no additional data can be collected. The sponsor may retain and continue to use any data collected before such withdrawal of consent. Unresolved AEs in withdrawn patients have to be followed.

5.5.2 Handling of Withdrawals

In all cases, the reason for withdrawal must be recorded in the eCRF and in the patient's medical records. In case of withdrawal of a patient at his/ her own request, the reason should be asked for as extensively as possible and documented.

5.5.3 Premature Closure of the Clinical Trial or a Single Center

The trial can be prematurely closed or suspended by the Sponsor after consulting the Coordinating Investigator. The Ethics Committee (EC) and the Competent Regulatory Authorities must then be informed. Furthermore, the Ethics Committee(s) and Competent Regulatory Authorities themselves may decide to stop or suspend the trial.

Should the trial be closed prematurely, all trial material (completed, partially completed, and blank CRFs, investigational medicinal product and other material) must be returned to the Sponsor in Heidelberg or treated according Sponsor notice.

All involved investigators have to be informed immediately about a cessation/suspension of the trial. The decision is binding to all trial centers and investigators.

The Sponsor after consulting the Coordinating Investigator has the right to close a center, at any time, in case of:

- Non-compliance with the protocol
- Poor data quality
- No recruitment

5.6 Prior and Concomitant Illnesses

Relevant additional illnesses present at the time of informed consent are regarded as concomitant illnesses and are documented on the appropriate pages of the electronic case report form (eCRF). Included are conditions that are seasonal, cyclic, or intermittent (e.g. seasonal allergies; intermittent headache).

Abnormalities that appear for the first time or worsen (intensity, frequency) during the trial are adverse events (AEs) and must be documented on the appropriate pages of the eCRF.

5.7 Concomitant Therapy

While only Dexamethasone has shown mortality benefits in advanced COVID-19 in a non-peer reviewed study, there are currently no established therapies for COVID-19 early in the course of disease to prevent progression. Nonetheless, centers will follow guidelines and explore other therapies applied to COVID-19 patients. In this trial, each center upfront reports their standard therapy for the COVID-19 study population defined in the RECOVER trial. Standard arm patients receive the therapy outlined by the investigator of the participating center. CP/PVP arm patients receive CP/PVP therapy in addition to standard therapy. In case that the treatment algorithm of participating centers changes (likely during the course of the trial), these changes are reported. All treatments prescribed as antiviral or SARS-Cov-2 specific therapies are indicated with dosing and timing in the eCRF. Therapies which need to be noted in the eCRF include: all antiviral drugs, anti-IL6 therapies, Chloroquin, Hydroxychloroquin,

Azithromycin, steroids as well as other therapies aimed at altering immune response or viral replication. Patients may receive these drugs or combinations thereof during the entire trial.

There is no prohibited co-medication during the trial.

6 Investigational Medicinal Product (IMP)

The IMP consists of human plasma obtained by apheresis from patients/donors who have recovered from COVID-19 infection or received a SARS CoV-2 vaccination and have consented to donate. The IMP is composed of human convalescent/vaccine-boosted plasma and a stabilizer solution of sodium citrate. IMP Name: RECOVER CT SARS-CoV-2-ab Plasma COVID-19 (HD)

6.1 General Information about Convalescent/Vaccine-boosted Plasma

Covalescent/Vaccine-boosted plasma (CP/PVP) is generated by the IKTZ Heidelberg (Institut für Klinische Transfusionsmedizin und Zelltherapie) using the equipment and technologies already in operation at the IKTZ following standard operating procedures. A detailed description of the manufacturing process is provided in the IMPD. The drug product will be kept frozen during storage and thawed immediately prior to transfusion. During the course of the trial, further centers may produce CP/PVP if all preconditions and formal requirements are met.

Origin:	Plasma from apheresis obtained from donors, who have recovered from SARS-CoV-2 infection or received a successful vaccination against SARS-CoV-2
Container:	Plastic Bag with CE certificate
Route of administration:	Intravenous
Storage conditions:	Temperature: ≤ - 30°C in<br>Maximum storage period: 2 years including the day of manufacture
Manufacturer / Importer:	Institut für Klinische Transfusionsmedizin und Zelltherapie Heidelberg gGmbH, Im Neuenheimer Feld 305, 69120 Heidelberg (IKTZ Heidelberg)

The IKTZ Heidelberg as a blood donation[1]center provides convalescent/vaccine-boosted plasma bags directly to the respective and named addressee of the trial site, according to sponsor´s indication. The IKTZ Heidelberg ensures that donors are healthy at the time of donation and have not received prior treatment with hydroxychloroquine and/or 4-aminoquinolines within 6 months before donation. CP/PVP is packaged and labelled in accordance with the Good Manufacturing Practice guidelines and national and/or local regulatory requirements. CP/PVP has to be kept in a secure place in a blood bank / blood depot under appropriate storage conditions.

[1] Only donors without exposure to hydroxychloroquine and / or 4-aminoquinolines within the last 6 months prior plasma donation are eligible.

6.2 Therapeutic / Diagnostic Effects

Although the therapeutic effect of COVID-19 CP is not well known, previous reports suggest that neutralizing antibodies present in the sera may suppress viremia, when transferred to SARS-CoV-2-infected patients – leading to an improvement of symptoms, a shorter duration of hospitalization and reduced mortality compared to patients not treated with CP [11,19,23]. Furthermore, infusion of CP at an early stage of other coronavirus infections (H1N1 and SARS) has been associated with high effectiveness [9,19,25]. Thus, we anticipate that early CP/PVP treatment of high-risk COVID-19 patients results in a favourable outcome in a vulnerable patient population.

6.3 Known Side Effects of Convalescent Plasma Infusions[2]

According to previous reports, no significant side effects or serious adverse events have been specifically associated with CP infusions so far [21,26,27]. The most common side effects of CP infusion are similar to side effects of application of human plasma [23,28]:

- Volume overload with high infusion rates and high infusion volumes (Transfusion associated circulatory overload TACO) especially in patients with cardiac risk factors with the risk of cardiac failure and lung edema.
- Citrate intoxication with high infusion rates and high infusion volumes especially in liver failure, shock, acidosis, hypothermia and in newborns.
- Allergic reactions, in rare cases anaphylactic shock.
- Rare: Risk of transfusion transmitted infections: bacteria, viruses (HIV, HCV, HBV, CMV and others).
- Rare: transfusion associated lung injury (TRALI)
- There are theoretical risks that antibodies might enhance viral entry into cells or that antibodies might trigger. Accordingly, CP/PVP effects will be closely evaluated in this trial.

6.4 Dosage Schedule

Preparation of CP/PVP infusions must be conducted in a manner consistent with recommended safety standards for handling blood and its derivates.

CP/PVP infusion is administered on two days: visit day 1 (≤ 7 days after screening/baseline) and day 2. Each CP/PVP bag contains approx. 238 - 337 ml anti-SARS-Cov-2 CP/PVP for infusion. The CP/PVP can be administered after thawing of cryopreserved CP/PVP ≤ -30°C. CP/PVP is used ABO compatible. The CP/PVPs must be administered with an infusion rate of 80-100 ml/ hour within a four hour period for each CP/PVP bag. CP/PVP is infused over a standard transfusion device with a pore size of 170-230 µm.

Patients can receive plasma either from convalescents and/or from vaccinated persons.

Patients with persistence of SARS-CoV-2 virus load after day 28 may receive additional treatment with CP/PVP. These situations have to be discussed on a patient by patient basis with the principle investigator.

[2] This section of the study protocol is defined as reference safety information (RSI).

6.5 Treatment discontinuation

Definition of conditions leading to CP/PVP treatment discontinuation:

- **Adverse event with the transfusion of CP/PVP**: continuation of CP/PVP treatment after AE resolution to < grade 2 according to CTCAE
- **Severe allergic reactions or anaphylactic shock / serious adverse event upon CP/PVP transfusion:** transfusion must be stopped immediately and no further CP/PVP treatment is applied. A second CP/PVP administration is skipped.

6.6 Enrolment, Randomisation, Treatment Assignment

A subject is considered enrolled when he or she has signed the Informed Consent form. The patient receives a screening number at the clinical site (number of site plus number of patient in ascending order, e. g. 001-001 for the first enrolled patient at site 001) via registration in the eCRF system (www.xxx.). The screening number is used to identify the subject throughout the clinical study and must be used on all study documentation related to the subject.

Clinical sites must complete the baseline case report forms for all enrolled subjects, even if the subject is not randomised or treated in this study.

Upon confirmation of eligibility (patients must meet all inclusion criteria and must not meet exclusion criteria), the clinical site must contact a centralized internet randomization system (https://randomizer.at/). Patients are randomized using block randomisation to one of the two arms, experimental arm or standard arm, in a 1:1 ratio considering a stratification accoding to the 4 risk groups (as described in the inclusion criteria).

All subjects randomised receive a unique randomisation number. All patients in the experimental group have to start CP/PVP treatment within 7 days of randomization. Patients withdrawn from the trial retain their Patient ID and randomization number.

The study is open-label.

6.7 Packaging and Labeling

Labels are prepared in accordance with Good Manufacturing Practice (GMP) and local regulatory guidelines. The labels fulfill German GCP Ordinance (GCP-V § 5).

The following aspects are part of labeling (minimum requirements):

1) Name, Address and phone number of the sponsor
2) Name of IMP
3) AB0 and Rhesus blood group
4) Unique coding of blood bag with clear batch number ("Ch.-B.")
5) Dosage form and route of Administration
6) Volume and Ingredients
7) Storage conditions
8) Directions for use
9) Manufacturing and expiry date
10) Protocol number; EudraCT number
11) Recipient: Name, Surname, Date of Birth, subject identification number / ID, visit number / day, Treating Department, Treating Physician

Caution: Must be used only for this clinical trial. Please follow the instructions in the information leaflet.

6.8 Cross Over

Cross-over into the experimental arm is intended for patients randomized into the standard arm if the following criteria is met:

- Starting at day 10 (+ 2 days) after randomization
- No improvement or worsening of clinical condition

For cross-over, all inclusion / exclusion criteria (except mechanical ventilation/ECMO) must again be met as specified in Sections 5.3 and 5.4.

The actual possibility for cross-over into the experimental arm is based on discussion with the coordinating investigator on a case-by-case decision.

6.9 Supplies and Accountability

The local investigator keeps an account of the trial medication and acknowledge the receipt of all shipments of the IMP (Investigational Medicinal Product). All trial IMP must be kept in a blood bank / blood depot with access restricted to designated trial staff. The trial IMP must be stored dry and in accordance with manufacturer's instructions.

The investigator also keeps accurate records of the quantities of trial IMP dispensed and used. The documentation must include date of dispense, patient's sreening number, batch/serial numbers or other identification of trial IMP. The site monitor periodically checks the supplies and documentation of trial IMP to ensure the correct accountability of all trial IMP used. At the end of the trial, all unused trial IMP and all medication containers are returned or destroyed locally according sponsor´s instruction. IMP return and/or destruction must be documented appropriately. The Investigator is responsible for a continuous drug accountability throughout the study. A final drug accountability report must be filed in the Investigator's site file (ISF) at the end of the trial.

6.10 Compliance

Trial IMP is dispensed/administered to the patients by the investigator or another authorized study physician. Compliance is assessed by drug accountability of trial IMP*.* Details are recorded on the Drug Accountability Form.

7 Trial Methods

7.1 Description of Study Visits

All visits, time points and study assessments are summarized in the Trial Schedule (Table 1).

All participating trial sites will be supplied with study specific visit worksheets that list all assessments / procedures to be completed at each visit.

Patients will be informed about the study and the informed consent obtained before starting any study-related procedures.

The screening/baseline visit may be conducted on up to 7 days before randomisation. Once all screening/baseline assessments are completed, patients will be randomized to one of the two treatment arms. The first CP/PVP administration occurs at day 1.

The order of assessments is at the discretion of the investigator except for the study day 1: at day 1, all blood samples for SARS-Cov-2 diagnostic and biobanking must be collected before CP/PVP administration / transfusion. To lessen patient's burden, all screning/baseline assessments maybe completed on day 1. In this case, all screening/baseline assessments must be completed before randomisation and CP/PVP administered after completion of all other visit procedures.

General comments on clinical laboratory assessments

Laboratory safety assessments (hematology, coagulation and blood chemistry) are measured according to the Trial Schedule (Table 1) until hospital discharge.

Urinalysis by dipstick should be performed at screening/baseline and then only if clinically indicated. Microscopic analysis should be performed by the hospital's local laboratory if required.

Any clinically significant abnormal laboratory values should be repeated as clinically indicated and recorded into the eCRF.

Serum or urine pregnancy test is performed for pre-menopausal women of childbearing potential within 14 days prior to the start of study treatment and or prior to randomisation. Tests are performed by the hospital's local laboratory. If results are positive the patient is ineligible/must be discontinued from the study. In the event of a suspected pregnancy during the study, the test should be repeated.

Viral load in nose/throat swabs and antibody titers will be determined for the last time at the time of discharge within 84 days after randomization.

Serology tests for SARS-CoV-2 virus IgG antibodies and SARS-CoV-2 neutralizing antibodies will be performed by a central laboratory: Zentrum für Infektiologie, Virologie, Universitätsklinikum Heidelberg, Im Neuenheimer Feld 344, 69120 Heidelberg.

Instructions regarding sample collection, storage and shipment will be provided in the laboratory manual.

Table 2: Clinical Laboratory Measurements

Category	Lab Parameters
Hematology (local lab)	Haemoglobin, RBC, platelets, WBC with differential cell counts at baseline. If absolute differentials are not available, % differentials should be provided.
Blood chemistry and Coagulation standard (local lab)	BUN, creatinine, albumin, AST/SGOT, ALT/SGPT, total bilirubin, gGT, AP, LDH, lactate, sodium, potassium, magnesium, calcium, serum uric acid, LDH, Troponin, CK and CK-MB, Quick, PTT, ATIII, D-Dimer, INR, Fibrinogen, CRP,
Blood chemistry and Coagulation extended (local lab)	Ferritin, transferrin levels and transferrin saturation, total protein, albumin, IL6, procalcitonin, total IgG, IgA, IgM
Viral Screen (local lab)	HAV, HBV, HCV, and HIV-1
Urinalysis (local lab)	pH, glucose, proteins (qualitative, dipstick accepted)
Others	Serum/urine pregnancy test (local lab)<br>SARS-Cov-2 Viral clearance (positive/negative on standard PCR test and load (semiquantitative via CT value) (central lab, Prof. H.G. Kräusslich)<br>Anti-SARS-Cov-2 antibodies and neutralizing antibody titers (central lab, Prof. H.G. Kräusslich, Prof. Ralf Bartenschlager)<br>Blood samples for Biobanking (central lab, Biobank Med V, PD Dr. K. Kriegsmann)

CROSS-OVER OPTION into experimental arm:

Cross-Over starts with baseline visit and follows the primary schedule of the study as described in the trial schedule (Table 1).

7.2 Methods of Data and Sample Collection

7.2.1 Data Collection and Handling

All findings including clinical and laboratory data are documented by the investigator or an authorized member of the study team in the patient's medical record and in the electronic case report forms (eCRFs). The investigator at the clinical site is responsible for ensuring that all sections of the eCRFs are completed correctly and that entries can be verified against source data. The eCRFs have to be filled out according to the specified CRF Completion Guidelines. The correctness of entries in the eCRFs is confirmed by dated signature of the responsible local principal investigator or deputy principal investigator.

7.2.2 Sample Collection and Handling

For translational analysis samples are obtained at the times indicated in Table 1. Transport is done by TNT courier service or a similar operator providing the needed transport conditions. Further details are provided in the laboratory manual.

Shipment address for Translational research:

Universitätsklinikum Heidelberg, Zentrum für Infektiologie, Mikrobiologie 2. OG, Im Neuenheimer Feld 324, 69120 Heidelberg.

7.3 Measurement of Efficacy Parameters

The endpoints are analysed according to established criteria as suggested by the WHO.

7.3.1 Seven point ordinal scale

The seven point ordinal scale value is determined at the time of study inclusion and afterwards until discharge within 84 days after randomization or end of trial. This endpoint was also recommended by the WHO R&D Blueprint expert group (Coronavirus disease (COVID-2019) R&D. Geneva: World Health Organization (https://www.who.int/blueprint/priority-diseases/key-action/novel-coronavirus/en/). The seven-category ordinal scale [24] consists of the following categories: 1, not hospitalized with resumption of normal activities; 2, not hospitalized, but unable to resume normal activities; 3, hospitalized, not requiring supplemental oxygen; 4, hospitalized, requiring supplemental oxygen; 5, hospitalized, requiring nasal high-flow oxygen therapy, noninvasive mechanical ventilation, or both; 6, hospitalized, requiring ECMO, invasive mechanical ventilation, or both; and 7, death.

This scale is clearly defined and values will be obtained daily for each patient until day 28,thereafter weekly until day 56, and finally at day 84. A change in two points is clinically meaningful in either direction. For example, a patient with an improvement from #4 (oxygen supplementation, hospitalized) to #2 (discharged, no oxygen) is clearly relevant. Also, a change from #5 (high flow oxygen) to #3 (hospitalized but no supplemental oxygen) is clearly relevant as well. Of note, inclusion criteria of the trial determine that all patients to be included are either in #3, #4 or #5 at time of inclusion into the trial.

Physicians or nurses record the scale value daily at the same time. The time can be fixed individually for each patient. Recordings are obtained +/- one hour of the intended time.

7.3.2 Oxygen saturation (SaO_2) while breathing ambient air

Oxygen saturation while breathing ambient air will only be analysed for patients with an ordinal scale value below 5. Patients requiring supplemental oxygen are going to be analyzed for SaO_2 while breathing ambient air. If SaO_2 levels at ambient air drop below 90%, supplementary oxygen is immediately provided again and the amount of oxygen (liter/min) will be recorded that is required to keep SaO_2 above 94%. Patients at ordinal scales at 5 or higher will not be analysed this way.

7.3.3 Laboratory parameters

Nose/throat swabs are collected at day 1 (<u>before first CP/PVP administration</u>) and subsequently at day 3, 5, 10, 14, 28 or until discharge. Serum for SARS-Cov-2 diagnostic is collected at day 1, 35, 10, 14, 28, 56 and 84. Details on sample collection and schedule will be provided in the laboratory manual.

Nasopharyngeal swabs are analysed for SARS-CoV-2 RNA viral load by semi-quantitative real-time RT-PCR in the laboratory of Prof. H.G. Kräusslich (Center for Infectious diseases, University Hospital Heidelberg). Patient serum obtained at the different time points is stored at -20° C until further use. Antibodies directed against SARS-CoV-2 are going to be analysed in recipient serum before (day1) and after (day 3) treatment. IgG Antibodies are detected in standard ELISA tests. Further, analyses for neutralizing antibodies are performed by Prof. Dr.

Ralf Bartenschlager (DKFZ, Heidelberg). Cytokine analyses is performed in the laboratory of PD Dr. Niels Halama (NCT Heidelberg) with the Bio-Plex Pro Human Cytokine 48-Plex Screening Panel (Biorad, Munich, Germany). To evaluate the effects of CP/PVP onto inflammation and cytokine levels, a first analysis is performed after the first randomized 40 patients. All other serum specimens are analyzed at the end of trial.

7.4 Measurement of Further Parameters

Vital signs

Height, weight, temperature and blood pressure are collected. Height (in cm) is measured at baseline only. Weight (in kg), temperature (in grade centigrade), and blood pressure/pulse are measured at baseline. Oxygen saturation is regularly assessed by pulse oximetry or invasive analysis if applicable.

Electrocardiogram (ECG)

A 12-lead ECG is performed at baseline as well as additionally at the investigator’s discretion.

Twelve-lead ECGs is obtained after the patient has been rested in a supine position for at least 5 minutes in each case. The Investigator or designated physician reviews the paper copies of each of the timed 12-lead ECGs on each of the study days when they are collected.

8 Ancillary and Post Trial Care

Study ends for individual patients on day 84 (experimental arm and standard arm without cross over), day 94 (standard arm in case of cross over), or death. All discharged patients are followed by regular phone calls (scheduled days: 3, 7, 10, 14, 28, 56, 84).

9 Assessment of Safety

9.1 Specification of Safety Parameters

9.1.1 Adverse Events

According to GCP, an adverse event (AE) is defined as follows: Any untoward medical occurrence in a patient administered a pharmaceutical product and which does not necessarily have a causal relationship with this treatment. An AE can therefore be any unfavorable and unintended sign (including an abnormal laboratory finding), symptom, or disease temporally associated with the use of a medicinal (investigational) product, whether or not related to the medicinal (investigational) product.

An AE may be:

- New symptoms/ medical conditions
- New diagnosis
- Changes of laboratory parameters
- Intercurrent diseases and accidents
- Worsening of medical conditions/ diseases existing before inclusion into the trial
- Recurrence of disease
- Increase of frequency or intensity of episodical diseases.

Each AE must be reported only once, indicating the worst CTC (Version 5.0) grade. If an event stops and later restarts, all occurrences must be reported.

A pre-existing disease or symptom is not considered an adverse event unless there is an untoward change in its intensity, frequency or quality. This change is documented by an investigator.

Surgical procedures themselves are not AEs; they are therapeutic measures for conditions that require surgery. The condition for which the surgery is required may be an AE. Planned surgical measures permitted by the clinical trial protocol and the condition(s) leading to these measures are not AEs, if the condition leading to the measure was present prior to inclusion into the trial.

AEs are classified as "non-serious" or "serious".

9.1.2 Serious Adverse Event

A serious adverse event (SAE) is one that at any dose:

- Results in death
- Is life-threatening (the term life-threatening refers to an event in which the patient was at risk of death at the time of event and not to an event which hypothetically might have cause death if it was more severe)
- Requires patient hospitalization or prolongation of existing hospitalization
- Results in persistent or significant disability/ incapacity
- Results in a congenital anomaly/ birth defect.

Is medically significant (e.g. suspected transmission of an infectious agent via medicinal product). Moreover there are other situations - such as important medical events that may not be immediately life threatening or result in death or hospitalisation but may jeopardize the patient or may require intervention to prevent one of the other outcomes listed above.

9.1.3 Expectedness

An ‘unexpected’ adverse event is one the nature or severity of which is not consistent with the expected side-effects of Convalescent/Vaccine-boosted Plasma Infusions mentioned in 6.3. Furthermore, reports which add significant information on specificity or severity of a known adverse reaction are counted as ‘unexpected’ events.

9.1.4 Suspected Unexpected Serious Adverse Reaction (SUSAR)

SAEs that are both suspected, i.e. possibly related to IMP, and ‘unexpected’, i.e. the nature and/or severity of which is not consistent with the applicable product information are to be classified as Suspected Unexpected Serious Adverse Reactions (SUSARs).

In case if either the investigator, who primarily reported the SAE, or the second assessor classify the SAE as ‘suspected’ (“reasonable possibility” or “unknown”, see below) and the SAE is also unexpected, it is categorized as a SUSAR.

All SUSARs are subject to an expedited reporting to the responsible ethics committee(s), the competent authority PEI and to all participating investigators.

9.2 Period of Surveillance and Documentation

All AEs reported by the patient or detected by the investigator are collected during the trial. AEs must also be documented in the patient’s medical records. Whenever possible, the investigator records the main diagnosis instead of the signs and symptoms normally included in the diagnoses.

In this trial, **all AEs that occur after signature of the informed consent** are documented on the pages provided in the CRF. AEs must be followed up to day 28 or until all drug-related toxicities have been resolved, whichever is later, or until the investigator assesses AEs as “chronic” or “stable”. Each AE must be reported, indicating the worst CTC (Version 5.0) grade. If an event stops and later restarts, all occurrences must be reported. A specific procedure for definition and reporting of SAEs is described in Section 9.3.

9.2.1 Grading of AEs

The grading of AEs in this trial is carried out on the basis of the 5-grade scale defined in the CTCAE v5.0.

**Grade 1:**	Mild; asymptomatic or mild symptoms; clinical or diagnostic observations only; intervention not indicated.
**Grade 2:**	Moderate; minimal, local or noninvasive intervention indicated; limiting age-appropriate instrumental ADL*
**Grade 3:**	Severe or medically significant but not immediately life-threatening; hospitalization or prolongation of hospitalization indicated; disabling; limiting self-care ADL**.
**Grade 4:**	Life-threatening consequences; urgent intervention indicated.
**Grade 5:**	Death related to AE

A Semi-colon indicates ‘or’ within the description of the grade.
Activities of Daily Living (ADL)
*Instrumental ADL refer to preparing meals, shopping for groceries or clothes, using the telephone, managing money, etc.
**Self-care ADL refer to bathing, dressing and undressing, feeding self, using the toilet, taking medications, and not bedridden.

The grading of all AEs listed in the CTCAE v5.0 is based on the information contained therein. The grading of all other AEs, i.e. those which are not listed in the CTCAE v5.0 is performed by a responsible investigator.

9.2.2 Coherence between AEs and the IMP

The Investigator must provide an assessment of causal relationship of each of the clinical trial IMPs to each AE according to the following scale:

**Y** (Yes)	There is a reasonable possibility that the IMP/s caused the AE.
**N** (No)	There is no reasonable possibility that the IMP/s caused the AE and other causes are more probable.
**Uk** (Unknown)	Only to be used in special situations where the Investigator has insufficient information (i.e., the patient was not seen at his/her centre) if none of the above can be used.

9.2.3 Outcome of AEs

The outcome of an AE at the time of the last surveillance is classified as:

**Recovered/ resolved**	All signs and symptoms of an AE disappeared without any sequels at the time of the last interrogation.
**Recovering/ resolving**	The intensity of signs and symptoms has been diminishing and/ or their clinical pattern has been changing up to the time of the last interrogation in a way typical for its resolution.
**Not recovered/ not resolved**	Signs and symptoms of an AE are mostly unchanged at the time of the last interrogation.
**Recovered/ resolved with sequel**	Actual signs and symptoms of an AE disappeared but there are sequels related to the AE.
**Fatal**	Resulting in death. If there are more than one AE only the adverse event leading to death (possibly related) is characterized as ‘fatal‘.
**Unknown**	The outcome is unknown or implausible and the information cannot be supplemented or verified.

9.2.4 Action taken

The action taken is assigned to one of the following categories:

**Dose not changed**	No change in the dose

**Dose reduced**	Reduction in the dose
**Temporary discontinuation**	Temporary discontinuation of treatment
**Drug withdrawn**	Discontinuation of treatment
**Unknown**	The information is unknown or implausible and it cannot be supplemented or verified.
**Not applicable**	The question is implausible (e.g. the patient is dead).

9.2.5 Countermeasures

The term 'Countermeasures' refers to the specific actions taken to treat or alleviate adverse events or to avoid their sequels. The following categories are used to categorize the countermeasures to adverse events:

**None**	No action taken
**Drug treatment**	Newly-prescribed medication or change in dose of a medication
**Others**	Other countermeasures, e.g. an operative procedure

9.3 Reporting of Serious Adverse Events by Investigator

All SAEs must be reported by the investigator to the responsible Safety Officer at the KKS Heidelberg (on behalf of sponsor) within 24 hours after the SAE becomes known using the "Serious Adverse Event" form. The initial report must be as complete as possible, including details of the current illness and (serious) adverse event as well as an assessment of the causal relationship between the event and the trial medication. The reporting is performed by faxing of a completed 'SAE Form' to the KKS Heidelberg.

Fax-number: + 49 (0)6221 56 33687

9.4 Expedited Reporting

SUSARs are to be reported to the ethics committee(s), regulatory authorities and to all participating investigators within regulative defined timelines, i.e. they are subject to an expedited reporting.

All SAEs are forwarded by e-mail immediately (not later than 24 hours after receipt) by the responsible person at KKS Heidelberg to the coordinating investigator or the scientific coordinators in order to perform a second assessment. The coordinating investigator or the scientific coordinators fill out a 'Second Assessment Form' for each SAE and return it by e-mail to the KKS Heidelberg within 48 hours.

E-mail: V-KKS.SAE@med.uni-heidelberg.de

The 'Second Assessment Form' contains the following information:

I) Assessment of relationship between SAE and IMP

II) Assessment of expectedness of SAE (derived from IB or SPC)

III) Assessment of relationship between SAE and the underlying disease

IV) Statement if the benefit/ risk assessment for the trial did change as a result of SAE.

The expedited reporting is carried out by KKS Heidelberg.

9.7 Emergency Treatment

During and following a patient's participation in the trial, the investigator should ensure that adequate medical care is provided to a patient for any AE, including clinically significant laboratory values. The investigator should inform a patient when medical care is needed for intercurrent illness(es) of which the investigator becomes aware.

9.7 Events of special interest

In cases of **transfusion reactions** after plasma transfusion e.g. anaphylaxis or hemolysis or transfusion related lung injury (TRALI), the safety desk of the KKS should be informed immediately by faxing of a completed SAE form (see above).

9.8 Deaths

All deaths that occur during the study, or before EOS, must be reported as follows:

- Death should be documented in the eCRF and should be reported as an SAE.
- Where death is not due (or not clearly due) to SARS-CoV-2, the AE causing the death must be reported to the Safety Officer at the KKS Heidelberg within 24 hours after the death becomes known using the "Serious Adverse Event" form. (9.1.2 Serious Adverse Event). The report should contain a comment regarding the co-involvement of SARS-Cov-2 disease, if appropriate, and should assign main and contributory causes of death

Deaths with an unknown cause should always be reported as a SAE.

10 Statistical Considerations

10.1 Study Design

This is a randomized open-label, multicenter phase-II study in patients with severe SARS-CoV-2 disease subjects

Randomisation (rf. also to chapter 6.8):

Randomisation is done either into experimental arm E or into standard arm S in a 1:1 ratio using block randomisation stratifed for the factor "patient group", defined by group 1 (patients with hematological malignancies, history of stem cell transplantation and/or active cancer therapy – incl. chemotherapy, radiotherapy, surgery – within the last 24 months or less.), group 2 (drug induced immunosuppression not meeting the criteria of group 1), group 3 (age ≥ 50 - 75 years and meeting neither the criteria of group 1 nor group 2 and Lymphopenia < 0.8 x G/l and/or D-dimer > 1µg/mL and group 4 (age > 75 years and meeting neither the criteria of group 1 nor group 2).

10.2 Analysis Variables

10.2.1 Primary endpoint and primary estimand

The main purpose of the study is to assess the time from randomization until an improvement within 84 days defined as two points on a seven point ordinal scale or live discharge from the hospital in high-risk patients (group 1 to group 4) with SARS-CoV-2 infection requiring hospital admission by infusion of plasma from subjects after convalescence of a SARS-CoV-2 infection or after vaccination or standard of care.

In the Addendum to the ICH E9 guideline (final version), the estimands framework is recommended as clear and transparent definition of "what is to be estimated" (International Council for Harmonization 2019). An estimand is defined through the treatment, the targeted population, the variable, a specification of how to handle intercurrent events (postrandomization events) and a population-level summary. In the following, the primary estimand corresponding to the primary objective is described.

The primary estimand corresponding the primary objective is defined as follows:

Treatment: Infusion of frozen CP/PVP (on two days of intervention from two different donors) vs. standard of care (as described in 5.7).

Population: The targeted population is defined through the in- and exclusion criteria.

Variable: Time from randomisation to clinical improvement within 84 days by two points on a seven point ordinal scale or live discharge from the hospital.

Post-randomisation events: live discharge from the hospital is incorporated into the variable definition (composite strategy), death from any cause within 84 days after randomization without previous improvement is taken into account by censoring deceased patients at day 84 (see sections 10.3 and 10.5.4 for rationale)); treatment switch will be ignored (treatment policy strategy, see sections 10.3 and 10.5.4 for rationale); event-free patients at the end of the follow-up period are censored and drop-outs are censored at the last observation (hypothetical strategy).

Summary measure: Hypothesis testing is conducted using a log-rank test. The summary measure for effect quantification is the hazard ratio for the endpoint "clinical improvement by two points or live discharge" between the two treatment arms (which is estimated using Cox regression).

10.3.2 Secondary Endpoints

Overall survival, defined as the time from randomization until death from any cause

28-day, 56-day and 84-day overall survival rates

SARS-CoV-2 viral clearance and load as well as antibody titres.

Requirement mechanical ventilation at any time during hospital stay (yes/no)

Time until discharge from randomization.

Viral load, changes in antibody titers and cytokine profiles are analysed in an exploratory manner using paired non-parametric tests (before – after treatment).

10.2.3 Safety Endpoints

This includes all AEs, their severity, SAEs, the relation of AEs to the study treatment, dose modifications for toxicity and discontinuation of study treatment during the trial phase. Toxic effects are graded according to the National Cancer Institute Common Toxicity Criteria (CTCAE) version 5.0.

10.3 Sample Size Calculation

Based on recently published data [24] median time to an improvement defined as two points on a seven point ordinal scale or live discharge from the hospital is assumed to be at day 16 from randomization. This median estimate considers patients who died without an improvement as censored at day 84 (right-censoring), which ensures that those patients are considered as "not improved" over the whole observation period. In our trial, we follow the same analysis strategy as in Cao *et al.*, 2020 who also censored deceased patients at the end of follow-up [24].

We assume that based on the mechanism of action (neutralizing antibodies) a rapid improvement is induced by fresh frozen plasma from subjects after convalescence of SARS-CoV-2 infection or vaccination on two days from two different donors with a median time of 10 days (HR=1.6). Based on first results obtained in vaccinated subjects, we allow by amendment apart from convalescent plasma also the use of plasma obtained from vaccinated subjects, in which high amounts of neutralizing antibodies are present. We assume that plasma from vaccinated subjects are at least as effective as convalescent plasma[12-14]. Assuming exponentially distributed event times, these assumptions correspond to a hazard of $\lambda_C = 0.043$ for the control arm, while the corresponding hazard in the experimental arm should amount to $\lambda_E = 0.043 \cdot 1.6 = 0.69$.

While it is allowed for patients from the control arm to switch to the experimental arm starting at day 10 (+ 2 days) (see section 6.8), we assume this will not have a major effect on the median time of clinical improvement for these patients, since we expect that plasma infusions will have a larger advantage if given early. While methods for handling patients switching from control to treatment group are available, they are subject to strong and unverifiable assumptions [29]. Hence, we will ignore treatment switch in the primary analysis, representing a treatment policy strategy (see Section 10.2.1), which can be regarded as a conservative approach since switching is only allowed from the control to the experimental arm. However, in supplementary analyses of the primary endpoint, we will consider such a hypothetical scenario in which a treatment effect is estimated for the case that patients would not have switched by means of inverse probability censoring weighting according to Robins & Finkelstein [30], relying on the assumption that there are no unmeasured confounders influencing the probability to switch, and a rank preserving structural failure time (RPSFT) model [31], assuming a common treatment effect which remains constant over time, regardless when treatment is initiated.

Since patients might die before experiencing a clinical improvement or being discharged from the hospital, death will be taken into account via censoring deceased patients at last day of follow-up (day 84). Based on recently published data [24], the mortality rate is assumed to be about 20% in the control arm. Thus, we expect that the cumulative improvement rate of the control group will not become larger than 0.8. Hence, fewer improvement events are expected during the follow-up of 84 days than under the assumption of an exponential distribution, since the improvement curves will flatten at some point. In case of the control group, the flattening is expected to occur at day 38, since the assumed cumulative improvement rate at day 38 amounts to $\exp(-\lambda_C \cdot 38) = \exp(-0.043 \cdot 38) \approx 0.8$. Accordingly, we conservatively assume a follow-up length of 38 days for the sample size calculation to properly estimate the probability of experiencing the event of interest, even though the actual follow-up for each patient will be longer.

To detect the assumed difference between treatment groups using a log-rank test comparing the cumulative improvement curves for the primary endpoint using a significance level of 5% (two-sided) with a power of 80%, a total number of 174 patients is required for the entire trial (87 patients per group) when additionally considering a dropout rate of 5%, meaning that n=164 patients who do not prematurely drop out of the study are required to be enrolled. The required number of events which was calculated using the formula by Schoenfeld, amounts to 142. It is expected that adjusting for the covariate "patient group" in the analysis will lead to an increase in power. Sample size calculation was done conservatively assuming an equally long follow-up period of 38 days for every patient. Sample size calculation was done using RPACT v 2.0.6.

For the sample size calculation, it was assumed that there would be a consistent median time to improvement and overall survival rate across all 4 patient groups. Since this might not necessarily be the case, and it could be likely that patients in group 1 and 2 might have a shorter median time to improvement, we conducted a simulation study to explore the robustness of our model under varying assumptions for the median event times. For our simulation study, we assumed exponentially distributed event times, with a median time to improvement of 16 days across all patient groups in the control group ($med_C^1 = med_C^2 = med_C^3 = med_C^4 = 16$), while we assumed differing median times to improvement in the patient groups 1 & 2 compared to patient groups 3 & 4 ($med_E^1 = med_E^2$, $med_E^3 = med_E^4$) in the experimental group. We considered a sample size of n=164 evaluable patients for the whole trial, with 42 patients belonging to groups 1 and 3 each, and 40 patients belonging to groups 2 and 4 each corresponding to patient group membership probability of about 25% for all four groups. Also, we assumed a 38-day mortality rate of 20% in the control arm, while we assumed that the corresponding mortality rate in the experimental arm amounted to 13% (corresponding to a HR for overall survival of 1.6) across all four patient strata assuming exponentially distributed

survival times. We simulated 10,000 trials per scenario corresponding to a maximum standard error of sqrt(0.5*0.5/10000)=0.005 for the simulated power.

We used a stratified log-rank test to assess whether the cumulative improvement curves in the two treatment groups (experimental/control) would be equal at a two-sided significance level of α=0.05 stratifying for the factor "patient group" for analysis. The analysis of the simulated datasets was done using the PROC LIFETEST procedure in SAS v9.4. The results are shown in Table 3.

Table 3: Power for the RECOVER trial considering varying treatment effects across patient groups, assuming medC1= medC2 = medC3 = medC4=16 days

Median time to improvement in intervention group for patient groups 1 and 2 in days	Median time to improvement in intervention group for patient groups 1 and 2 in days	Simulated Power 1-β
med_E^1= med_E^2=10	med_E^1= med_E^2=10	0.785
med_E^1= med_E^2=9	med_E^1= med_E^2=11	0.794
med_E^1= med_E^2=8	med_E^1= med_E^2=12	0.813

Assumptions: α=0.05 (two-sided), 10,000 simulations per scenario, n=164 patients not prematurely dropping out of the trial enrolled.

The results in Table 3 show that the aspired power of 80% is approximately achieved in all scenarios, with an even increased power in case of a treatment effect differing between patient groups in the experimental group. This indicates that our planned analysis strategy will achieve the aspired power even under varying treatment effects across the patient strata, when the "average" median time to improvement in the experimental group still amounts to 10 days.

10.4 Analysis Populations

10.4.1 Full Analysis Population

The Full Analysis Population (FAP) includes all randomised patients with treatment groups assigned in accordance with the randomisation, regardless of the treatment actually received. Patients who were randomised but did not subsequently receive treatment are included in the Full analysis population. The analysis of data using the Full analysis population therefore follows the Intention-to-Treat principles (ITT).

10.4.2 Per Protocol Population

In the per protocol population, patients with important protocol deviations are excluded. Definition of important protocol deviations are given in the statistical analysis plan (SAP).

10.4.3 Safety Population

All enrolled patients who received treatment are subjected to the safety analysis. Patients will be evaluated with regard to the treatment actually received, meaning that patients who cross over from the control to the experimental arm will be evaluated in the experimental arm at day 10 (+ 2 days) onwards. Details of the safety analysis are specified in the statistical analysis plan (SAP).

10.5 Statistical Methods

10.5.1 General Considerations

The statistical evaluation is carried out under the supervision of the Supervising Statistician. Statistical analysis is based on the International Conference on Harmonization (ICH) Guidelines “Structure and Content of Clinical Study Reports” and “Statistical Principles for Clinical Trials”. All statistical procedures are done according to the current Standard Operating Procedures (SOPs) of the Institute of Medical Biometry and Informatics, University of Heidelberg (IMBI).

All data recorded in the eCRF describing the sample, the efficacy and the safety are analyzed descriptively. Categorical data are presented in contingency tables with frequencies and percentages. Continuous data are summarized with at least the following: missing values, median, quartiles, mean, standard deviation, minimum and maximum.

The detailed methodology for the statistical analysis is described in the statistical analysis plan (SAP), which is finalized before database lock. Statistical analysis is performed using SAS v9.4 or higher.

10.5.2 Demographic and other Baseline Characteristics

Patients’ disposition and reasons for ending the study are presented in frequency distribution tables and individual data listing. Descriptive statistics of the baseline characteristics are generated across all treated patients. Frequency distributions are presented for the categorical variables. Summary statistics and the number of assessed patients are calculated, as appropriate, for the quantitative variables. Individual data is presented in listings.

Categorical baseline characteristics, like sex, concomitant illness at trial initiation, and concomitant treatment maintained, is summarized by frequency tables. Summary statistics such as mean, standard deviation, median, first and third, quartile, as well as minimum and maximum, are provided for quantitative variables like age, weight, laboratory values.

10.5.3 Study Therapy, Treatment Compliance, and Follow-up

Summary statistics are presented for the amount and dosage of CP/PVP received, dose modifications for toxicity, discontinuation and withdrawal from study treatment, as well as drop-out from the follow-up.

10.5.4 Analysis of the Primary Endpoint

The primary analysis will assess the null hypothesis “the cumulative improvement curves for the primary endpoint in the experimental and control arm are equal”, i.e. H_0: $S_E = S_C$ against the alternative hypothesis H_1: $S_E \neq S_C$ at a two-sided significance level of α=5%. This will be achieved by using the log-rank test stratified for the factor “patient group” as used in the randomisation procedure. The event “death from any cause” will be handled by censoring those patients at day 84 (in analogy to the approach of Cao et al, NEJM 2020). Using this approach ensures that deceased patients are considered as “not improved” over the whole observation period of 84 days. The hazard ratio for treatment group will be determined using a Cox regression model stratifiying for the factor “patient group” together with a 95% confidence interval.. The cumulative improvement curves are calculated using the Kaplan-Meier method together with 95% log-log-type confidence bands, and will be calculated separately for both treatment groups. In addition, cumulative improvement curves will also be calculated separately for each patient group per treatment group.

The fact that some patients from the control group might switch to the experimental group at day 10 (+ 2 days) will be ignored in the primary analysis, representing a treatment policy approach according to the ICH E9 (R1) addendum on estimands [32]. Patients who drop out during the trial or are lost to follow-up are taken into account as censored observations. The primary analysis will be based on the FAS according to the ITT principle.

A supplementary analysis of the primary endpoint will involve the inverse probability censoring weighting (IPCW) approach to estimate a treatment effect in the hypothetical scenario that patients from the control group had not switched to the experimental group [30]. This type of model requires to estimate the probability for a patient to switch to the experimental group based on (time-dependent) covariates, which in our case will be the baseline factor "patient group", together with the time-varying factors "seven point ordinal scale measuring clinical improvement" and "viral load" over time. It should be noted that this model implicitly assumes that all relevant confounders predicting whether a patient switches from control to treatment have been included into the model, an assumption which is hardly verifiable, especially for such a recently emerged disease about which we currently only have limited understanding, thus being the reason why we did not choose this as the strategy for the primary analysis.

As another supplementary analysis intending to estimate the treatment effect in the hypothetical scenario which assumes that control patients had not switched to the treatment group, a rank preserving structural failure time (RPSFT) model will be fitted [31]. This model assumes a common treatment effect, meaning that the treatment effect is the same for all individuals (with respect to time spent on treatment) regardless of when treatment is received. Since this is another assumption which is hardly verifiable, and because we especially assume that patients will have a larger treatment effect when plasma is given early, we consider this approach as a supplementary analysis only.

Further sensitivity analyses of the primary endpoint include an analysis based on the per protocol population.

10.5.5 Analysis of the Secondary Endpoints

Overall survival is analyzed by using a Cox regression model adjusting for treatment and patient group, determining hazard ratios with 95% confidence intervals and (descriptive) p-values. Survival estimates are calculated using the Kaplan-Meier method together with 95% log-log-type confidence bands, and the 28-day survival rate will be given for both treatment groups together with 95% confidence intervals. Analogously as for the supplementary analysis of the primary endpoint, an IPCW model and a RPSFT model will additionally be fitted for the secondary endpoint overall survival to assess the potential treatment effect in case patients had not switched from the control to the experimental arm.

Time until discharge will be assessed similarly to the primary endpoint, providing Kaplan-Meier estimates for the cumulative discharge rate for both treatment groups and conducting a log-rank test stratified for the factor "patient group", and censoring patients who died from any cause at day 84 in analogy to the analysis of the primary endpoint. Analogously as for the sensitivity analysis of the primary endpoint, an IPCW and a RPSFT model will additionally be fitted for the secondary endpoint 'time until discharge' to assess the potential treatment effect in case patients had not switched from the control to the experimental arm.

The secondary endpoint "requirement of mechanical ventilation (yes/no)" is analyzed by means of a logistic regression model adjusting for the factors treatment and patient group. Also, absolute and relative frequencies will be given for this endpoint, together with 95% confidence intervals.

The secondary endpoints SARS-CoV-2 viral load and antibody titres will be assessed over time by means of linear mixed models adjusting for treatment group, patient group, and baseline value as fixed factors, as well as time as repeated factor using an unstructured covariance matrix, calculating least square means estimates and 95% confidence intervals.

While it is indirectly assumed by these models that data will be missing at random, i.e. that the missingness can be explained by observed covariate and outcome values, we will assess the

robustness of this assumption by means of sensitivity analyses by means of pattern mixture models assuming that data will be not missing at random.

For all secondary endpoints, the analysis will be conducted based on the FAS and as a sensitivity analysis based on the per protocol population.

Safety Analysis

The assessment of safety is based mainly on the frequency of adverse events and on the number of laboratory values that fall outside of pre-determined ranges and/or show prominent worsening from baseline during the study phase. Safety analysis is based on the safety population. Adverse events are summarized by presenting the number and percentage of patients having any adverse events or serious adverse events, and having each individual adverse event, and by determining and summarizing the maximum individual toxicity grade (over all forms of toxicity). Furthermore, the most common AEs (those occurring in at least 10% of the treatment group) are determined. Any other information collected (e.g. severity or relatedness to study drug) are summarized as appropriate. Laboratory data are summarized by presenting summary statistics of raw data and change from baseline values. Incidence rates are summarized along with two-sided Clopper-Pearson 95% confidence intervals and analyzed by (descriptive) chi-squared tests.

Other Analyses

- Patients' disposition and reasons for ending the study are presented in frequency distribution tables and individual data listing.

Individual data are presented in listings.

Further, the impact of type of plasma (convalescent, vaccine-boosted) is assessed on an explorative basis using univariable analysis as well as multivariable models for the primary and secondary endpoints.

10.5.6 Handling of Missing Data

For patients with incomplete follow-up, time to last follow-up date is used as the censoring time in the analysis of time-to-event data. Missing data of continuous outcomes over time will be handled via the multi-level approach making the implicit assumption that data are missing at random, thus not requiring any direct imputation of missing continuous data. The robustness of this assumption will be explored in sensitivity analyses by means of pattern mixture models assuming that data are not missing at random. Otherwise, no imputation of missing data will be conducted.

11 Data Management

11.1 Data Collection

As used in this protocol, the term Case Report Form (CRF) should be understood to refer either to a paper form or an electronic data record or both, depending on the data collection method used in this trial.

The data collection will be performed using an eCRF in accordance with the German Corona Consensus Data Set (GECCO).

Data collection using the eCRF can only be done by authorized persons. All study data are password-protected. The eCRF provides several checks for completeness and consistency. Each entry or change of data is tracked with name and exact date. When data has been entered, reviewed, edited and Source Data Verification (SDV) performed, the investigator is notified to sign the CRF electronically as per agreed project process. A copy of the electronic-CRF will be archived at the study site.

All findings including clinical and laboratory data are documented by the investigator or an authorized member of the study team in the patient's medical record and in the case report form (CRF). The investigator is responsible for ensuring that all sections of the CRF are completed correctly and that entries can be verified by source data. The CRF has to be filled out according to the specified CRF Completion Guidelines.

11.2 Data Handling

Data entries undergo an automatic online check for plausibility and consistency. In case of implausibility, 'warnings' are produced. A responsible investigator is obliged either to correct the implausible data or to confirm its authenticity, and to give appropriate explanation. If not corrected, the data is flagged, enabling a convenient check of all questionable entries. A responsible monitor checks all flagged data and generates questions that are sent back to the responsible investigator. The investigator has to resolve all 'discrepancies'.

Further checks for plausibility, consistency, and completeness of data are performed after completion of the study. Queries are generated on the basis of these checks, combined with a visual control by a responsible monitor/data manager.

All missing data or inconsistencies are reported back to the sites and clarified by the responsible investigator. If no further corrections are to be made in the trial database it is declared closed and used for statistical analysis.

All data management activities are done according to the current Standard Operating Procedures (SOPs) of the IMBI.

11.3 Storage and Archiving of Data

According to §13 of the German GCP-Regulation all important trial documents (e.g. CRFs) will be archived for at least 10 years after the trial termination. Responsible for archiving TMF including protocol, CRFs and reports is the CI.

The investigator(s) will archive all trial data (source data and Investigator Site File (ISF) including Patient Identification List and relevant correspondence) according to the section 4.9 of the ICH Consolidated Guideline on GCP (E6) and to local law or regulations. The Patient Identification List will be archived for at least 15 years after trial termination.

If the investigator relocates, retires, or for any reason withdraws from the study, the Sponsor should be prospectively notified. The study records must be transferred to an acceptable designee, such as another investigator, another institution, or to the Sponsor. The investigator must obtain Sponsor´s written permission before disposing of any records, even if archiving requirements have been met.

12 Ethical and Legal Aspects

12.1 Good Clinical Practice

The procedures set out in this trial protocol, pertaining to the conduct, evaluation, and documentation of this trial, are designed to ensure that all persons involved in the trial abide by Good Clinical Practice (ICH-GCP E6 (R2)) and the ethical principles described in the current version of the Declaration of Helsinki. The trial is carried out in keeping with local legal and regulatory requirements.

12.2 Patient Information and Informed Consent

Before being admitted to the clinical trial, the patient must consent in written form to participate after the nature, scope, and possible consequences of the clinical trial have been explained in a form understandable to him or her. The original personally signed and dated Informed

Consent Form must be kept on file by the investigator(s), and documented in the case report form.

A copy of the signed informed consent document must be given to the patient. The documents must be in a language easily understandable to the patient and must clearly state who informed the patient, which is confirmed by the dated signature of the responsible investigator.

If new safety information results in significant changes in the risk/benefit assessment, or if changes are made in the protocol, the consent form should be reviewed and updated if necessary. All patients (including those already being treated) should be informed of the new information and must give their written informed consent to continue the study.

12.3 Confidentiality

The data obtained in the course of the trial is treated pursuant to the applicable Data Protection Law (EU General Data Protection Regulation – GDPR - 2016/679), the Federal Data Protection Act (Bundesdatenschutzgesetz, BDSG), the State Data Protection Act of Baden-Württemberg (Landesdatenschutzgesetz, LDSG BW) as well as § 40 (2a) AMG.

During the clinical trial, patients are identified solely by means of an individual identification code (Patient ID). Storage of trial findings on a computer are done in accordance with local data protection law and are handled in strictest confidence. For protection of these data, organizational procedures are implemented to prevent distribution of data to unauthorized persons. The appropriate regulations of local data legislation are fulfilled in its entirety.

The patient consents in writing to relieve the investigator from his/her professional discretion in so far as to allow inspection of original data for monitoring purposes by health authorities and authorized persons (inspectors, monitors, auditors). Authorized persons (clinical monitors, auditors, inspectors) may inspect the patient-related data collected during the trial, ensuring the data protection law.

The investigator maintains a patient identification list (Patient IDs with the corresponding patient names) to enable records to be identified.

Patients who did not consent to circulate their pseudonymized data are not included into the trial.

12.4 Responsibilities of Investigator

The Principal Investigator should ensure that all persons assisting with the trial are adequately informed about the protocol and any amendments, the trial treatments, and their trial-related duties and functions.

The Principal Investigator should maintain a list of investigators and other appropriately qualified persons to whom he or she has delegated significant trial-related duties (Log of Staff).

The investigator(s) should support monitoring, auditing and inspections as described in sections 14.1 and 14.2.

12.5 Approval of Trial Protocol and Amendments

Before the start of the trial, the trial protocol, informed consent document, and any other appropriate documents are submitted to the independent Ethics Committee (EC) as well as to the competent authority. A written favorable vote of the EC and an (implicit) approval by the competent authority are a prerequisite for initiation of the clinical trial. The statement of EC should contain the title of the trial, the trial code, the trial site, and a list of reviewed documents. It must mention the date on which the decision was made and must be officially signed by a committee member.

Before the first patient is enrolled in the trial, all ethical and legal requirements must be met. All planned substantial changes (see §10, (1) of German GCP-Ordinance) are submitted to EC and the competent authority in writing as amendments. They have to be approved by the EC and the competent authority.

The Coordinating Investigator or the NCT Trial Center, and if applicable the investigator(s) keep a record of all communication with the EC and the regulatory authorities.

12.6 Continuous Information to Independent Ethics Committee

Pursuant to the German Drug Law (AMG) and the GCP Ordinance, the EC and the competent authority are informed of all suspected serious unexpected adverse reactions (SUSARs) and all AEs resulting in death or being live-threatening which occur during the trial. Both institutions are informed in case the benefit-risk assessment did change or any others new and significant hazards for patients' safety or welfare did occur. Furthermore, a report on all observed serious adverse events (SAEs) is submitted once a year (Development Safety Update Report (DSUR)).

The EC and the regulatory authorities must be informed of the end of the study. They are provided with a summary of trial results within one year after EOS.

12.7 Notification of Regulatory Authorities

The local regulatory authorities as responsible for each particular investigator and the competent authority are informed before the beginning, during and at the end of the trial according to §67 AMG and §13 GCP-V. Each investigator is obliged to notify his/ her local regulatory authority and the competent authority according §67 AMG and §12 (1, 2, 6) GCP-V if nothing else is agreed upon by contract between investigator and sponsor.

12.8 Registration of the Trial

Prior to the beginning of the clinical phase (FPI) the coordinating investigator registers the trial at a public accessible clinical trial register having the status of a primary register according to the International Clinical Trials Registry Platform (ICTRP) and correspondingly is listed at the International Clinical Trials Registry Platform of the World Health Organization (WHO, http://www.who.int/ictrp/en/). The requirements are fulfilled by the European Clinical Trials Register and submission of EMA Module 1 (Clinical Trial Application Form).

The registration is a prerequisite for a publication in many peer-reviewed journals (see Uniform Requirements for Manuscripts Submitted to Biomedical Journals by the International Committee of Medical Journal Editors; (http://www.icmje.org/ publishing_10register.html).

12.9 Insurance

According to § 40 AMG, the sponsor has to subscribe to an insurance policy which covers in its terms and provisions its legal liability for injuries caused to participating persons. The insurance policy also covers any damage done to the patient, even if the harm done arises out of strictly following the procedures described in this protocol and abiding as applicable law and professional standards. The insurance was taken out at **HDI Global SE**, Niederlassung Düsseldorf (insurance number: **Versicherungsschein-Nr.: 5701031003018**, Policy: **Anmelde-Nr.: 14012020107** maximum limit: € 500.000 per participating person).Any impairment of health which might occur in consequence of trial participation must be notified to the insurance company. The patient is responsible for notification. The insured person must agree to help clarify the cause and the extent of damage with all appropriate measures. He is also obliged to take measures himself to reduce damage as much as possible. During the conduct of the trial, the patient must not undergo other clinical treatment except for cases of emergency. The patient is bound to inform the investigator immediately about any adverse events and additionally drugs taken. The terms and conditions of the insurance must be delivered to the patient.

The insurance company has to be informed about all amendments that could affect patients' safety, and must also receive the actual version of the informed consent.

13 Quality Assurance

13.1 Monitoring

Monitoring is done routinely by personal visits from a clinical monitor according to SOPs of the KKS. The monitor will ensure that the clinical trial is conducted according to the protocol and regulatory requirements by review of source documents, entries into the CRFs and essential documents. The monitor will document the visits in a report for the sponsor. The site will be provided with a follow-up letter of the findings and the necessary actions to be taken.

The investigator must allow the monitor to verify all essential documents and must provide support at all times to the monitor.

If personal visits are not possible due to COVID-19 restrictions alternative measures, e.g. phone visits, might be an alternative option.

Details of monitoring will be defined in the monitoring plan.

13.2 Inspections/ Audits

Regulatory authorities and an auditor authorized by the sponsor may request access to all source documents, CRF, and other trial documentation. Direct access to these documents must be guaranteed by the investigator, who must provide support at all times for these activities.

14 Agreements

14.1 Financing of the Trial

The trial is co-financed by the BMBF program on emergency research funding for COVID-19. and the funds of Heidelberg University Hospital.

14.2 Declaration of Interests

Before the start of the trial, the investigator discloses to the sponsor any proprietary or financial interests he or she might hold in the sponsors/ a funding company, in the investigational product(s), or any commercial organization being involved in the clinical trial. The investigator has also to confirm that he/she has not entered into any financial arrangement whereby the value of compensation paid could affect the outcome of the clinical trial.

The investigator agrees to update this information in case of significant changes.

14.3 Dissemination Policy

14.3.1 Access to data

After the trial has been completed and published, it is planned to make trial data available for re- and meta-analyses. An appropriate repository is defined at the end of the trial.

14.3.2 Reports

The Biostatistician prepares the final trial report together with the Coordinating Investigator within 12 months after the end of the study (EOS).

Annual safety reports (DSURs) are prepared by the pharmacovigilance officer together with the Coordinating Investigator in accordance with legally required timeframes; data reconciliation is carried out where necessary and possible together with the data management of the NCT Trial Center based on already available CRF-AE data.

14.3.3 Publication

All information concerning the trial is confidential before publication. Trial results will be published in medical journals.

15 Signatures

The present trial protocol was subject to critical review and has been approved in the present version by the persons undersigned. The information contained is consistent with:

The current benefit-risk assessment of the investigational medicinal product

Moral, ethical, and scientific principles governing clinical research as set out the principles of GCP and in the applicable version of Declaration of Helsinki.

Date: Signature: ______________________________

16 Declaration of Investigator

I have read the above trial protocol and confirm that it contains all information to conduct the clinical trial. I pledge to conduct the clinical trial according to the protocol.

I will enroll the first patient only after all ethical and regulatory requirements are fulfilled. I pledge to obtain written consent for trial participation from all patients before enrolment.

I know the requirements for accurate reporting of serious adverse events, and I pledge to document and notify such events as described in the protocol.

I pledge to retain all trial-related documents and source data as described.

Date: ____________________

Signature: ______________________________

Name (block letters): ______________________________

Trial Center (address): ______________________________

____

____

17 References

1 WHO. https://www.who.int/blueprint/priority-diseases/key-action/novel-coronavirus/en/. (2020).

2 Yu, J., Ouyang, W., Chua, M. L. K. & Xie, C. SARS-CoV-2 Transmission in Patients With Cancer at a Tertiary Care Hospital in Wuhan, China. *JAMA Oncol*, doi:10.1001/jamaoncol.2020.0980 (2020).

3 Liang, W. *et al.* Cancer patients in SARS-CoV-2 infection: a nationwide analysis in China. *Lancet Oncol* **21**, 335-337, doi:10.1016/s1470-2045(20)30096-6 (2020).

4 Wu, Z. & McGoogan, J. M. Characteristics of and Important Lessons From the Coronavirus Disease 2019 (COVID-19) Outbreak in China: Summary of a Report of 72314 Cases From the Chinese Center for Disease Control and Prevention. *Jama*, doi:10.1001/jama.2020.2648 (2020).

5 UpToDate.com. https://www.uptodate.com/contents/coronavirus-disease-2019-covid-19#. (2020).

6 Rosenbaum, L. Facing Covid-19 in Italy - Ethics, Logistics, and Therapeutics on the Epidemic's Front Line. *N Engl J Med*, doi:10.1056/NEJMp2005492 (2020).

7 Zhou, B., Zhong, N. & Guan, Y. Treatment with convalescent plasma for influenza A (H5N1) infection. *N Engl J Med* **357**, 1450-1451, doi:10.1056/NEJMc070359 (2007).

8 Yeh, K. M. *et al.* Experience of using convalescent plasma for severe acute respiratory syndrome among healthcare workers in a Taiwan hospital. *J Antimicrob Chemother* **56**, 919-922, doi:10.1093/jac/dki346 (2005).

9 Hung, I. F. *et al.* Convalescent plasma treatment reduced mortality in patients with severe pandemic influenza A (H1N1) 2009 virus infection. *Clin Infect Dis* **52**, 447-456, doi:10.1093/cid/ciq106 (2011).

10 Luke, T. C., Kilbane, E. M., Jackson, J. L. & Hoffman, S. L. Meta-analysis: convalescent blood products for Spanish influenza pneumonia: a future H5N1 treatment? *Ann Intern Med* **145**, 599-609, doi:10.7326/0003-4819-145-8-200610170-00139 (2006).

11 Shen, C. *et al.* Treatment of 5 Critically Ill Patients With COVID-19 With Convalescent Plasma. *Jama*, doi:10.1001/jama.2020.4783 (2020).

12 Liu, Y. *et al.* Neutralizing Activity of BNT162b2-Elicited Serum - Preliminary Report. *N Engl J Med*, doi:10.1056/NEJMc2102017 (2021).

13 Sadoff, J. *et al.* Interim Results of a Phase 1-2a Trial of Ad26.COV2.S Covid-19 Vaccine. *N Engl J Med*, doi:10.1056/NEJMoa2034201 (2021).

14 Voysey, M. *et al.* Single-dose administration and the influence of the timing of the booster dose on immunogenicity and efficacy of ChAdOx1 nCoV-19 (AZD1222) vaccine: a pooled analysis of four randomised trials. *The Lancet*, doi:10.1016/S0140-6736(21)00432-3.

15 Duan, K. *et al.* The feasibility of convalescent plasma therapy in severe COVID-19 patients: a pilot study. *medRxiv*, 2020.2003.2016.20036145, doi:10.1101/2020.03.16.20036145 (2020).

16 Chen, G. *et al.* Clinical and immunologic features in severe and moderate Coronavirus Disease 2019. *J Clin Invest*, doi:10.1172/jci137244 (2020).

17 Desborough, M. *et al.* Fresh frozen plasma for cardiovascular surgery. *Cochrane Database Syst Rev*, Cd007614, doi:10.1002/14651858.CD007614.pub2 (2015).

18 Wan, Y. *et al.* Molecular Mechanism for Antibody-Dependent Enhancement of Coronavirus Entry. *J Virol* **94**, doi:10.1128/jvi.02015-19 (2020).

19 Cheng, Y. *et al.* Use of convalescent plasma therapy in SARS patients in Hong Kong. *Eur J Clin Microbiol Infect Dis* **24**, 44-46, doi:10.1007/s10096-004-1271-9 (2005).

20 Chen, L., Xiong, J., Bao, L. & Shi, Y. Convalescent plasma as a potential therapy for COVID-19. *Lancet Infect Dis* **20**, 398-400, doi:10.1016/s1473-3099(20)30141-9 (2020).

21 Roback, J. D. & Guarner, J. Convalescent Plasma to Treat COVID-19: Possibilities and Challenges. *Jama*, doi:10.1001/jama.2020.4940 (2020).

22 Casadevall, A. Passive antibody administration (immediate immunity) as a specific defense against biological weapons. *Emerg Infect Dis* **8**, 833-841, doi:10.3201/eid0808.010516 (2002).

23 Casadevall, A. & Pirofski, L. A. The convalescent sera option for containing COVID-19. *J Clin Invest*, doi:10.1172/jci138003 (2020).

24 Cao, B. *et al.* A Trial of Lopinavir-Ritonavir in Adults Hospitalized with Severe Covid-19. *N Engl J Med*, doi:10.1056/NEJMoa2001282 (2020).

25 Soo, Y. O. *et al.* Retrospective comparison of convalescent plasma with continuing high-dose methylprednisolone treatment in SARS patients. *Clin Microbiol Infect* **10**, 676-678, doi:10.1111/j.1469-0691.2004.00956.x (2004).

26 Mair-Jenkins, J. *et al.* The effectiveness of convalescent plasma and hyperimmune immunoglobulin for the treatment of severe acute respiratory infections of viral etiology: a systematic review and exploratory meta-analysis. *J Infect Dis* **211**, 80-90, doi:10.1093/infdis/jiu396 (2015).

27 van Griensven, J. *et al.* Evaluation of Convalescent Plasma for Ebola Virus Disease in Guinea. *New England Journal of Medicine* **374**, 33-42, doi:10.1056/NEJMoa1511812 (2016).

28 Pandey, S. & Vyas, G. N. Adverse effects of plasma transfusion. *Transfusion* **52 Suppl 1**, 65s-79s, doi:10.1111/j.1537-2995.2012.03663.x (2012).

29 Watkins, C., Huang, X., Latimer, N., Tang, Y. & Wright, E. J. Adjusting overall survival for treatment switches: commonly used methods and practical application. *Pharm Stat* **12**, 348-357, doi:10.1002/pst.1602 (2013).

30 Robins, J. M. & Finkelstein, D. M. Correcting for noncompliance and dependent censoring in an AIDS Clinical Trial with inverse probability of censoring weighted (IPCW) log-rank tests. *Biometrics* **56**, 779-788, doi:10.1111/j.0006-341x.2000.00779.x (2000).

31 Robins, J. M. & Tsiatis, A. A. Correcting for non-compliance in randomized trials using rank preserving structural failure time models. *Communications in Statistics - Theory and Methods* **20**, 2609-2631, doi:10.1080/03610929108830654 (1991).

32 International Council for Harmonization. E9(R1): Addendum on estimands and sensitivity analysis in clinical trials. International Council for Harmonization. E9(R1): Addendum on estimands and sensitivity analysis in clinical trials, 2019. (2019).

33 Lambden, S., Laterre, P. F., Levy, M. M. & Francois, B. The SOFA score—development, utility and challenges of accurate assessment in clinical trials. *Critical Care* **23**, 374, doi:10.1186/s13054-019-2663-7 (2019).

18 Appendices

18.1 Ordinal Scale for Clinical Improvement

According to Cao et al, NEJM 2020 [24].

1. not hospitalized with resumption of normal activities;

2. not hospitalized, but unable to resume normal activities;

3. hospitalized, not requiring supplemental oxygen;

4. hospitalized, requiring supplemental oxygen;

5. hospitalized, requiring nasal high-flow oxygen therapy or, noninvasive mechanical ventilation

6. hospitalized, requiring ECMO, invasive mechanical ventilation, or both

7. death.

18.2 Sequential Organ Failure Assessment (SOFA) score

Assessment of the Sequential Organ Failure Assessment (SOFA) score [33]

Respiratory system

PaO2/FiO2 (mmHg)	Score
> 400	0
< 400	1
< 300	2
< 200 with respiratory support	3
< 100 with respiratory support	4

Nervous system

Glasgow Coma Scale	Score
15	0
13–14	1
10–12	2
6–9	3
< 6	4

Cardiovascular system

Mean arterial pressure (MAP) OR administration of vasopressors required	Score
MAP > 70 mmHg	0
MAP < 70 mm/Hg	1
Dopamine ≤ 5 µg/kg/min or dobutamine any dose	2
Dopamine > 5 µg/kg/min OR epinephrine ≤ 0.1 µg/kg/min OR norepinephrine 0.1 µg/kg/min	3
Dopamine > 15 µh/kg/min OR epinephrine > 0.1 µg/kg/min OR norepinephrine >0.1 µg/kg/min	4

Liver

Bilirubin (mg/dl) [µmol/L] SOFA score	Score
< 1.2 (< 20)	0
1.2–1.9 [20–32]	1
2.0–5.9 [33–101]	2
6.0–11.9 [102–204]	3
> 12.0 [> 204]	4

Coagulation

Platelets ×103/ml SOFA score	Score
> 150	0
< 150	1
< 100	2
< 50	3
< 20	4

Kidneys

Creatinine (mg/dl) [µmol/L]; urine output	Score
< 1.2 [< 110]	0
1.2–1.9 [110–170]	1
2.0–3.4 [171–299]	2
3.5–4.9 [300–440] (or urine output < 500 ml/day)	3
> 5.0 [> 440]; urine output < 200 ml/day	4

https://www.mdcalc.com/sequential-organ-failure-assessment-sofa-score

**RECOVER | Protocol No:** SARS-CoV-2CP-HD-001 **| EudraCT No.:** 2020-001632-10<br><br>**Summary of Changes V1.2 → V3.0**			
Intermediate Version 2.0 29th Oct 2020 (Approval 10th Nov 2020)			
**Protocol Section**	**Content**	**Justification**	**Date of approval**
All sections	Date and Version number updated in the header.	For clarity.	
Title page	Footnote with clarification inserted regarding the study title.	For clarity.	30st Mar 2021
Responsibilities	- Project Management – Dr. Julia Gáll added<br>- Protocol Writing Committee – Dr. Julia Gáll added<br>- Data Management – Christina Klose deleted	Update.	10th Nov 2020
Responsibilities	- Contact telephone number and email-address of the NCT trial center corrected<br>- Protocol Writing Committee – correction of academic titles	Update.	30st Mar 2021
Throughout Protocol	Cross-over at Day 10 - + 2 days added	Window for cross-over was expanded.	10th Nov 2020
Throughout Protocol	Addition of the use of plasma of vaccinated donors (Updated IMP name: RECOVER CT SARS-CoV-2-ab Plasma COVID-19 (HD): Patients can receive plasma either from convalescents and/or from vaccinated persons.	With data becoming available of increased titers of antibodies in vaccinated donors and increased numbers of people being vaccinated (i.e. increased access to donors), we considered vaccinated donors in addition to convalescent donors	30st Mar 2021
Synopsis	Number of Trial Sites expanded to 10-15 medical centers	to increase enrollment and speed of enrollment	10th Nov 2020
Synopsis and 5.3	Inclusion criteria 4: Blood hemoglobin concentration changed from ≥ 10 g/dl to ≥ 8 g/dl	Main concern for limitation was the amount of blood collections from the trial. With decreasing blood collections, we also decreased the hemoglobin cut-off to increase the number of eligible patients, particularly those with preexisting hematological malignancies.	10th Nov 2020

Synopsis – Trial Schedule	The Trial Schedule was revised by reducing the number of assessments, blood collections and follow-up tests	To ensure a smooth conduct of the study by the study teams and to expose patients to less follow-up treatments. The revision was done by ensuring patient´s safety without impacting the study endpoints.	10th Nov 2020
Synopsis – Trial Schedule	Sentence added:<br>*** Day 1 = day of randomisation into standard arm or day of first plasma administration in the experimental arm	Definition of Day 1 for clarity and consistency within the study protocol and between the study protocol and other study documents.	30st Mar 2021
Synopsis – Trial Schedule – Footnote 26	Revision of wording:<br>Patients must be followed for AEs up to day 28…	Correction for clarity and consistency within the study protocol and between the study protocol and other study documents.	30st Mar 2021
Abbreviations	- CFS (Clinical Frailty Scale) added<br>- ECOG (Eastern Cooperative Oncology Group) added	According to abbreviations in the text.	10th Nov 2020
Abbreviations	- PVP – Post-Vaccination Plasma = vaccine-boosted plasma added	According to abbreviations in the text.	30st Mar 2021
1 Introduction	Sentence added according to new literature:<br>Furthermore, reports from SARS-CoV-2 vaccination trials suggest that subjects after the second boost immunization develop high titers of neutralizing antibodies and thus may be excellent candidates for plasma donation[12-14].	Update.	30st Mar 2021
4 Trial Duration and Schedule	Section revised according to the new trial schedule. Days for measurement of viral load in nasopharyngeal swabs and assessment of treatment response updated.	Consistency with revised trial schedule.	10th Nov 2020
5.4 Exclusion criteria	Deletion of exclusion criteria 7.	Inconsistency in the protocol was corrected (see exclusion criteria in Synopsis). Exclusion criteria 7 was not deleted by mistake after the last protocol amendment.	30st Mar 2021
6.4 Dosage Schedule	The sentence: “CP infusions is administered on two days: visit day 1 (≤ 7 days after baseline) and day 2” was changed to “CP infusions is administered on two days: visit day 1 (≤ 7 days after screening/baseline) and day 2”	Consistency with trial schedule.	10th Nov 2020

6.4 Dosage Schedule	Sentence: The CP can be administered after thawing of cryopreserved CP < -30°C corrected to ≤ 30°C.	Correction.	10th Nov 2020
6.4 Dosage Schedule	In the sentence: The CPs are going to be administered with an infusion rate of 80-100 ml/ hour within a four hour period for each CP bag – the expression “is going to be administered” was replaced by “must be administered”.	Correction of grammar.	10th Nov 2020
6.4 Dosage Schedule	Addition of the following sentences for clarification:<br>Patients can receive plasma either from convalescents and/or from vaccinated persons.<br>Patients with persistence of SARS-CoV-2 virus load after day 28 may receive additional treatment with CP/PVP. These situations have to be discussed on a patient by patient basis with the principle investigator.	Update.	30st Mar 2021
7.1 Description of Study Visits	The sentence was added:<br>To lessen patient’s burden, all screening/baseline assessments maybe completed on day 1. In this case, all screening/baseline assessments must be completed before randomisation and CP administered after completion of all other visit procedures.	For clarification and better understanding that screening/baseline assessments can be completed also at day 1 before randomisation. This also facilitates enrollment/ inclusion, especially in case of severe course of disease.	10th Nov 2020
7.1 Description of Study Visits	The Sentence: “**Laboratory safety assessments** (hematology, coagulation and blood chemistry) are measured according at each visit until hospital discharge” was changed to “**Laboratory safety assessments** (hematology, coagulation and blood chemistry) are measured according to the Trial Schedule (Table 1) until hospital discharge.”	Consistency with revised trial schedule.	10th Nov 2020
7.1 Description of Study Visits	Table 2 (Clinical laboratory measurements) was updated according to the revised trial schedule (separation of laboratory measurements for standard and extended blood chemistry and coagulation).	Consistency with revised trial schedule.	10th Nov 2020
7.2.2 Sample Collection and Handling	The shipment address for Translational Research was corrected.	Correction.	10th Nov 2020

7.3.1 Seven point ordinal scale	The sentence: “This scale is clearly defined and values will be obtained daily for each patient until day 28, thereafter weekly until day 56, then on day 70 and finally at day 84.” was changed to “This scale is clearly defined and values will be obtained daily for each patient until day 28, thereafter weekly until day 56, and finally at day 84.”	Consistency with revised trial schedule.	10th Nov 2020
7.3.1 Seven point ordinal scale	In the sentence “Of note, inclusion criteria of the trial determine that all patients to be included are either in #4 or #5 at time of inclusion into the trial.” #3 was added.	Consistency with in-/exclusion criteria defined in Synopsis and 5.3 of the protocol.	10th Nov 2020
7.3.3. Laboratory parameters	First paragraph was revised according to revised trial schedule. Days for collection of nose/thoat swabs and serum for SARS-Cov-2 diagnostic were updated.	Consistency with revised trial schedule.	10th Nov 2020
8 Ancillary and Post Trial Care	Section updated according to revised trial schedule. The sentence: “A telephone survey of patients (if applicable) or treating physician is conducted weekly after hospital discharge before day 84 (experimental arm and standard arm without cross over) or day 94 (standard arm in case of cross over)” was replaced by the sentence: “All discharged patients are followed by regular phone calls (scheduled days: 3, 7, 10, 14, 28, 56, 84).”	Consistency with revised trial schedule.	10th Nov 2020
9.2 Period of Surveillance and Documentation	Revision of wording:<br>AEs must be followed up to day 28…	Correction according to update in the Trial Schedule.	30st Mar 2021
10.3 Sample size calculation	The sentence was added:<br>Based on first results obtained in vaccinated subjects, we allow by amendment apart from convalescent plasma also the use of plasma obtained from vaccinated subjects, in which high amounts of neutralizing antibodies are present. We assume that plasma from vaccinated subjects are at least as effective as convalescent plasma[12-14].	Explanation of the rationale of the amendment.	30st Mar 2021

10.5.5 Analysis of the Secondary Endpoints	The sentence was added:<br>Further, the impact of type of plasma (convalescent, vaccine-boosted) is assessed on an explorative basis using univariable analysis as well as multivariable models for the primary and secondary endpoints.	Explanation of the planned evaluation.	30st Mar 2021
17 References	Addition of references from actual literature.	Update.	30st Mar 2021

SAP for Final Analysis

RECOVER

A Randomized Open label Phase-II Clinical Trial with or without Infusion of Plasma from Subjects after Convalescence of SARS-CoV-2 Infection in High-Risk Patients with Confirmed Severe SARS-CoV-2 Disease

Clinical Trial Code: SARS-CoV-2CP-HD-001

EudraCT No.: 2020-001632-10

SAP refers to Protocol version 3.0

Signatures

We, the undersigned, have read the Statistical Analysis Plan and agree that it contains all information necessary for the interim/final analysis.

Representative of the Responsible Biometrician

Author of the Statistical Analysis Plan

Lukas Baumann

Date / Signature

Responsible Biometrician

Dr. sc. hum Johannes Krisam

Date / Signature

Coordinating Investigator

Prof. Dr. Carsten Müller-Tidow

Date / Signature

Contents

Signatures ... 2
1 Purpose of this SAP ... 5
2 Objective of the Trial ... 5
3 Study design ... 5
4 Analysis sets ... 6
5 Definitions of endpoints to be analysed ... 6
5.1 Primary Endpoint ... 6
5.2 Secondary Endpoints ... 7
5.3 Exploratory Endpoints ... 7
5.4 Safety Endpoints ... 10
6 Methods for Withdrawals and Missing Data ... 10
7 Statistical methods ... 10
7.1 Descriptive methods ... 11
7.2 Baseline characteristics ... 11
7.3 Primary analysis ... 15
7.4 Sensitivity and supplementary analyses ... 16
7.4.1 Sensitivity analysis 1 ... 16
7.4.2 Sensitivity analysis 2 ... 16
7.4.3 Supplementary analysis 1 ... 16
7.4.4 Supplementary analysis 2 ... 17
7.4.5 Supplementary analysis 3 ... 18
7.4.6 Supplementary analysis 4 ... 18
7.4.7 Supplementary analysis 5 ... 18
7.5 Further analyses based on the primary endpoint ... 19
7.5.1 Impact of timing of treatment ... 19

7.5.2 Impact of type of treatment 19
7.5.3 Impact of type of COVID infection wave 19
7.5.4 Impact of reception of monoclonal antibodies 19
7.5.5 Impact of virus variant 19
7.5.6 Impact of vaccination status 20
7.5.7 Impact of number of neutralizing antibodies at baseline 20
7.6 Analysis of the secondary endpoints 20
7.6.1 Analysis of overall survival 20
7.6.2 Analysis of time until discharge 21
7.6.3 Analysis of requirement of mechanical ventilation 21
7.6.4 Analysis of SARS-CoV-2 viral load and antibody titres 21
7.6.5 Analysis of cytokine levels 22
7.7 Analysis of exploratory endpoints 22
7.8 Analysis of safety endpoints 22
7.9 Subgroup analyses 23
8 Differences to trial protocol 23
9 Interpretation of results 23
10 Data problems 23
11 Estimands framework 23
11.1 Main estimand 23
12 Software 24
13 References 24

1 Purpose of this SAP

In the trial protocol the assumptions, methods and procedures for the statistical evaluation are already described. The statistical analysis plan (SAP) aims to specify more precisely the evaluations carried out in the statistical analysis. This SAP refers to the final analysis after study completion, no interim analysis was planned or conducted.

2 Objective of the Trial

The primary objective of the trial is to assess the time from randomization until an improvement within 84 days defined as two points on a seven point ordinal scale (given in chapter 5.1) or live discharge from the hospital in high-risk patients (group 1 to group 4 as defined below) with SARS-CoV-2 infection requiring hospital admission by infusion of plasma from subjects after convalescence of SARS-CoV-2 infection/vaccination against SARS-CoV-2 or standard of care.

The four high-risk patient groups are:

1. Patients with pre-existing or concurrent hematological malignancy and/or active cancer therapy (including chemotherapy, radiotherapy, surgery) within the last 24 months or less.
2. Patients with chronic immunosuppression not meeting the criteria of group 1.
3. Patients of age ≥ 50 - 75 years meeting neither the criteria of group 1 nor group 2 and at least one of these criteria: Lymphopenia < 0.8 x G/l and / or D-dimer > 1µg/mL.
4. Patients of age ≥ 75 years meeting neither the criteria of group 1 nor group 2.

The link between the primary objective and the primary analysis is also described in the sense of the new estimands framework which is given in chapter 10.

Secondary objectives of the trial are:

- to assess overall survival, and the overall survival rate at 28, 56 and 84 days,
- to assess SARS-CoV-2 viral clearance and load as well as antibody titres,
- to assess percentage of patients that required mechanical ventilation, and
- to assess time from randomization until discharge
- to assess cytokine changes over time.

3 Study design

RECOVER is a randomized, open-label, multicenter phase II trial, designed to assess the clinical outcome of SARS-CoV-2 disease in high-risk patients (group 1 to group 4) following treatment with plasma from subjects after convalescence of a SARS-CoV-2 infection or after vaccination against SARS-CoV-2 compared with standard of care. Randomisation is done

either into the experimental arm “infusion of convalescent plasma (CP) or vaccine-boosted plasma (=Post-Vaccination Plasma/PVP) ” or into the standard arm “standard of care” in a 1:1 ratio using block randomisation stratified for the factor “patient group”, defined by groups 1 to 4 as given in Chapter 2. The total planned sample size is n = 174 (84 per group).

4 Analysis sets

Full Analysis Set

The Full Analysis Set (FAS) includes all randomised patients with treatment groups assigned in accordance with the randomisation, regardless of the treatment actually received. Patients who were randomised but did not subsequently receive treatment are included in the FAS. The analysis of data using the FAS follows the Intention-to-Treat principles (ITT).

Per Protocol Set

In the per protocol set (PPS), patients with important protocol deviations (e.g. patients not meeting the inclusion/exclusion criteria, too early cross-over, reception of only one instead of two bags of infusion) are excluded. Analyses based on the PPS are known to be biased and are therefore of minor importance for the interpretation of the results and only serve as supplementary analyses. The determination of patients with major protocol deviations will be done in a data review meeting which will take place before database lock.

Safety Set

All enrolled patients who received treatment are subjected to the safety analysis. Patients will be evaluated with regard to the treatment actually received, meaning that patients who cross over from the control to the experimental arm will be evaluated in the experimental arm at day 10 (+ 2 days) onwards.

5 Definitions of endpoints to be analysed

5.1 Primary Endpoint

The primary efficacy endpoint is time from randomisation to clinical improvement within 84 days by two points on a seven point ordinal scale (in comparison to the status at randomisation) or live discharge from the hospital. If a patient needs to be transferred to another hospital, he or she will not be considered as discharged. If a patient is transferred to a rehabilitation clinic or nursing home, he or she will be considered as discharged. The categories of the seven point ordinal scale are:

1. Not hospitalized with resumption of normal activities
2. Not hospitalized, but unable to resume normal activities

3. Hospitalized, not requiring supplemental oxygen
4. Hospitalized, requiring supplemental oxygen
5. Hospitalized, requiring nasal high-flow oxygen therapy, or noninvasive mechanical ventilation
6. Hospitalized, requiring ECMO, invasive mechanical ventilation, or both
7. Death

5.2 Secondary Endpoints

Secondary endpoints are:

- Overall survival, defined as the time from randomization until death from any cause
- 28-day, 56-day and 84-day overall survival rate
- Requirement of mechanical ventilation at any time during hospital stay (yes/no)
- Time from randomization until discharge. If a patient needs to be transferred to another hospital, he or she will not be considered as discharged. If a patient is transferred to a rehabilitation clinic or nursing home, he or she will be considered as discharged.
- SARS-CoV-2 viral clearance and load as well as antibody titres (until hospital discharge on day 1, 3, 5, 10, 14, 28, 56 and 84), comprising:
    - Result of smear (negative / positive / invalide)
    - CT-value
    - Serodiagnostic by ELISA (OD ratio)
    - Neutralizing antibody titre
- Cytokine profiles

5.3 Exploratory Endpoints

Exploratory endpoints are:

- Vital signs (on day 1, 3, 5, 10 and 14), comprising:
    - ECOG performance status (ordinal, 0 to 4)
    - Systolic blood pressure in mmHg
    - Diastolic blood pressure in mmHg
    - Pulse rate in beats/min
    - Respiratory rate in breaths/min
- Seven point ordinal scale as defined in chapter 5.1 (until hospital discharge daily from day 1-28, weekly from day 29-55 and on day 56 and 84)
- Sequential Organ Failure Assessment (SOFA) Score (until hospital discharge on day 1 and 10)

- Oxygen saturation:
    - Partial pressure of oxygen (PaO_2) in mmHg
    - Partial pressure of carbon dioxide ($PaCO_2$) in mmHg
    - ECMO (yes / no)
    - Type of ventilation (no / nasal high-flow oxygen therapy / noninvasive mechanical ventilation / invasive ventilation / tracheotomy / ambient air / standard oxygen sources)
    - Amount of oxygen in litres/min
    - Oxygen saturation (SaO_2) in percent
    - Amount of supplemental oxygen that is required to keep SaO_2 above 94% in litres/min
    - Fraction of inspired oxygen (FiO_2) in percent
- Hematology (until hospital discharge on day 1, 3, 5, 10, 14, 28, 56 and 84), comprising:
    - Hemoglobin
    - RBC
    - WBC
    - Thrombocytes
- Blood chemistry and coagulation (until hospital discharge on day 1, 3, 5, 10, 14, 28, 56 and 84), comprising:
    - BUN
    - Creatinine
    - Albumin
    - AST/SGOT
    - ALT/SGPT
    - Total bilirubin
    - GGT
    - AP
    - LDH
    - Sodium
    - Potassium
    - Magnesium
    - Calcium
    - Uric acid
    - Troponin
    - CK
    - CK-MB

    - PTT
    - ATIII
    - D-dimer
    - Fibrinogen
    - Ferritin (measured at day 1 only)
    - Transferrin (measured at day 1 only)
    - Transferrin saturation (measured at day 1 only)
    - CRP
    - Total protein (measured at day 1 only)
    - IL6 (measured at day 1 only)
    - Procalcitonin (measured at day 1 only)
    - Total IgG (measured at day 1 only)
    - IgA (measured at day 1 only)
    - IgM (measured at day 1 only)
    - Lactate
    - INR
- Assessment of concomitant medications
- Signs/symptoms (on day 10 in case of cross-over), comprising:
    - Cough (yes / no)
    - Fever (yes / no)
    - Myalgia (yes / no)
    - Fatigue (yes / no)
    - Diarrhoea (yes / no)
    - Vomiting (yes / no)
    - Headache (yes / no)
    - Abdominal pain (yes / no)
    - Nausea (yes / no)
    - Olfactory or taste disorder (yes / no)
    - Dyspnoea (yes / no)
    - Confusion (yes / no)
- Physical examination (on day 10 in case of cross-over), comprising:
    - General appearance (normal / abnormal)
    - Lung (normal / abnormal)
    - Cardiac auscultation (normal / abnormal)
    - Cardiac percussion (normal / abnormal)
    - Abdominal auscultation (normal / abnormal)

  - Abdominal percussion (normal / abnormal)
  - Abdominal palpation (normal / abnormal)
  - Palpation of lymph node sites (normal / abnormal)
  - Neurological examination (normal / abnormal)
  - Peripheral edema (normal / abnormal)
- ECG, comprising
  - ECG abnormal / abnormal
  - Clinically significant (yes / no)
  - Heart rate in beats/min
  - Rhythm (normal sinus / other)

5.4 Safety Endpoints

Safety endpoints comprise:

- Adverse Events (AEs)
- Severity of AEs
- Serious adverse events (SAEs)
- Relation of AEs to the study treatment
- Dose modifications for toxicity
- Discontinuation of study treatment

6 Methods for Withdrawals and Missing Data

Withdrawals according to study protocol will not be excluded unless the patient withdrew his/her consent declining explicitly the use of the data for analysis (s. informed consent). As long as a written informed consent is available without denied permission to evaluate the documented data, the data already collected as well as follow-up data will be included in the final analysis.

For patients with incomplete follow-up, time to last follow-up date is used as the censoring time in the analysis of time-to-event data. Otherwise, no imputation of missing data will be conducted.

7 Statistical methods

The statistical evaluation is carried out under the supervision of the Supervising Statistician. Statistical analysis is based on the International Conference on Harmonization (ICH) Guidelines “Structure and Content of Clinical Study Reports” and “Statistical Principles for Clinical Trials”. All statistical procedures are done according to the current Standard Operating Procedures (SOPs) of the Institute of Medical Biometry, University of Heidelberg (IMBI).

7.1 Descriptive methods

A CONSORT flow diagram will be created to display the progress of all participants through the trial. This includes the number of patients assessed for eligibility and the number of patients excluded because they did not meet inclusion criteria, declined to participate, or any other reason, the number of randomized patients (per group), and number of missed intervention assignments / incomplete interventions. In addition, the number of patients in the FAS and PPS will be given and reported reasons for exclusion from the PP set will be summarized per treatment group.

Continuous variables will be described using number of observations, mean, standard deviation, median, Q1, Q3, minimum, maximum and number of missing values. For categorical variables, absolute and relative frequencies will be given with missing values being reported as a separate category. Percentages for categorical variables will be based on all non-missing values in the respective groups. Time to event endpoints will be described by Kaplan-Maier estimates (including the numbers at risk for different time points), median survival time (or other quantiles if median survival time cannot be calculated), number of events and number of censorings. The descriptive methods described above will be used separately for each treatment group.

The test to be applied is specified in the description of the analyses of the respective endpoint. If a test is conducted to compare treatment groups, point estimates with respective 95% confidence intervals will be provided additionally to the descriptive p-value. For the chi-square test, the difference of rates and/or the risk ratio will be given where appropriate, for the t-test the difference between means will be provided and for the Mann-Whitney U test, the Hodges-Lehmann estimate will be presented as point estimate.

Descriptive results will be presented in tables, appropriately supplemented with figures if specified in the respective analysis section. Survival endpoints will always be supplemented with a Kaplan-Meier survival curve. Listings will be used to present data collected in free-text formats.

7.2 Baseline characteristics

The following baseline characteristics will be collected and described in the FAS set, the PP set and the safety set:

- Demographics, comprising:
  - Sex (male / female)
  - Year of birth
  - Ethnic origin (asian / black / caucasian/white / hispanic / other)

- Medical history
    - Haematological malignancy
    - Solid tumor
    - Immunosuppression due to disease (immunodeficiency)
    - Diabetes
    - Cardiovascular disease
    - Obesity
    - Lung disease
- – Assessment of Concomitant medications
    - COVID19 therapy from 7 days before study entry up to day 10
        - Steroids
        - Remdesivir or other antiviral drugs
        - Monoclonal antibodies
        - Tociluzimab
        - Ruxolitinib
- Smoking history, comprising
    - Yes / non-smoker / ex-smoker
    - Smoking duration in years
    - Smoking amount in packs per day
- Signs/symptoms, comprising:
    - Cough (yes / no)
    - Fever (yes / no)
    - Myalgia (yes / no)
    - Fatigue (yes / no)
    - Diarrhoea (yes / no)
    - Vomiting (yes / no)
    - Headache (yes / no)
    - Abdominal pain (yes / no)
    - Nausea (yes / no)
    - Olfactory or taste disorder (yes / no)
    - Dyspnoea (yes / no)
    - Confusion (yes / no)
- Vital signs, comprising:
    - ECOG performance status (ordinal, 0 to 4)
    - Body temperature in degrees Celsius
    - Systolic blood pressure in mmHg
    - Diastolic blood pressure in mmHg

- Pulse rate in beats/min
- Respiratory rate in breaths/min

- Physical examination, comprising:
  - General appearance (normal / abnormal)
  - Lung (normal / abnormal)
  - Cardiac auscultation (normal / abnormal)
  - Cardiac percussion (normal / abnormal)
  - Abdominal auscultation (normal / abnormal)
  - Abdominal percussion (normal / abnormal)
  - Abdominal palpation (normal / abnormal)
  - Palpation of lymph node sites (normal / abnormal)
  - Neurological examination (normal / abnormal)
  - Peripheral edema (normal / abnormal)
- Clinical Frailty Scale (very fit / well / managing well / vulnerable / mildly frail / moderately frail / severely frail / very severely frail / terminally ill)
- Seven point ordinal scale as defined in chapter 5.1
- Oxygen saturation
  - Partial pressure of oxygen (PaO_2) in mmHg
  - Partial pressure of carbon dioxide ($PaCO_2$) in mmHg
  - ECMO (yes / no)
  - Type of ventilation (no / nasal high-flow oxygen therapy / noninvasive mechanical ventilation / invasive ventilation / tracheotomy / ambient air / standard oxygen sources)
  - Amount of oxygen in litres/min (will only be evaluated for any time point if patient does not simultaneously receive any oxygen)
  - Oxygen saturation (SaO_2) in percent (will only be evaluated for any time point if patient does not simultaneously receive any oxygen)
  - Amount of supplemental oxygen that is required to keep SaO_2 above 94% in litres/min
  - Fraction of inspired oxygen (FiO_2) in percent
- ECG, comprising
  - ECG abnormal / abnormal
  - Clinically significant (yes / no)
  - Heart rate in beats/min
  - Rhythm (normal sinus / other)
- Hematology (until hospital discharge on day 1, 3, 5, 10, 14, 28, 56 and 84), comprising:
  - Hemoglobin

- RBC
- WBC
- Thrombocytes

- Blood chemistry and coagulation (until hospital discharge on day 1, 3, 5, 10, 14, 28, 56 and 84), comprising:
  - BUN
  - Creatinine
  - Albumin
  - AST/SGOT
  - ALT/SGPT
  - Total bilirubin
  - GGT
  - AP
  - LDH
  - Sodium
  - Potassium
  - Magnesium
  - Calcium
  - Uric acid
  - Troponin
  - CK
  - CK-MB
  - PTT
  - ATIII
  - D-dimer
  - Fibrinogen
  - Ferritin (measured at day 1 only)
  - Transferrin (measured at day 1 only)
  - Transferrin saturation (measured at day 1 only)
  - CRP
  - Total protein (measured at day 1 only)
  - IL6 (measured at day 1 only)
  - Procalcitonin (measured at day 1 only)
  - Total IgG (measured at day 1 only)
  - IgA (measured at day 1 only)
  - IgM (measured at day 1 only)

   - Lactate
   - INR
- Urinalysis, comprising:
   - pH-value
   - Glucose
   - Protein
- Other virology, comprising:
   - Anti-HAV (negative / positive)
   - Anti-HBV (negative / positive)
   - Anti-HCV IgG (negative / positive)
   - HIV (negative / positive)

Baseline characteristics will be summarized for each treatment group separately using the methods described in Section 7.1.

7.3 Primary analysis

The primary analysis will assess the null hypothesis "the cumulative improvement curves for the primary endpoint in the experimental and control arm are equal", i.e.

$$H_0: S_E = S_C$$

against the alternative hypothesis

$$H_1: S_E \neq S_C$$

at a two-sided significance level of α=5%. This will be achieved by using the log-rank test stratified for the factor "patient group" as used in the randomisation procedure. The event "death from any cause" will be handled by censoring those patients at day 84 (in analogy to the approach of Cao et al, NEJM 2020). Using this approach ensures that deceased patients are considered as "not improved" over the whole observation period of 84 days. The primary analysis will be based on the FAS and handles intercurrent events as described in chapter 11.1.

The main estimate (corresponding to the main estimand as described in chapter 11.1) for the hazard ratio for treatment group will be computed using a Cox regression model stratifying for the factor "patient group" together with a 95% profile-likelihood confidence interval. The cumulative improvement curves are calculated using the Kaplan-Meier method together with 95% log-log-type confidence bands, and will be calculated separately for both treatment groups. In addition, cumulative improvement curves will also be calculated separately for each patient group per treatment group, and descriptive log-rank tests will be done to compare the cumulative improvement groups for each patient group. Patients who drop out during the trial or are lost to follow-up are taken into account as censored observations.

7.4 Sensitivity and supplementary analyses

All sensitivity analyses are conducted in the FAS and handle intercurrent events as described in chapter 11.1. If a supplementary analyses is conducted in a different analysis set or handles an intercurrent event differently this is explicitly mentioned.

7.4.1 Sensitivity analysis 1

An unstratified log-rank test will be conducted, which is based on the assumption of identical baseline risk in the different patient groups. As a sensitivity estimate a hazard ratio for the treatment group based on an unstratified Cox-regression will be calculated together with a 95% profile-likelihood confidence interval.

7.4.2 Sensitivity analysis 2

A Gray's test (Gray, 1988) stratified for "patient group" will be conducted where the death from any cause without previous improvement is treated as a competing risk. As a sensitivity estimate a subdistribution hazard based on a stratified Fine and Gray's competing risk regression using PROC PHREG will be calculated together with 95% confidence intervals.

7.4.3 Supplementary analysis 1

A supplementary analysis of the primary endpoint will involve the inverse probability censoring weighting (IPCW) approach to estimate a treatment effect in the hypothetical scenario that patients from the control group had not switched to the experimental group (Robins & Finkelstein (2000)). This type of model requires to estimate the probability for a patient to switch to the experimental group based on (time-dependent) covariates, which in our case will be the baseline factor "patient group", together with the time-varying factors "seven point ordinal scale measuring clinical improvement" and "viral load". For this analysis, patients who cross over to the standard treatment will be considered as censored observations. Thus, however, patients who crossover due to poor prognosis will not contribute to the same degree as patients with a favourable prognosis less likely to switch. Thus, in order to adjust for informative censoring in case of treatment switch, patients in the control group will receive a weighting, such that uncensored patients with a high crossover risk due to unfavourable prognostic factors will receive higher weights than patients with a low crossover risk due to favourable prognostic factors. It should be noted that this method relies on the assumption that there are no unmeasured confounders influencing the probability to crossover, which is hardly verifiable, results will therefore needed to be cautiously interpreted.

o conduct the IPCW method, firstly, the data needs to be transformed into a long format, and the follow-up period needs to be split up in time intervals. In a first step, the probability to remain on the assigned treatment will be estimated for the control group using a repeated measures logistic regression model via PROC GENMOD with an unstructured covariance

matrix, including only the patient group and the baseline ordinal scale as predictors. This model will estimate the probabilities

$$p_{ij} := P\big[\text{No crossover for patient } i \text{ in follow} - \text{up period } [t_j, t_{j+1}) \mid \text{ Baseline covariates}\big]$$

will be calculated. Subsequently, the probability for each control group patient to remain on the assigned treatment will be estimated using additionally the time-varying seven-point ordinal scale and the viral load, and probabilities

$$p_{ij}^{*} := P\big[\text{No crossover for patient } i \text{ in follow} - \text{up period } [t_j, t_{j+1}) \mid \text{ Baseline and time} - \text{dependent covariates}\big]$$

will be given. Then, stabilized weights for each time period $[t_j, t_{j+1})$, j=1,… for each patient i will be calculated by $w_{ij} := \prod_{k=1}^{j} p_{ik} \,/\, \prod_{k=1}^{j} p_{ik}^{*}$. These are the so-called stabilized weights proposed by Robins et al. (2000), which ensure that the weights are not too extreme, i.e. not too far away from 1. The weights of crossover patients are set to zero after the crossover occurs. Patients from the treatment group will receive constant and equal weights of 1 since they do not crossover. Then, a cox proportional hazard model with the time-varying weights w_{ij} and the fixed factor treatment arm stratified for patient group will be calculated to estimate an adjusted hazard ratio for the treatment effect for the hypothetical scenario that no switching occurred.

7.4.4 Supplementary analysis 2

As another supplementary analysis intending to estimate the treatment effect in the hypothetical scenario which assumes that control patients had not switched to the treatment group, a rank preserving structural failure time (RPSFT) model will be fitted (Robins & Tsiatis, 1991). This model assumes a common treatment effect of the intervention, meaning that the treatment effect is the same for all individuals (with respect to time spent on treatment) regardless of when treatment is received, which is a strong and hardly verifiable assumption, thus requiring cautious interpretation of the modelled treatment effect.

As described by Latimer et al. (2017), the method splits the observed event time, T_i, for each patient into two, that is the event time when the patient is on the control treatment, T_{A_i} , and the event time when the patient is on the intervention treatment, T_{B_i}. For patients who are randomised to the intervention treatment, T_{A_i} is equal to zero. For patients randomised to the control group who do not switch onto the intervention (i.e. compliance is full in the control group), T_{B_i}. is equal to zero. However, for patients who switch treatments, both T_{A_i} and T_{B_i} will be greater than zero. The RPSFT method relates T_i to the counterfactual event time (U_i), i.e. the time an individual would have had, had he or she received the control treatment, with the following causal model: $U_i = T_{A_i} + e^{\psi_0} T_{B_i}$. The value of ψ is estimated using a grid search, and

the true value of $\psi = \psi_0$ is that for which $U_i(\psi)$ is independent of the randomised group. This will be assessed via a logrank test stratified for patient group. ψ will be determined using the approach by Danner & Sarkar (2018), and event times for the control group adjusted via this counterfactual model (i.e. a comparison of $e^{-\psi_0}U_i$ for patients in the intervention arm with U_i for patients in the control arm) will be assessed via Kaplan Meier Curves and a Cox regression model stratified for patient group, producing an adjusted hazard ratio.

This adjustment also needs to be considered when determining event status for crossover patients on the control arm, as the recovery time for patients who crossed over from the control arm is likely to be artificially prolonged via the RPSFT approach, leading to fewer events in the control arm due to the data cutoff at 84 days. A correction to minimize this effect in censoring time is mentioned in Robins and Tsiatis (1991), and they propose to use Min (C, C*exp(ψ)) as the date of administrative censoring, where C is the follow-up time for each patient. As ψ is expected to be positive for the primary endpoint of the RECOVER trial due to the expected benefit in recovery time, we will use Max(C, C*exp(ψ)) as the date of administrative censoring, thereby increasing the “hypothetical” follow-up time for the control arm. It should be noted that for the RPSFT analyses on overall survival, the originally proposed adjustment of Min (C, C*exp(ψ)) will be done.

7.4.5 Supplementary analysis 3

As a further supplementary analysis the analysis as described in chapter 7.3 will be conducted in the PPS as defined in chapter 4. A supplementary estimate will also be calculated based on the Cox regression model described in chapter 7.3. Analyses in the PPS don’t target any causal effect and are known to be biased, hence results of this supplementary analyses are of secondary importance to the interpretation of the trial results.

7.4.6 Supplementary analysis 4

As a further supplementary analysis the analysis as described in chapter 7.3 will be conducted in the as-treated set, in which patients who crossed over will be evaluated in the experimental group. A supplementary estimate will also be calculated based on the Cox regression model described in chapter 7.3.

7.4.7 Supplementary analysis 5

A further supplementary analysis will focus on the effect of cross-over. To evaluate the effect of cross-over of patients randomized into the Standard-treatment arm, a subset of patients eligible for cross-over is defined (still hospitalized at day 10, no improvement on the 7 point ordinal scale) and analyzed with regard to the primary and secondary endpoints according to plasma-treatment.

7.5 Further analyses based on the primary endpoint

7.5.1 Impact of timing of treatment

To assess the impact of the timing of treatment, a Cox regression model adjusting for the factor treatment and time between randomization and first dose of treatment, stratified for patient group, is conducted. The hazard ratio and a 95% profile-likelihood confidence interval for “time between randomization and first dose of treatment” will be obtained from the model.

7.5.2 Impact of type of treatment

To assess the impact of the type of treatment (convalescent plasma/vaccine-boosted plasma/standard of care), a Cox regression model adjusting for type of treatment, and stratified for patient group is conducted. The hazard ratios and 95% profile-likelihood confidence intervals for “convalescent plasma vs standard of care”, “vaccine-boosted plasma vs standard of care” and “convalescent plasma vs vaccine-boosted plasma” will be obtained from the model. Patients who did receive both convalescent and vaccine-boosted plasma will be analysed together with those patients who received only vaccine-boosted plasma. Additionally, the cumulative improvement curves are calculated using the Kaplan-Meier method together with 95% log-log-type confidence bands, and will be calculated separately for the three types of treatment.

7.5.3 Impact of type of COVID infection wave

To assess the impact of the type of COVID infection wave (second wave: 3.9.2020 – 14.02.2021/third wave: 15.02.2021- 27.06.2021/fourth wave: since 28.06.2021), the cumulative improvement curves per treatment and patient group for each wave are calculated using the Kaplan-Meier method together with 95% log-log-type confidence bands.

7.5.4 Impact of reception of monoclonal antibodies

In case that more than 10% of all patients received monoclonal antibodies, the impact of this treatment will be assessed by calculating the cumulative improvement curves per treatment and patient group for patients with and without monoclonal antibody treatment using the Kaplan-Meier method together with 95% log-log-type confidence bands, and will be calculated separately for the three waves of treatment.

7.5.5 Impact of virus variant

To assess the impact of the type of virus variant (Wild-type/Alpha/Delta/Omikron), the cumulative improvement curves per treatment and patient group for each variant are calculated using the Kaplan-Meier method together with 95% log-log-type confidence bands.

7.5.6 Impact of vaccination status

To assess the impact of the type of vaccination status (vaccinated/not vaccinated), the cumulative improvement curves per treatment and patient group for vaccinated and non-vaccinated patients are calculated using the Kaplan-Meier method together with 95% log-log-type confidence bands. Patients will be considered as vaccinated if they completed the cycle as per definition of the vaccine (1 vaccination for Johnson & Johnson vaccine, 2 vaccinations otherwise).

7.5.7 Impact of number of neutralizing antibodies at baseline

To assess the impact of the number of neutralizing antibodies at baseline (<1:40, ≥1:40), the cumulative improvement curves per treatment and patient group for each variant are calculated using the Kaplan-Meier method together with 95% log-log-type confidence bands.

Furthermore, the number of neutralizing antibodies at baseline will be included as continuous covariate in a Cox regression model additionally adjusting for treatment group, while stratifying for patient group. The hazard ratios and 95% profile-likelihood confidence intervals will be obtained from this model.

7.6 Analysis of the secondary endpoints

For all secondary endpoints, the analysis will be conducted based on the FAS and as a supplementary analysis based on the PPS.

7.6.1 Analysis of overall survival

Overall survival is analyzed by using a Cox regression model adjusted for the factor treatment and stratified by patient group, determining hazard ratios with 95% profile-likelihood confidence intervals and (descriptive) p-values. Survival estimates are calculated using the Kaplan-Meier method together with 95% log-log-type confidence bands, and the 28-day survival rate will be given for both treatment groups together with 95% log-log type confidence intervals. In addition, overall survival curves will also be calculated separately for each patient group per treatment group, and descriptive log-rank tests will be done to compare the two treatment groups for each patient group.

Analogously as for the supplementary analysis of the primary endpoint, an IPCW model and a RPSFT model will additionally be fitted for the overall survival to assess the potential treatment effect in the hypothetical scenario where patients did not switch from the control to the experimental arm.

7.6.2 Analysis of time until discharge

Time until discharge will be analyzed using a log-rank test stratified for the factor "patient group" and censoring patients who died from any cause at day 84 as described in chapter 7.3. Kaplan-Meier estimates for the cumulative discharge rate for both treatment groups together with 95% log-log-type confidence bands will be computed.

A Cox regression model, stratified for patient group, will be conducted to obtain an estimate for the hazard ratio for the treatment and a corresponding 95% profile-likelihood confidence interval.

IPCW and a RPSFT model will additionally be fitted for the endpoint 'time until discharge' to assess the potential treatment effect in the hypothetical scenario where patients did not switch from the control to the experimental arm.

7.6.3 Analysis of requirement of mechanical ventilation

The endpoint "requirement of mechanical ventilation (yes/no)" is analyzed by means of a logistic regression model adjusting for the factors treatment and patient group. If a patient who did not previously receive mechanical ventilation dies, this patient will be considered as having required mechanical ventilation. An estimate for the odds ratio for the factor treatment together with a 95% profile-likelihood confidence interval will be obtained from the model.

Also, absolute and relative frequencies will be given for this endpoint, together with 95% confidence intervals.

7.6.4 Analysis of SARS-CoV-2 viral load and antibody titres

Viral clearance (defined as having a viral load that is either non-measurable or <33) will be determined for every follow-up visit. Patients who died before experiencing a viral clearance will be regarded as non-cleared. Viral clearance at specific time points will be compared between the two treatment groups using a logistic regression model adjusting for the factors treatment and patient group.

Ct values for viral load will be determined as Ct=–log(viral load at follow-up/viral load at baseline) for days 5 and 10. Nonparametric van Elteren test will be used to compare the two treatment groups with regard to the Ct values while stratifying for patient group.

OD ratio and antibody titres will be evaluated on day 3 and 5 combined. In case that a measurement exists on both day 3 and day 5, the maximum will be used for analysis. The day 3/day 5 value will be descriptively assessed and compared to baseline. Nonparametric van Elteren test will be used to compare the two treatment groups with regard to the OD ratio and antibody titres while stratifying for patient group. Boxplots will be given for all time points for each treatment group to graphically investigate the Ct values, OD ratio, and antibody titres.

7.6.5 Analysis of cytokine levels

The cytokine levels will only be analysed descriptively. The ratio of the cytokine level at day 3/ day 4 / day 5 to the cytokine level at baseline will be calculated, and descriptive measures will be given for each treatment group.

7.7 Analysis of exploratory endpoints

All exploratory endpoints will be analyzed descriptively using the methods described in chapter 7.1. For endpoints that are assessed at multiple time points, each time point will be analyzed separately and line plots over time will be generated. The line plots will show the time point on the x-axis and either the mean value per group for parametically evaluated continuous endpoints, the median value per group for all non-parametrically evaluated continuous and ordinal endpoints or the relative frequency per group for binary endpoints on the y-axis.

The exploratory endpoint SOFA score will be analysed following the recommendations of Lambden et al. (2019). The highest possible score of 24, corresponding to the worst outcome, will be used for patients who died before the SOFA score could be assessed during follow-up. In case that one or more items of a SOFA assessment are missing, mean imputation will be used to derive the total score. In case that all items are missing, no imputation will be done.

7.8 Analysis of safety endpoints

The assessment of safety is based mainly on the frequency of adverse events and on the laboratory values Safety analysis is based on the safety population. Adverse events are summarized by presenting the number and percentage of patients having any adverse events or serious adverse events, and having each individual adverse event, and by determining and summarizing the maximum individual toxicity grade (over all forms of toxicity). Furthermore, the most common AEs (those occurring in at least 10% of the treatment group) are determined. Event rates are summarized along with two-sided Clopper-Pearson 95% confidence intervals and analyzed by (descriptive) chi-squared tests. Any other information collected (e.g. severity or relatedness to study drug) are summarized using the descriptive methods described in chapter 7.1.

All laboratory values on day 5 will be descriptively evaluated as continuous measures and tabulated per treatment group. To compare laboratory values between treatment groups, two-

sample t-tests will be conducted. In case a p-value smaller or equal to 0.05 is achieved, boxplots will additionally be given for this respective laboratory value.

7.9 Subgroup analyses

The primary endpoint and all secondary endpoints will be separately assessed for patient group 1, as well as for patient groups 1+2 combined.

8 Differences to trial protocol

Instead of recruiting the planned number of 174 patients, only 134 patients were enrolled to the trial as recruitment was stopped on January 20, 2022. The reason was a suspected inefficiency of the collected plasma bags for patients infected with the Omicron variant.

As viral load and antibody titres are not assumed to be normally distributed, they will be analysed using descriptive and non-parametric methods with no imputation being done for these endpoints.

9 Interpretation of results

As the planned number of n=174 was not reached, the study does not have the aspired power of 80% to detect the assumed difference between the two treatment groups with respect to the primary endpoint.

10 Data problems

In case that less than 60% of virus variants can be determined, a subgroup analysis based on this predictor (laid out in section 7.5.5) will not take place.

11 Estimands framework

In the Addendum to the ICH E9 guideline (final version), the estimands framework is recommended as clear and transparent definition of “what is to be estimated” (International Council for Harmonization 2019). The estimand is a construct of the treatment, the targeted population, the variable, a specification of how intercurrent events are handled, and a population-level summary measure.

11.1 Main estimand

The *main estimand* is described (referring to the primary efficacy analysis):

Treatment: Infusion of CP/PVP (on two days of intervention from two different donors) vs. standard of care

Population: The targeted population is defined through the in- and exclusion criteria.

Variable: Time from randomisation to clinical improvement within 84 days by two points on a seven point ordinal scale (as defined in chapter 5.1) or live discharge from the hospital

Intercurrent events:

- live discharge from the hospital is incorporated into the variable definition (composite strategy)
- death from any cause within 84 days after randomization without previous improvement is taken into account by censoring deceased patients at day 84 (composite strategy)
- treatment switch will be ignored (treatment policy strategy)

Population-level summary: Hazard ratio

12 Software

SAS Version 9.4 will be used for all statistical analyses. R (http://r-project.org) version 4.0.0 or higher as well as ggplot2 (Wickham 2016) can be used for the creation of figures.

13 References

Cao, B. et al. A Trial of Lopinavir-Ritonavir in Adults Hospitalized with Severe Covid-19. N Engl J Med, doi:10.1056/NEJMoa2001282 (2020).

Danner, B. J., & Sarkar, I. (2018). Implementing the Rank-Preserving Structural Failure Time Model in SAS® and R. Paper EP04, URL: https://www.pharmasug.org/proceedings/2018/EP/PharmaSUG-2018-EP04.pdf.

Lambden, S., Laterre, P.F., Levy, M.M. et al. The SOFA score—development, utility and challenges of accurate assessment in clinical trials. Crit Care 23, 374 (2019).

Latimer, N. R., Abrams, K. R., Lambert, P. C., Crowther, M. J., Wailoo, A. J., Morden, J. P., ... & Campbell, M. J. (2017). Adjusting for treatment switching in randomised controlled trials–a simulation study and a simplified two-stage method. Statistical methods in medical research, 26(2), 724-751.

Robins, J. M., Hernan, M. A., & Brumback, B. (2000). Marginal structural models and causal inference in epidemiology. Epidemiology, 11(5), 550-560.

Robins, J. M. & Finkelstein, D. M. (2000). Correcting for noncompliance and dependent censoring in an AIDS Clinical Trial with inverse probability of censoring weighted (IPCW) log-rank tests. Biometrics 56, 779-788,

Robins, J. and A.Tsiatis. (1991). Correcting for Non-Compliance in Randomized Trials Using Rank Preserving Structural Failure Time Models. Communication in Statistics – Theory and Methods 20(8):2609-2631.

Wickham H (2016). ggplot2: Elegant Graphics for Data Analysis. Springer-Verlag New York. ISBN 978-3-319-24277-4, https://ggplot2.tidyverse.org.

**RECOVER | Protocol No:** SARS-CoV-2CP-HD-001 **| EudraCT No.:** 2020-001632-10<br><br>**Changes to the SAP V1.0**		
**Content**	**Justification**	**Date**
Requirement of mechanical ventilation: Patients with a follow-up time of at most one day were excluded from the analysis, this was not pre-specified.	Duration was considered too short for outcome to occur	At time of analysis

Text supplement S2 Information on Plasma donation

Convalescent plasma was procured from individuals after a minimum of three weeks from symptom resolution throughout the duration of the trial. Plasma from vaccinated individuals (used since May 18, 2021) was obtained from donors with at least two doses of mRNA vaccines. Only plasma from consented donors with high levels of neutralizing antibodies against the prevailing variant at the time (titers ≥ 1:80; less than 25% of donors) at initial evaluation was used (details on the neutralizing assay are provided below). Plasma was later also characterized with NeutraLISA assay (Euroimmun, Lübeck, Germany) for comparability against measurements in patients. Plasma from COVID-19 convalescent patients or vaccinated healthy donors (CVP) was collected according to standard operating procedures for fresh-frozen plasma in accordance with national regulatory requirements and EU- Good Manufacturing Practice (GMP) Guidelines. A specific manufacturing license for plasma within the RECOVER trial was obtained from the regional authorities (Regierungspraesidium Tuebingen). Plasma was frozen and shipped to trial sites upon randomization of a patient into the plasma-arm of the study.

Text supplement S3 Live virus neutralization assay used for determination of neutralizing antibody titer in donors

VeroE6 cells were seeded into 96-well plates one day before the assay. Serial dilutions of sera starting from a 1:10 dilution were prepared in OptiMEM (ThermoFisher Scientific, Waltham, MA, USA) in a final volume of 75 µl and incubated with ~24,000 plaque-forming-units of SARS-CoV-2 (BavPat1/2020 strain, European Virus Archive) per well for 1 hour at 37 °C in a final volume of 150 µl. One third of each serum-virus mixture (50 µl) was then used for infection (final multiplicity of infection ~0.25). Infections were performed in duplicates. At 20 hours post-infection, cells were fixed with 5% formaldehyde and immunostained using a primary mouse antiserum binding to double-stranded RNA, a viral replication intermediate (J2 mouse antibody; Scicons, Szirák, Hungary) and a secondary anti-mouse horseradish peroxidase-coupled antibody (Merck, Darmstadt, Germany). The signal was developed using the KPL

SureBlueTM TMB peroxidase substrate (Seracare, Milford, MA, USA) and measured in a plate reader at a wavelength of 450 nm. Data were normalized to no-serum control (100%) and mock-infected control (0%). The neutralization (NT) titer was defined as the highest serum dilution resulting in more than 50% reduction of the normalized signal. Testing was performed against the most prevalent variant at the time (wildtype, alpha, delta).

Text supplement S4 Statistical analysis plan

SAP for Final Analysis

RECOVER

A Randomized Open label Phase-II Clinical Trial with or without Infusion of Plasma from Subjects after Convalescence of SARS-CoV-2 Infection in High-Risk Patients with Confirmed Severe SARS-CoV-2 Disease

Clinical Trial Code: SARS-CoV-2CP-HD-001

EudraCT No.: 2020-001632-10

SAP refers to Protocol version 3.0

1 Purpose of this SAP

In the trial protocol the assumptions, methods and procedures for the statistical evaluation are already described. The statistical analysis plan (SAP) aims to specify more precisely the evaluations carried out in the statistical analysis. This SAP refers to the final analysis after study completion, no interim analysis was planned or conducted.

2 Objective of the Trial

The primary objective of the trial is to assess the time from randomization until an improvement within 84 days defined as two points on a seven point ordinal scale (given in chapter 5.1) or live discharge from the hospital in high-risk patients (group 1 to group 4 as defined below) with SARS-CoV-2 infection requiring hospital admission by infusion of plasma from subjects after convalescence of SARS-CoV-2 infection/vaccination against SARS-CoV-2 or standard of care.

The four high-risk patient groups are:

1. Patients with pre-existing or concurrent hematological malignancy and/or active cancer therapy (including chemotherapy, radiotherapy, surgery) within the last 24 months or less.
2. Patients with chronic immunosuppression not meeting the criteria of group 1.
3. Patients of age ≥ 50 - 75 years meeting neither the criteria of group 1 nor group 2 and at least one of these criteria: Lymphopenia < 0.8 x G/l and / or D-dimer > 1µg/mL.
4. Patients of age ≥ 75 years meeting neither the criteria of group 1 nor group 2.

The link between the primary objective and the primary analysis is also described in the sense of the new estimands framework which is given in chapter 10.

Secondary objectives of the trial are:

- to assess overall survival, and the overall survival rate at 28, 56 and 84 days,
- to assess SARS-CoV-2 viral clearance and load as well as antibody titres,
- to assess percentage of patients that required mechanical ventilation, and

- to assess time from randomization until discharge
- to assess cytokine changes over time.

3 Study design

RECOVER is a randomized, open-label, multicenter phase II trial, designed to assess the clinical outcome of SARS-CoV-2 disease in high-risk patients (group 1 to group 4) following treatment with plasma from subjects after convalescence of a SARS-CoV-2 infection or after vaccination against SARS-CoV-2 compared with standard of care. Randomisation is done either into the experimental arm “infusion of convalescent plasma (CP) or vaccine-boosted plasma (=Post-Vaccination Plasma/PVP) ” or into the standard arm “standard of care” in a 1:1 ratio using block randomisation stratified for the factor “patient group”, defined by groups 1 to 4 as given in Chapter 2. The total planned sample size is n = 174 (84 per group).

4 Analysis sets

Full Analysis Set

The Full Analysis Set (FAS) includes all randomised patients with treatment groups assigned in accordance with the randomisation, regardless of the treatment actually received. Patients who were randomised but did not subsequently receive treatment are included in the FAS. The analysis of data using the FAS follows the Intention-to-Treat principles (ITT).

Per Protocol Set

In the per protocol set (PPS), patients with important protocol deviations (e.g. patients not meeting the inclusion/exclusion criteria, too early cross-over, reception of only one instead of two bags of infusion) are excluded. Analyses based on the PPS are known to be biased and are therefore of minor importance for the interpretation of the results and only serve as supplementary analyses. The determination of patients with major protocol deviations will be done in a data review meeting which will take place before database lock.

Safety Set

All enrolled patients who received treatment are subjected to the safety analysis. Patients will be evaluated with regard to the treatment actually received, meaning that patients who cross over from the control to the experimental arm will be evaluated in the experimental arm at day 10 (+ 2 days) onwards.

5 Definitions of endpoints to be analysed

5.1 Primary Endpoint

The primary efficacy endpoint is time from randomisation to clinical improvement within 84 days by two points on a seven point ordinal scale (in comparison to the status at randomisation) or live discharge from the hospital. If a patient needs to be transferred to another hospital, he or she will not be considered as discharged. If a patient is transferred to a rehabilitation clinic or nursing home, he or she will be considered as discharged. The categories of the seven point ordinal scale are:

1. Not hospitalized with resumption of normal activities
2. Not hospitalized, but unable to resume normal activities
3. Hospitalized, not requiring supplemental oxygen
4. Hospitalized, requiring supplemental oxygen
5. Hospitalized, requiring nasal high-flow oxygen therapy, or noninvasive mechanical ventilation
6. Hospitalized, requiring ECMO, invasive mechanical ventilation, or both
7. Death

5.2 Secondary Endpoints

Secondary endpoints are:

- Overall survival, defined as the time from randomization until death from any cause
- 28-day, 56-day and 84-day overall survival rate
- Requirement of mechanical ventilation at any time during hospital stay (yes/no)

- Time from randomization until discharge. If a patient needs to be transferred to another hospital, he or she will not be considered as discharged. If a patient is transferred to a rehabilitation clinic or nursing home, he or she will be considered as discharged.
- SARS-CoV-2 viral clearance and load as well as antibody titres (until hospital discharge on day 1, 3, 5, 10, 14, 28, 56 and 84), comprising:
    - Result of smear (negative / positive / invalide)
    - CT-value
    - Serodiagnostic by ELISA (OD ratio)
    - Neutralizing antibody titre
- Cytokine profiles

5.3 Exploratory Endpoints

Exploratory endpoints are:

- Vital signs (on day 1, 3, 5, 10 and 14), comprising:
    - ECOG performance status (ordinal, 0 to 4)
    - Systolic blood pressure in mmHg
    - Diastolic blood pressure in mmHg
    - Pulse rate in beats/min
    - Respiratory rate in breaths/min
- Seven point ordinal scale as defined in chapter 5.1 (until hospital discharge daily from day 1-28, weekly from day 29-55 and on day 56 and 84)
- Sequential Organ Failure Assessment (SOFA) Score (until hospital discharge on day 1 and 10)
- Oxygen saturation:
    - Partial pressure of oxygen (PaO_2) in mmHg
    - Partial pressure of carbon dioxide ($PaCO_2$) in mmHg
    - ECMO (yes / no)

    - Type of ventilation (no / nasal high-flow oxygen therapy / noninvasive mechanical ventilation / invasive ventilation / tracheotomy / ambient air / standard oxygen sources)
    - Amount of oxygen in litres/min
    - Oxygen saturation (SaO_2) in percent
    - Amount of supplemental oxygen that is required to keep SaO_2 above 94% in litres/min
    - Fraction of inspired oxygen (FiO_2) in percent
- Hematology (until hospital discharge on day 1, 3, 5, 10, 14, 28, 56 and 84), comprising:
    - Hemoglobin
    - RBC
    - WBC
    - Thrombocytes
- Blood chemistry and coagulation (until hospital discharge on day 1, 3, 5, 10, 14, 28, 56 and 84), comprising:
    - BUN
    - Creatinine
    - Albumin
    - AST/SGOT
    - ALT/SGPT
    - Total bilirubin
    - GGT
    - AP
    - LDH
    - Sodium
    - Potassium
    - Magnesium
    - Calcium

  - Uric acid
  - Troponin
  - CK
  - CK-MB
  - PTT
  - ATIII
  - D-dimer
  - Fibrinogen
  - Ferritin (measured at day 1 only)
  - Transferrin (measured at day 1 only)
  - Transferrin saturation (measured at day 1 only)
  - CRP
  - Total protein (measured at day 1 only)
  - IL6 (measured at day 1 only)
  - Procalcitonin (measured at day 1 only)
  - Total IgG (measured at day 1 only)
  - IgA (measured at day 1 only)
  - IgM (measured at day 1 only)
  - Lactate
  - INR
- Assessment of concomitant medications
- Signs/symptoms (on day 10 in case of cross-over), comprising:
  - Cough (yes / no)
  - Fever (yes / no)
  - Myalgia (yes / no)
  - Fatigue (yes / no)
  - Diarrhoea (yes / no)
  - Vomiting (yes / no)

    - Headache (yes / no)
    - Abdominal pain (yes / no)
    - Nausea (yes / no)
    - Olfactory or taste disorder (yes / no)
    - Dyspnoea (yes / no)
    - Confusion (yes / no)
- Physical examination (on day 10 in case of cross-over), comprising:
    - General appearance (normal / abnormal)
    - Lung (normal / abnormal)
    - Cardiac auscultation (normal / abnormal)
    - Cardiac percussion (normal / abnormal)
    - Abdominal auscultation (normal / abnormal)
    - Abdominal percussion (normal / abnormal)
    - Abdominal palpation (normal / abnormal)
    - Palpation of lymph node sites (normal / abnormal)
    - Neurological examination (normal / abnormal)
    - Peripheral edema (normal / abnormal)
- ECG, comprising
    - ECG abnormal / abnormal
    - Clinically significant (yes / no)
    - Heart rate in beats/min
    - Rhythm (normal sinus / other)

5.4 Safety Endpoints

Safety endpoints comprise:

- Adverse Events (AEs)
- Severity of AEs
- Serious adverse events (SAEs)

- Relation of AEs to the study treatment
- Dose modifications for toxicity
- Discontinuation of study treatment

6 Methods for Withdrawals and Missing Data

Withdrawals according to study protocol will not be excluded unless the patient withdrew his/her consent declining explicitly the use of the data for analysis (s. informed consent). As long as a written informed consent is available without denied permission to evaluate the documented data, the data already collected as well as follow-up data will be included in the final analysis.

For patients with incomplete follow-up, time to last follow-up date is used as the censoring time in the analysis of time-to-event data. Otherwise, no imputation of missing data will be conducted.

7 Statistical methods

The statistical evaluation is carried out under the supervision of the Supervising Statistician. Statistical analysis is based on the International Conference on Harmonization (ICH) Guidelines "Structure and Content of Clinical Study Reports" and "Statistical Principles for Clinical Trials". All statistical procedures are done according to the current Standard Operating Procedures (SOPs) of the Institute of Medical Biometry, University of Heidelberg (IMBI).

7.1 Descriptive methods

A CONSORT flow diagram will be created to display the progress of all participants through the trial. This includes the number of patients assessed for eligibility and the number of patients excluded because they did not meet inclusion criteria, declined to participate, or any other reason, the number of randomized patients (per group), and number of missed intervention assignments / incomplete interventions. In addition, the number of patients in the FAS and PPS will be given and reported reasons for exclusion from the PP set will be summarized per treatment group.

Continuous variables will be described using number of observations, mean, standard deviation, median, Q1, Q3, minimum, maximum and number of missing values. For categorical variables, absolute and relative frequencies will be given with missing values being reported as a separate category. Percentages for categorical variables will be based on all non-missing values in the respective groups. Time to event endpoints will be described by Kaplan-Maier estimates (including the numbers at risk for different time points), median survival time (or other quantiles if median survival time cannot be calculated), number of events and number of censorings. The descriptive methods described above will be used separately for each treatment group.

The test to be applied is specified in the description of the analyses of the respective endpoint. If a test is conducted to compare treatment groups, point estimates with respective 95% confidence intervals will be provided additionally to the descriptive p-value. For the chi-square test, the difference of rates and/or the risk ratio will be given where appropriate, for the t-test the difference between means will be provided and for the Mann-Whitney U test, the Hodges-Lehmann estimate will be presented as point estimate.

Descriptive results will be presented in tables, appropriately supplemented with figures if specified in the respective analysis section. Survival endpoints will always be supplemented with a Kaplan-Meier survival curve. Listings will be used to present data collected in free-text formats.

7.2 Baseline characteristics

The following baseline characteristics will be collected and described in the FAS set, the PP set and the safety set:

- Demographics, comprising:
    - Sex (male / female)
    - Year of birth
    - Ethnic origin (asian / black / caucasian/white / hispanic / other)
- Medical history

- Haematological malignancy
    - Solid tumor
    - Immunosuppression due to disease (immunodeficiency)
    - Diabetes
    - Cardiovascular disease
    - Obesity
    - Lung disease
- – Assessment of Concomitant medications
    - COVID19 therapy from 7 days before study entry up to day 10
        - Steroids
        - Remdesivir or other antiviral drugs
        - Monoclonal antibodies
        - Tociluzimab
        - Ruxolitinib
- Smoking history, comprising
    - Yes / non-smoker / ex-smoker
    - Smoking duration in years
    - Smoking amount in packs per day
- Signs/symptoms, comprising:
    - Cough (yes / no)
    - Fever (yes / no)
    - Myalgia (yes / no)
    - Fatigue (yes / no)
    - Diarrhoea (yes / no)
    - Vomiting (yes / no)
    - Headache (yes / no)
    - Abdominal pain (yes / no)
    - Nausea (yes / no)

    - Olfactory or taste disorder (yes / no)
    - Dyspnoea (yes / no)
    - Confusion (yes / no)
- Vital signs, comprising:
    - ECOG performance status (ordinal, 0 to 4)
    - Body temperature in degrees Celsius
    - Systolic blood pressure in mmHg
    - Diastolic blood pressure in mmHg
    - Pulse rate in beats/min
    - Respiratory rate in breaths/min
- Physical examination, comprising:
    - General appearance (normal / abnormal)
    - Lung (normal / abnormal)
    - Cardiac auscultation (normal / abnormal)
    - Cardiac percussion (normal / abnormal)
    - Abdominal auscultation (normal / abnormal)
    - Abdominal percussion (normal / abnormal)
    - Abdominal palpation (normal / abnormal)
    - Palpation of lymph node sites (normal / abnormal)
    - Neurological examination (normal / abnormal)
    - Peripheral edema (normal / abnormal)
- Clinical Frailty Scale (very fit / well / managing well / vulnerable / mildly frail / moderately frail / severely frail / very severely frail / terminally ill)
- Seven point ordinal scale as defined in chapter 5.1
- Oxygen saturation
    - Partial pressure of oxygen (PaO_2) in mmHg
    - Partial pressure of carbon dioxide ($PaCO_2$) in mmHg
    - ECMO (yes / no)

    - Type of ventilation (no / nasal high-flow oxygen therapy / noninvasive mechanical ventilation / invasive ventilation / tracheotomy / ambient air / standard oxygen sources)
    - Amount of oxygen in litres/min (will only be evaluated for any time point if patient does not simultaneously receive any oxygen)
    - Oxygen saturation (SaO_2) in percent (will only be evaluated for any time point if patient does not simultaneously receive any oxygen)
    - Amount of supplemental oxygen that is required to keep SaO_2 above 94% in litres/min
    - Fraction of inspired oxygen (FiO_2) in percent
- ECG, comprising
    - ECG abnormal / abnormal
    - Clinically significant (yes / no)
    - Heart rate in beats/min
    - Rhythm (normal sinus / other)
- Hematology (until hospital discharge on day 1, 3, 5, 10, 14, 28, 56 and 84), comprising:
    - Hemoglobin
    - RBC
    - WBC
    - Thrombocytes
- Blood chemistry and coagulation (until hospital discharge on day 1, 3, 5, 10, 14, 28, 56 and 84), comprising:
    - BUN
    - Creatinine
    - Albumin
    - AST/SGOT
    - ALT/SGPT
    - Total bilirubin

- GGT
- AP
- LDH
- Sodium
- Potassium
- Magnesium
- Calcium
- Uric acid
- Troponin
- CK
- CK-MB
- PTT
- ATIII
- D-dimer
- Fibrinogen
- Ferritin (measured at day 1 only)
- Transferrin (measured at day 1 only)
- Transferrin saturation (measured at day 1 only)
- CRP
- Total protein (measured at day 1 only)
- IL6 (measured at day 1 only)
- Procalcitonin (measured at day 1 only)
- Total IgG (measured at day 1 only)
- IgA (measured at day 1 only)
- IgM (measured at day 1 only)
- Lactate
- INR

- Urinalysis, comprising:

    - pH-value
    - Glucose
    - Protein
- Other virology, comprising:
    - Anti-HAV (negative / positive)
    - Anti-HBV (negative / positive)
    - Anti-HCV IgG (negative / positive)
    - HIV (negative / positive)

Baseline characteristics will be summarized for each treatment group separately using the methods described in Section 7.1.

7.3 Primary analysis

The primary analysis will assess the null hypothesis “the cumulative improvement curves for the primary endpoint in the experimental and control arm are equal”, i.e.

H_0: $S_E = S_C$

against the alternative hypothesis

H_1: $S_E \neq S_C$

at a two-sided significance level of α=5%. This will be achieved by using the log-rank test stratified for the factor “patient group” as used in the randomisation procedure. The event “death from any cause” will be handled by censoring those patients at day 84 (in analogy to the approach of Cao et al, NEJM 2020). Using this approach ensures that deceased patients are considered as “not improved” over the whole observation period of 84 days. The primary analysis will be based on the FAS and handles intercurrent events as described in chapter 11.1.

The main estimate (corresponding to the main estimand as described in chapter 11.1) for the hazard ratio for treatment group will be computed using a Cox regression model stratifying for the factor “patient group” together with a 95% profile-likelihood confidence interval. The

cumulative improvement curves are calculated using the Kaplan-Meier method together with 95% log-log-type confidence bands, and will be calculated separately for both treatment groups. In addition, cumulative improvement curves will also be calculated separately for each patient group per treatment group, and descriptive log-rank tests will be done to compare the cumulative improvement groups for each patient group. Patients who drop out during the trial or are lost to follow-up are taken into account as censored observations.

7.4 Sensitivity and supplementary analyses

All sensitivity analyses are conducted in the FAS and handle intercurrent events as described in chapter 11.1. If a supplementary analysis is conducted in a different analysis set or handles an intercurrent event differently this is explicitly mentioned.

7.4.1Sensitivity analysis 1

An unstratified log-rank test will be conducted, which is based on the assumption of identical baseline risk in the different patient groups. As a sensitivity estimate a hazard ratio for the treatment group based on an unstratified Cox-regression will be calculated together with a 95% profile-likelihood confidence interval.

7.4.2 Sensitivity analysis 2

A Gray’s test (Gray, 1988) stratified for “patient group” will be conducted where the death from any cause without previous improvement is treated as a competing risk. As a sensitivity estimate a subdistribution hazard based on a stratified Fine and Gray’s competing risk regression using PROC PHREG will be calculated together with 95% confidence intervals.

7.4.3 Supplementary analysis 1

A supplementary analysis of the primary endpoint will involve the inverse probability censoring weighting (IPCW) approach to estimate a treatment effect in the hypothetical scenario that patients from the control group had not switched to the experimental group (Robins & Finkelstein (2000)). This type of model requires to estimate the probability for a patient to

switch to the experimental group based on (time-dependent) covariates, which in our case will be the baseline factor “patient group”, together with the time-varying factors “seven point ordinal scale measuring clinical improvement” and “viral load”. For this analysis, patients who cross over to the standard treatment will be considered as censored observations. Thus, however, patients who crossover due to poor prognosis will not contribute to the same degree as patients with a favourable prognosis less likely to switch. Thus, in order to adjust for informative censoring in case of treatment switch, patients in the control group will receive a weighting, such that uncensored patients with a high crossover risk due to unfavourable prognostic factors will receive higher weights than patients with a low crossover risk due to favourable prognostic factors. It should be noted that this method relies on the assumption that there are no unmeasured confounders influencing the probability to crossover, which is hardly verifiable, results will therefore needed to be cautiously interpreted.

o conduct the IPCW method, firstly, the data needs to be transformed into a long format, and the follow-up period needs to be split up in time intervals. In a first step, the probability to remain on the assigned treatment will be estimated for the control group using a repeated measures logistic regression model via PROC GENMOD with an unstructured covariance matrix, including only the patient group and the baseline ordinal scale as predictors. This model will estimate the probabilities

$$p_{ij} := P\left[\text{No crossover for patient } i \text{ in follow} - \text{up period } \left[t_j, t_{j+1}\right) \mid \text{ Baseline covariates}\right]$$

will be calculated. Subsequently, the probability for each control group patient to remain on the assigned treatment will be estimated using additionally the time-varying seven-point ordinal scale and the viral load, and probabilities

$$p_{ij}^{*} := P\left[\text{No crossover for patient } i \text{ in follow} - \text{up period } \left[t_j, t_{j+1}\right) \mid \text{ Baseline and time} - \text{dependent covariates}\right]$$

will be given. Then, stabilized weights for each time period $\left[t_j, t_{j+1}\right)$, j=1,... for each patient i will be calculated by $w_{ij} := \prod_{k=1}^{j} p_{ik} \,/ \prod_{k=1}^{j} p_{ik}^{*}$. These are the so-called stabilized weights proposed by Robins et al. (2000), which ensure that the weights are not too extreme, i.e. not

too far away from 1. The weights of crossover patients are set to zero after the crossover occurs. Patients from the treatment group will receive constant and equal weights of 1 since they do not crossover. Then, a cox proportional hazard model with the time-varying weights w_{ij} and the fixed factor treatment arm stratified for patient group will be calculated to estimate an adjusted hazard ratio for the treatment effect for the hypothetical scenario that no switching occurred.

7.4.4 Supplementary analysis 2

As another supplementary analysis intending to estimate the treatment effect in the hypothetical scenario which assumes that control patients had not switched to the treatment group, a rank preserving structural failure time (RPSFT) model will be fitted (Robins & Tsiatis, 1991). This model assumes a common treatment effect of the intervention, meaning that the treatment effect is the same for all individuals (with respect to time spent on treatment) regardless of when treatment is received, which is a strong and hardly verifiable assumption, thus requiring cautious interpretation of the modelled treatment effect.

As described by Latimer et al. (2017), the method splits the observed event time, T_i, for each patient into two, that is the event time when the patient is on the control treatment, T_{A_i} , and the event time when the patient is on the intervention treatment, T_{B_i}. For patients who are randomised to the intervention treatment, T_{A_i} is equal to zero. For patients randomised to the control group who do not switch onto the intervention (i.e. compliance is full in the control group), T_{B_i}. is equal to zero. However, for patients who switch treatments, both T_{A_i} and T_{B_i} will be greater than zero. The RPSFT method relates T_i to the counterfactual event time (U_i), i.e. the time an individual would have had, had he or she received the control treatment, with the following causal model: $U_i = T_{A_i} + e^{\psi_0} T_{B_i}$. The value of ψ is estimated using a grid search, and the true value of $\psi = \psi_0$ is that for which $U_i(\psi)$ is independent of the randomised group. This will be assessed via a logrank test stratified for patient group. ψ will be determined using the approach by Danner & Sarkar (2018), and event times for the control group adjusted via this counterfactual model (i.e. a comparison of $e^{-\psi_0} U_i$ for patients in the intervention arm with U_i

for patients in the control arm) will be assessed via Kaplan Meier Curves and a Cox regression model stratified for patient group, producing an adjusted hazard ratio.

This adjustment also needs to be considered when determining event status for crossover patients on the control arm, as the recovery time for patients who crossed over from the control arm is likely to be artificially prolonged via the RPSFT approach, leading to fewer events in the control arm due to the data cutoff at 84 days. A correction to minimize this effect in censoring time is mentioned in Robins and Tsiatis (1991), and they propose to use Min (C, C*exp(ψ)) as the date of administrative censoring, where C is the follow-up time for each patient. As ψ is expected to be positive for the primary endpoint of the RECOVER trial due to the expected benefit in recovery time, we will use Max(C, C*exp(ψ)) as the date of administrative censoring, thereby increasing the "hypothetical" follow-up time for the control arm. It should be noted that for the RPSFT analyses on overall survival, the originally proposed adjustment of Min (C, C*exp(ψ)) will be done.

7.4.5 Supplementary analysis 3

As a further supplementary analysis the analysis as described in chapter 7.3 will be conducted in the PPS as defined in chapter 4. A supplementary estimate will also be calculated based on the Cox regression model described in chapter 7.3. Analyses in the PPS don't target any causal effect and are known to be biased, hence results of these supplementary analyses are of secondary importance to the interpretation of the trial results.

7.4.6 Supplementary analysis 4

As a further supplementary analysis the analysis as described in chapter 7.3 will be conducted in the as-treated set, in which patients who crossed over will be evaluated in the experimental group. A supplementary estimate will also be calculated based on the Cox regression model described in chapter 7.3.

7.4.7 Supplementary analysis 5

A further supplementary analysis will focus on the effect of cross-over. To evaluate the effect of cross-over of patients randomized into the Standard-treatment arm, a subset of patients eligible for cross-over is defined (still hospitalized at day 10, no improvement on the 7 point ordinal scale) and analyzed with regard to the primary and secondary endpoints according to plasma-treatment.

7.5 Further analyses based on the primary endpoint

7.5.1 Impact of timing of treatment

To assess the impact of the timing of treatment, a Cox regression model adjusting for the factor treatment and time between randomization and first dose of treatment, stratified for patient group, is conducted. The hazard ratio and a 95% profile-likelihood confidence interval for “time between randomization and first dose of treatment” will be obtained from the model.

7.5.2 Impact of type of treatment

To assess the impact of the type of treatment (convalescent plasma/vaccine-boosted plasma/standard of care), a Cox regression model adjusting for type of treatment, and stratified for patient group is conducted. The hazard ratios and 95% profile-likelihood confidence intervals for “convalescent plasma vs standard of care”, “vaccine-boosted plasma vs standard of care” and “convalescent plasma vs vaccine-boosted plasma” will be obtained from the model. Patients who did receive both convalescent and vaccine-boosted plasma will be analysed together with those patients who received only vaccine-boosted plasma. Additionally, the cumulative improvement curves are calculated using the Kaplan-Meier method together with 95% log-log-type confidence bands, and will be calculated separately for the three types of treatment.

7.5.3 Impact of type of COVID infection wave

To assess the impact of the type of COVID infection wave (second wave: 3.9.2020 – 14.02.2021/third wave: 15.02.2021- 27.06.2021/fourth wave: since 28.06.2021), the cumulative improvement curves per treatment and patient group for each wave are calculated using the Kaplan-Meier method together with 95% log-log-type confidence bands.

7.5.4 Impact of reception of monoclonal antibodies

In case that more than 10% of all patients received monoclonal antibodies, the impact of this treatment will be assessed by calculating the cumulative improvement curves per treatment and patient group for patients with and without monoclonal antibody treatment using the Kaplan-Meier method together with 95% log-log-type confidence bands, and will be calculated separately for the three waves of treatment.

7.5.5 Impact of virus variant

To assess the impact of the type of virus variant (Wild-type/Alpha/Delta/Omikron), the cumulative improvement curves per treatment and patient group for each variant are calculated using the Kaplan-Meier method together with 95% log-log-type confidence bands.

7.5.6 Impact of vaccination status

To assess the impact of the type of vaccination status (vaccinated/not vaccinated), the cumulative improvement curves per treatment and patient group for vaccinated and non-vaccinated patients are calculated using the Kaplan-Meier method together with 95% log-log-type confidence bands. Patients will be considered as vaccinated if they completed the cycle as per definition of the vaccine (1 vaccination for Johnson & Johnson vaccine, 2 vaccinations otherwise).

7.5.7 Impact of number of neutralizing antibodies at baseline

To assess the impact of the number of neutralizing antibodies at baseline (<1:40, ≥1:40), the cumulative improvement curves per treatment and patient group for each variant are calculated using the Kaplan-Meier method together with 95% log-log-type confidence bands.

Furthermore, the number of neutralizing antibodies at baseline will be included as continuous covariate in a Cox regression model additionally adjusting for treatment group, while stratifying for patient group. The hazard ratios and 95% profile-likelihood confidence intervals will be obtained from this model.

7.6 Analysis of the secondary endpoints

For all secondary endpoints, the analysis will be conducted based on the FAS and as a supplementary analysis based on the PPS.

7.6.1 Analysis of overall survival

Overall survival is analyzed by using a Cox regression model adjusted for the factor treatment and stratified by patient group, determining hazard ratios with 95% profile-likelihood confidence intervals and (descriptive) p-values. Survival estimates are calculated using the Kaplan-Meier method together with 95% log-log-type confidence bands, and the 28-day survival rate will be given for both treatment groups together with 95% log-log type confidence intervals. In addition, overall survival curves will also be calculated separately for each patient group per treatment group, and descriptive log-rank tests will be done to compare the two treatment groups for each patient group.

Analogously as for the supplementary analysis of the primary endpoint, an IPCW model and a RPSFT model will additionally be fitted for the overall survival to assess the potential treatment effect in the hypothetical scenario where patients did not switch from the control to the experimental arm.

7.6.2 Analysis of time until discharge

Time until discharge will be analyzed using a log-rank test stratified for the factor “patient group” and censoring patients who died from any cause at day 84 as described in chapter 7.3. Kaplan-Meier estimates for the cumulative discharge rate for both treatment groups together with 95% log-log-type confidence bands will be computed.

A Cox regression model, stratified for patient group, will be conducted to obtain an estimate for the hazard ratio for the treatment and a corresponding 95% profile-likelihood confidence interval.

IPCW and a RPSFT model will additionally be fitted for the endpoint ‘time until discharge’ to assess the potential treatment effect in the hypothetical scenario where patients did not switch from the control to the experimental arm.

7.6.3 Analysis of requirement of mechanical ventilation

The endpoint “requirement of mechanical ventilation (yes/no)” is analyzed by means of a logistic regression model adjusting for the factors treatment and patient group. If a patient who did not previously receive mechanical ventilation dies, this patient will be considered as having required mechanical ventilation. An estimate for the odds ratio for the factor treatment together with a 95% profile-likelihood confidence interval will be obtained from the model.

Also, absolute and relative frequencies will be given for this endpoint, together with 95% confidence intervals.

7.6.4 Analysis of SARS-CoV-2 viral load and antibody titres

Viral clearance (defined as having a viral load that is either non-measurable or <33) will be determined for every follow-up visit. Patients who died before experiencing a viral clearance will be regarded as non-cleared. Viral clearance at specific time points will be compared between the two treatment groups using a logistic regression model adjusting for the factors treatment and patient group.

Ct values for viral load will be determined as Ct=–log(viral load at follow-up/viral load at baseline) for days 5 and 10. Nonparametric van Elteren test will be used to compare the two treatment groups with regard to the Ct values while stratifying for patient group.

OD ratio and antibody titres will be evaluated on day 3 and 5 combined. In case that a measurement exists on both day 3 and day 5, the maximum will be used for analysis. The day 3/day 5 value will be descriptively assessed and compared to baseline. Nonparametric van Elteren test will be used to compare the two treatment groups with regard to the OD ratio and antibody titres while stratifying for patient group. Boxplots will be given for all time points for each treatment group to graphically investigate the Ct values, OD ratio, and antibody titres.

7.6.5 Analysis of cytokine levels

The cytokine levels will only be analysed descriptively. The ratio of the cytokine level at day 3/ day 4 / day 5 to the cytokine level at baseline will be calculated, and descriptive measures will be given for each treatment group.

7.7 Analysis of exploratory endpoints

All exploratory endpoints will be analyzed descriptively using the methods described in chapter 7.1. For endpoints that are assessed at multiple time points, each time point will be analyzed separately and line plots over time will be generated. The line plots will show the time point on the x-axis and either the mean value per group for parametrically evaluated continuous endpoints, the median value per group for all non-parametrically evaluated continuous and ordinal endpoints or the relative frequency per group for binary endpoints on the y-axis.

The exploratory endpoint SOFA score will be analysed following the recommendations of Lambden et al. (2019). The highest possible score of 24, corresponding to the worst outcome, will be used for patients who died before the SOFA score could be assessed during follow-up. In case that one or more items of a SOFA assessment are missing, mean imputation will be used to derive the total score. In case that all items are missing, no imputation will be done.

7.8 Analysis of safety endpoints

The assessment of safety is based mainly on the frequency of adverse events and on the laboratory values Safety analysis is based on the safety population. Adverse events are summarized by presenting the number and percentage of patients having any adverse events or serious adverse events, and having each individual adverse event, and by determining and summarizing the maximum individual toxicity grade (over all forms of toxicity). Furthermore, the most common AEs (those occurring in at least 10% of the treatment group) are determined. Event rates are summarized along with two-sided Clopper-Pearson 95% confidence intervals and analyzed by (descriptive) chi-squared tests. Any other information collected (e.g. severity or relatedness to study drug) are summarized using the descriptive methods described in chapter 7.1.

All laboratory values on day 5 will be descriptively evaluated as continuous measures and tabulated per treatment group. To compare laboratory values between treatment groups, two-sample t-tests will be conducted. In case a p-value smaller or equal to 0.05 is achieved, boxplots will additionally be given for this respective laboratory value.

7.9 Subgroup analyses

The primary endpoint and all secondary endpoints will be separately assessed for patient group 1, as well as for patient groups 1+2 combined.

8 Differences to trial protocol

Instead of recruiting the planned number of 174 patients, only 134 patients were enrolled to the trial as recruitment was stopped on January 20, 2022. The reason was a suspected inefficiency of the collected plasma bags for patients infected with the Omicron variant.

As viral load and antibody titres are not assumed to be normally distributed, they will be analysed using descriptive and non-parametric methods with no imputation being done for these endpoints.

9 Interpretation of results

As the planned number of n=174 was not reached, the study does not have the aspired power of 80% to detect the assumed difference between the two treatment groups with respect to the primary endpoint.

10 Data problems

In case that less than 60% of virus variants can be determined, a subgroup analysis based on this predictor (laid out in section 7.5.5) will not take place.

11 Estimands framework

In the Addendum to the ICH E9 guideline (final version), the estimands framework is recommended as clear and transparent definition of “what is to be estimated” (International Council for Harmonization 2019). The estimand is a construct of the treatment, the targeted population, the variable, a specification of how intercurrent events are handled, and a population-level summary measure.

11.1 Main estimand

The *main estimand* is described (referring to the primary efficacy analysis):

Treatment: Infusion of CP/PVP (on two days of intervention from two different donors) vs. standard of care

Population: The targeted population is defined through the in- and exclusion criteria.

Variable: Time from randomisation to clinical improvement within 84 days by two points on a seven point ordinal scale (as defined in chapter 5.1) or live discharge from the hospital

Intercurrent events:

- live discharge from the hospital is incorporated into the variable definition (composite strategy)
- death from any cause within 84 days after randomization without previous improvement is taken into account by censoring deceased patients at day 84 (composite strategy)

- treatment switch will be ignored (treatment policy strategy)

Population-level summary: Hazard ratio

12 Software

SAS Version 9.4 will be used for all statistical analyses. R (http://r-project.org) version 4.0.0 or higher as well as ggplot2 (Wickham 2016) can be used for the creation of figures.

13 References

Cao, B. et al. A Trial of Lopinavir-Ritonavir in Adults Hospitalized with Severe Covid-19. N Engl J Med, doi:10.1056/NEJMoa2001282 (2020).

Danner, B. J., & Sarkar, I. (2018). Implementing the Rank-Preserving Structural Failure Time Model in SAS® and R. Paper EP04, URL: https://www.pharmasug.org/proceedings/2018/EP/PharmaSUG-2018-EP04.pdf.

Lambden, S., Laterre, P.F., Levy, M.M. et al. The SOFA score—development, utility and challenges of accurate assessment in clinical trials. Crit Care 23, 374 (2019).

Latimer, N. R., Abrams, K. R., Lambert, P. C., Crowther, M. J., Wailoo, A. J., Morden, J. P., ... & Campbell, M. J. (2017). Adjusting for treatment switching in randomised controlled trials–a simulation study and a simplified two-stage method. Statistical methods in medical research, 26(2), 724-751.

Robins, J. M., Hernan, M. A., & Brumback, B. (2000). Marginal structural models and causal inference in epidemiology. Epidemiology, 11(5), 550-560.

Robins, J. M. & Finkelstein, D. M. (2000). Correcting for noncompliance and dependent censoring in an AIDS Clinical Trial with inverse probability of censoring weighted (IPCW) log-rank tests. Biometrics 56, 779-788,

Robins, J. and A.Tsiatis. (1991). Correcting for Non-Compliance in Randomized Trials Using Rank Preserving Structural Failure Time Models. Communication in Statistics – Theory and Methods 20(8):2609-2631.

Wickham H (2016). ggplot2: Elegant Graphics for Data Analysis. Springer-Verlag New York. ISBN 978-3-319-24277-4, https://ggplot2.tidyverse.org.

CONSORT 2010 checklist of information to include when reporting a randomised trial*

Section/Topic	Item No	Checklist item	Reported on page No
**Title and abstract**			
	1a	Identification as a randomised trial in the title	1
	1b	Structured summary of trial design, methods, results, and conclusions (for specific guidance see CONSORT for abstracts)	3
**Introduction**			
Background and objectives	2a	Scientific background and explanation of rationale	4
	2b	Specific objectives or hypotheses	4
**Methods**			
Trial design	3a	Description of trial design (such as parallel, factorial) including allocation ratio	11-13
	3b	Important changes to methods after trial commencement (such as eligibility criteria), with reasons	na
Participants	4a	Eligibility criteria for participants	11,12
	4b	Settings and locations where the data were collected	11
Interventions	5	The interventions for each group with sufficient details to allow replication, including how and when they were actually administered	12, 13
Outcomes	6a	Completely defined pre-specified primary and secondary outcome measures, including how and when they were assessed	13-15
	6b	Any changes to trial outcomes after the trial commenced, with reasons	na
Sample size	7a	How sample size was determined	14,15
	7b	When applicable, explanation of any interim analyses and stopping guidelines	14
Randomisation:			
Sequence generation	8a	Method used to generate the random allocation sequence	12,13
	8b	Type of randomisation; details of any restriction (such as blocking and block size)	12,13
Allocation concealment mechanism	9	Mechanism used to implement the random allocation sequence (such as sequentially numbered containers), describing any steps taken to conceal the sequence until interventions were assigned	12,13
Implementation	10	Who generated the random allocation sequence, who enrolled participants, and who assigned participants to interventions	12,13
Blinding	11a	If done, who was blinded after assignment to interventions (for example, participants, care providers, those	

		assessing outcomes) and how	15
	11b	If relevant, description of the similarity of interventions	na
Statistical methods	12a	Statistical methods used to compare groups for primary and secondary outcomes	14,15
	12b	Methods for additional analyses, such as subgroup analyses and adjusted analyses	15
**Results**			
Participant flow (a diagram is strongly recommended)	13a	For each group, the numbers of participants who were randomly assigned, received intended treatment, and were analysed for the primary outcome	5,6 and 17-22 (tables 1,2)
	13b	For each group, losses and exclusions after randomisation, together with reasons	5,6 and Fig.1
Recruitment	14a	Dates defining the periods of recruitment and follow-up	5
	14b	Why the trial ended or was stopped	6
Baseline data	15	A table showing baseline demographic and clinical characteristics for each group	17-20 (table1) Suppl. Appendix pg. 48-51 (table S5)
Numbers analysed	16	For each group, number of participants (denominator) included in each analysis and whether the analysis was by original assigned groups	6-8
Outcomes and estimation	17a	For each primary and secondary outcome, results for each group, and the estimated effect size and its precision (such as 95% confidence interval)	6-8
	17b	For binary outcomes, presentation of both absolute and relative effect sizes is recommended	6-8, 21-22
Ancillary analyses	18	Results of any other analyses performed, including subgroup analyses and adjusted analyses, distinguishing pre-specified from exploratory	6-8
Harms	19	All important harms or unintended effects in each group (for specific guidance see CONSORT for harms)	8,9 and Suppl. Appendix pg.56-60 (tables S7,S8)
**Discussion**			
Limitations	20	Trial limitations, addressing sources of potential bias, imprecision, and, if relevant, multiplicity of analyses	11
Generalisability	21	Generalisability (external validity, applicability) of the trial findings	12
Interpretation	22	Interpretation consistent with results, balancing benefits and harms, and considering other relevant evidence	9,10

**Other information**			
Registration	23	Registration number and name of trial registry	3, 16
Protocol	24	Where the full trial protocol can be accessed, if available	5, Suppl: study protocol and 26 Ref. No. 31
Funding	25	Sources of funding and other support (such as supply of drugs), role of funders	16

*We strongly recommend reading this statement in conjunction with the CONSORT 2010 Explanation and Elaboration for important clarifications on all the items. If relevant, we also recommend reading CONSORT extensions for cluster randomised trials, non-inferiority and equivalence trials, non-pharmacological treatments, herbal interventions, and pragmatic trials. Additional extensions are forthcoming: for those and for up to date references relevant to this checklist, see www.consort-statement.org.
