# Supplementary material for: Anti-SARS-CoV-2 antibody-containing plasma improves outcome in patients with hematologic or solid cancer and severe COVID-19: a randomized clinical trial
Source: Nat Cancer. 2022 Dec 29;4(1):96–107. doi: 10.1038/s43018-022-00503-w (PMC9886549; doi:10.1038/s43018-022-00503-w)
Supplement: Supplementary file 2 — Reporting Summary [file 43018_2022_503_MOESM2_ESM.pdf]

nature portfolio

Corresponding author(s): Carsten Müller-Tidow

Last updated by author(s): Nov 22, 2022

Reporting Summary

Nature Portfolio wishes to improve the reproducibility of the work that we publish. This form provides structure for consistency and transparency in reporting. For further information on Nature Portfolio policies, see our Editorial Policies and the Editorial Policy Checklist.

Statistics

For all statistical analyses, confirm that the following items are present in the figure legend, table legend, main text, or Methods section.

n/a	Confirmed	
☐	☒	The exact sample size (*n*) for each experimental group/condition, given as a discrete number and unit of measurement
☐	☒	A statement on whether measurements were taken from distinct samples or whether the same sample was measured repeatedly
☐	☒	The statistical test(s) used AND whether they are one- or two-sided<br>*Only common tests should be described solely by name; describe more complex techniques in the Methods section.*
☐	☒	A description of all covariates tested
☐	☒	A description of any assumptions or corrections, such as tests of normality and adjustment for multiple comparisons
☐	☒	A full description of the statistical parameters including central tendency (e.g. means) or other basic estimates (e.g. regression coefficient) AND variation (e.g. standard deviation) or associated estimates of uncertainty (e.g. confidence intervals)
☐	☒	For null hypothesis testing, the test statistic (e.g. *F*, *t*, *r*) with confidence intervals, effect sizes, degrees of freedom and *P* value noted<br>*Give P values as exact values whenever suitable.*
☒	☐	For Bayesian analysis, information on the choice of priors and Markov chain Monte Carlo settings
☐	☒	For hierarchical and complex designs, identification of the appropriate level for tests and full reporting of outcomes
☒	☐	Estimates of effect sizes (e.g. Cohen's *d*, Pearson's *r*), indicating how they were calculated

Our web collection on statistics for biologists contains articles on many of the points above.

Software and code

Policy information about availability of computer code

Data collection	All findings including clinical and laboratory data were documented by the investigator or an authorised member of the study team in the patient's medical record and in the electronic case report forms (eCRFs). The investigator at the clinical site was responsible for ensuring that all sections of the eCRFs were completed correctly and that entries could have been verified against source data. The eCRFs had to be filled out according to the specified CRF Completion Guidelines. The correctness of entries in the eCRFs was confirmed by dated signature of the responsible local principal investigator or deputy principal investigator.<br>Data entries underwent an automatic online check for plausibility and consistency. In case of implausibility, 'warnings' were produced. A responsible investigator was obliged either to correct the implausible data or to confirm its authenticity, and to give appropriate explanation. If not corrected, the data was flagged, enabling a convenient check of all questionable entries. A responsible monitor checked all flagged data and generates questions that were sent back to the responsible investigator. The investigator had to resolve all 'discrepancies'.<br>Further checks for plausibility, consistency, and completeness of data were performed after completion of the study. Queries were generated on the basis of these checks, combined with a visual control by a responsible monitor/data manager.<br><br>eCRF: ClinCase Software Version 2.7.0.3
Data analysis	Analyses were performed using the software package SAS® Version 9.4 (SAS Inc., Cary/NC, USA), R Base (v4.0, https://r-project.org) and Graphpad Prim version 9.

For manuscripts utilizing custom algorithms or software that are central to the research but not yet described in published literature, software must be made available to editors and reviewers. We strongly encourage code deposition in a community repository (e.g. GitHub). See the Nature Portfolio guidelines for submitting code & software for further information.

Data

Policy information about availability of data

All manuscripts must include a data availability statement. This statement should provide the following information, where applicable:

- Accession codes, unique identifiers, or web links for publicly available datasets
- A description of any restrictions on data availability
- For clinical datasets or third party data, please ensure that the statement adheres to our policy

Source data for Fig. 1-4 and Extended Data Fig. S1-S7 have been provided as Source Data files. Due to the data protection regulations of the informed consent data it cannot be made available in a publicly accessible repository. The data can only be made available from the authors on reasonable request. Please contact Carsten.müller-tidow@med.uni-heidelberg.de for data availability.

Human research participants

Policy information about studies involving human research participants and Sex and Gender in Research.

Reporting on sex and gender	In this trial we included patients fullfilling pre-defined inclusion criteria with severe COVID-19 performing convenience sampling. We included only high-risk patients with severe COVID-19. Patients with severe COVID-19 are predominantly male. Accordingly, 43 patients of the included patients were female (32.1%).<br>Gender was not assessed in this trial.
Population characteristics	A total of 136 patients meeting eligibility criteria were randomized. Two patients were excluded due to absence of a signed informed consent and withdrawal of consent after signature, respectively.<br>Average age was 69 years (range, 36-95 years) and 43 patients were female (32.1%). ECOG performance status (median 2), clinical frailty scale (median 3) and time from symptom onset to randomization (median 7.0) were similar in both arms. The allocation of patients to the predefined high-risk patient groups were: group-1 42% (n=56, Figure S1), group-2 12% (n=16), group-3 27% (n=36), group-4 19% (n=26). The most common cancers were B-cell malignancies (n=20), acute myeloid leukemia/ myelodysplastic syndrome (n=12) and myeloma (n=11), and solid cancer (n=9). Two patients suffered from Hodgkin´s lymphoma and one patient each from chronic myeloid leukemia or T-cell lymphoma . The most common cause for chronic immunosuppression in group-2 was solid organ transplantation (n=12). In group-3, 27 patients showed lymphopenia and 21 patients elevated d-dimers, both criteria were present in 12 patients.
Recruitment	Patients were recruited at the respective centers according to the inclusion and exclusion criteria of the clinical trial protocol. Data collection and analysis were not performed blind to the conditions of the experiments.
Ethics oversight	The study was approved by the federal institute for vaccines and biomedicines (Paul-Ehrlich-Institute) and the ethics committee Heidelberg. Regulatory authority requirements with respect to plasma manufacturing according to §67 Arzneimittelgesetz (Germany) and §13 GCP-V were met. Ethics committees for the recruiting centers approved the respective recruiting center.<br>All included patients provided written informed consent.

Note that full information on the approval of the study protocol must also be provided in the manuscript.

Field-specific reporting

Please select the one below that is the best fit for your research. If you are not sure, read the appropriate sections before making your selection.

☒ Life sciences ☐ Behavioural & social sciences ☐ Ecological, evolutionary & environmental sciences

For a reference copy of the document with all sections, see nature.com/documents/nr-reporting-summary-flat.pdf

Life sciences study design

All studies must disclose on these points even when the disclosure is negative.

Sample size	To detect the assumed difference between treatment groups using a log-rank test comparing the cumulative improvement curves for the primary endpoint using a significance level of 5% (two-sided) with a power of 80%, a total number of 174 patients is required for the entire trial (87 patients per group) when additionally considering a dropout rate of 5%, meaning that n=164 patients who do not prematurely drop out of the study are required to be enrolled. The required number of events which was calculated using the formula by Schoenfeld, amounts to 142. It is expected that adjusting for the covariate “patient group” in the analysis will lead to an increase in power. Sample size calculation was done conservatively assuming an equally long follow-up period of 38 days for every patient. Sample size calculation was done using RPACT v 2.0.6.<br>For the sample size calculation, it was assumed that there would be a consistent median time to improvement and overall survival rate across all 4 patient groups. Since this might not necessarily be the case, and it could be likely that patients in group 1 and 2 might have a shorter median time to improvement, we conducted a simulation study to explore the robustness of our model under varying assumptions for the median event times. For our simulation study, we assumed exponentially distributed event times, with a median time to improvement of 16

days across all patient groups in the control group (medC1= medC2 = medC3 = medC4=16), while we assumed differing median times to improvement in the patient groups 1 & 2 compared to patient groups 3 & 4 (medE1= medE2 , medE3 = medE4) in the experimental group. We considered a sample size of n=164 evaluable patients for the whole trial, with 42 patients belonging to groups 1 and 3 each, and 40 patients belonging to groups 2 and 4 each corresponding to patient group membership probability of about 25% for all four groups. Also, we assumed a 38-day mortality rate of 20% in the control arm, while we assumed that the corresponding mortality rate in the experimental arm amounted to 13% (corresponding to a HR for overall survival of 1.6) across all four patient strata assuming exponentially distributed survival times. We simulated 10,000 trials per scenario corresponding to a maximum standard error of sqrt(0.5*0.5/10000)=0.005 for the simulated power.
We used a stratified log-rank test to assess whether the cumulative improvement curves in the two treatment groups (experimental/control) would be equal at a two-sided significance level of α=0.05 stratifying for the factor "patient group" for analysis. The analysis of the simulated datasets was done using the PROC LIFETEST procedure in SAS v9.4. The trial was terminated early after enrollment after enrolment of 77% of the target population.

Data exclusions

Two patients were excluded due to absence of a signed informed consent and withdrawal of consent after signature, respectively.

Replication

The findings are derived from unique patient samples combined with observed clinical data and cannot be replicated.

Randomization

A computer-generated randomisation list was created. Participants were assigned random numbers based on consecutive enrolment.
A subject was considered enrolled when he or she had signed the Informed consent form. The patient received a screening number at the clinical site (number of site plus number of patient in ascending order, e. g. 001-001 for the first enrolled patient at site 01) via registration in the eCRF system (www.xxx.). The screening number was used to identify the subject throughout the clinical study and was used on all study documentation related to the subject.

All subjects randomised received a unique randomisation number. All patients in the experimental group had to start CP treatment within 7 days of randomisation. Patients withdrawn from the trial retain their Patient ID and randomisation number.

Blinding

The study was open-label.

Reporting for specific materials, systems and methods

We require information from authors about some types of materials, experimental systems and methods used in many studies. Here, indicate whether each material, system or method listed is relevant to your study. If you are not sure if a list item applies to your research, read the appropriate section before selecting a response.

Materials & experimental systems

n/a	Involved in the study	
☐	☒	Antibodies
☐	☒	Eukaryotic cell lines
☒	☐	Palaeontology and archaeology
☒	☐	Animals and other organisms
☐	☒	Clinical data
☒	☐	Dual use research of concern

Methods

n/a	Involved in the study	
☒	☐	ChIP-seq
☒	☐	Flow cytometry
☒	☐	MRI-based neuroimaging

Antibodies

Antibodies used

Primary antibodies:
1. Sino Biological SARS- coV/ SARS-coV-2 nucleocapsid antibody, mouse; catalogue number: 40143-MM05; current lot no. MA15JA2101-T
2. Scicons anti-dsRNA mAb SCICONS J2, catalogue number: 10010500; lot no unknown
Secondary antibody:
Sigma- Aldrich anti- mouse- IgG (whole molecule) – peroxidase conjugated; antibody generated in goat; catalogoue number: A4416-5X1ML; lot no. unknown

Validation

Statements of the manufactures website, relevant citations:
Primary Antibodies: Anti-nucleocapsid: RRID: AB_2827977 and https://www.sinobiological.com/antibodies/cov-nucleocapsid-40143-mm05 and Anti-dsRNA antibody: RRID: AB_2651015 and https://nordicmubio.com/products/mouse-anti-double-stranded-rna-j2/10010500
Secondary antibody: https://www.sigmaaldrich.com/DE/de/product/sigma/a4416

Eukaryotic cell lines

Policy information about cell lines and Sex and Gender in Research

Cell line source(s)	VeroE6 cells:<br>- cell line source: ATCC Cat.#CRL-1586
Authentication	Since the cell line was newly obtained from ATCC, we did not confirm authenticity
Mycoplasma contamination	The cells are tested for mycoplasma contamination in 3-months intervals using the MycoAlert kit (Lonza; catalogue number LT07-118)
Commonly misidentified lines<br>(See ICLAC register)	na

Clinical data

Policy information about clinical studies

All manuscripts should comply with the ICMJE guidelines for publication of clinical research and a completed CONSORT checklist must be included with all submissions.

Clinical trial registration	https://www.clinicaltrialsregister.eu/ctr-search/trial/2020-001632-10/DE
Study protocol	The study protocol is provided as supplementary data with all changes included.
Data collection	Fifteen trial sites in Germany enrolled study participants (10 university and 5 urban hospitals, table S1 and S3). Data was collected using eCRFs. Monitoring was performed according to the study protocol and clinical trial regulations. Data collection and analysis were not performed blind to the conditions of the experiments. The first patient was randomized on September 3rd, 2020, the last patient was enrolled in January 2022 when the omicron variant became dominant in Germany and the trial was terminated early.
Outcomes	The primary outcome was time to improvement on a 7 point ordinal scale which is routinely used for COVID-19 patients. Patients were evaluated daily until discharge. For details see protocol. Secondary outcomes are detailed in the manuscript and in the study trial protocol. Secondary endpoints were overall survival (time from randomization until death from any cause, applying the same censoring rules as the primary endpoint for withdrawal of informed consent, loss to follow-up and administrative censoring at day 84); antibody titers; requirement of mechanical ventilation at any time during the hospital stay; time from randomization until live hospital discharge (applying the same censoring rules as the primary endpoint for withdrawal of informed consent, loss to follow-up and administrative censoring at day 84, as well censoring patients who died from any cause at day 84 analogously to the primary endpoint).
